# Assessing the Ecological Risks of Per‐ and Polyfluoroalkyl Substances: Current State‐of‐the Science and a Proposed Path Forward

**DOI:** 10.1002/etc.4869

**Published:** 2020-11-06

**Authors:** Gerald T. Ankley, Philippa Cureton, Robert A. Hoke, Magali Houde, Anupama Kumar, Jessy Kurias, Roman Lanno, Chris McCarthy, John Newsted, Christopher J. Salice, Bradley E. Sample, Maria S. Sepúlveda, Jeffery Steevens, Sara Valsecchi

**Affiliations:** ^1^ Great Lakes Toxicology and Ecology Division, US Environmental Protection Agency Duluth Minnesota USA; ^2^ Science and Risk Assessment Division, Environment and Climate Change Canada, Gatineau Quebec Canada; ^3^ DuPont, Wilmington Delaware USA; ^4^ Aquatic Contaminants Research Division, Environment and Climate Change Canada, Montreal Quebec Canada; ^5^ Land and Water, Commonwealth Scientific and Industrial Research Organisation Urrbrae South Australia Australia; ^6^ The Ohio State University, Columbus Ohio USA; ^7^ Jacobs Engineering, Boston Massachusetts USA; ^8^ Ramboll, Emeryville California USA; ^9^ Department of Biological Sciences, Towson University Towson Maryland USA; ^10^ Ecological Risk, Rancho Murieta California USA; ^11^ Department of Forestry and Natural Resources, Purdue University West Layette Indiana USA; ^12^ US Geological Survey, Columbia Environmental Research Center Columbia Missouri USA; ^13^ Water Research Institute, National Research Council Brugherio Monza and Brianza Italy

**Keywords:** Per‐ and poly‐fluoroalkyl substances, Ecological risk, Review, Strategy

## Abstract

Per‐ and poly‐fluoroalkyl substances (PFAS) encompass a large, heterogenous group of chemicals of potential concern to human health and the environment. Based on information for a few relatively well‐understood PFAS such as perfluorooctane sulfonate and perfluorooctanoate, there is ample basis to suspect that at least a subset can be considered persistent, bioaccumulative, and/or toxic. However, data suitable for determining risks in either prospective or retrospective assessments are lacking for the majority of PFAS. In August 2019, the Society of Environmental Toxicology and Chemistry sponsored a workshop that focused on the state‐of‐the‐science supporting risk assessment of PFAS. The present review summarizes discussions concerning the ecotoxicology and ecological risks of PFAS. First, we summarize currently available information relevant to problem formulation/prioritization, exposure, and hazard/effects of PFAS in the context of regulatory and ecological risk assessment activities from around the world. We then describe critical gaps and uncertainties relative to ecological risk assessments for PFAS and propose approaches to address these needs. Recommendations include the development of more comprehensive monitoring programs to support exposure assessment, an emphasis on research to support the formulation of predictive models for bioaccumulation, and the development of in silico, in vitro, and in vivo methods to efficiently assess biological effects for potentially sensitive species/endpoints. Addressing needs associated with assessing the ecological risk of PFAS will require cross‐disciplinary approaches that employ both conventional and new methods in an integrated, resource‐effective manner. *Environ Toxicol Chem* 2021;40:564–605. © 2020 The Authors. *Environmental Toxicology and Chemistry* published by Wiley Periodicals LLC on behalf of SETAC. This article has been contributed to by US Government employees and their work is in the public domain in the USA.

## BACKGROUND AND INTRODUCTION

Per‐ and poly‐fluoroalkyl substances (PFAS) have achieved prominence in terms of public visibility and regulatory concern not seen since endocrine‐disrupting chemicals were identified as a potential widespread risk to human health and the environment more than 2 decades ago (Hotchkiss et al. 2008). Different PFAS have been employed, in some cases for more than 70 yr, for a variety of consumer and industrial applications such as nonstick and protective coatings, manufacturing lubricants, and dispersants (Interstate Technology Regulatory Council 2020a). An additional use of certain PFAS has been as fire suppressors in aqueous film‐forming foams (AFFFs), which can be an important source of the chemicals to some environments. Concern for PFAS in many parts of the world has emphasized possible human health effects, particularly from the standpoint of contaminated drinking water, but there is also concern for ecological issues associated with these chemicals (McCarthy et al. 2017). For example, some PFAS have been listed as persistent organic pollutants (POPs) under the Stockholm Convention based on both potential human health and ecosystem effects (United Nations Environment Programme 2019). One challenge for scientists and risk assessors addressing PFAS involves identification of the actual universe of chemicals of concern. The most inclusive definition and associated categorization of chemical structures of PFAS is provided by the Organisation for Economic Co‐operation and Development (OECD; 2018a), and includes more than 4000 compounds. Although different regulatory/jurisdictional constructs may refine/modify the number of PFAS of potential concern (e.g., ~1200 PFAS are listed on the US Environmental Protection Agency [USEPA; 2020a] Toxic Substances Control Act Inventory, with ~600 of these considered “active”), the fact remains that whatever definition or list is employed, there is a substantial diversity of chemical structures and properties that may need to be considered in terms of ecological effects, often with little or no empirical fate or toxicity data. (Note: a glossary of PFAS giving the nomenclature used in the present review is presented in the Supplemental Data SI‐1).

There is a range of risk assessment scenarios—and associated data and research needs—relevant to the potential ecological effects of PFAS. For example, there is a need to assess PFAS in both prospective and retrospective assessments. Certain methods/tools are needed for both approaches. As a simple example, in vivo assays with potentially sensitive species and endpoints could be used to generate data either for a PFAS‐contaminated sample from the environment (retrospective assessment) or for a PFAS not yet released to the environment (prospective assessment). However, there are also some differences in the data and tools required for the 2 different assessment scenarios. For example, prospective assessments dealing with chemicals for which few empirical data exist usually employ predictive methods such as computational models to estimate environmental persistence, fate, or potential toxicity. In contrast, retrospective assessments often employ empirical data from the field to directly quantify chemical fate and biological effects.

Assessment tools and resultant data for PFAS also require flexibility with respect to the spatial scale of an ecological assessment, which might range from localized “hot spots” (e.g., near fire‐fighting training) to more global contamination resulting from atmospheric transport or ocean circulation. Another critical consideration is that the chemicals both occur and often enter the environments as poorly characterized complex mixtures of parent molecules and their precursors, degradation products, and metabolites. Mixture assessment is hardly a new concern in the field of ecotoxicology, but the nature of PFAS use in hundreds of commercial products and the potential for widespread occurrence highlights mixtures as an important consideration in developing approaches to estimating risk.

There are several broad needs relative to assessing the ecological risks of PFAS; 4 of the most pressing are: 1) development of empirical physicochemical property data to support testing and development of predictive models; 2) identification of susceptible species and toxicity endpoints as a basis for selecting appropriate in vitro and in vivo assays/toxicity tests; 3) development of effects‐based toxicity endpoint values (e.g., screening levels, benchmarks, criteria) in different matrices (water, sediment, soil, tissue); and 4) identification of methods to efficiently measure or predict the bioaccumulation and biomagnification potential of PFAS. A significant uncertainty in the identification of sensitive species and endpoints as well as derivation of benchmarks arises from limitations in the number of PFAS and taxa for which empirical laboratory or field data exist. Although there may be adequate toxicological information to employ “traditional” approaches to develop effects benchmarks for a few high‐visibility PFAS (e.g., perfluorooctane sulfonate [PFOS] and perfluorooctanoate [PFOA]), there are not sufficient data to derive toxicity effects values for the great majority of PFAS currently present in or potentially entering the environment. Finally, from the standpoint of bioaccumulation, it is widely acknowledged that the types of models developed and successfully used to predict the bioaccumulation (and biomagnification) of nonionic organic chemicals based on lipid partitioning are not suitable for most PFAS due to differences in basic physicochemical properties. Consequently, there is a pressing need for approaches to estimate/screen the bioaccumulation potential of PFAS for both retrospective and prospective scenarios.

A recent workshop sponsored by the Society of Environmental Toxicology and Chemistry (SETAC) focused on the fate, exposure, and effects of PFAS in the environment in the context of human health and ecological risks (Johnson et al. 2020). The present review describes the outcome of discussions by a workgroup considering ecological impacts of PFAS and addresses what currently is known concerning environmental exposure and effects, significant data gaps and uncertainties, and strategies and tools to help address these uncertainties in the context of the various risk assessment needs described above.

## PRIORITIZING PFAS FOR MONITORING AND TESTING

The first step in either prospective or retrospective risk assessments is problem formulation. A critical component of problem formulation involves identification and prioritization of those chemicals with the most potential to cause unacceptable effects on human health or the environment. Chemical prioritization is an important aspect of many chemical regulatory programs around the globe, serving as the basis for impact evaluations, priority setting, and regulatory action (Bu et al. 2013). Given the very large number of PFAS of possible ecological concern, prioritization is critically important in terms of ensuring that limited monitoring and testing resources are used in an optimal manner.

A variety of decision‐support tools are available for use in the screening and prioritization of chemicals (Gauthier et al. 2015). One of the oldest examples of chemical prioritization is the United Nations POPs program, in which chemical persistence, bioaccumulation, toxicity, and potential for long‐range transport are employed as prioritization criteria (Lallas 2001). The USEPA ToxCast program, which uses computational modeling, high‐throughput testing (HTT) assays, and toxicogenomic tools, is an example of a 21st century approach to chemical prioritization (e.g., Dix et al. 2007). Examples of other major regulatory frameworks that incorporate chemical prioritization include the European Chemicals Agency (ECHA) Registration, Evaluation, Authorisation, and Restriction (REACH) program, the USEPA Endocrine Disruptor Screening and Testing and Toxic Substances Control Act programs, the Health Canada and Environment and Climate Change Canada's Domestic Substances List categorization activity and Chemicals Management Plan, and various REACH‐like programs in countries such as South Korea, Taiwan, Turkey, and so on. Although the exact details of the prioritization schemes used in these programs may differ, there are many commonalities, including consideration of a substance's physicochemical properties, environmental fate, hazard (ecological and human health), volumes in use (typically as a surrogate for exposure or indication of the potential for widespread environmental distribution), use patterns, and frequently, some level of assessment of risk. The latter aspect of a prioritization program (i.e., evaluation of risk) typically synthesizes the other categories of information to provide a science‐based prioritization that identifies those substances most likely to present some level of risk to human health and the environment and/or that require the generation of additional data to more completely assess potential risks.

The following is a brief discussion of the various types of information or data concerning PFAS that may be useful for prioritization in the context of assessing ecological risk.

### Production technique, production volume, and product use

Chemical production techniques and volumes influence the nature and concentrations of PFAS that may enter the environment. Different PFAS have been manufactured for over 70 yr and are present in almost every aspect of our daily lives. Three techniques have been used to manufacture PFAS: electrochemical fluorination (ECF), telomerization, and oligomerization (Organisation for Economic Co‐operation and Development 2018b), with ECF and telomerization used most frequently. The ECF process was licensed in the 1940s and typically results in odd‐ and even‐numbered carbon chain length mixtures comprised of approximately 70% linear and 30% branched substances. Telomerization was developed in the 1970s and results in principally even‐numbered linear carbon chain length substances. Products containing PFAS may include the intentionally manufactured substances as well as unreacted raw materials, byproducts, and processing aids. The OECD has identified over 4700 PFAS‐related CAS numbers (Organisation for Economic Co‐operation and Development 2018b). Production volume is frequently used as a surrogate for exposure in assessment or prioritization schemes. Total global production volumes for individual PFAS may not be readily available in some instances. Recently, data were reported by Dreveton (2016) for 2 groups of PFAS, which suggested that the global production of fluoropolymers and fluoroelastomers in the 2012 to 2013 timeframe was approximately 273 000 and 30 000 metric tonnes, respectively. The ECHA REACH program, which has been in place since 2010, provides an example of available data for the production volumes of some PFAS manufactured or imported into the European Union. Searching the ECHA registered chemicals webpage using the terms “perfluoro” and “polyfluoro” yields a list of approximately 150 substances. Evaluation of this list indicates that approximately 40% are intermediates or “Notification of New Substances” registrations for which volumes do not need to be reported. Approximately 25% of the substances have registration volumes less than10 tonnes/yr, approximately 15 to 20% have volumes between 10 and 100 tonnes/yr, and the remaining 15 to 20% have registration volumes of >100 tonnes/yr. There are also other examples of programs that collect production volume information for some PFAS (e.g., Chemical Data Reporting associated with Toxic Substances Control Act in the United States), which can provide information useful for assessing potential environmental occurrence.

How a substance is used will dictate in what manner and where it enters the environment, a critical consideration relative to prioritization in the context of potential exposure. Different PFAS have been used for a wide variety of applications. The PFAS are often divided into 2 subclasses, polymeric and nonpolymeric. Nonpolymeric PFAS include perfluoroalkyl acids (PFAAs), perfluoroalkane sulfonyl fluoride‐derived substances, fluorotelomer‐based substances, and per‐and poly‐fluoroalkyl ether‐based substances (Organisation for Economic Co‐operation and Development 2013). The polymeric PFAS subclass includes fluoropolymers (e.g., polytetrafluoroethylene, polyvinylidene fluoride), side‐chain fluorinated polymers, and perfluoropolyethers. In the nonpolymer subclass (which includes surfactants), PFAS are used for a variety of applications/industries: aviation, aerospace and defense, biocides, construction products, electronics, fire‐fighting, household products, metal plating, oil production and mining, and industrial polymer production. Applications for the PFAS polymer subclass include automotive, aviation, aerospace and defense, construction, electronics and semiconductors, energy production, fire‐fighting, food processing and packaging, household products, medical items, paper and packaging, textiles, leather, and apparel (Organisation for Economic Co‐operation and Development 2013).

### Physicochemical properties

Prioritization of PFAS in terms of potential exposure must consider that the substances come in every physical form (i.e., gas, liquid, solid), and may exhibit different characteristics even within a given physical form, for example, nonvolatile versus extremely volatile liquids. As might be expected for a diverse set of hundreds to thousands of substances, PFAS can have vastly different physicochemical properties even when they are closely related structurally. An example of this disparity across a range of homologous PFAS is provided by Krafft and Riess (2015). These authors present data demonstrating that for a homologous series of fluorotelomer alcohols with carbon chain lengths from 4 to10, the experimental water solubility, air/water partition coefficient, octanol/water partition coefficient, octanol/air partition coefficient, and organic carbon/water partition coefficients can vary by 2 to 5 orders of magnitude. This variability in physicochemical properties can also be observed within and across other PFAS classes and will yield vastly different transport and fate profiles and relevant environmental compartments for assessment if substances are released to the environment.

In addition to affecting the transport and fate of substances released to the environment, differences in physicochemical properties will also influence the uptake of PFAS by aquatic and terrestrial organisms. One of the principal concerns related to the environmental presence of PFAS is that they are unlikely to undergo ultimate biodegradation or mineralization (Blum et al. 2015). Some PFAS undergo no degradation at all, whereas others typically degrade to terminal products that do not further degrade and, thus, potentially continue to increase in concentration in various environmental matrices. Although the bioaccumulation profiles of these terminal degradation products may differ, with some bioaccumulating (e.g., perfluorodecanoic acid [PFDA]) and some not (Conder et al. 2008), the broader concern is related to environmental concentrations that could potentially increase to levels that might cause adverse ecological effects.

### Persistence and distribution: Parents, precursors, and degradation products

Parent substances historically have been the main target of regulatory assessments, although some programs in the United States and Europe have begun to request information on major degradation products for some chemicals. These requests have typically been related to situations in which environmental degradation (abiotic, biotic) may yield products that are more persistent or bioaccumulative than parent/precursor substances. This situation can exist for PFAS where the original substance (e.g., the 8:2 fluorotelomer alcohol [8:2 FTOH]) can yield substances that are far more persistent, such as PFAAs (e.g., PFOA). Particularly for perfluorinated carboxylic acids (PFCAs) and perfluorinated sulfonic acids (PFSAs), it is important to remember that they can be both parent substances and terminal degradation products.

The presence of the parent substances and terminal degradation products in environmental matrices will depend on the emission profiles of the relevant substances, their physicochemical properties, and the relevant mode of degradation (i.e., abiotic vs biotic). As an example, manufacturing operations may result in the discharge of polymer processing aids in manufacturing effluents and/or via stack emissions with concomitant advective downstream transport and, potentially, long‐range atmospheric transport (Barton et al. 2007, 2008). Atmospheric deposition and/or removal in wastewater treatment plant biosolids with subsequent land application of biosolids can result in contamination of both soil and groundwater/drinking water resources (Venkatesan and Halden 2013).

An excellent overview of the environmental fate and transport of PFAS is presented by the Interstate Technology and Regulatory Council (2020b). The 2 broad classes of PFAS that typically are of greatest interest in terms of their environmental fate and transport include per‐ and polyfluoroalkyl acids (especially surfactants) and side‐chain fluorinated polymers. These classes contain substances (e.g., fluorotelomers) that can degrade to terminal products (e.g., PFCAs such as PFOA, and PFSAs such as PFOS). They also include polymer processing aids (e.g., per‐ and polyfluoroalkyl ether carboxylates such as hexafluoropropylene oxide dimer acid [GenX]) as well as other types of PFAAs (e.g., perfluorophosphonic and perfluorphosphinic acids) that are substances of considerable interest to both researchers and regulators. Terminal degradation products such as PFCAs (e.g., perfluorohexanoic acid [PFHxA]) and PFSAs (e.g., perfluorohexane sulfonate [PFHxS]) can be water soluble, mobile in soils, and very persistent; they may undergo long‐range transport in both water and aerosols/air, and are not typically metabolized in organisms (Henry et al. 2018). In contrast, fluoropolymers and perfluoropolyethers are high‐molecular‐weight, chemically inert materials. Fluoropolymers have limited water solubility, do not undergo thermal, (photo)chemical, hydrolytic, oxidative, or biological degradation, are not subject to long‐range transport, and have limited bioavailability (Henry et al. 2018).

The environmental fate and associated abiotic and biotic degradation of PFAS contained in fluorotelomer‐based AFFFs, surfactants, and side‐chain polymers are complex. As an example, the fluorotelomer methacrylates and acrylates that form the side chains in fluorinated side‐chain polymers can be degraded to various per‐ and polyfluorinated substances including fluorotelomer alcohols ((n:2) FTOHs). In turn, the (n:2) FTOHs may degrade to a variety of fluorotelomer saturated ((n:2) FTCAs) and unsaturated carboxylic acids ((n:2) FTUCAs; e.g., 6:2 FTCA, 6:2 FTUCA) and then finally to terminal degradation products such as PFCAs (PFHxA).

Some PFAS have also been introduced into multiple environmental matrices via the use of AFFFs to suppress hydrocarbon fires around the globe on ships and at military bases and airports, commercial airports, petrochemical storage facilities, and manufacturing plant sites. The environmental transport mechanisms just discussed for polymer processing aids are also relevant for AFFFs because their use can lead to PFAS water transport, long‐range atmospheric transport, deposition to soil, and contamination of groundwater/drinking water. Long‐range transport in both air and water (oceanic circulation) is frequently cited as the cause for detection of PFAS in remote environments such as the Arctic and Antarctic. Although either (or both) of these phenomena may play a role in the reported polar distribution of PFAS, the potential for local sources of these compounds also needs to be considered. The presence of military bases and associated activities in the Arctic and near‐Arctic areas around the globe provide the potential for localized inputs via fire‐fighting activities using AFFFs as well as other human‐associated activities (e.g., waste disposal; Collins 1998). In addition, these same regions contain nonmilitary airports of various sizes as well as a limited number of population centers with wastewater treatment plants that may not efficiently remove PFAS. Several studies have discussed some of the potential implications of these installations relative to other contaminants, as well as PFAS (Poland et al. 2001; Stow et al. 2005; Gewurtz et al. 2020).

Two additional historical sources of PFCAs and their precursors to the Arctic are shipping (naval and commercial) and activities associated with the petroleum industry. Specifically, AFFF has been and in some instances continues to be used for shipboard fire‐fighting for naval and commercial shipping of many countries, as well as in petroleum industry fire‐fighting activities at oil platforms (e.g., in the North Sea), refineries, and pipelines. Exhaustive data concerning the current use and storage of PFAS by military organizations around the world are not readily available. Furthermore, training and actual fire‐fighting activities have resulted in direct releases of unknown amounts of AFFF to the oceans. There are also well‐documented incidents of fire‐associated naval accidents and shipwrecks in the Arctic (e.g., see Bellona 2019).

### Bioaccumulation and bioactivity estimates

Bioaccumulation and biological effects of PFAS, reviewed in the later sections *Current Knowledge about Ecological Effects of PFAS* and *Current Knowledge about Ecological Exposures to PFAS*, are related to the physicochemical properties of the substances, their concentrations in the environment, and the physiology of the relevant organism(s). The large number of PFAS that could require potential evaluation suggests that new approaches are needed to facilitate rapid biological evaluations to support prioritization. The in vitro HTT and “omic” tools discussed in the *New Approach Method Applications in PFAS Ecological Risk Assessment* section are examples of rapid, cost‐effective techniques that could help prioritize PFAS for testing and monitoring based on bioactivity. Other relevant approaches that may be useful include in silico methods such as machine learning and computational models for predicting potential bioactivity (Cheng and Ng 2019) and methods to more quickly determine (or accurately estimate) elimination half‐lives in organisms, because elimination half‐life is related to both bioaccumulation and effects. For example, Goss et al. (2018) proposed that elimination half‐life could be used as a surrogate for actual bioaccumulation study endpoints. Differences in parameters such as organism half‐life can be utilized to help prioritize additional in vivo testing in terms of both organism selection and test design. A clear demonstration of the importance of test species and half‐life is captured by differences in half‐life among rat, monkey, and human for 4‐ and 8‐carbon PFCAs and PFSAs. For example, the half‐lives for perfluorobutanoate (PFBA) in rat, monkey, and human were 0.3, 2.0, and 3 to 4 d, respectively, compared with the half‐lives for PFOA, which were 5, 21, and 1000 d, respectively. Similarly, for perfluorobutane sulfonate (PFBS) the half‐lives for rat, monkey, and human were 0.2, 4.0, and 26 d, respectively, compared with PFOS, with half‐lives of 25, 45, and 1500 d, respectively (Organisation for Economic Co‐operation and Development 2013).

A potentially important aspect of bioactivity estimation can be the relevance (and measurement) of exposure concentrations utilized for testing relative to observed concentrations in the environment or projected future concentrations in the environment. This topic has previously been addressed by Holden et al. (2016), who argue that for another set of highly varied substances (engineered nanomaterials), evaluations utilizing environmentally relevant exposure concentrations provide the most meaningful data for environmental risk assessments.

### Essentiality

Recently there has been discussion about adding another aspect to prioritization schemes applied to PFAS. The “essentiality” of a substance in its applied uses has been proposed as important to consider in prioritization (Cousins et al. 2019). In the Madrid Statement (Blum et al. 2015), it was maintained that the production and use of PFAS should be limited. However, it is impractical that a blanket limitation could be instituted to cover the manufacture and use of all PFAS. Thus, the concept of limiting the use of PFAS to only those demonstrated to be essential to the health, safety, and functioning of society has been proposed (Cousins et al. 2019), as was done through the Montreal Protocol, which phased out the use of chlorofluorocarbons (United Nations Environment Programme 1987). In any discussion of essentiality, it is important to consider the physicochemical properties and environmental profile of the individual PFAS. Sometimes a use is not necessarily essential, but the environmental profile of the substance indicates it is not a concern and there may be a potential societal benefit from its use.

## CURRENT KNOWLEDGE ABOUT ECOLOGICAL EXPOSURES TO PFAS

Many studies in the open literature have addressed different facets of environmental PFAS exposures, including numerous monitoring studies. Given the scope of the present review, we cannot provide a comprehensive accounting or synthesis of this information. Instead, we summarize what is broadly known (see Supplemental Data SI‐2 text and tables), with the goal of providing an appropriate backdrop for discussing different aspects of assessing the ecological risks of PFAS. In addition, a companion paper emanating from the same SETAC workshop as the present review provides more detailed discussions of the current status of knowledge of human and wildlife exposure to PFAS (De Silva et al. 2020, in this issue). A brief summary of the points salient to the characterization of ecological exposure to PFAS follows.

- A variety of PFAS are released into the environment through manufacturing, use, waste streams, or incidents (e.g., fires). Consequently, PFAS are ubiquitous in the environment (McCarthy et al. 2017), having been measured in multiple media worldwide. Older environmental monitoring studies have focused predominantly on PFOS and PFOA, whereas the focus of more recent research has been the measurement and detection of many additional PFAS, as both targeted and nontargeted analytical methods evolve (De Silva et al. 2020, in this issue).
- Different PFAS partition into and are enriched in abiotic environmental matrices based on chemical‐, media‐, and site‐specific attributes. For example, some PFAS are highly mobile in water, with most mass transported in the aqueous phase including leaching from soil to groundwater and surface water (Ahrens and Bundschuh 2014; Conder et al. 2019; Interstate Technology Regulatory Council 2020b). Others are insoluble in water, thus limiting any exposure via this route.
- Organisms residing in environments into which PFAS have been released contact media containing PFAS and may accumulate the compounds. Although quantitative measurements of the interaction of organisms with PFAS in the environment such as bioconcentration factors (BCFs) and bioaccumulation factors (BAFs) are well defined for substances like PFOS and PFOA, little is known for the vast majority of PFAS. Some, but certainly not all, PFAS that have been studied are considered bioaccumulative.
- Uptake of PFAS by exposed organisms and subsequent internal distribution is a function of chemical‐, species‐, and tissue‐specific attributes. For example, some PFAS display a preferential affinity for proteins (e.g., Ng and Hungerbühler 2013, 2014, 2015), which limits the application of models based solely on lipid partitioning to predict bioaccumulation and distribution. Similarly, some PFAS are efficiently metabolized by organisms whereas others are not.
- Empirical evidence for the bioaccumulation/biomagnification of some PFAS at higher trophic levels in food webs exists (see Supplemental Data SI‐2; Tomy et al. 2004, 2009; Kelly et al. 2009), although the mechanisms underlying the phenomenon are uncertain. Available data indicate that PFAS exhibit a higher degree of biomagnification in upper trophic air‐breathing wildlife, compared with aquatic organisms and food webs (Kelly et al. 2009). Information on key physicochemical properties, such as protein/water partitioning coefficients and membrane/water distribution coefficients, may be useful for evaluating bioaccumulation potential of PFAS.

## CURRENT KNOWLEDGE ABOUT ECOLOGICAL EFFECTS OF PFAS

In this section, we seek to synthesize what is currently known concerning the toxicity of PFAS in species relevant to ecological risk assessment. For several of the taxa included in this analysis (invertebrates, fish, amphibians), we employed the ECOTOX Knowledgebase, an open‐access resource that curates results from ecotoxicological studies and is maintained by the USEPA (US Environmental Protection Agency 2020b). The ECOTOX Knowledgebase employs a documented literature search and data extraction capturing chemical, species, endpoint, effect concentrations, and so on, and a presentation archetype that makes the system convenient for this type of comparative evaluation. The ECOTOX Knowledgebase is extensive, containing thousands of records; however, not every PFAS toxicity study is captured in ECOTOX. For example, coverage of the non‐peer–reviewed gray literature and some non‐English journals can be inconsistent. Also, although additional chemicals are continually being added to ECOTOX searches, there can be a lag time between publication of results with newer chemicals/products, and actual appearance of the data in the knowledgebase. However, the various summaries/analyses identified through ECOTOX searches are more than adequate to provide important insights as to the universe of PFAS that have been tested, and the species and endpoints that have been used for this testing. In terms of using the ECOTOX output provided in the Supplemental Data of the present review, the reader is encouraged to 1) be aware of the fact that ECOTOX is updated quarterly, so searches may rapidly become outdated, and 2) obtain the original cited studies for any key decision that might be made concerning regulatory activities or research planning.

A summary is presented below of available laboratory‐based toxicity data for aquatic and terrestrial invertebrates and different classes of vertebrates (fish, amphibians, reptiles, birds, and mammalian wildlife). Following that, an overview is provided of studies focused on the potential effects of PFAS on different taxa in the field.

### Aquatic and terrestrial invertebrates

Numerous studies providing overviews of the toxicity of certain PFAS to select aquatic invertebrates have been published over the past 15 yr (Beach et al. 2006; Giesy et al. 2010; Qi et al. 2011; Ahrens and Bundschuh 2014; McCarthy et al. 2017; Valsecchi et al. 2017; Lee et al. 2019; Liu et al. 2019). For the present review, comprehensive ECOTOX searches were conducted to generate data for an up‐to‐date summary, as well as information for terrestrial invertebrates, which previously has not been reviewed. The specific procedure used to extract the data from ECOTOX (e.g., search terms) and to prepare tables for aquatic and terrestrial invertebrates, as well as the actual data, are presented in the various tables in the Supplemental Data, SI‐3 files.

#### PFAS and species tested

The ECOTOX Knowledgebase includes ecotoxicological studies on invertebrates for 47 different PFAS that can be grouped into 6 broad categories: 19 fluorotelomer substances, 13 PFCAs, 5 PFSAs, 3 fluorocyclohexanes, 3 perfluoroalkane sulfonamides (FASAs), and 4 fluorinated plant protection products (FPPPs). Of the 4 FPPPs, only one has a structure that is consistent with PFCA and PFSA compounds, sulfluramid (N‐ethylperfluorooctane sulfonamide). Despite the comparatively large number of PFAS that have been tested, most of the data (95% of the total) are from 3 of the listed classes; PFCAs (30%), PFSAs (47%), and FPPPs (18%), and 2 substances, PFOA and PFOS, dominate, comprising 21 and 39% of the entire dataset (Supplemental Data, Table SI‐3.1). In aquatic organisms, only a few substances (PFOA, PFOS, flubendiamide, and hexaflumuron) have been tested in a wide number of taxa (Supplemental Data, Table SI‐3.2), with the 2 most frequently tested groups being cladocerans (*Daphnia* spp.) and midges (*Chironomus* spp.). Toxicity information for terrestrial invertebrates is relatively limited (Supplemental Data, Table SI‐3.3), with much of it coming from species commonly used for soil testing, such as earthworms (*Eisenia fetida*) and nematodes (*Caenorhabditis elegans*). For terrestrial insect taxa, only PFOS and the 4 FPPPs have been tested.

### Toxicity overview

#### Aquatic invertebrates

Effects of PFAS on apical endpoints in aquatic invertebrates tested in acute and chronic exposures are summarized in the Supplemental Data, Table SI‐3.4. Minimum acute toxicity values ranged from 0.0001 to 195 mg/L depending on PFAS class and species tested (Supplemental Data, Table SI‐3.5a). In general, crustaceans often were the most sensitive of the aquatic invertebrate taxa. Furthermore, toxicity in the same PFAS class tends to increase with increased fluorocarbon chain length. Based on the PFAS in the present analysis, the FPPPs, used as insecticides in some countries were the most toxic, with median lethal/effect concentration (LC50/EC50) values ranging from 0.0001 to 1.8 mg/L, followed by FASAs, (n:2)FTCAs, (n:2)FTUCAs, and PFSAs, with the PFCAs being least toxic. Toxicity data are also available for several replacement products, including GenX, a replacement for PFOA, where the 96‐h no‐observed‐effect concentration (NOEC) based on survival in *Daphnia magna* was 97 mg/L (Hoke et al. 2016).

Except for PFOS and FPPPs, EC50 and LC50 values for PFAS from chronic exposures generally ranged from 0.03 to more than 100 mg/L and mostly were in the same order of magnitude as values from acute exposures within the same species (Supplemental Data, Table SI‐3.4). For PFOS, interspecies difference in acute toxicity can range by approximately 2 to 3 orders of magnitude. One example of this difference is between the amphipod *Monoporeia affinis*, with a 3‐wk LC50 of 0.064 mg PFOS/L (Jacobson et al. 2010), and the daphnid *D. magna*, with 7‐ to 21‐d LC50 values ranging from 9 to 43 mg/L (Supplemental Data, Table SI‐3.4). However, factors other than species sensitivity may have contributed to this difference in toxicity because *D. magna* were exposed in a water‐only system and *M. affinis* were exposed in a water–sediment system. In the water–sediment system, a substantial flux of PFOS from the overlying water to the sediment can occur, with sediment potentially acting as a second route of exposure (Marziali et al. 2019). As a result, the effects levels based only on water concentration may have underestimated the total exposure of *M. affinis*, resulting in the LC50 being artificially low.

Growth, development, and reproduction have been assessed for fluorotelomers ((n:2)FTCAs, (n:2)FTUCAs), PFCAs, PFSAs, and FPPPs (Supplemental Data, Table SI‐3.5b). Of these compounds, PFOS has been the most studied and in the most taxa, including rotifers, molluscs, crustaceans, and insect larvae. To date, the chironomid *Chironomus riparius* appears to be one of the most sensitive species, with adverse reproductive effects occurring at 4 µg PFOS/L; however, variability associated with toxicity results from other species can be relatively large, making it difficult to evaluate interspecies sensitivity. For instance, based on reproductive effects in daphnids, NOEC and lowest‐observable effect (LOEC) concentrations can range from a few µg/L to 10 s of mg/L, a range that approaches what is observed in chironomids (Boudreau et al. 2003; Lu et al. 2015). Moreover, toxicity between PFAS groups within a species can also overlap, as is observed for other PFAS such as 10:2 FTCA, 10:2 FTUCA, and perfluoronoanoate (PFNA), where reproductive LOECs in daphnids can range from 33 to 200 µg/L (MacDonald 2006; Wang et al. 2014; Lu et al. 2015), values similar to those observed with PFOS. Lastly, PFOA did not impair reproduction below 6.25 mg/L in any species (Ji et al. 2008), and GenX did not affect reproduction in *D. magna* exposed to 102 mg/L (Hoke et al. 2016). In general, developmental effects of PFAS in invertebrates occur at lower concentrations than observed for growth and reproductive endpoints (Supplemental Data, Table SI‐3.5b). For instance, in the Mediterranean mussel (*Mytilus galloprovincialis*), a decrease in normal larval development was observed at 0.1 µg PFOS/L (Fabbri et al. 2014). Likewise, adverse effects on time to first emergence, rate of emergence, and total emergence have been noted in chironomids with LOECs that ranged from 2.0 µg PFOS/L for *Chironomus tentans* to 10 µg PFOA/L for *C. riparius* (MacDonald et al. 2004; MacDonald 2006; Stefani et al. 2014; Marziali et al. 2019).

Acute and chronic toxicity studies have been conducted with only a few PFAS in saltwater species (Supplemental Data, Table SI‐3.4). For flubendiamide, the 96‐h NOEC for *Crassostrea virginica* was 0.049 mg/L (US Environmental Protection Agency 1992), and for hexaflumuron, the 14‐d LC50 in the marine shrimp (*Penaeus aztecus*) was 0.0001 mg/L (Dow Chemical 1990). Overall, when compared with freshwater species, marine invertebrates tend to show a higher sensitivity to PFOA and PFOS, but given the limited nature of the available studies, the true magnitude of this difference is uncertain (Supplemental Data, Table SI‐3.5).

#### Terrestrial invertebrates

Few toxicological studies with PFAS have been conducted with terrestrial invertebrates (Supplemental Data, Table SI‐3.6). Moreover, although many of these researchers have studied FPPPs, there are only limited data associated with other classes of PFAS. In honeybees (*Apis mellifera*), oral exposure to PFOS resulted in a 72‐h oral median lethal dose (LD50) of 0.40 mg/bee, a value of concern in terms of pesticide toxicity to bees (Wilkins 2001). In *E. fetida*, 14‐d LC50 values for PFOS and PFOA were 373 mg/kg and 760 mg/kg soil dry weight, respectively (Sindermann et al. 2002), and for FPPPs, 14‐d LC50 values ranged from 374 to 400 mg/kg dry weight (Wang et al. 2012a, 2012b).

In studies with 2 earthworm species (*E. fetida* and *Aporrectodea caliginosa*), 3‐ to 4‐wk NOEC and LOEC values were 1 and 100 mg/kg soil (dry wt) for PFHxS, PFOS, perfluoroheptanoic acid (PFHpA), PFNA, and PFOA (Xu et al. 2013; Zareitalabad et al. 2013; Karnjanapiboonwong et al. 2018). Exposure of springtails (*Folsomia candida*) to PFOS resulted in a 28‐d 10% inhibitory concentration (IC10) of 57 mg/kg dry soil (Princz et al. 2018). Developmental toxicity was assessed for several fluorotelomers, PFCAs, and PFSAs utilizing a high‐throughput system with *C. elegans* (Supplemental Data, Table SI‐3.6). Toxicity (48‐h half‐maximal activity concentration) ranged from 4.1 µM for henicosafluoroundecanoic acid (2.3 mg/L in culture) to 408 µM for 8:2 FTOH (189 mg/L in culture); for 6:2 FTOH, PFHpA, and PFHxS, toxicity values were 1000 µM (364–400 mg/L in culture) for all 3 chemicals (Boyd et al. 2016). In general, developmental toxicity increased with increasing length of the fluorinated alkyl chain within a PFAS group; trends in toxicity between different PFAS groups was as follows: FASAs > PFSAs > PFCAs > (n:2) FTOH. Overall, based on the limited amount of data currently available for terrestrial invertebrates, it is not yet possible to evaluate the potential toxicological differences related to different PFAS groups, much less understand interspecies differences in individual PFAS. Given the importance of these taxa to soil processes and other ecological functions, this is a large data gap that needs to be addressed.

#### Multigenerational invertebrate studies

The multigenerational effects of PFAS have been studied in select species including *C. riparius* (Marziali et al. 2019) and *D. magna* (Jeong et al. 2016). In a 10‐generation study with *C. riparius*, exposure to PFBS, PFOA, and PFOS (8, 9, and 4 µg/L respectively) resulted in significant effects on growth, reproduction, and development across several generations compared with controls (Marziali et al. 2019). In the same study, pre‐exposure to PFOS or PFOA did not induce tolerance but rather seemed to enhance susceptibility, with the magnitude of effect being greater for endpoints such as early emergence and adult weight when organisms were re‐exposed to PFBS, PFOA, and PFOS. Exposure regime can also influence toxicity, as observed in a continuous exposure of daphnids to PFOS across multiple generations. In that study, adverse reproductive effects decreased, with the LOEC increasing from 0.1 to 1 mg/L in later generations, whereas an individual fitness endpoint (i.e., acetylcholinesterase activity) LOECs decreased from more than 10 to 0.1 mg/l in later generations (Jeong et al. 2016). In a discontinuous exposure (F0 exposed, F1 not exposed, F2 exposed), toxic effects were noted for all endpoints in F0 but lessened in F1. However, adverse effects were enhanced when F2 was re‐exposed to PFOS beyond that observed in the F2 generation from the continuous exposure. This effect was similar to that observed with the terrestrial insect *Drosophila hydei* exposed to PFOS (Van Gossum et al. 2010). The results from these 2 studies emphasize the need for additional research in understanding the impact of diverse, multigenerational exposure scenarios that may more accurately represent exposure in natural systems, as well as the need to better understand the linkage between individual fitness endpoints and population dynamics in aquatic and terrestrial invertebrate communities.

#### Mechanism‐based invertebrate toxicity studies

Relationships between PFAS exposure and effects at the molecular, cellular, physiological, and morphological levels have been evaluated in several invertebrate species. The PFAS have been shown to cause signs of oxidative stress and to affect the antioxidant defense systems of aquatic and terrestrial invertebrates, including changes in lipid peroxidation (LPO), increases in reactive oxygen species (ROS), and changes in antioxidant enzyme activities and expression (Supplemental Data, Table SI‐3.7a). Exposure to PFAS has also been associated with genotoxic effects including DNA strand breaks and fragmentation, chromosomal breaks, and apoptosis in several invertebrate species (Supplemental Data, Table SI‐3.7b).

Neurotoxic effects have also been observed in several invertebrate species including the planarian *Dugesia japonica*, exposed to PFOA or PFOS (Supplemental Data, Table SI‐3.7c). In this species, exposure resulted in changes such as altered brain morphology and a significant reduction in locomotor velocity (Yuan et al. 2014, 2015, 2016, 2018). Behavioral abnormalities related to feeding and filtration rates, swimming, valve closure, burrowing, and locomotor endpoints such as number and direction of movements have been observed in other aquatic and terrestrial invertebrates exposed to PFAS (Supplemental Data, Tables SI‐3.4 and SI‐3.6). Metabolic effects of PFOS have also been examined in *D. magna*, and included a decrease in amino acids in adults (Kovacevic et al. 2019) and changes in metabolic profiles between age groups, with neonates prioritizing growth and adults prioritizing reproduction compared with controls (Wagner et al. 2017; Supplemental Data, Table SI‐3.7d). In a multigenerational study, Li et al. (2020) conducted an exposure with wild‐type N2 *C. elegans* and the daf‐2 mutant for 4 consecutive generations (F0–F3) to PFOA. They found obesogenic effects (fat accumulation due to increased fatty acid synthesis) in exposed generations and the unexposed offspring due to alterations of a complex combination of various enzymes and pathways including peroxisome proliferator‐activated receptor (PPAR), mitogen‐activated protein kinase, and insulin signaling pathways.

Invertebrates do not have an adaptive immune system; instead they mostly rely on innate immune responses that can be associated with hemocytes or coelomocytes. These cells play a crucial role in nonspecific cellular immunity and, along with immune‐related enzymes and other cellular components, form the innate immune system. Several PFSAs and PFCAs have been shown to adversely affect immune‐related cell viability in aquatic crustaceans, bivalves, and terrestrial earthworms (Supplemental Data, Table SI‐3.7e). Mayilswami et al. (2016) evaluated the gene expression profile changes in *E. fetida* chronically exposed to PFOA‐amended soil (10 mg/kg dry wt) and observed alterations in genes involved in lipid metabolism, neuronal development, calcium homeostasis, apoptotic process, and reproduction. In *Mytilus californianus*, significant inhibition of p‐glycoprotein transporter activity was observed after exposure to PFOA, PFNA, PFDA, and PFHxS, but no significant effects were noted in mussels treated with PFBS, PFOS, PFDS, perfluoroundecanoic acid (PFUnDA), and short‐chain PFCAs (Stevenson et al. 2006). Interestingly, inhibition of the p‐glycoprotein transporter did not appear to be related to differences in bioaccumulation because PFOS concentrations in mussel gill tissue were greater than concentrations of either PFHxS or PFOA, but the effect on the transporter was less, illustrating the utility of having tissue residue data for comparative interpretation of biological effects.

The endocrine‐disrupting potential of PFAS has been studied in 2 invertebrate species, *D. magna* and *C. elegans* (Supplemental Data, Table SI‐3.7f). In *D. magna* chronically exposed to 0.06 mg/L perfluoroethylcyclohexane sulfonate, vitellogenin (VTG; egg yolk protein) content was decreased, and exposure to 0.6 mg/L reduced the expression of VTG‐related genes relative to controls; however, no effects on survival, molting, or reproduction were observed in any of the treatment groups (Houde et al. 2016). The expression of fecundity‐related genes was assessed in *C. elegans* treated with PFBS and PFOS. Interestingly, *vit*‐6 (encoding vitellogenin) was down‐regulated, whereas expression of *nhr*‐14 (encoding the estrogen receptor) was up‐regulated by both compounds, potentially reducing VTG levels (Chen et al. 2018a).

### Fish

#### PFAS and species tested

The ECOTOX Knowledgebase reports studies with fish for 29 different PFAS belonging to 6 categories: 9 fluorotelomers, 11 PFCAs, 5 PFSAs, 2 FASAs, 1 FPPP, and 1 polyfluorinated ether sulfonate. As with the invertebrate literature, the great majority of the data (more than 90%) has focused on PFCAs (47%) and PFSAs (44%), with the remaining 9% comprised mostly of fluorotelomers (4.7%; Supplemental Data, Tables SI‐3.8 and SI‐3.9). Toxicity data for PFOA and PFOS make up 62% of all the data available for fish, and there are more than twice the entries for PFOS compared with PFOA.

Supplemental Data, Tables SI‐3.10 and SI‐3.11 summarize PFAS toxicity data based on fish family. Most of the data available are for freshwater Cyprinidae (59%), followed by Salmonidae (18%), Adrianichthyidae (represented only by medaka species; 12%), and other families (11%). Within the Cyprinids, 90% of the data comes from zebrafish, (*Danio rerio*), with 47% of all records. Other Cyprinids in the dataset include fathead minnow (*Pimephales promelas*), common carp (*Cyprinus carpio*), creek chub (*Semotilus atromaculatus*), Chinese rare minnows (*Gobiocypris rarus*), and goldfish (*Carassius auratus*). Atlantic salmon (*Salmo salar*) and rainbow trout (*Oncorhynchus mykiss*) are the only salmonids in the ECOTOX Knowledgebase with PFAS toxicity data.

Marine fishes have been much less well studied for PFAS toxicity than freshwater species and accounted for less than 5% of the total entries. Marine species for which there are data include marine medaka (*Oryzias melastigma*), mullet (*Mugil* spp.), flounder (multiple genera), rockfish (*Sebastes schelgelii*), sea bream (*Sparus aurata*), cod (*Gadus morhua*), and sheepshead minnows (*Cyprinodon variegatus*; Supplemental Data, Table SI‐3.11).

#### Toxicity overview

There is a comparatively large amount of acute toxicity data for PFAS in fish. Overall, PFAS have relatively low acute toxicity compared with invertebrate taxa. In Cyprinids, LC50 values for PFAAs are in the hundreds to thousands of mg/L for C4 compounds (PFBS and PFBA) compared with PFAAs with chain lengths of 6 or more (i.e., C6, C8, C9, and C10) that are in the 10 s and single‐digit mg/L range. Within the same chain length (C8), sulfonates are typically more toxic than carboxylates, with upper‐end 96‐h LC50 values in Cyprinids, Salmonids, and other families for PFOA being 759, 4000, and 634 mg/L, respectively, compared with 82, 4.2, and 49 mg/L for PFOS, respectively. This relationship holds true for most of the fish families studied. For fluorotelomers, 96‐h LC50 values ranged from 34 to more than 300 mg/L for 6:2 FTOH and from 7 to 25 mg/L for 6:2 FTCA. In a limit test with rainbow trout exposed to GenX, the 96‐h NOEC based on survival was 97 mg/L (Hoke et al. 2016).

Several chronic fish toxicity tests have been conducted with PFAS. The ECOTOX Knowledgebase summarizes a variety of endpoints for these tests (e.g., survival, growth, reproduction, changes in enzymes, hormones, genes, etc.). In the present summary/analysis, we focus on the types of apical endpoints typically considered to be predictive of ecologically relevant responses. Reported NOECs, LOECs, and point estimates of effect concentrations (EC*x*) for mortality, growth, development, and reproduction are summarized in the Supplemental Data, Tables SI‐3.10 and SI‐3.11. In Cyprinids, a similar trend to that observed in acute toxicity is seen in chronic toxicity relative to fluorinated chain length and functional groups (Supplemental Data, Table SI‐3.10). For example, no effects on growth were detectable after exposure to 137 mg PFBA/L PFBA, and for PFOA, PFOS, PFNA, and perfluorotridecanoic acid (PFTrDA), LOECs ranged from 0.0007 to 5.6 mg/L. Effects of 2 fluorotelomers on growth have also been studied, and NOEC or LOEC values were found to range from 0.05 mg/L for 6:2 FTOH to 7.9 mg/L for 10:2 FTCA. Interestingly, in a study with zebrafish embryos exposed to F‐53B (a mixture comprised of ~80% chlorinated‐polyfluoroalkylether substances [Cl‐PFAES]), the 48‐h postfertilization LOEC was 1.5 mg/L. Although data on growth effects are sparse in fish taxa other than Cyprinidae, toxicity values followed a similar trend (Supplemental Data, Table SI‐3.11). For families of fish for which data are available, NOEC or LOEC values for PFOA and PFOS ranged from 0.1 to 40 mg/L and 0.01 to 3 mg/L, respectively. In an early life stage study with rainbow trout exposed to GenX, the 91‐d NOEC based on the percentage of hatch was 8.89 mg/L, a value that falls within the range of PFOA toxicity values (Hoke et al. 2016).

There are a few fish reproduction studies with PFAS. In Cyprinids, NOEC or LOEC reproductive values were lower than those for growth (0.0034 and 0.2 mg/L for PFOA and PFOS, respectively). These values tended to be higher for other fish taxa, including medaka (10 and 1 mg/L for PFOA and PFOS, respectively), but the overall dataset is small. Reproductive studies with (n:2) FTOHs in Cyprinids produced LOEC values of 0.03 to 0.27 mg/L.

#### Multigenerational fish studies

Multigenerational studies have been conducted in fish exposed to several PFAS. A 2‐generation study with medaka found that offspring (F1) from fish exposed to 0.01 mg PFOS/L and 0.1 mg PFOA/L had decreased survival (Ji et al. 2008). Adverse effects were more pronounced in fish continuously exposed from F0 to F1 generations. In another study, 30 mg PFOA/L decreased fecundity in medaka exposed to PFOA for 3 generations (F0–F2); however, fecundity was not altered in fish exposed to 3 mg/L (Lee et al. 2017a). Wang et al. (2011) exposed zebrafish to 0.25 mg PFOS/L for 5 mo and reported that the PFOS body burden in F1 larvae derived from maternal transfer correlated negatively with survival and changes in swimming behavior. Another multigenerational study with zebrafish evaluated the toxicity of PFNA and reported thyroid‐mediated responses in F1 larvae exposed to 0.05 mg/L (Liu et al. 2011). A multigenerational PFAS mixture study (exposure to equal concentrations of PFNA, PFOS, PFOA, and PFBS in water) with medaka found decreased hatching success and gonad size in the F1 fish and estrogenic effects (induction of VTG) in the F2 generation (Lee et al. 2017b). In another multigenerational study with zebrafish, exposure to 0.6 µg PFOS/L resulted in adverse effects on growth, but no significant effects on growth were noted in fish exposed to higher concentrations. As a result, the toxicological significance of this result is uncertain given the lack of dose–response relationship (Keiter et al. 2012).

#### Mechanism‐based fish toxicity studies

Understanding the biochemical and physiological basis of the toxicity of different PFAS provides the critical conceptual underpinnings needed for extrapolation of effects from tested to untested chemicals and/or species. In this section, we briefly describe what is known concerning different aspects of PFAS toxicity in fish from a mechanistic perspective. Readers seeking more information on this topic should consult Lee et al. (2019), who recently published a review concerning toxicity mechanisms/pathways for PFAS in fish.

Different PFAS have been shown to elicit oxidative stress and apoptosis in fish both in vitro and in vivo. The hallmark cellular changes associated with oxidative stress (such as increased ROS, decreased glutathione levels, decreased superoxide dismutase and catalase levels, and increased LPO) have been reported in several studies (Lee et al. 2019). The actual mechanism(s) through which PFAS induce oxidative stress are not well understood, but increased β‐oxidation of fatty acids and mitochondrial toxicity have been proposed as triggers.

Exposure of fish to several PFAS has been associated with disruption of the hypothalamus–pituitary–gonadal and –thyroidal (HPG and HPT, respectively) axes, which can affect aspects of adult reproduction and larval sexual development (e.g., Ankley et al. 2005; Jo et al. 2014; Zhang et al. 2016). Some PFAS (e.g., PFOA, PFNA, PFDA, and PFUnDA) have been reported to be estrogenic in vivo, inducing VTG in juvenile rainbow trout, with PFOA and PFOS reported to bind in vitro to the trout estrogen receptor (Benninghoff et al. 2011). Changes in the gonadal expression of several genes involved in steroid synthesis and HPG axis regulation have been reported in female zebrafish exposed to 0.01 mg PFNA/L (Zhang et al. 2016) and 0.01 mg PFTrDA/L (Jo et al. 2014), corresponding to decreased egg production and hatching success. Both PFCAs also increased plasma 17β‐estradiol levels in males, which may be linked to reductions in male gonadosomatic index. In male fathead minnows, 0.3 mg/L PFOS caused an increased concentration of androgens and decreased testicular aromatase activity (Ankley et al. 2005).

Exposure of fish to some PFAS has also been associated with perturbation of the HPT axis. Zebrafish embryos exposed to perfluorododecanoic acid (PFDoA; 1.2 mg/L) experienced growth reduction accompanied by dysregulation in the expression of several genes associated with thyroid function (Zhang et al. 2018). A similar study with 0.5 mg PFOS/L caused decreased growth, decreased thyroid hormone levels, and down‐regulation of HPT axis genes (Kim et al. 2011). Long‐term exposure of zebrafish (embryo to reproductive adults) to 0.25 mg PFOS/L decreased whole‐body thyroid hormone levels and caused a generalized down‐regulation of thyroid‐related genes, as well as cellular changes in the thyroid follicles (Chen et al. 2018b). A similar exposure with zebrafish exposed to 0.05 mg PFNA/L resulted in elevated plasma T3 levels and thyroid gland cellular changes, along with changes in the expression of thyroid‐related genes (Liu et al. 2011).

As observed in mammalian species, several PFAS including PFOA and PFOS have been shown to activate nuclear receptors involved in lipid metabolism, such as PPARs in fish. (e.g., Arukwe and Mortensen 2011; Yang et al. 2014). Activation of PPARs by PFOS in mammals results in increased β‐oxidation of fatty acids, which increases liver lipid content and results in severe steatosis (fatty liver), and a coincident decrease in serum levels of apolipoproteins (Cheng et al. 2016). In zebrafish, hepatic effects were reported in animals exposed to 0.25 mg PFOS/L, which included severe fatty liver degeneration in F0 males and a dysregulation in the expression of genes associated with lipid metabolism in F1 larvae (Cheng et al. 2016). These results highlight the utility of linking molecular changes to apical effects to understand the possible consequences of perturbation of specific biological pathways.

### Amphibians

#### PFAS and species tested

Only a limited number of toxicity studies on PFAS in amphibians have been published (Supplemental Data, Tables SI‐3.12 and SI‐3.13). As seen in the ECOTOX Knowledgebase, a total of 8 PFAS belonging to 3 categories have been tested in amphibians: 4 PFCAs, 3 PFSAs, and 3 fluorotelomers. As with other taxonomic groups, the great majority of the data are for PFOS and PFOA.

Four species of amphibians have been studied in toxicity tests. Two are native to Africa, the African clawed frog (*Xenopus laevis)* and the western clawed frog (*X*. *tropicalis*); one is from Asia, the Asiatic toad (*Bufo gargarizans*); and one is native to North America, the northern leopard frog (*Rana pipiens*). A recently published study examining PFOS and PFOA mixture effects in American bullfrogs (*Rana catesbeiana*; Flynn et al. 2019) was not in ECOTOX at the time data for the present review were assembled. Two additional amphibian species, the eastern tiger salamander (*Ambystoma tigrinum*) and the American toad (*Anaxyrus americanus*), have been used in laboratory‐based bioaccumulation studies with PFOS and PFOA in which no adverse biological effects were observed under the exposure conditions employed (Abercrombie et al. 2019).

#### Toxicity overview

As in fish, PFAS are not highly toxic to amphibians in short‐term exposures, but their observed potency is influenced by fluorinated chain length and functional group. For example, acute 96‐h LC50 values in amphibian tadpoles across species for PFOA, PFOS, PFDA, and PFUnDA averaged 377, 48, 76, and 68 mg/L, respectively. A single study reported a comparatively higher 96‐h LC50 value (332 mg/L) for PFNA in *X. laevis*. In American bullfrogs, 96‐h LC50 values for PFOS were an order of magnitude lower than for PFOA, and effects of the 2 chemicals in combination appeared to be additive (Flynn et al. 2019).

Effects of PFAS on growth and development of early amphibian life stages have been observed in several species. In a study with *R. pipiens* tadpoles, exposure to 0.01 mg PFHxS/L and 0.1 mg PFOS/L resulted in 40‐d LOEC values based on delayed development (Hoover et al. 2017). The effects of PFOS on metamorphosis have varied, with 8‐d LOEC values ranging from 3.6 to 48 mg/L. The NOEC value for 6:2 fluorotelomer sulfonate was 1.0 mg/L. In another study with *R. pipiens* tadpoles, exposure to PFOA and PFOS for 30 d resulted in decreased brain dopamine levels but no effects on serotonin, norepinephrine, γ‐amino butyric acid, glutamate, or acetylcholine (Foguth et al. 2019). An experiment using seminatural exposure conditions (mesocosms) reported a PFOS 30‐d LOEC of 0.06 µg/L based on developmental delays, much lower than that reported for the same species under controlled conditions (10 µg/L; Flynn et al. 2020). A 72‐d exposure of American bullfrogs to PFOS, PFOA, and a mixture of the 2 chemicals showed effects that varied markedly by endpoint. Based on tadpole mass and development stage, toxicity was driven by PFOS, and effects appeared to be additive. However, based on snout–vent length, only the PFOA exposure caused a reduction in this endpoint whereas the toxicity observed in the PFOA–PFOS mixture was nonadditive and greater than expected. These results demonstrate the need to better understand how mixtures behave toxicologically (Flynn et al. 2019).

### Birds

#### PFAS and species tested

Laboratory studies have been conducted with a small number of bird species, employing endpoints suitable for risk assessment (survival/mortality, growth, reproduction, and developmental parameters) with accepted protocols established by regulatory agencies (Supplemental Data, Table SI‐3.14). To date, acute and chronic toxicity studies with PFAS have been conducted with chickens (*Gallus* spp.), northern bobwhite (*Colinus virginianus*), Japanese quail (*Coturnix japonica*), and mallards (*Anas platyrhynchos*). Specific PFAS that have been tested in birds include PFOS, PFBS, PFHxS, and PFOA, as well as 2 AFFF formulations, one a product with more than 90% PFOS as the fluorochemical content, and the other comprised primarily of 6:2 fluorotelomer thioamido sulfonate. Although other avian studies have been conducted with PFAS, the focus of most of this research has been on investigating mechanistic aspects of biological effects, employing endpoints related to gene expression, sex hormones, thyroid hormones, and lipid homeostasis (e.g., O'Brien et al. 2011, 2013). Several other published studies have examined the bioaccumulation and tissue distribution of PFAS in avian species in the laboratory (Gebbink and Letcher 2012; Tarazona et al. 2015).

#### Toxicity overview

Acute dietary studies have shown that the toxicity of PFAS is influenced by the carbon chain length and the functional head group (Newsted et al. 2006, 2008; Bursian et al. 2019). Although data are limited, the ranking of toxic potency of PFAS in birds agrees with rodent studies in which sulfonates were found to be more toxic than carboxylates for similar fluorocarbon‐chain lengths, and 8‐carbon chain length compounds were more toxic than shorter chain PFAS (Supplemental Data, Table SI‐3.15). The toxicity of PFAS (greatest to least) based on 8‐d LC50 values from studies with northern bobwhite and Japanese quail was PFOS > PFOA ≫ PFBS ~ a 6:2 fluorotelomer thioamido sulfonate AFFF. The acute toxicity of PFBS and the AFFF was undetermined because no birds died in either study at concentrations exceeding 1000 mg/kg feed. Potential differences in species sensitivity are difficult to ascertain given the limited available acute toxicity data. However, some insight can be gained by examining the acute toxicity of PFOS, with the greatest variation being observed between mallards (8‐d LC50: 603 mg/kg feed) and northern bobwhites (8‐d LC50: 212 mg/kg feed), a less than 5‐fold difference. The ecological relevance of this difference when other avian wildlife and PFAS are considered is uncertain given that that analysis only considered PFOS and included only 3 species, 2 of which are closely related phylogenetically. Additional studies with a broader suite of both PFAS and avian species are needed to better understand the range of sensitivities that may be representative of other groups such as colonial water birds and upper trophic predators. These types of species are commonly used in various monitoring programs and have been the focus of risk assessments for other types of chemicals like polychlorinated biphenyls (PCBs).

To date, chronic reproduction studies have been conducted with 3 avian species, mallards, northern bobwhite, and Japanese quail with PFOS, PFBS, PFHxS, and a PFOS‐based AFFF (Newsted et al. 2006, 2007; Bursian et al. 2019; Supplemental Data, Table SI‐3.16). In mallards and northern bobwhites exposed to 50 and 100 mg PFOS/kg, overt toxicity (lethality) was observed within 5 wk from the onset of exposure whereas no effects on survival and/or morbidity were observed in a 10‐mg PFOS/kg treatment (Newsted et al. 2007). In the 10‐mg PFOS/kg treatments, no significant effects were noted in mallard adults and their offspring exposed to 1.5 mg PFOS/kg body weight/d whereas in northern bobwhites, significant effects on adult female body weight and reproduction were associated with doses of 0.77 mg/kg body weight/d. Adverse reproductive effects in Japanese quail were observed in adults exposed to 2.1 mg PFOS/kg feed (~0.28 mg/kg body wt/d; Bursian and Link 2018). In that study, effects on adult female and 7‐d‐old chick body weights were observed in birds treated with 4.0 mg PFOS/kg (~0.55 mg/kg body wt/d) whereas no effects were noted in the 2.1‐mg PFOS/kg feed treatment group. In a study with Japanese quail exposed to a PFAS‐based AFFF formulation, no‐observed‐adverse‐effect level (NOAEL) and lowest‐observed‐adverse‐effect level (LOAEL) values based on 7‐d chick survival were 5.0 mg PFOS/kg (~0.66 mg/kg body wt/d) and 11 mg PFOS/kg (1.4 mg/kg body wt/d), respectively. These values are within a factor of 2 of the PFOS LOEC determined with northern bobwhite quail, suggesting that at least for these 2 closely related species, the effects of PFAS on chronic apical endpoints are relatively similar. In a dietary study with northern bobwhites exposed to PFBS for 21 wk (Newsted et al. 2008), there were no significant effects on survival, growth, or reproductive success in adults exposed to 900 mg/kg in the feed (88 mg/kg body wt/d). In northern bobwhites exposed for 90 d to PFOS or a 1.2:1 ratio mixtures of PFOS:PFHxS via drinking water (Dennis et al. 2020), the PFOS:PFHxS mixture reduced weight gain in females at 3.1 × 10^−3^ mg/kg body weight/d, whereas exposure to PFOS resulted in adverse effects on liberation from the shell post pipping at 2.45 × 10^−3^ mg/kg body wt/d. The results of PFOS alone and the PFOS:PFHxS mixture were similar, suggesting additivity. Variability associated with measured endpoints and the lack of measured tissue concentrations make it difficult to compare these results with those observed in the dietary studies. Overall, the selection and application of these data in the development of toxicity reference values (TRVs) should be conducted carefully.

Egg‐injection studies have also been conducted with PFAS, which have included mechanistic as well as apical embryonic endpoints in several avian species (Supplemental Data, Table SI‐3.17; Pinkas et al. 2010; Nordén et al. 2016). In these studies, adverse effects on embryonic survival and hatchability have been observed at PFOS egg injections ranging from 0.1 up to 100 µg/g egg, with no discernible difference in cross‐species sensitivity. This concentration range encompasses a lowest‐observed‐effect level based on reproductive effects in bobwhite quail of 62 µg PFOS/mL of egg yolk (Newsted et al. 2006). The lack of significant differences between species may be due to differences in experimental design, injection methodologies, and endpoint selection/definition, as well as the variability observed in these studies. Somewhat analogously, studies with PFOA, PFHxA, PFHxS, and F‐53B in chicken embryos did not demonstrate expected structure–toxicity relationships based on pipping success, developmental endpoints, and thyroid hormone levels (Cassone et al. 2012a; Nordén et al. 2016; Briels et al. 2018). Thus, although the egg injection model can provide insights into the mechanistic aspects of PFAS effects on reproductive and developmental processes (Stromqvist et al. 2012; Jiang et al. 2013; Mattsson et al. 2015; Jacobsen et al. 2018; Geng et al. 2019), additional in vivo studies are needed to better understand their relationship to other endpoints including potential effects on the adult birds, such as impacts on egg quality, male fertility, and parental behavior during and after hatch (Heinz et al. 2006).

### Reptiles

To date, few toxicity studies with PFAS have been conducted with reptiles. In a laboratory study with PFOS, effects on immune and clinical chemistry endpoints in blood were noted in western fence lizards (*Sceloporus occidentalis*; DeWitt et al. 2012). In brown anoles (*Anolis segrei*), exposure to 3 mg/kg body weight/d for 35 d resulted in decreased growth of juveniles, and exposure to PFHxS resulted in decreased egg viability in female anoles (Furst et al. 2019). Field studies with several reptilian species have included potential biomarkers of PFAS exposure and effects (Keller et al. 2012; Guerranti et al. 2014; Bangma et al. 2019). However, although some of these studies have found correlations between PFAS exposure and changes in biomarker responses, linkages to apical endpoints relevant to ecological risk assessments are uncertain. Thus additional studies are needed to better understand the toxicity of PFAS to reptiles.

### Mammalian wildlife

Although laboratory PFAS toxicity studies with mammalian wildlife have not yet been published, numerous studies with rodents, rabbits, and monkeys have been conducted that include apical endpoints and have been used to derive TRVs and support ecological risk assessments. One laboratory study is underway at the US Army Public Health Center (Gunpowder, MD) with a wild species of mouse (*Peromyscus* spp.) and PFOS, PFOA, PFBS, and PFHxS. Additional studies in mammalian laboratory species have examined the effects of PFAS on immunological, neurological, and histopathological endpoints. Although these endpoints are not typically used in ecological risk assessments, immunological and neurological endpoints have been employed to assess potential effects of PFAS in several wildlife groups including polar bears (Bourgeon et al. 2017; Dietz et al. 2018), other marine mammals (Fair and Houde 2018; Grønnestad et al. 2018), and sled dogs (Sonne 2010). Overall, published studies with results that support the derivation of TRVs have been reviewed and summarized in Conder et al. (2019) and include approximately 9 PFCAs, 3 PFSAs, 3 fluorotelomer substances, and 3 “replacement” compounds including GenX and ADONA (ammonium 4,8‐dioxa‐3H‐perfluorononanoate). Of the PFAS studied to date, PFOS followed by PFOA has the greatest amount of toxicological data relevant to the development of TRVs for mammalian wildlife.

Based on rodent data, no‐observed‐effect levels for ecologically relevant endpoints in mammalian wildlife ranged from 30 mg/kg body weight/d for PFBS and PFHxA to 0.1 mg/kg body weight/d for PFOS (Supplemental Data, Table SI‐3.18). The least toxicity was associated with PFCAs with fluorocarbon chains of C6 or less whereas the greatest toxicity was observed for C8 to C12 compounds. Interestingly, perfluorotetradecanoic acid (C14) was less toxic than C8 to C12 PFCAs. For PFSAs, fluorocarbon chain length also appeared to influence toxicity, with PFOS (C8) being the most toxic followed by PFHxS (C6) and then PFBS (C4). Studies with 6:2 and 8:2 FTOHs resulted in less toxicity than other C6 PFAS compounds. The significance of this finding is unknown given that fluorotelomer substances comprise a vast number of different structures based on fluorocarbon chain and functional head group that could greatly influence their persistence, bioaccumulation, and toxicity. One example is 6:2 Cl‐PFAES, a major component of F‐53B, and a proposed alternative for PFOS in China that is approximately equivalent in toxicity to PFOS (Supplemental Data, Table SI‐3.18). This result perhaps might be expected because 6:2 Cl‐PFAES has physicochemical properties and environmental fate/transport characteristics similar to those of PFOS. The challenge in assuming that a replacement chemistry will be less toxic and/or persistent (with breakdown products having less toxicity) than the original PFAS is illustrated by GenX and ADONA, both intended as alternatives for PFOA. Based on a limited number of rat studies, ADONA has been shown to be less toxic than PFOA, with NOAELs based on reproductive endpoints of 1.3 and 90 mg/kg body weight/d, respectively (Gordon 2011). In contrast, GenX has been shown to be more toxic than PFOA and displays a level of toxicity in rats similar to that of PFOS based on equivalent serum and liver concentrations for these chemicals (Gomis et al. 2018). This finding highlights the importance of understanding the persistence and kinetics of replacement chemicals when one is assessing potential ecological risks.

When mammalian toxicity data are used in ecological risk assessments it is important to understand potential interspecies differences in sensitivity to a chemical of concern. This is a critical uncertainty, in part due to the limited number of species that have been tested for each PFAS as well as differences in experimental design and selected endpoints. However, given the large number of studies conducted with PFOS, some insights can be gained from an examination of common apical endpoints measured in these studies (Supplemental Data, Table SI‐3.19). Based on body weight/body weight gain effects, there were no discernible differences between species exposed to PFOS during gestation. Even when reproductive effects such as pup viability and/or weight gain are considered, PFOS effect levels between species were typically 10‐fold or less based on dose. Even so, due to the limited number of laboratory and mechanistic studies, extrapolation of toxicity data from these species to mammalian wildlife such as cetaceans and mustelids should be undertaken with caution, especially when nonapical endpoints are used, such as those related to neurological/behavioral endpoints as well as immunological function (Supplemental Data, Table SI‐3.20). For example, PFOS exposure has been shown to adversely affect several aspects of innate and cellular immune function in different rodent species dosed by gavage at concentrations approximately 1 to 2 orders of magnitude lower than those causing apical effects. Similarly, adverse effects have been observed in in vitro bioassays using peripheral blood leukocytes collected from bottlenose dolphins (*Tursiops truncatus*), in which PFOS exposure was associated with an increase in pro‐inflammatory interferon‐γ and an increased susceptibility to disease (Soloff et al. 2017). The linkages of PFAS effects on immune systems in mammalian wildlife have not yet been fully established; the possible implications of compromised immune function in susceptible species is an area that deserves additional investigation and inclusion in ecological risk assessments.

### Field‐based effects studies in wildlife

Measurable tissue concentrations occur in a wide array of organisms at different trophic levels. In addition, many studies have reported the toxicological effects of at least some PFAS in laboratory organisms representing a subset of taxa that are or may be exposed to PFAS in the field. There are, however, few examples in which the effects of PFAS on organisms in the field have been explored. As of the writing of the present review, 39 articles had been identified from the published literature in which effects of PFAS were investigated in wild‐caught animals. Of these published studies, 19 were conducted in avian species, 11 in mammals, and only 3, 2, and 2 in reptile, fish, and invertebrate species, respectively. When we consider the volume of research conducted on PFAS, there are clearly comparatively few studies on effects in free‐living animals. In part, this likely reflects some of the difficulties associated with conducting field studies, which often present logistical challenges with sampling and are usually costly; in addition the results may be confounded or influenced by a myriad of other factors including non‐PFAS chemical stressors. Nonetheless, field studies can provide a critical perspective on whether actual exposures in free‐living organisms can reach levels that cause observable adverse effects. In addition, these studies can generate hypotheses leading to new lines of research and questioning that advance our understanding of potential risks.

Field studies on avian species have generally focused on measuring PFAS primarily in plasma or eggs while also exploring effects ranging from changes in biochemical or immune parameters to endpoints closely related to reproductive success. One key challenge in conducting field studies lies in endpoint selection. Endpoints reported from studies on avian species have covered a range from suborganismal responses, including biomarkers and measures of oxidative stress (e.g., Blévin et al. 2017a, 2017b; Lopez‐Antia et al. 2017, 2019), to effects related to reproductive success such as eggshell thinning (Miljeteig et al. 2012) and hatching success (e.g., Tartu et al. 2014; Custer et al. 2019; Groffen et al. 2019). The avian studies that have included measures of reproductive success are especially valuable given the clear link between reproduction and potential population‐level impacts. Custer et al. (2012) conducted an early avian study with tree swallows and showed that the percentage of hatch decreased at approximately 150 ng PFOS/g wet weight in eggs collected from Lake Johanna, Minnesota (USA). In that study PFOS accounted for approximately 94% of the total PFAS measured. Similarly, Custer et al. (2014) showed the same decrease in hatching success in tree swallow eggs containing 150 ng PFOS/g wet weight from the upper Mississippi River near Minneapolis, Minnesota. In contrast, near a US Air Force base in Michigan, there was no impact on hatching success in tree swallow eggs at concentrations exceeding 600 ng PFOS/g, a value less than TRVs derived from laboratory studies (Custer et al. 2019). The authors suggested that previous observations of the effects of PFOS on hatching success may have been due to the presence of co‐contaminants present at the Minnesota locations. Groffen et al. (2019) showed decreasing trends in eggshell thickness and hatching success in great tit (*Parus major*) nesting along a PFAS contamination gradient (measured in eggs and the environment) emanating from a former fluorochemical manufacturing plant. However, the associations between egg PFAS concentration and the magnitude of effect were less than that observed in swallows (Custer et al. 2012, 2014). Tartu et al. (2014) also reported a negative association between plasma PFDoA (C12) concentrations in black‐legged kittiwakes (*Rissa tridactyla*) and hatching success. Collectively, avian field effects data indicate that PFAS are associated with impacts on a variety of suborganismal endpoints as well as reproductive endpoints, although mechanism‐based quantitative linkages between exposure and effect have not yet been established.

Field studies on the effects of PFAS in mammals similarly have included a suite of different endpoints in a variety of species including megafauna such as polar bears (*Ursus maritimus*) and cetaceans. More so than in birds, researchers have noted significant associations between PFAS concentrations and effects in these species. In polar bears, significant correlations were observed between brain concentrations of PFOS and PFCAs with changes in a suite of enzymes integral to the neurochemical transmitter system (Pedersen et al. 2015). Kidney concentrations of summed PFCAs, summed PFSAs, and 4 specific PFSAs from 16 species of cetaceans that had been stranded on beaches in Hawaii were significantly correlated with PPARα and cytochrome P4504a (*cyp4a*) expression in liver (Kurtz et al. 2019). Correlations between PFAS and the status of different immunologic parameters or apparent immunologic function have been investigated in walruses (*Odobenus rosmarus*; Scotter et al. 2019), bottlenose dolphins (*Tursiops trancatus*; Fair et al. 2013), and sea otters (*Enhydra lutris*; Kannan et al. 2006). Collectively, the studies with mammals in the field consistently show an association between PFAS exposure and alterations in biomarkers of exposure and effect. However, additional research is needed to relate changes in these endpoints to apical endpoints that have been more commonly used in ecological risk assessment as well as incorporating other approaches such as environmental epidemiology that have already been used in several cetacean studies (e.g., DeWitt et al. 2019).

There have been a few PFAS‐oriented field effects studies in reptiles, and none in amphibians. Studies measuring PFAS in tissues of field‐collected reptiles have included freshwater turtles, sea turtles, and crocodiles, but there have been few published studies with field‐collected lizards or snakes. One exception is an AFFF site in Australia that measured PFOS concentrations in snakes (Richards et al. 2018). Concentrations of PFAS have been associated with alterations in biomarkers and some apical endpoints in several reptile species. In a study with diamondback terrapins (*Malaclemys terrapin*), reduced biomass was associated with PFOS and PFNA in males whereas PFHxS was associated with reduced biomass in both males and females (Bangma et al. 2019). Possible adverse effects of PFOS on uroporphyrin concentrations were noted in loggerhead turtles (*Caretta caretta*) collected from the Mediterranean Sea (Guerranti et al. 2014). In an evaluation of leatherback, hawksbill, green sea, Kemp Ridley, and loggerhead turtles, there was a correlation of PFOS with responses indicative of immunosuppression, including a decreased T‐cell–dependent IgM antibody response (Keller et al. 2012).

To date, few field studies have been conducted that measured both tissue PFAS concentrations and effects on fish populations. Based on liver concentrations of 15 PFAS in wild‐caught striped mullet (*Mugil cephalus*), Bangma et al. (2018) did not observe any adverse relationships between liver concentrations and measures of reproductive success; they did, however, observe a positive relationship between liver concentrations and fecundity (number of eggs), which the authors attributed to greater food consumption. In Etobicoke Creek, Toronto (ON, Canada), enlarged livers, depressed peroxisomal β‐oxidation, and hepatic oxidative stress were observed in fish collected downriver 9 d after an AFFF release; however, liver PFAS concentrations and other biomarker responses returned to baseline levels within 120 d of the release (Oakes et al. 2010). The authors concluded that PFAS exposure did not result in substantial long‐term adverse effects. A recent study by Guillette et al. (2020) measured serum concentrations of 23 PFAS in striped bass (*Morone saxatilis*) from the Cape Fear River (North Carolina, USA) and correlated residue concentrations with health‐related serum parameters and biomarkers in the liver and immune system. Significant associations were observed between the various endpoints and PFAS concentrations (which were dominated by PFOS). Clearly, more data on whether exposure to PFAS in the field causes adverse effects in fish are warranted.

Two studies were identified in which the effects of PFAS on wild invertebrates were evaluated. The stream macrobenthic community was assessed upstream and downstream of a PFOA discharge point related to a fluorotelomer plant in Northern Italy (Rusconi et al. 2015). At this site there was some evidence of an ecological impact based on the presence of more sensitive taxa upstream compared with downstream locations; however, when the data were examined in a multimetric index, no significant differences were observed. The authors also included a measure of genetic diversity for a tricoptera species (*Hydropsyche modesta*) and found 6.8% variation between the sites that suggested some genetic drift in the downstream population; however, the genetic diversity could not be causally linked to PFAS exposure. In a study conducted in Norway, field monitoring data near former fire training facilities were used to propose PFOS sediment quality criteria thresholds for a range of effects from no anticipated effects to probable acute effects (Norwegian Pollution Control Authority 2008; Bakke et al. 2010).

Collectively, data on the effects of PFAS on wildlife are few and varied. Certainly, PFAS exposures occur in the field, some of which have been correlated with a wide array of suborganismal and, occasionally, apical effects (e.g., reproduction in avian species). Interpretation of field studies, however, is challenging in that it is possible that other unidentified factors contribute to observed effects, and that any effects observed are influenced by the choice of endpoint. For example, more reproductive studies are conducted with bird taxa because nests can be relatively easy to sample and monitor. Longer duration studies may be useful in identifying potentially subtle but meaningful effects of PFAS. In general, more field studies in which effects are investigated would be beneficial in building the dataset and our collective understanding of how PFAS may affect wild animal populations. In addition, evaluation of the concordance of effects in laboratory studies (e.g., with invertebrates, fish, birds, etc.) versus observed effects in field and monitoring studies with similar species would be useful.

## NEW APPROACH METHOD APPLICATIONS IN PFAS ECOLOGICAL RISK ASSESSMENT

One of the most significant challenges posed by PFAS relative to assessing ecological risk is the large number of chemicals for which few or no data exist concerning potential effects. However, PFAS represent only a comparatively small subset of the chemical universe for which this is a dilemma. Over the last 15 yr, there has been increasing awareness in both the scientific and regulatory communities that too little is known concerning the possible human health and ecological hazards of the great majority of chemicals in commerce and/or the environment (National Academy of Sciences 2007). Furthermore, this realization is occurring against a backdrop of diminishing resources for toxicity testing, as well as a desire to optimize/reduce animal use in toxicology. Consequently, there has been an emphasis on the development and implementation of novel approaches to support predictive chemical safety assessment (National Academy of Sciences 2007). These approaches include knowledgebases to summarize and communicate diverse biological or toxicological information, and generation of data using new approach methods (NAMs) such as in vitro bioactivity assays (including those conducted in an HTT format), short‐term in vivo tests with pathway‐specific molecular/biochemical endpoints, and bioinformatic/computational models. Several of these pathway‐based predictive toxicology avenues offer opportunities to address data gaps and uncertainties associated with assessment of the ecological risk of PFAS.

An example of an open‐access knowledgebase supporting both empirical and predictive analyses of the ecological effects of PFAS is ECOTOX, which was employed to assist in the literature assessment described in the previous section, *Current Knowledge about Ecological Effects of PFAS*. In some capacity, ECOTOX has been available to scientists and risk assessors for more than 40 yr (US Environmental Protection Agency 2020b). However, recent developments in terms of literature searching, data management, and information interfacing have resulted in a system well suited to handling the ever increasing amount of ecotoxicity data available in the open literature. Significantly, curators of the knowledgebase have flagged PFAS studies as high priority, so the quarterly ECOTOX updates should capture the latest relevant test data. In addition to providing risk assessors with ready access to the latest ecotoxicological data for specific taxa, ECOTOX output is being employed in more of a predictive capacity relative to PFAS, such as the generation of species sensitivity distributions (SSDs) for effects‐based benchmarks and, as described in greater detail later in this section, the development of approaches to predict the effects of PFAS when test data are limited.

Due to resource limitations, the lack of biological effects data for most PFAS cannot be readily addressed through conventional in vivo testing. Hence, there is a need for methods that enable rapid determination of the potential bioactivity of both single PFAS and their mixtures. Moreover, if measures of bioactivity can lend insights as to the mechanisms through which PFAS might exert toxicity, this would enhance the identification of susceptible species/endpoints for focused testing, as well as support cross‐species extrapolation of possible adverse effects. Various in vitro and in vivo test systems have already been employed, at least to some degree, to identify pathway‐specific bioactivities of a variety of PFAS (see Lee et al. 2019 for a recent review). As one example of this type of work, the USEPA is conducting an effort to test approximately 130 different PFAS in different HTT assays, including Attagene, a commercially available multiplexed platform that assesses approximately 70 different biological pathways, many of which have nuclear receptors as transcription factors (Romanov et al. 2008; Martin et al. 2010). Although the Attagene system currently employs mammalian gene constructs, many of the biological targets are structurally well conserved across vertebrates (LaLone et al. 2018), suggesting that PFAS activity data from the analyses should have broad applicability at least across these animal classes. Different in vitro and/or in vivo screening‐level assays with nonmammalian species have also been used to assess the bioactivity of PFAS. For example, various studies have evaluated “global” changes in gene or protein expression in nonmammalian systems (cells or intact animals) to help identify biological pathways/processes affected by exposure to PFAS (e.g., O'Brien et al. 2011, 2013; Cassone et al. 2012b; Huang et al. 2012; Zhang et al. 2012a; Chen et al. 2014; Jantzen et al. 2016; Dusgupta et al. 2019; Lee et al. 2019; Khazaee et al. 2020). Although many of these omics studies have to date focused on high‐visibility compounds like PFOS and PFOA, the approaches would also be useful for identifying key biological activities associated with less well‐characterized PFAS.

New approach methods potentially useful to the ecological assessment of PFAS also include different computational/bioinformatic approaches. For example, Cheng and Ng (2019) recently described a machine learning‐based approach to develop quantitative structure–activity relationships (QSARs) to predict specific bioactivities of almost 3500 different PFAS. There are also computational methods that enable consideration of specific uncertainties related to predicting the ecological effects of PFAS. One example is an on‐line tool called SeqAPASS (Sequence Alignment to Predict Across Species Susceptibility), which aims to enhance prediction of the biological effects of chemicals across diverse taxonomic groups (US Environmental Protection Agency 2019). The tool employs data from an exhaustive protein sequence database maintained by the National Center for Biotechnology Information (2010) to determine the degree of cross‐species structural conservation of specific protein targets of chemicals (e.g., receptors, enzymes, transporters). If the protein target(s) of a given chemical can be identified, SeqAPASS provides the basis for a first‐level analysis of the relative potential susceptibility of different species to a given chemical/chemical class (e.g., estrogen receptor agonists; Ankley et al. 2016). In this context, the tool provides a technical basis on which to determine the degree to which chemical‐specific data from mammalian assay systems (e.g., the HTT tests used with model PFAS) are suitable for helping assess effects of the test chemicals in nonmammalian species (e.g., LaLone et al. 2018).

Although the types of NAMs described above can produce effects data more efficiently and rapidly than conventional whole‐animal tests, there remains the task of translating this mechanistic information into the apical endpoints meaningful to risk assessors/managers. To help address this translation challenge, the adverse outcome pathway (AOP) concept was proposed by Ankley et al. (2010). The AOP framework provides a transparent depiction of causal linkages between initial chemical perturbation of a biological system (the molecular initiating event [MIE]) and subsequent measurable changes at progressively higher levels of biological organization (key events) that ultimately lead to an adverse outcome in individuals or populations. The framework also serves as an effective tool for curating and integrating diverse data‐streams (e.g., chemical effects data from in vitro and in vivo systems, physiological information) in a manner conducive to supporting predictive approaches to effects assessment (Leist 2017; Ankley and Edwards 2018). The AOP concept has received considerable attention in terms of application to chemical research and regulation. For example, in 2012 the OECD initiated an effort to support development and use of AOPs for chemical safety assessments (Organisation for Economic Co‐operation and Development 2019). This has resulted in formalized guidelines for deriving and describing AOPs, including weight‐of‐evidence approaches for their evaluation, and a knowledgebase for developing and archiving AOP information. An important component of this knowledgebase is the AOP wiki, an open‐source tool that currently contains more than 300 AOPs pertinent to human health and the environment (Society for the Advancement of Adverse Outcomes Pathways 2016). Given the needs and types of data likely to be used for ecological risk assessment of PFAS, the AOP framework provides a logical basis for data integration, translation, and communication.

## SELECT PFAS ECOLOGICAL RISK ASSESSMENT ACTIVITIES FROM AROUND THE WORLD

To date risk assessments within regulatory frameworks have largely focused on conducting nationwide monitoring assessments and developing effects‐based thresholds/benchmarks to support retrospective assessments. There are only limited established thresholds available from around the world for the effects of PFAS in the environment. Most thresholds are for direct effects of PFOS in freshwater aquatic species. Publicly available data from standardized tests with model test species conducted under laboratory conditions (see the previous section, *Current Knowledge about Ecological Effects of PFAS*) serve as the basis for most of the established thresholds. Values vary due to differences in the final data sets selected, methodologies for threshold development, and regional regulatory frameworks. A summary of available regulatory values and other thresholds for PFAS from around the world is provided in Table 1. A brief description of the development of these values and other aspects of PFAS regulation and risk assessment within several countries/regions around the world follows.

**Table 1:** Per‐ and poly‐fluoroalkyl substance regulatory thresholds and screening values for ecological effects from around the world

Jurisdiction	Source	Chemical common name	Threshold value	Threshold unit	Threshold type and other notes
Minnesota, USA	Stevens and Coryell (2007a)	PFOS	19	µg/L	ccc
Minnesota, USA	Stevens and Coryell (2007b)	PFOA	1,700	µg/L	ccc
Michigan, USA	Michigan Department of Environmental Quality (2017)	PFOS	140	µg/L	Tier II FCV
Michigan, USA	Michigan Department of Environmental Quality (2017)	PFOS	780	µg/L	Tier II AMV
Michigan, USA	Michigan Department of Environmental Quality (2017)	PFOS	1,600	µg/L	Tier II FAV
Michigan, USA	Michigan Department of Environmental Quality (2017)	PFOA	880	µg/L	Tier II FCV
Michigan, USA	Michigan Department of Environmental Quality (2017)	PFOA	7,700	µg/L	Tier II AMV
Michigan, USA	Michigan Department of Environmental Quality (2017)	PFOA	15,000	µg/L	Tier II FAV
Michigan, USA	Michigan Department of Community Health (2015)	PFOS	0.035	µg/L	Surface water value protective of avian wildlife
Michigan, USA	Michigan Department of Community Health (2015)	PFOS	0.084	µg/L	Surface water value protective of mammalian wildlife
Canada	Environment and Climate Change Canada (2018)	PFOS	6.8	µg/L	FEQG, surface water
Canada	Environment and Climate Change Canada (2018)	PFOS	9.4	mg/kg wet wt	FEQG, fish tissue based on the fish data and bioaccumulation factors for bluegill from Drottar et al. (2002)
Canada	Environment and Climate Change Canada (2018)	PFOS	8.2	µg/kg wet wt food	Dietary value for avian wildlife
Canada	Environment and Climate Change Canada (2018)	PFOS	4.6	µg/kg wet wt food	Dietary value for mammalian wildlife
Canada	Environment and Climate Change Canada (2018)	PFOS	1.9	µg/g wet wt	Whole egg value protective of avian wildlife
Canada	Environment Canada (2012b)	PFOA	20	µg/L	PNEC for freshwater pelagic organisms
Canada	Environment Canada (2012b)	PFOA	30	µg/L	PNEC for rare freshwater minnows
Canada	Environment Canada (2012b)	PFOA	158	µg/kg wet wt food	PNEC for dietary concentrations of mammalian wildlife
European Union	European Commission (2011, 2013)	PFOS	36	µg/L	MAC‐EQS (freshwater)
European Union	European Commission (2011, 2013)	PFOS	7.2	µg/L	MAC‐EQS (saltwater)
European Union	European Commission (2011)	PFOS	0.23	µg/L	QS (freshwater pelagic community)
European Union	European Commission (2011)	PFOS	0.023	µg/L	QS (saltwater pelagic community)
European Union	European Commission (2011)	PFOS	0.033	mg/kg wet wt	QS_biota_ (secondary poisoning: predators)
European Union	European Commission (2011)	PFOS	0.002	µg/L	QS_freshwater_: freshwater concentration leading to secondary poisoning in predators
European Union	European Commission (2011)	PFOS	0.00047	µg/L	QS_saltwater_: saltwater concentration leading to secondary poisoning in predators
European Union	European Commission (2011, 2013)	PFOS	9.1	µg/L	EQS (biota)
European Union	European Commission (2011, 2013)	PFOS	0.00065	µg/L	AA‐EQS (freshwater)
European Union	European Commission (2011, 2013)	PFOS	0.00013	µg/L	AA‐EQS (saltwater)
Italy	Valsecchi et al. (2017)	PFOA	2220	µg/L	MAC‐EQS (freshwater)
Italy	Valsecchi et al. (2017)	PFOA	450	µg/L	MAC‐EQS (saltwater)
Italy	Valsecchi et al. (2017)	PFOA	30	µg/L	QS (based on direct toxicity to freshwater pelagic community)
Italy	Valsecchi et al. (2017)	PFOA	3	µg/L	QS (based on direct toxicity to saltwater pelagic community)
Italy	Valsecchi et al. (2017)	PFOA	0.9	µg/kg wet wt	QS_biota_ (secondary poisoning: predators)
Italy	Valsecchi et al. (2017)	PFOA	0.1	µg/L	QS_freshwater_: freshwater concentration leading to secondary poisoning in predators
Italy	Valsecchi et al. (2017)	PFOA	0.02	µg/L	QS_saltwater_—saltwater concentration leading to secondary poisoning in predators
Italy	Italian Parliament (2015); Valsecchi et al. (2017)	PFOA	0.1	µg/L	AA‐EQS (freshwater) for RBSP at the national level
Italy	Italian Parliament (2015); Valsecchi et al. (2017)	PFOA	0.02	µg/L	AA‐EQS (saltwater) for RBSP at the national level
Italy	Valsecchi et al. (2017)	PFBA	1100	µg/L	MAC‐QS (freshwater)
Italy	Valsecchi et al. (2017)	PFBA	110	µg/L	MAC‐QS (saltwater)
Italy	Valsecchi et al. (2017)	PFBA	110	µg/L	QS (based on direct toxicity to freshwater pelagic community)
Italy	Valsecchi et al. (2017)	PFBA	11	µg/L	QS (based on direct toxicity to saltwater pelagic community)
Italy	Italian Parliament (2015); Valsecchi et al. (2017)	PFBA	7	µg/L	AA‐EQS (freshwater) for RBSP at the national level
Italy	Italian Parliament (2015); Valsecchi et al. (2017)	PFBA	1.4	µg/L	AA‐EQS (saltwater) for RBSP at the national level
Italy	Valsecchi et al. (2017)	PFPeA	3200	µg/L	MAC‐QS (freshwater)
Italy	Valsecchi et al. (2017)	PFPeA	320	µg/L	MAC‐QS (saltwater)
Italy	Valsecchi et al. (2017)	PFPeA	32	µg/L	QS (based on direct toxicity to freshwater pelagic community)
Italy	Valsecchi et al. (2017)	PFPeA	3.2	µg/L	QS (based on direct toxicity to saltwater pelagic community)
Italy	Italian Parliament (2015); Valsecchi et al. (2017)	PFPeA	3	µg/L	AA‐EQS (freshwater) for RBSP at the national level
Italy	Italian Parliament (2015); Valsecchi et al. (2017)	PFPeA	0.6	µg/L	AA‐EQS (saltwater) for RBSP at the national level
Italy	Italian Parliament (2015); Valsecchi et al. (2017)	PFHxA	1	µg/L	AA‐EQS (freshwater) for RBSP at the national level
Italy	Italian Parliament (2015); Valsecchi et al. (2017)	PFHxA	0.2	µg/L	AA‐EQS (saltwater) for RBSP at the national level
Italy	Valsecchi et al. (2017)	PFBS	3720	µg/L	MAC‐EQS (freshwater)
Italy	Valsecchi et al. (2017)	PFBS	372	µg/L	MAC‐EQS (saltwater)
Italy	Valsecchi et al. (2017)	PFBS	372	µg/L	QS (based on direct toxicity to freshwater pelagic community)
Italy	Valsecchi et al. (2017)	PFBS	37	µg/L	QS (based on direct toxicity to saltwater pelagic community)
Italy	Italian Parliament (2015); Valsecchi et al. (2017)	PFBS	3	µg/L	AA‐EQS (freshwater) for RBSP at the national level
Italy	Italian Parliament (2015); Valsecchi et al. (2017)	PFBS	0.6	µg/L	AA‐EQS (saltwater) for RBSP at the national level
Australia and New Zealand	National Chemicals Working Group (2020)	PFOS	31	µg/L	80% species protection value (draft freshwater DGV and also to be used as an interim draft saltwater DGV)
Australia and New Zealand	National Chemicals Working Group (2020)	PFOS	2	µg/L	90% species protection value (draft freshwater DGV and also to be used as an interim draft saltwater DGV)
Australia and New Zealand	National Chemicals Working Group (2020)	PFOS	0.13	µg/L	95% species protection value (draft freshwater DGV and also to be used as an interim draft saltwater DGV)
Australia and New Zealand	National Chemicals Working Group (2020)	PFOS	0.00023	µg/L	99% species protection value (draft freshwater DGV and also to be used as an interim draft saltwater DGV)
Australia and New Zealand	National Chemicals Working Group (2020)	PFOA	1,824	µg/L	80% species protection value (draft freshwater DGV and also to be used as an interim draft saltwater DGV)
Australia and New Zealand	National Chemicals Working Group (2020)	PFOA	632	µg/L	90% species protection value (draft freshwater DGV and also to be used as an interim draft saltwater DGV)
Australia and New Zealand	National Chemicals Working Group (2020)	PFOA	220	µg/L	95% species protection value (draft freshwater DGV and also to be used as an interim draft saltwater DGV)
Australia and New Zealand	National Chemicals Working Group (2020)	PFOA	19	µg/L	99% species protection value (draft freshwater DGV and also to be used as an interim draft saltwater DGV)
Australia and New Zealand	National Chemicals Working Group (2020)	PFOS + PFHxS, PFOS only, PFHxS only	8.2	µg/kg wet wt food	Dietary value for avian wildlife, based on Environment and Climate Change Canada (2018)
Australia and New Zealand	National Chemicals Working Group (2020)	PFOS + PFHxS, PFOS only, PFHxS only	4.6	µg/kg wet wt food	Dietary value for mammalian wildlife, based on Environment and Climate Change Canada (2018)
Australia and New Zealand	National Chemicals Working Group (2020)	PFOS + PFHxS, PFOS only, PHxS only	0.2	µg/g wet wt	Whole egg value protective of avian wildlife, adapted from Environment and Climate Change Canada (2018) using an additional uncertainty factor
Australia and New Zealand	National Chemicals Working Group (2020)	PFOS	1	mg/kg	Interim soil DGV for public open space, protective of direct exposure
Australia and New Zealand	National Chemicals Working Group (2020)	PFOS	0.01	mg/kg	Interim soil DGV: indirect exposure, applicable to all land uses; criterion is based on dietary exposure of a secondary consumer as the most sensitive exposure pathway assessed, based on Environment and Climate Change Canada (2018)
Australia and New Zealand	National Chemicals Working Group (2020)	PFOS	0.14	mg/kg	For intensively developed sites with no secondary consumers and minimal potential for indirect ecological exposure, a higher criterion may be used to trigger a detailed site‐specific investigation of risk
Australia and New Zealand	National Chemicals Working Group (2020)	PFOA	10	mg/kg	Interim soil DGV for public open space, protective of direct exposure

AA‐EQS = annual average environmental quality standard; AMV (tier II) = aquatic maximum value calculated based on tier II methodology of US Environmental Protection Agency (1985) guidelines; Biota_sec pois_ = standard for concentration in fish tissue protective of secondary poisoning in predators; ccc = continuous chronic criterion; DGV = default guideline value; EQS = environmental quality standard; FAV (tier II) = final acute value calculated based on tier II methodology of US Environmental Protection Agency (1985) guidelines; FCV (tier II) = final chronic value calculated based on tier II methodology of US Environmental Protection Agency (1985) guidelines; FEQG = federal environmental quality guidelines; MAC‐EQS = maximum acceptable environmental quality standard; PFBA = perfluorobutanoate or perfluorobutanoic acid; PFBS = perfluorobutane sulfonate or perfluorobutanesulfonic acid; PFHxA = perfluorohexanoic acid; PFOA = perfluorooctanoate or perfluorooctanoic acid; PFOS = perfluorooctane sulfonate or perfluorooctanesulfonic acid; PFPeA = perfluoropentanoate or perfluoropentanoic acid; PNEC = predicted no‐effect concentration; QS = quality standard; QS_biota_ = specific quality standard for concentration in prey biota tissue protective of secondary poisoning in predators; QS_freshwater_ = specific quality standard for concentration in fresh water protective of secondary poisoning in predators; QS_saltwater_ = specific quality standard for concentration in marine water protective of secondary poisoning in predators; RBSP = river‐based specific pollutant.

### Canada

Environment and Climate Change Canada has published national‐scale ecological risk assessments for PFOS, PFOA, and long‐chain (C9–C20) PFCAs, and their salts and precursors (Environment Canada 2006, 2012a, 2012b). These assessments concluded, overall, that PFAS were entering or may enter the environment in a quantity or concentration or under conditions that have or may have an immediate or long‐term harmful effect on the environment or its biological diversity. The conclusions were based on a weight‐of‐evidence approach that considered persistence, bioaccumulation, increasing temporal trends in residues in some species (e.g., polar bears), long‐range transport, and the widespread occurrence of these substances in the Canadian environment (including the Great Lakes and the Arctic) and in biota. Consequently, PFOS, PFOA, and long‐chain PFCAs have been placed on Canada's List of Toxic Substances, a step that allowed risk management actions to be taken (Environment Canada 2012c).

In addition, different Canadian Federal Environmental Quality Guideline (FEQG) values have been developed for PFOS (Table 1), and comparable values for PFOA are currently under development. These guidelines are benchmarks for the quality of the ambient environment and can be used for comparison with environmental monitoring results, or to assist in evaluating the performance of remedial measures. They are voluntary unless adopted into regulation. Several FEQG values for PFOS for use by Federal departments were published in 2018 for surface water for the protection of freshwater aquatic life, fish tissue, wildlife diet for mammalian and avian consumers, and in bird eggs (Environment and Climate Change Canada 2018). The freshwater aquatic life value is a 95% protection value based on an empirical SSD using 20 species. The dietary item thresholds are based on food‐chain models with conservative assumptions for representative avian (Wilson's storm petrel) and mammalian (mink) species consuming fish. In addition, in 2018 the Canadian Council of Ministers of Environment developed and released for public comment, draft FEQG values for PFOS in soil and groundwater for the assessment and remediation of contaminated sites. These guidelines are currently undergoing final approval.

### Australia and New Zealand

Draft freshwater default guideline values (DGVs) for PFOS and PFOA were developed through the Water Quality Guideline Framework toxicant DGV publication approval process, using the method developed for revising the Australian and New Zealand Guidelines for Freshwater and Marine Water Quality (Table 1; Warne et al. 2018). The values are based on chronic laboratory studies that link exposure to direct adverse effects in aquatic organisms. The derived DGVs are not mandatory and have no formal legal status, but state, territory, or local jurisdictions may incorporate DGVs into their water quality protection policies and regulatory tools. Although not an assumption that harm will occur, an exceedance of the DGVs in a water body or inputs to a waterbody may trigger further investigation such as site‐specific risk assessments to better define possible risks, including risk through food web exposure. The draft freshwater DGVs for PFOS were derived from the results of 18 reliable chronic toxicity studies on test species comprising algae, crustaceans, insects, fish, and amphibians, including 4 longer term partial life cycle, full life cycle, or multigenerational studies considered relevant to persistent, bioaccumulative, and toxic (PBT) chemicals. Based on the SSDs from these data, draft DGVs were established at 80, 90, 95, and 99% protection levels.

For toxicants that are not bioaccumulative, a 95% species protection level is recommended in the Guidelines to ensure protection of a slightly to moderately disturbed aquatic ecosystem (Australian Government 2018). However, Australia's National Environmental Management Plan for PFAS (Australian and New Zealand Environment and Conservation Council/Agriculture and Resource Management Council of Australia and New Zealand 2000; Australian Government 2018; National Chemicals Working Group, Heads of EPA Australia and New Zealand 2018, 2020) recommends that a more restrictive 99% species protection level be used as a guideline for PFOS. By extension, the 95% species protection DGV could be used for highly disturbed ecosystems instead of the 90% species protection DGV. A lower level of protection than this for highly disturbed sites is not recommended for chemicals like PFOS that bioaccumulate and potentially biomagnify in wildlife.

The Cooperative Research Center for Contamination Assessment and Remediation of the Environment (2017) in Australia published draft marine values for PFOS and PFOA, using the same guidelines. These marine values were not included in the National Environmental Management Plan (National Chemicals Working Group, Heads of EPA Australia and New Zealand 2018). Freshwater DGVs are to be used on an interim basis for marine systems until final marine quality values can be set using the nationally agreed‐on process under the Australian and New Zealand Guidelines for Freshwater and Marine Water Quality (National Chemicals Working Group, Heads of EPA Australia and New Zealand 2018, 2020). Based on a literature review and increase in the number of ecotoxicity studies published since 2015, the DGVs are being revised and updated with an increased number of species in the SSD for PFOS and PFOA. These revised DGVs are currently under review.

### European Union

A number of thresholds for PFOS are available from the European Union (Table 1), as described in the Environmental Quality Standard (EQS) Dossier for PFOS (European Commission 2011). These include maximum acceptable EQS values for freshwater and marine ecosystems, and annual average EQS values for the same ecosystems. Standards are also available for the freshwater pelagic community, the marine pelagic community, and predators via secondary poisoning (i.e., consideration of biomagnification through the consumption of contaminated prey). In 2013, PFOS was included in the amended list of priority substances of Directive 2000/60/EC as set forth in Directive 2013/39/EU (European Commission 2013), and EQS values were set accordingly.

On 14 June 2013, the Member State Committee, referred to in Article 76(1)(e) of Regulation (EC) No 1907/2006 (REACH; European Commission 2006), identified PFOA as a PBT substance in accordance with Article 57(d) of that Regulation. On 20 June 2013, PFOA was included in the Candidate List of Substances of Very High Concern for possible inclusion in Annex XIV to the REACH regulation. In this framework, a support document was prepared for identification of PFOA as a substance of very high concern because of its carcinogenic, mutagenic, or reprotoxic and PBT properties. An environmental hazard assessment and PBT assessment (relevant for ecological risk assessment) are reported in the same support document.

Italy has also developed EQSs for PFOA, PFBS, PFBA, perfluoropentanoate, and PFHxA (Table 1; Valsecchi et al. 2017) employing European Technical Guidance for Deriving Environmental Quality Standards (European Commission 2018). Some of the standards were adopted as River Based Specific Pollutants at the national level in Italian Legislative Decree 172/2015 (Italian Parliament 2015; see also Valsecchi et al. 2017).

Research on effects in benthic invertebrates after direct exposure to sediments contaminated with PFAS is limited, and there are no published benchmarks for this environmental matrix. In its EQS Dossier for PFOS (European Commission 2011), the European Union took the position that insufficient data are available to confirm the need for a sediment quality standard or to derive a threshold, thus electing not to develop a value. Similarly, a workgroup in Northern Italy concluded that there was no need for a sediment EQS for PFOA, PFBS, PFBA, or perfluoropentanoate and that data for a sediment EQS for PFHxA were insufficient to derive a sediment EQS (Valsecchi et al. 2017). However, observational data and monitoring have been used in Norway to develop an understanding of which exposure may be associated with adverse effects. A no‐effect threshold of 220 µg PFOS/kg sediment dry weight, a chronic toxicity range of 220 to 630 µg PFOS/kg sediment, and an acute short‐term effects range of 630 to 3100 µg PFOS/kg sediment were established (Norwegian Pollution Control Authority 2008; Bakke et al. 2010). Although these Norwegian sediment thresholds were proposed for PFOS in marine sediments, they may also provide some basis for screening‐level risk decisions for freshwater sediments.

### United States

A USEPA Per‐ and Polyfluoroalkyl Substances (PFAS) Action Plan was first released in February 2019, and was updated a year later (US Environmental Protection Agency 2020a). The focus was primarily on human health risk, although some of the actions, such as source control, reducing discharges to waterways, and remedial actions, will benefit the environment as well. The plan included some longer term actions that are specific to ecological risk including identifying sensitive or susceptible species, developing a better understanding of bioaccumulation through food chains, and developing ecological risk‐based thresholds.

There are not yet ecological risk‐based guideline values for any PFAS from the US Federal Government. However, the USEPA is evaluating published toxicity data to determine whether they are sufficient to develop national water quality criteria for PFAS in aquatic environments for aquatic life (e.g., fish, invertebrates) and aquatic‐dependent wildlife species (e.g., birds). The US national water quality criteria are recommendations; states and authorized tribes may adopt the national criteria or develop their own scientifically defensible criteria, which the USEPA must review and approve before state/tribal use as regulatory standards. Preliminary analysis suggests that a sufficient quantity of data is available to develop single‐chemical freshwater aquatic life thresholds for PFOS and PFOA. Methods for developing criteria for other PFAS with little (or no) toxicity data are also being evaluated. The data evaluation by the USEPA for aquatic‐dependent wildlife has only recently been initiated, so there are no conclusions yet as to whether sufficient data exist to develop values for this endpoint. From the standpoint of bioaccumulation‐based guideline values for PFAS, food‐chain models like those used to develop the Canadian FEQGs are commonly used within the United States, including for retrospective risk assessments at contaminated waste sites. However, for the TRVs or bioaccumulation factors commonly used in food‐chain models, there are no USEPA established or approved values for PFAS. Although it is not a regulatory authority with respect to PFAS, the US Department of Defense has contracted the US Department of Energy's Argonne National Laboratory to develop ecological risk‐based screening values for use in site‐specific ecological risk assessments. The results of this ongoing effort could be available as early as 2020.

Some individual states have established criteria intended to protect aquatic organisms in surface waters (Table 1). For example, Michigan has established ambient water quality criteria for PFOS and PFOA based on Rule 57 (Michigan Department of Environmental Quality 2017). These include both final chronic values and aquatic maximum values somewhat analogous to the European Union's EQSs. The PFOA and PFOS numeric criteria for Michigan are all tier 2 values, meaning they are based on less than the full set of data requirements used for national water quality criteria (US Environmental Protection Agency 1985) or tier 1 values. Because these tier 2 values are based on a more limited amount of peer‐reviewed aquatic toxicity data, uncertainty factors are included, making them more stringent than tier 1 values. The Michigan Department of Community Health (2015) has also established provisional PFOS surface water values for mammalian wildlife, based on otter and mink characteristics and for avian wildlife based on eagle, kingfisher, and herring gull characteristics. The State of Minnesota has also derived continuous chronic criteria values for the protection of aquatic biota for PFOS and PFOA (Stevens and Coryell 2007a, 2007b). No other surface water values have been derived for PFAS in either of these states. Values for other states are currently unavailable but are underway in Florida, Wisconsin, New Hampshire, and Vermont.

## INTERACTIVE BREAKOUT SESSION: HIGHLIGHTING NEEDS FOR ASSESSING PFAS ECOLOGICAL RISKS

The first 6 sections of our review highlight current knowledge about PFAS properties, possible exposures, and potential effects in the context of ecological risk assessment and start to identify important knowledge gaps and uncertainties. Much of this information was introduced in the various presentations during the opening sessions of the Focused Topic Meeting (see Johnson et al. 2020). For the remainder of our review, we will be providing recommendations for addressing the various needs identified in prior sections. These recommendations are a result of facilitated discussions from a day and half ecological‐effects breakout session in which the coauthors of the present review served as expert panelists. The discussions specifically addressed questions and needs related to: 1) conducting exposure assessments for new and existing PFAS; 2) evaluation of the potential effects of PFAS entering or present in the environment; 3) employing pathway‐based approaches to help assess ecological hazards of PFAS; and 4) considering PFAS as mixtures in prospective and retrospective assessments. Although the expert panel initiated the deliberations on these topics/areas at the meeting, the many scientific, risk assessment, and other professionals who attended the breakout session as observers made substantial contributions to the discussion and, ultimately, aspects of the recommendations in the following sections. That is, the discussions were open, and the observers readily provided input based on their experiences and different organizational activities. Although it is not practically feasible to individually acknowledge the various contributions from the observers, Supplemental Data, Table SI‐4, lists the names and affiliations of the 90 plus meeting attendees who self‐identified with the ecological‐effects breakout session.

## NEEDS AND CONSIDERATIONS FOR ADVANCING EXPOSURE ASSESSMENT

### Environmental monitoring: Scope and opportunities

The environmental monitoring of PFAS in abiotic (e.g., air, water, sediment, soil) and biotic (e.g., fish, wildlife) matrices is essential to determine the global distribution of these chemicals. Ongoing and new exposure assessments should focus on suites of common PFAS (like PFOS and PFOA) as well as other less studied molecules such as short‐chain PFCAs and PFSAs (Ateia et al. 2019; Li et al. 2020), fluoroalkylether compounds (Munoz et al. 2019), and polyfluorinated alkane sulfonates. Ongoing efforts by organizations such as the OECD concerning categorization and prioritization of PFAS will help align the analyses to be conducted in specific samples.

Well‐designed long‐term studies with adequate statistical power are necessary to enable probabilistic exposure assessments and evaluate time‐trends of PFAS in the environment with respect to the effectiveness of regulatory mitigation measures. These studies should encompass freshwater, marine, and terrestrial environments and include impacted sites (i.e., hot spots) as well as remote locations influenced by long‐range transport of contaminants. The use of standardized targeted analytical methods can allow comparisons between sites and years. Recent analytical developments now also allow the use of suspect screening and nontargeted techniques for PFAS assessment. These evolving and promising techniques have already been used to explore complex mixtures and identify many unexpected PFAS, but sample preparation and data analysis procedures in many instances have yet to be standardized (Nakayama et al. 2019). Analytical standards are also needed to accurately identify and quantify novel PFAS.

Systematic monitoring programs should be implemented to routinely assess PFAS concentrations and the spatial extent of PFAS in multiple environmental matrices: soil, water, sediment, and biota. For example, in the United States, the National Water‐Quality Assessment and the National Water Quality Network programs coordinated by the US Geological Survey monitor for contaminants and water quality across a network of surface monitoring stations and wells, respectively. Incorporating PFAS analysis into these programs would provide a systematic assessment of PFAS distribution and concentrations that go beyond the various regional analyses already conducted (Bradley et al. 2018; Boone et al. 2019). Analogously, the National Oceanic and Atmospheric Administration National Status and Trends Mussel Watch Program has monitored contaminants in mussel tissues across coastal areas of the United States including Chesapeake Bay (Apeti et al. 2018) and the Great Lakes (Kimbrough et al. 2018). The program started in 1986 and has archived mussel samples that could be valuable for determining historical trends in PFAS concentrations. The Mussel Watch Program has already conducted studies in California that provided important data concerning PFAS contamination associated with urban areas (Dodder et al. 2014; Maruya et al. 2014). The Northern Contaminants Program in Canada has also been monitoring PFAS in different marine and terrestrial species (e.g., fish, seabirds, seal, belugas, polar bears, caribou) across the Canadian Arctic. This program allows time trends assessment of PFAS as well as the surveillance of additional PFAS entering these remote ecosystems. The data concerning the presence of PFAS in the Arctic environment represent the strongest evidence available of probable long‐range transport; this information is crucial to international agreements such as the Stockholm Convention on POPs. In the European Union, member states have been monitoring PFOS in biota (mainly fish) since 2018 at a minimum frequency able to provide sufficient data for a reliable long‐term trend analysis, as required by EQS Directive 2013/39/EU (European Commission 2013). Results of these measurements have allowed a robust assessment of the extent of PFOS contamination in European waters.

Formal sampling programs encompassing multiple trophic levels should also be developed to assess biomagnification of PFAS in different food webs. Specific criteria should be followed when trophic magnification studies are designed, as summarized by Kidd et al. (2019). The inclusion of organisms from the same habitat linked by diet with a balance between lower and higher trophic level species and the use of appropriate baseline organisms are important components of a testing program. Sample size, time of sampling, and tissue to be analyzed are also critical for the stable isotope and contaminant analyses that are the basis for understanding bioaccumulation in any system (Kidd et al. 2019). Again, there should be an emphasis on the collection of data for PFAS known to bioaccumulate/biomagnify as well as PFAS for which there is less information.

Finally, consideration should be given to evaluating novel environmental matrices possibly relevant to PFAS fate and transport. For example, sea surface microlayers and biofilms are a potentially critical partitioning matrix for PFAS but are not typically assessed in conjunction with contaminant monitoring. Microbes attached to biofilm surfaces are ubiquitous in nature and can be dominant in numbers and metabolic activity in nutrient‐sufficient aquatic ecosystems (Costerton et al. 1995). Bacteria encase themselves in a matrix comprised of polysaccharides, protein, lipids, and/or extracellular DNA depending on the organism (Flemming and Wingender 2010); PFAS are known to bind to proteins and other biopolymers (MacManus‐Spencer et al. 2009; Bischel et al. 2010). Biofilms collected in the field were found to contain higher concentrations of PFAS than sediments and to bioconcentrate PFAS from the water (Munoz et al. 2017). The PFAS were suspected of binding to the surface, as well as directly adsorbing to biopolymers such as protein extrapolymeric substances. Biofilms serve as a valuable food source for small invertebrates (Lawrence et al. 2002) and could alter bioavailability and exposure of higher trophic levels to PFAS via biofilm grazing. Although very little currently is known concerning PFAS partitioning in biofilms, a study by Bertin et al. (2016) found increased PFAS accumulation in *Gammarus* in the presence of detritus and biofilms. If biofilms are indeed an important exposure route for PFAS, this may considerably change the approaches used to assess and model PFAS bioaccumulation.

### Measuring and predicting bioaccumulation of PFAS

Understanding bioaccumulation is arguably the most significant exposure challenge relative to PFAS, with critical ramifications not only for ecological but also for human health effects. Empirical modeling approaches, utilizing field‐based data and biomagnification or trophic magnification factors (BMF and TMF respectively), and mechanistic modeling based on physicochemical properties, are important tools for predicting bioaccumulation and exposure levels (Armitage et al. 2014; Gobas et al. 2018). There are several important uncertainties in assessing and predicting the bioaccumulation of PFAS in aquatic organisms (Conder et al. 2008). Methods addressing early challenges such as matrix interferences and low analyte concentrations have, for the most part, been addressed when PFAS are analyzed in biological tissues (Lanza et al. 2017). However, substantial data exist showing that factors dictating bioaccumulation of PFAS are very different from those for persistent bioaccumulative contaminants such as PCBs, requiring different approaches and kinetic information to model bioaccumulation in freshwater ecosystems (Bertin et al. 2016; Prosser et al. 2016). For example, there are substantial differences in BAFs and biota–sediment accumulation factors (BSAFs) for PFAS compared with PCBs (Houde et al. 2006a; Giesy et al. 2010; Burkhard et al. 2012). Basically, the lipid‐based partitioning models typically used to predict the bioaccumulation of neutral organic compounds like PCBs are not appropriate for predictive models of PFAS bioaccumulation. In fact, several studies have demonstrated that BSAF values for PFAS should not be normalized to organism lipid content because of the potential binding of PFAS to proteins and biopolymers such as chitin (Higgins et al. 2007; Prosser et al. 2016).

As is the case for toxicity studies, PFOS has been the most commonly evaluated PFAS relative to bioaccumulation testing. Several studies have explored variables that may influence the bioaccumulation of PFOS in laboratory‐based assays, including exposure duration, PFOS test concentration, gut clearance, organic carbon composition, and organism‐specific feeding and life strategies. Some of these studies suggest that laboratory‐based predictions of bioaccumulation may not be applicable to the field. For example, standard bioaccumulation methods using sediment exposures with the freshwater oligochaete *Lumbriculus variegatus* may underpredict the bioaccumulation of PFAS by organisms in the environment because field exposures likely occur through both water uptake and sediment ingestion. Consistent with this hypothesis, the uptake rate of PFAS in bivalves was shown to be highly dependent on water concentration, but also binding of PFAS to algae and the filtration rate of the organisms (Haukås et al. 2007; Jeon et al. 2010a). Furthermore, standard 28‐d bioaccumulation assays using aquatic invertebrates (*L. variegatus, C. riparius, Gammarus* spp.) may not reach steady state for PFOS, PFNA, PFUnA, and PFDoA, so reliable BSAF values may not be obtained for these chemicals (Higgins et al. 2007; Bertin et al. 2014, 2016). Other factors that may affect results from standard bioaccumulation assays include the number of overlying water changes during the test (Prosser et al. 2016), feeding during testing (Haukås et al. 2007), and binding of PFAS to protein in food during the study (Weathers et al. 2015). Although not unique to PFAS, the development of environmentally realistic standard tests using relevant aquatic species with different feeding ecology (e.g., sediment dwelling, epibenthic, pelagic) is warranted to better evaluate bioaccumulation of PFAS via water and sediment exposure.

Predicting bioaccumulation of PFAS is perhaps even more challenging than conducting empirical measures of bioaccumulation. However, predictive models for bioaccumulation are critical tools for some risk assessment scenarios. For example, regulatory programs involved in prospective assessments of new chemicals that may be released into the environment (like Toxic Substances Control Act in the United States) rely almost exclusively on predictive models to define characteristics of compounds. As noted in the previous paragraph, the highly successful lipid‐based equilibrium partitioning models used for neutral organic chemicals are not appropriate for predicting bioaccumulation of many PFAS, because different portions of the molecule may be simultaneously hydrophobic, ionizing, hydrophilic, and lipophobic (Conder et al. 2008). Some portions of the perfluorinated molecule certainly can interact with lipids (Jing et al. 2009), but the presence of a functional group (e.g., carboxylate or sulfonate) can impart high hydrophilicity. The octanol/water partition coefficient (log *K*_OW_) has been widely used to describe the partitioning of neutral organic chemicals from water to the lipid of organisms, to predict bioaccumulation potential. Currently, a meaningful log *K*_OW_ value cannot be used for surface‐active and ionizing organic chemicals such as PFOS, PFOA, and shorter chain PFAS. Escher et al. (2020) noted that *K*_OW_‐based models developed for neutral chemicals could be adapted to ionizing organic chemicals by replacing the *K*_OW_ with the ionization‐corrected octanol/water distribution ratio (*D*_OW_ [pH]), assuming that the charged species plays no role in uptake. However, this assumption is not wholly justified for ionizing organic chemicals, including certain PFAS. Octanol is not a suitable surrogate for these types of chemicals given that anions have a higher affinity to proteins than their neutral counterparts (Escher et al. 2020). Models that utilize molecular structure to predict behavior in organic and aqueous solvents are capable of estimating log *K*_OW_ values for the neutral portion of PFAS (e.g., COSMOtherm; Wang et al. 2011). However, Ng and Hungerbühler (2014) have demonstrated that modeling bioaccumulation using the log *K*_OW_ of the neutral portion of a given PFAS alone fails as an accurate predictor of bioaccumulation potential, because many of these substances (e.g., PFCA, PFSA) are almost completely ionized at environmental pH values. Studies have also shown that, at the organismal level, protein‐rich tissues (such as yolk, liver, and blood), rather than lipids, are the primary repositories for some PFAS (Martin et al. 2003b; Houde et al. 2006a). The transport of these substances into cells is likely controlled by a combination of passive diffusion and active facilitation by transporter proteins such as organic anion transporter proteins (Ng and Hungerbühler 2013), resulting in binding to fatty acid–binding proteins and lipoproteins/albumin, and then sequestering in protein‐rich tissues (Cassone et al. 2012a). Thus protein‐partitioning coefficients, rather than log *K*_OW,_ may best describe the mechanisms governing partitioning for PFAS. There are, however, few wildlife protein‐partitioning coefficients available. Most available empirical protein‐partitioning coefficients for PFAS are for bovine or artificial membranes (Lehmler et al. 2006; Xie et al. 2010; Bischel et al. 2011; Droge 2019).

The equilibrium partitioning–based approach used in bioaccumulation models has typically been applied to organochlorine chemicals that are neutral, hydrophobic, nonvolatile, and slowly metabolized (Kelly et al. 2004). Bioaccumulation would thus be high in the absence of depuration via metabolism, urine excretion, or respiration. The rate of depuration of certain PFAS in fish (i.e., 3–35 d) could be considered slow in comparison with other surfactants (e.g., linear alkylbenzene sulfonates, nonylphenols; Tolls et al. 1994; Martin et al. 2003a). However, the rate of depuration of PFAS in fish could be considered rapid compared with persistent organochlorine chemicals (e.g., PCBs, mirex, and chlorinated alkanes, which have half‐lives of 20–65 d; Fisk et al. 1998). This can be partly attributed to the lack of biotransformation or excretion of certain PFAS in fish (Martin et al. 2003a). For example, juvenile rainbow trout were exposed simultaneously to a homologous series of PFCAs and PFSAs for 34 d in the diet, followed by a 41‐d depuration period (Martin et al. 2003a). The assimilation efficiency from the diet ranged from 59 to 130% for all compounds detected. Carcass and liver BAFs were low (0.038–1.0). Precursors to some PFAS are metabolized in rodents and in wildlife. For example, Nabb et al. (2007) showed that 8:2 fluorotelomer alcohol can be metabolized to PFOA in Sprague–Dawley rats. Similarly, Letcher et al. (2014) showed in a liver in vitro microsomal assay that polar bears can rapidly de‐alkylate perfluorooctane sulfonamide to PFOS. Thus precursors could be entering food chains by partitioning into biota and then undergoing degradation to PFOS, PFOA, and other short‐chain PFCAs/PFSAs somewhere along the food chain. Therefore, the presence of precursors and their metabolic transformation products in wildlife can contribute to the overall bioaccumulation of PFOS, PFOA, and shorter chain PFCAs/PFSAs, which results in an increased probability for exposure and possible toxicity of these terminal end products.

Many equilibrium partitioning–based models assume that if uptake occurs by the same mechanism in “water‐breathing” organisms (e.g., fish and aquatic invertebrates) and air‐breathing organisms (e.g., terrestrial/marine mammals and birds), then similar uptake rates should be observed (Mackay and Fraser 2000; Kelly et al. 2004). For classical nonionic organic pollutants, this allows for the use of fish BCF or BAF data to estimate bioaccumulation in air‐breathing organisms. However, for some PFAS, studies show that food‐web bioaccumulation seems to occur to a greater extent in air‐breathing organisms (e.g., polar bears, dolphins, seals, Arctic birds) than in aquatic organisms (Environment Canada 2006, 2012a). Thus, for PFAS, fish bioaccumulation data cannot be used alone to reliably predict bioaccumulation in air‐breathing organisms. Several hypotheses have been advanced to try to explain differences in PFAS accumulation between air‐breathing organisms versus organisms that respire via the water. For example, fish gills provide an additional mode of elimination that birds and terrestrial and marine mammals do not possess. The fish gills may result in a more rapid elimination of PFAS to the water phase (via gill exchange) and an associated reduction in their bioaccumulation. Another possibility is that bioaccumulation in air‐breathing organisms is driven primarily by volatility rather than polarity. For nonvolatile PFAS, this allows for a relatively slow elimination to air, resulting in higher bioaccumulation in air‐breathing organisms (Kelly et al. 2004). Finally, the variability in biological persistence (and, hence, apparent bioaccumulation) between different species may be partially related to body size, with larger air‐breathing organisms having slower rates of depuration. Perfluoroalkyl substances also display substantial interspecies variability in tissue distribution and clearance rates, as well as sex‐specific differences in elimination rates (Lee and Schultz 2010; Zhang et al. 2012b; Ng and Hungerbühler 2013; Abercrombie et al. 2019).

Two fish bioaccumulation models have been developed that account for some of the physicochemical characteristics of PFAS. One is the BIOconcentration model for Ionogenic Organic Compounds (BIONIC) model (from Armitage et al. 2012, 2013), and the other is a protein‐partitioning bioaccumulation model from Ng and Hungerbühler (2013, 2014). Due to data availability, these models were built on training sets limited to PFCAs with carbon chain lengths greater than 7. Both models focused on predictions in freshwater fish. Given the lack of protein‐partitioning values for fish, the protein‐partitioning component of the bioaccumulation model (i.e., Ng and Hungerbühler 2013, 2014) used rat and human protein‐partitioning values. The BIONIC model (Armitage et al. 2012, 2013) considers phospholipids, rather than proteins, as the primary repository for PFAS, but the model does recognize the ionization potential of these substances. However, because PFAS bind primarily to fatty acid–binding proteins and lipoproteins/albumin, and then are sequestered in protein‐rich tissues, these proteins are important to consider. The bioconcentration predictions from the BIONIC model (Armitage et al. 2012, 2013) were best in liver and agreed with available empirical bioaccumulation data for freshwater fish for PFCAs with a carbon chain length greater than 7. The predictions from the protein‐partitioning model (Ng and Hungerbühler 2013, 2014) were best in predicting blood concentrations and agreed with available empirical BCF/BAF data for freshwater fish for PFCAs with a carbon chain length greater than 7. However, the protein‐partitioning model underestimated the bioaccumulation of PFHxS (6 carbons) and generally underestimated whole‐body bioaccumulation. Thus, it is possible that the active clearance and reabsorption processes described in the protein‐partitioning model do not operate in the same way or to the same extent in fish as in rats or humans. None of these models offer predictions for short‐chain (less than 7 carbon) PFAS, but available empirical data from Martin et al. (2003a, 2003b), used in both models, showed that short‐chain PFCAs (i.e., PFHxA, PFHpA) did not bioconcentrate in any rainbow trout tissue. However, Boisvert et al. (2019) showed that PFHxS, PFBA, and PFHxA had food‐web BAFs greater than 1 in polar bears. In freshwater riverine food webs, TMFs were greater than 1 for PFHxA (values of 0.36–3.7), perfluoroheptane sulfonate (values of 0.65–8.3), and perfluorodecane sulfonate (values of 0.73–17.9; Simmonet‐Laprade et al. 2019). In addition, PFHxS had food‐web BAFs greater than 1 in other air‐breathing organisms (i.e., dolphins, seals, and birds; Kannan et al. 2005; Houde et al. 2006a, 2006b; Haukås et al. 2007; Van den Heuvel‐Greve et al. 2009), despite the low measured bioaccumulation values (BCF/BAF less than 2000) for water‐breathing organisms (Martin et al. 2003a, 2003b; Jeon et al. 2010a, 2010b; Naile et al. 2013). These results suggest that the preferential food‐web bioaccumulation of PFAS in air‐breathing organisms can result in higher tissue concentrations compared with water‐breathing organisms. It is, therefore, worthwhile to consider future models that factor in both protein‐ and lipid‐partitioning and can offer food‐web bioaccumulation predictions for air‐breathing organisms.

In summary, there are significant information needs to support development of assays and models to better assess and predict the bioaccumulation in freshwater and marine species of PFAS from various structural groups. Data on the binding of different classes of PFAS to proteins in representative taxa of concern, and knowledge concerning PFAS metabolism in both invertebrates and vertebrates, are critical to improving modeling and prediction. Empirical data on the uptake, tissue distribution, and excretion of a range of PFAS in organisms other than those frequently tested (i.e., fish) are needed for model development and validation. These types of information will support improvement of existing bioaccumulation models for fish, as well as the development of models for other taxa of concern relative to PFAS bioaccumulation. Finally, considerable data indicate that some PFAS are bioaccumulative and readily transported within food webs, accumulating to greater concentrations at higher trophic levels. Thus BMF and TMF values may be the most relevant basis currently available for determining the overall bioaccumulation potential of some PFAS at upper levels of the food chain, as opposed to using BCF and BAF values derived from fish. In the absence of predictive bioaccumulation models, empirically derived BMFs and particularly TMFs could currently provide acceptably robust predictions of overall potential for food chain transfer of PFAS.

## NEEDS AND CONSIDERATIONS FOR TOXICITY TESTING TO SUPPORT HAZARD ASSESSMENT

### Identifying the scope of needed testing

The relative lack of toxicological data for PFAS as a broad class of compounds is a significant uncertainty facing ecological risk assessors. This shortcoming is manifested both in terms of the number/type of species for which there are toxicity data and the comparatively small number of PFAS that have been tested. As detailed in the previous section *Current Knowledge about Ecological Effects of PFAS*, there are extensive toxicity data for PFOS and PFOA and more limited information for in vivo effects of several other PFAS, but nothing is known concerning the potential toxicity of most PFAS. Consequently, there needs to be an organized effort to collect additional in vivo effects data. It is unrealistic from a resource or timing perspective to expect that empirical data can be collected for all PFAS present in or potentially entering the environment, but this rote‐type of testing approach is not necessary. Instead, a more strategic, systematic effort to address key data gaps should be employed. This would logically start with the step undertaken in the *Current Knowledge about Ecological Effects of PFAS* section—assembly and evaluation of what is already known concerning the effects of different PFAS in ecologically relevant species. This type of curation effort needs to be both publicly available and ongoing as new data are generated and published. The ECOTOX Knowledgebase utilized for our analysis is one, but certainly not the only resource suitable for the needed data extraction, assembly, and communication. Furthermore, the utility of the curated data in terms of guiding needed testing would be enhanced by a systematic review of the quality of existing information. In terms of new in vivo testing, the tenets we described previously in *Prioritizing PFAS for Monitoring and Testing* should be used as a basis to prioritize specific PFAS (and PFAS classes) for testing. That is, an approach toward a testing focus based on considerations such as production volume, use patterns, potential for persistence and bioaccumulation, and anticipated biological activity is essential. This type of prioritization ideally would be informed largely by existing knowledge and models but would also be augmented by inexpensive/rapid measures of variables like persistence and biological activity (e.g., using the types of methods we describe in *New Approach Method Applications in PFAS Ecological Risk Assessment* and *Opportunities for Application of NAMs to Ecological Hazard Assessments for PFAS*). An additional factor worthy of consideration in terms of prioritizing PFAS for more focused in vivo characterization could be based on structural classification, that is, most testing to date has been limited from a structural perspective (primarily PFCAs and PFSAs). Even a minimal amount of data for PFAS structural classes that have received little or no attention in terms of in vivo testing could be extremely valuable in helping to reduce risk assessment uncertainties. Consequently, one possible pragmatic step that could be taken—even in the context of limited resources—would be the identification of a suite of “model” PFAS representative of different classes that could serve as the basis for generation of baseline toxicity data for ecologically relevant species.

Consideration also should be given to the limited taxonomic representation of organisms that have been used thus far for PFAS testing. The goal of ecological risk assessment is, of course, not to protect just a single or few species, but entire assemblages of organisms that comprise exposed communities and ecosystems. To achieve this, there needs to be confidence that whatever test(s) employed for a given risk assessment scenario include potentially sensitive species (and endpoints). This is a problematic supposition relative to PFAS because even those that have been best studied (PFOS, PFOA) have been evaluated in comparatively few—largely aquatic—phyla, predominantly fish, crustaceans, and insects (see the previous section *Current Knowledge about Ecological Effects of PFAS*). There is far less toxicological information for PFOS or PFOA in other classes of vertebrates (mammals other than rodents, amphibians, birds, reptiles), many aquatic invertebrate phyla (e.g., mussels, annelids, cnidarians, etc.), and most terrestrial invertebrates. Even less is known concerning the toxicity of these comparatively well‐characterized PFAS to aquatic or terrestrial plants and microbial communities, in terms of either structure or function. Focused comparative testing with PFOS and PFOA in a phylogenetically broader suite of organisms would provide important baseline data. Although it is not feasible from a resource perspective to advocate routine testing of numerous species representing diverse phyla, it is possible to envision strategic selection of a few under‐represented taxa for testing both with PFOS/PFOA and with other less well‐characterized, priority PFAS. Selection of additional taxa for this testing should be informed by several criteria, including 1) potential for environmental exposure to PFAS; 2) evidence of possible impacts in field settings; 3) ecological importance in the context of ecosystem services; and 4) possession of biological pathways uniquely susceptible to different PFAS. For example, it is possible that some terrestrial plants, for which little is known concerning PFAS effects, could meet several of these criteria. Of course, another critical consideration in the selection of additional taxa for enhanced testing involves the existence of (or potential to readily develop) methods and/or feasibility for controlled testing. For example, although there is concern for the possible effects of PFAS in some cetaceans (see the *Mammalian wildlife* section), it is improbable that direct in vivo testing of species in this order would ever be possible.

Expanding the universe of species for which toxicity data exist is certainly important, but in doing so, it is critical to consider test designs relative to duration and endpoints. Again, using PFOS and PFOA as illustrations of existing knowledge, the great majority of toxicity data have come from short‐term tests focused on lethality. Although acute tests are important in terms of providing baseline data, they are not necessarily well suited to examining potential long‐term effects of persistent and bioaccumulative substances like some PFAS. Furthermore, tests focused solely on lethality would not detect responses associated with perturbation of some of the biological pathways of concern for some PFAS, such as effects on lipid metabolism, thyroid hormone signaling, or immunocompetence, for example (Lee et al. 2019). We are not suggesting that all in vivo testing for PFAS needs to involve chronic tests with sublethal endpoints—this clearly is impossible. However, there needs to be more chronic data than are currently available. There are approaches through which it may be possible to effectively identify those PFAS that might require chronic test data. For example, PFAS that have been shown or predicted to bioaccumulate would logically be candidates for chronic as well as acute toxicity assessments. There also is the potential to use in vitro data to identify PFAS that could be targeted for chronic testing. Specifically, as described in the *New Approach Method Applications in PFAS Ecological Risk Assessment* section, HTT assays are currently being employed to screen large numbers of different PFAS for pathway‐specific bioactivity. Information from these types of assays can highlight PFAS with the potential to impact pathways whose perturbation could cause developmental or reproductive effects as opposed to more nonspecific impacts that would result, for example, in lethality due to narcosis (Fay et al. 2018).

Finally, in considering the multidimensional nature of specific PFAS, taxa, and endpoints that theoretically could be emphasized in future in vivo assays, it is important to do so with the realization that resources for toxicity testing in the field of regulatory ecotoxicology are unlikely to increase. Furthermore, the desire to decrease the use of vertebrates for any type of testing will continue. Consequently, any testing should be focused and prioritized to the extent possible using existing knowledge and models. Furthermore, testing that is conducted should be done in a manner that maximizes both the quality and amount/type of information obtained from different assays. Considerations relative to generating high‐quality information from PFAS testing are covered in detail in the following section, *Limitations in PFAS ecotoxicity testing to date*. In terms of the type(s) of data collected, there should be an emphasis when possible not just on the apical endpoints needed for risk assessment/decision‐making (i.e., survival, growth, reproduction), but on pathway‐based biochemical, physiological, and histological responses indicative of the chemical's mechanism of action. For example, data from toxicity tests that can be used to support the validation and/or development of AOPs (including key events at the molecular and cellular levels) provide a critical basis for extrapolation of effects across chemical structures and species.

### Limitations in PFAS ecotoxicity testing to date

Conducting quality ecological risk assessments and developing environmental quality guidelines for PFAS requires access to high‐quality toxicity data. Although standardized toxicity test and data reporting protocols exist for a variety of ecologically relevant taxa in many jurisdictions worldwide, this does not always guarantee that toxicological research and testing conducted to date meet the basic requirements of quality toxicity data (Hanson et al. 2017).

A critical criterion for the generation of high‐quality toxicity data is analytical verification of the identity and concentration/dose of chemicals being tested. Without this exposure characterization, the quality of study results is diminished (Hanson et al. 2017). Exposure verification of PFAS may be especially problematic for several reasons: 1) there are few laboratories qualified/certified to perform PFAS analyses, and contracted analyses can be comparatively expensive; 2) there is a lack of widely accepted, validated methods for the analysis of many PFAS in a variety of matrices (e.g., no standards, matrix interference, inadequate accuracy and precision); and 3) exposure media are inadequately characterized; chemical confirmation may be limited to stocks and not exposure media.

To assess how the aquatic toxicology community has been performing relative to conducting PFAS tests, a set of approximately 20 published studies from the open literature was assessed relative to test conditions and data reporting (Supplemental Data, Table SI‐5). This analysis certainly does not purport to cover all tests that have been conducted with PFAS; instead, our goal was to illustrate some of the more pervasive data quality issues that have occurred in past testing. In the studies examined, test concentrations of PFAS in water were not consistently reported. Occasionally this was due to a lack of authentic standards and analytical methods, but more often, PFAS measurements were simply not conducted. This is not an uncommon problem in the field of ecotoxicology, but this type of omission would render data from these studies either useless, or would result in a very low data quality ranking, for example, in employing the study data for risk assessment or criteria derivation (US Environmental Protection Agency 1985). Of the studies listed in the Supplemental Data, Table SI‐5, that measured PFAS, many reported the occurrence of detectable PFAS in the control or dilution water. This is also a major data quality problem, but perhaps not surprising given that some PFAS are ubiquitous contaminants and are often found in water sources used by testing laboratories. In this context, it is conceivable that many laboratory waters emanating from surface water could contain detectable PFAS, such that it may be necessary to treat control/dilution water prior to testing. It is also possible to contaminate samples, especially control and/or very low‐level treatments through poor laboratory housekeeping; this is particularly an issue in laboratories not well versed in proper handling and disposal techniques for PFAS.

In addition to a relatively frequent lack of verification of PFAS concentrations, other shortcomings were commonly observed in the studies reviewed in the Supplemental Data, Table SI‐5. Overall, PFOS and PFOA were the most common test materials, but several other PFAS were also evaluated. All tests reported the purity of the test compound (generally greater than 98%), but quite often CAS numbers were not provided, which could be problematic for studies with chemicals like PFAS with variable composition and naming conventions. The studies evaluated also varied greatly relative to their use of carrier solvents for the test materials, with approximately 50% of them employing a solvent (e.g., dimethyl sulfoxide, triethylene glycol). A solvent carrier was used in almost half of the of PFOS assays, even though PFOS test concentrations were often well below water solubility limits of the chemical. In general, it is inadvisable to utilize a carrier solvent unless the test material is comparatively insoluble in water, because solvents can result in tests being conducted at chemical concentrations above water solubility, which is problematic relative to translating results to the environment. Finally, a large percentage of the PFAS toxicity tests captured in the Supplemental Data, Table SI‐5 employed static‐renewal rather than flow‐through conditions, which is not an optimal design relative to ensuring consistent chemical delivery, especially in chronic tests. The static‐renewal approach is particularly problematic when coupled with lack of analytical verification of chemical concentrations in the test water. One challenge, of course, in employing the preferred flow‐through approaches is that a greater amount of PFAS waste can be generated, requiring extraction/treatment of test system water with materials such as carbon or anion exchange resins before discharge.

The reviewed tests were of variable quality relative to the use of thoroughly documented methods and reporting of key test conditions. Not employing standard protocols when conducting toxicity tests can be problematic if the derived data are to be used for risk assessment purposes. Most of the experiments were conducted in glass or plastic containers, but a small percentage of the studies did not report test system materials. Given uncertainties as to the potential sorption of different PFAS to test materials, this is critical information to report, although it is less of an issue when PFAS concentrations are measured in the test solutions. Test medium or water used in the reviewed studies was quite variable, ranging from various reconstituted waters to surface water to well water, which is not unusual in the field of aquatic toxicology. However, only a small group of the studies reported a full suite of measured basic water quality parameters such as dissolved oxygen, pH, water hardness and alkalinity, and dissolved organic carbon levels. Although most studies reported test temperature, a small number of studies provided none of the above test parameters, which renders data from these experiments much less useful for risk assessment purposes. An example of both the importance of measured test substance concentration and measured water quality parameters during PFAS testing can be found in Colombo et al. (2008). This study reported the results of a 90‐d rainbow trout early life stage test with ammonium perfluorooctanoate in which the authors were able to demonstrate that the ammonium counterion portion of the test substance potentially contributed to the observed effects based on measurements of pH and total ammonia.

### Practical recommendations for future PFAS testing

Regardless of the extent of the chemical universe defined as PFAS from any given regulatory perspective (see *Background and Introduction*), structures that may require testing are extremely diverse from a physicochemical perspective, varying greatly in properties such as water solubility, volatility, stability, and sorption to biotic and abiotic components of test systems. Furthermore, the availability of well‐defined, high‐purity PFAS for testing (or supporting analytical verification) is sometimes problematic. Although some researchers have written about the practical challenges that can be encountered when testing different types of PFAS (e.g., Rewerts et al. 2020), there is no single guidance document detailing all the problems that may be encountered and how to address these problems. Consequently, it is essential that laboratories testing these substances using either aquatic or terrestrial species adhere to recommended best practices in terms of test design, conduct, and data reporting. This is not to say that all tests must be done using standard tests and model species; this is neither practical, nor even desirable, when there are important uncertainties as to the most susceptible species and endpoints. Experimental design flexibility is critical to supporting research to provide insights in terms of better understanding the toxicity of different PFAS. That said, however, all tests should be conducted “in the spirit of GLP” (Good Laboratory Practices) relative to data collection and reporting, with careful attention given to addressing the shortcomings highlighted in the previous section, *Limitations in PFAS ecotoxicity testing to date*. This type of philosophy will help ensure that the data generated from PFAS toxicity tests are optimally useful for risk assessment and regulation.

Discussions at the meeting resulted in one especially notable recommendation relative to conducting PFAS tests. Specifically, there was a strong sentiment that both aquatic and terrestrial toxicity testing with PFAS should include determination of tissue residues (including in eggs when appropriate) of the test chemicals (and, when known, relevant metabolites) whenever possible, but especially in conjunction with chronic tests. Tissue residue determinations would not obviate the need for measurement of the test chemicals in the external testing environment (water, sediment, soil) but, rather, would be a complementary measure relating exposure to dose, thereby supporting derivation of critical body residues associated with effects (McCarty and Mackay 1993; Jarvinen and Ankley 1999). Measurement of tissue residues addresses several important challenges and uncertainties inherent in the testing of PFAS, including a way to help circumvent the effects of poorly defined interactions of PFAS with diverse physicochemical properties with different test media or assay system materials in the context of bioavailability and toxicokinetics. Furthermore, translation of toxicity data to field settings can be greatly simplified when a common dose metric can be employed, that is, residues associated with effects in the laboratory versus tissue residues measured in samples from the field. Expressing toxicity based on tissue residues can also enhance comparative analysis of the relative potency of structurally different PFAS, perhaps lending insights into to their toxic mode(s) of action. For example, Vogs et al. (2019) showed the utility of tissue residues in examining the toxicokinetics of 4 PFAS (PFOS, PFOA, PFHxS, and PFBA) in zebrafish embryos, observing a biphasic uptake pattern with slow uptake before hatching and faster uptake post hatch. More importantly, observed differences in toxic potency among the PFAAs were reduced by 3 orders of magnitude when internal effect concentrations were compared with concentrations measured in the test medium. Finally, expressing the effects of PFAS based on tissue concentration can enhance prediction of inherent cross‐species susceptibility to the chemicals because this can help eliminate consideration of species‐specific differences in adsorption, distribution, metabolism, and elimination.

## OPPORTUNITIES FOR APPLICATION OF NAMS TO ECOLOGICAL HAZARD ASSESSMENTS FOR PFAS

Highlighted throughout our review is the fact that currently there is insufficient knowledge to adequately assess the potential ecological effects of all PFAS that occur or could potentially enter the environment. Given the number of substances involved, the need to consider both single chemicals and mixtures, and the diversity of ecosystems/species that might be exposed and impacted, this knowledge cannot realistically be attained using conventional chemical‐by‐chemical testing approaches employing in vivo assays and apical responses. Although these types of tests are critical to providing foundational insights for in‐depth risk analysis, resource limitations preclude their sole use for assessing PFAS ecological hazards/risks. However, as described previously (in the *New Approach Method Applications in PFAS Ecological Risk Assessment* section), there are a variety of NAMs that have been—and could be further—employed for this purpose. Below we provide considerations concerning the use of these types of tools to address challenges faced in the ecological assessment of PFAS.

New/emerging tools and knowledgebases that can be employed in the systematic assessment of PFAS range from curated databases with different types of biological effects data, to short‐term in vitro and in vivo assays with mechanistic/pathway‐based endpoints, to computational models. However, for information from these varied sources to be effectively used for predictive assessments, there is a need to organize the material in a coherent manner that emphasizes causal linkages among and between responses at different biological levels of organization. Model‐based predictions or even direct in vitro or in vivo evidence that a given PFAS can cause molecular or biochemical changes is of limited direct utility to most types of risk assessments. The role of the AOP framework in helping address this challenge is apparent; however, this type of application is predicated on the existence of an AOP(s) relevant to an observed/predicted molecular or biochemical change (i.e., corresponding to early key events such as the MIE), which often is not the case. Although there is an ever increasing number of AOPs available (Society for the Advancement of Adverse Outcomes Pathways 2016), they only capture a fraction of the MIEs potentially relevant for a given chemical based, for instance, on HTT data (e.g., Fay et al. 2018), and do not adequately consider the “universe” of taxa/biological responses of potential concern from an ecological effects perspective. Consequently, expanding AOP coverage in terms of pathways, species, and responses relevant to PFAS would be a logical research priority. For example, based on existing and emerging knowledge concerning molecular/biochemical perturbations caused by different PFAS, pathways/apical outcomes associated with signaling controlled by estrogen, thyroid hormone, and PPARs would be worthy of consideration in terms of AOP development. These also were identified by Lee et al. (2019) as potentially important targets of PFAS in nonmammalian vertebrates.

Analogous to the AOP situation, many of the available computational and in vitro NAMs—and the resultant data generated—are limited in terms of the taxonomic and pathway coverage needed to assess the ecological risks of PFAS. For example, most available QSARs, including those available for PFAS, are based on biological responses from mammalian systems (e.g., Cheng and Ng 2019). Similarly, many of the in vitro methods that have been/could be used to directly generate bioactivity data for PFAS utilize mammalian constructs (Villeneuve et al. 2019). Based on knowledge of conservation of MIEs/biological pathways from the extant comparative physiology literature and insights provided by bioinformatic tools such as the SeqAPASS, bioactivity extrapolations from mammalian systems to other vertebrates in many instances may be technically reasonable (e.g., Ankley et al. 2016; LaLone et al. 2018), although there may be a need to account for cross‐species differences in the metabolism of PFAS (e.g., Nabb et al. 2007; Letcher et al. 2014). However, extrapolation uncertainties to other ecologically important taxa (invertebrates, plants, microbes) remain substantial. Therefore, a pressing need exists for computational models and in vitro assays based on nonmammalian taxa that can be used for the rapid assessment of PFAS.

Short‐term in vivo assays with pathway‐based responses, including transcriptomic, proteomic, and metabolomic measurements, are further advanced in terms of ecological coverage/application than computational and in vitro NAMs. Recent progress from both technical and conceptual perspectives has been promising in terms of increasing taxonomic representation and types of environmental stressors that can be addressed with these omic‐based assays (Leung 2018; Martyniuk 2018; Song et al. 2018; Zhang et al. 2018; Gouveia et al. 2019; Schuttler et al. 2019). Although there have been some short‐term in vivo/toxicogenomic studies with different PFAS in nonmammalian vertebrates (e.g., Huang et al. 2012; Dusgupta et al. 2019; Lee et al. 2019; Khazaee et al. 2020), this is an area where work with additional PFAS and species would be warranted to help identify biological perturbations of potential concern.

Given the current state‐of‐the‐science, most of the NAMs we have described are not yet appropriate for generating effects data on which to base quantitative ecological risk assessments for PFAS. Although there have been promising developments relative to the derivation of quantitative AOPs potentially suitable for employing biochemical/molecular data in formal risk assessment (e.g., Conolly et al. 2017; Wittwehr et al. 2017), it would be premature to do so for most PFAS, whose biological activities are often poorly understood. However, there are several potential applications for NAMs relative to different facets of assessing the ecological effects of PFAS, including prioritization of untested chemicals for more in‐depth consideration based on bioactivity profiles. Specifically, it may be possible to use NAM data to help categorize different PFAS based on bioactivity “fingerprints,” ranging from those that have comparatively little activity to those that seem capable of impacting multiple biological targets.

Data from NAMs can also be useful for guiding higher tier testing of PFAS. Specifically, when observed bioactivities/targets can be linked to known pathways/processes, for example using AOPs, insights as to susceptible species and sensitive endpoints can be gained. As a simple illustration of this, Stinckens et al. (2018) reported that PFOA caused significant inhibition of the activity of deiodinases involved in thyroid hormone synthesis using in vitro assays with liver tissue from pigs. Computational analyses by LaLone et al. (2018) indicated a high degree of structural conservation in deiodinases across vertebrates, suggesting that the enzymes might also be inhibited by PFOA in nonmammalian vertebrates. In querying the AOP wiki, there are 2 AOPs relevant to ecological effects that have a MIE of deiodinase inhibition, one involving delayed metamorphosis in amphibians (AOP#190; Society for the Advancement of Adverse Outcomes Pathways 2016) and the second, impacted swim bladder development in fish (AOP#155; Society for the Advancement of Adverse Outcomes Pathways 2016). This information serves as a foundation for hypothesis‐based testing and/or assay development. In the case of the observed deiodinase inhibition in a mammalian in vitro assay, follow‐up in vivo studies with zebrafish have documented that PFOA does indeed affect swim bladder development (Stinckens et al. 2018), as seemingly do other PFAS that affect thyroid hormone signaling (Wang et al. 2020).

There are additional points worthy of note relative to the use of NAMs to help address knowledge gaps and uncertainties associated with PFAS assessments. First, many of the basic techniques/concepts (e.g., HTT in vitro assays; toxicogenomic measurements coupled to short‐term in vivo studies; the AOP framework, etc.) have existed for a decade or longer. However, there has been hesitancy in many instances to employ these newer approaches for regulatory decision‐making. There are multiple factors contributing to this limited acceptance, but one that is particularly important involves a lack of interaction between researchers developing and researchers (potentially) using different emerging technologies (Carusi et al. 2018; Leung 2018; Martyniuk 2018). This highlights the need for close collaborations between scientific experts in the development of relevant NAMs and risk assessors/regulators involved in assessing PFAS, perhaps in the context of focused case studies/applications. This type of interaction has proved fruitful in the conceptual and practical application of NAMs to the evaluation of potential human health and ecological effects of endocrine‐disrupting chemicals (e.g., Browne et al. 2017; Noyes et al. 2019; Knapen et al. 2020), a regulatory effort that faces many challenges in common with PFAS (i.e., a large number of highly diverse, untested chemicals).

A final observation relative to employing NAMs to assess PFAS involves human health–ecological connections. Specifically, most NAMs used for effects assessment focus on predictions or measurements in early portions of biological pathways, which, in many instances, can be highly conserved across species. Some pathways/processes are common from microbes to humans (e.g., aspects of energy metabolism), whereas others are more specific to a given taxonomic group (e.g., lactation in mammals). Given our continually evolving knowledge concerning conservation of key components of biological pathways, the separation between traditional test models for human health versus ecological toxicology (e.g., rodents vs fish) becomes much less important when there is an emphasis on initial biological perturbations. Better integration of human health and ecological toxicology based on selection of models appropriate for assessing biological pathways of concern rather than an automatic default to traditional test species/assays is not a new concept (for reviews, see Perkins et al. 2013; Rivetti et al. 2020). However, this becomes a particularly salient consideration when one is faced with limited resources for assessment of a chemical universe as broad as that of PFAS.

## ADDRESSING THE CHALLENGE OF PFAS MIXTURES

One of the greatest uncertainties in ecological risk assessment is predicting the effects of chemical mixtures. Like most environmental contaminants, PFAS primarily occur as mixtures in the environment. For example, monitoring programs focused on surface water and/or tissues have commonly reported the occurrence of detectable concentrations of a few to a dozen or more discrete PFAS from some aquatic environments (New Jersey Department of Environmental Protection 2018; Liu et al. 2019; Munoz et al. 2019). A somewhat unique aspect of the PFAS mixture challenge relative to many other aquatic contaminants is that PFAS are often introduced to the environment as end‐use formulations comprised of complex mixtures. This highlights the need to understand potential PFAS mixture effects not only in a retrospective context (i.e., chemicals already present in the environment), but from the standpoint of prospective assessments (i.e., prediction of the toxicity of new chemicals that may be released to the environment). For example multiple AFFF formulations, which have been used extensively for decades and still serve as an important source of PFAS in the environment, were commonly comprised of several PFAS including, primarily, PFOS, PFHxS, and sometimes the 6:2 fluorotelomer sulfonate (Backe et al. 2013; Hu et al. 2016; Barzen‐Hanson et al. 2017; Dubocq et al. 2019). The exact formulation, however, can vary through time and by manufacturer of a given brand of AFFF. Furthermore, these variations can be either intended or occur as a byproduct of evolving manufacturing processes. Once applied for fire suppression training or emergency response, PFAS from AFFF continue to present a mixture challenge, but one in which the exact chemicals and their proportions typically differ from what was applied due to interactions with environmental media (Hu et al. 2016; Barzen‐Hanson et al. 2017; Hatton et al. 2018; Munoz et al. 2019). Another concentration point of PFAS in the environment can be wastewater treatment effluents and application of contaminated biosolids as soil amendments in agricultural fields. Here PFAS inputs vary as a function of waste source/stream(s) and treatment systems; the resulting contaminated environments again often contain a combination of many different PFAS and co‐occuring contaminants (Exner and Färber 2006; Lindstrom et al. 2011; Houtz et al. 2016; Hu et al. 2016; Dauchy et al. 2017). Given the nature of PFAS production and use, it is inevitable that ecological receptors are—or will be—exposed to PFAS mixtures. Most toxicity data, however, have been generated based on single PFAS chemicals or constructed mixtures that may or may not reflect those present in the environment. A key challenge for ecological risk assessment of PFAS therefore lies in improving our understanding of both exposures to PFAS mixtures and the effects of these mixtures.

To address this challenge, it is necessary to characterize and prioritize PFAS mixtures (and their individual components) based on relevance to ecological receptors in the context of both exposure and effects. Ideally, if we were convinced that a few PFAS chemicals and mixtures were most relevant to exposure and/or effects, this would simplify ecological risk assessments for the chemicals. Knowing which PFAS are most likely to occur at the highest concentrations and with greatest frequency, combined with information as to which PFAS are potentially most biologically active and potent would allow us to highlight those chemicals requiring further testing and assessment. Criteria on which to base this type of prioritization are discussed in the *Prioritizing PFAS for Monitoring and Testing* section. However, an important uncertainty in discerning potential mixture effects is that analytical methods and/or standards are not available for all PFAS, so critically important PFAS that could have relatively greater toxicity or high environmental concentrations may be missed. In considering these types of uncertainties, below we suggest several tools and concepts potentially useful for addressing PFAS mixtures in ecological risk assessments.

A predictive approach toward PFAS mixture characterization should include not just analytical, but toxicological assessment of PFAS formulations (also referred to as whole mixture testing), such as those used for industrial and fire suppression applications. For example, Bursian et al. (2020) exposed juvenile Japanese quail to PFOS, PFOA, PFOS + PFOA, and 2 AFFF formulations in feed for 5 d during a subacute study. They found rather consistent effects on juvenile mortality for PFOS, PFOA, and the PFOS + PFOA mixture. However, the toxicity of the 2 AFFF formulations differed in that one formulation did not cause any toxicity whereas the second AFFF was less toxic than PFOS, the dominant PFAS in the mixture. Similar approaches are now being utilized by researchers and risk assessors associated with the US Department of Defense to screen potential replacement fire‐fighting foams. Although not necessarily reflective of the PFAS mixture ecological receptors ultimately might be exposed to, this type of biological screening approach could aid in identifying active PFAS or PFAS combinations that warrant attention (e.g., more in‐depth testing) and may be cause for concern if released into the environment. Alternatively, retrospective analyses would entail analyzing existing environmental samples to determine which PFAS ecological receptors would likely be exposed to (East et al. 2020) and could form the basis of effects and exposure assessments for estimates of ecological risk (e.g., Larson et al. 2018; Salice et al. 2018). For example, identification of the most common PFAS mixtures in surface water samples from US Air Force Bases (East et al. 2020) was recently used to inform PFAS mixtures for testing in bobwhite quail (Dennis et al. 2020).

A key factor in both prospective and retrospective analyses lies in whether analytical methods can adequately capture the spectrum of PFAS likely to occur in the environment and/or be available to cause a biological response. Several validated analytical methods have been in use and are continuing to be refined for quantifying some of the many thousands of potential PFAS in commerce or in the environment (Moody et al. 2001; US Environmental Protection Agency 2020c). However, the target analyte list of many of these methods is limited. In other instances, methods validated for PFAS analytes in one type of sample (e.g., water) have not been validated in another (e.g., tissues). For example, ongoing PFAS monitoring at US Air Force bases where AFFF was used only include up to 18 PFAS (and often considerably fewer), with an emphasis on water and sediment samples (Anderson et al. 2016). Another analytical and assessment challenge is the occurrence of PFAS precursors that can be biotically and/or abiotically transformed to “end use” PFAS (Ellis et al. 2004; Harding‐Marjanovic et al. 2015). Precursors occur in formulations and in the environment, but targeted analytical methods to identify specific precursors are often limited in large part due to a lack of standards (Newton et al. 2017; Dubocq et al. 2019). In any case, both unidentified/unmeasured PFAS and PFAS precursors currently are a challenge for mixture assessment because they contribute uncertainty to our understanding of which PFAS will ultimately interact with ecological receptors.

Researchers have attempted to measure the total quantity of organofluorine compounds as a means of characterizing PFAS mixtures in the absence of the ability to quantify each constituent PFAS. These approaches vary greatly in terms of sensitivity, selectivity, and applicability to different sample matrices (McDonough et al. 2019). For example, methods to measure total organic fluorine have been developed based on combustion and conversion of organic and/or inorganic fluorine compounds in aqueous solution to hydrogen fluoride and then measured as total fluorine by ion chromatography (Miyake et al. 2007; Wagner et al. 2013). Particle‐induced gamma ray emission spectroscopy has also been used to measure total fluorine from surfaces (Ritter et al. 2017). These methods are rapid screening techniques but do not allow identification of specific PFAS or PFAS classes unless sample preparation strategies and fractionation are implemented to separate organofluorine fractions with different chemical characteristics. In addition, the sensitivity of these total fluorine liberation/measurement methods is generally too low for most environmental samples, limiting their application to heavily contaminated samples (McDonough et al. 2019). Another approach to detecting the presence of potentially unmeasured PFAS is the persulfate wet oxidation method, known as the total oxidizable precursor (TOP) assay (Houtz et al. 2016). The TOP assay is readily applicable to aqueous samples and is comparatively sensitive (ng/L), but it selects only precursor compounds that can be chemically oxidized to form target PFAAs. Furthermore, the products of chemical oxidation do not always correspond to the PFAAs produced by the environmental or in vivo degradation of precursors, and inclusivity is limited to chemically oxidized fluorinated precursors that can be analyzed by liquid chromatography methods. Selectivity is dictated by the choice of which oxidized products are monitored, so consequently precursors may be missed that are oxidized to unmonitored PFAS. In addition, low and variable recoveries may lead to false negatives, especially in samples that do not have high precursor concentrations (McDonough et al. 2019). Despite these limitations, the TOP assay combined with targeted analyses was successfully applied to sediment and biota samples to determine how potential PFAS mixtures vary within a trophic food web. The proportion of unidentified PFAA precursors decreased from sediments to invertebrates and fish, perhaps suggesting biotransformation of the precursors across trophic levels, which could contribute to the biomagnification of some PFAS (Simmonet‐Laprade et al. 2019).

The broad analytical techniques just described allow a crude estimate of the occurrence and identity of precursors and unidentified PFAS. However, it is critical to have robust identification of previously uncharacterized PFAS, which requires liquid chromatography linked to nontargeted high‐resolution mass spectrometry (LC‐HRMS; Dubocq et al. 2019; McDonough et al. 2019). For unequivocal and reliable PFAS identification and quantification, pure analytical standards must be available; currently, such standards are commercially available for less than 50 potentially relevant PFAS. In addition to identification of unknown PFAS, LC‐HRMS can recognize a series of PFAS homologs that differ only in the fluorinated chain length while sharing the same chemical functional group(s). In this case, the identification is more reliable and semiquantitative even without specific analytical standards (Strynar et al. 2015; Newton et al. 2017). If bioactivity‐based research shows that PFAS belonging to the same homolog series act via the same MIEs/AOPs, but differ only in potency, a toxicological or ecological risk characterization of the mixture of PFAS homologs based on the relative potency factor (RPF) approach described in the next paragraph may be viable (Organisation for Economic Co‐operation and Development 2018a).

Although it clearly is critical to identify those individual PFAS of greatest concern relative to exposure and/or effects, there remains the very real possibility that in some scenarios PFAS mixtures will cause adverse effects that are not captured by a single or few PFAS. Assessing the potential for this is, again, exacerbated by the fact that the full suite of relevant PFAS is not readily identifiable due to knowledge gaps in analytical chemistry and toxicology. However, even if these basic knowledge gaps relative to individual PFAS did not exist, there have been comparatively few empirical studies with defined PFAS (component) mixtures, so there is uncertainty as to appropriate models to employ for prediction of interactive effects. Many of the existing component mixture studies with PFAS have focused on PFOS and PFOA combined in laboratory exposures (Supplemental Data, Table SI‐6) or evaluated with other classes of toxicants. It currently is not feasible to reliably predict how different PFAS might interact with one another. Indeed, in a single study, synergism, additivity, and antagonism were all reported in zebrafish for different combinations/ratios of PFOS and PFOA and different endpoints (Ding et al. 2013). The challenges in understanding the combined effects of these most commonly studied PFAS highlights how difficult it may be to adequately predict effects of more complex PFAS mixtures. For mixtures of some organic chemicals, for example, PCBs, polychlorinated dibenzofurans, and polychlorinated dibenzo‐*p*‐dioxins, an RPF‐based approach has been used to estimate potential mixture effects based on a common MIE, activation of the aryl hydrocarbon receptor (Van den Berg et al. 1998). Currently, however, even for well‐studied PFAS like PFOS and PFOA, some MIEs relevant to the chemicals are established (e.g., PPARs), but not all the mechanisms via which these chemicals might exert toxicity are known (Lee et al. 2019). Identification of relevant MIEs for different PFAS using the types of pathway‐based tools described in *New Approach Method Applications in PFAS Ecological Risk Assessment* could provide a framework for categorization based on shared modes of action, which would enhance the development of predictive models for mixture effects. In the absence of knowledge concerning molecular targets/mechanisms of action for PFAS, other approaches to the development of models for predicting mixture effects might be useful; such approaches may involve activation of a suite of responses or a multivariate activation index that can be used to identify biological active PFAS and PFAS mixtures (Martin et al. 2010; Neale et al. 2015). However, an advantage to developing RPFs based on suites of shared MIEs/pathways for PFAS is that there is already a regulatory precedent, which would simplify application to PFAS ecological risk assessments.

Effects and risks of PFAS mixtures might also be effectively addressed through the use of linked biology–chemistry methods to first detect and then identify specific chemicals of concern. This basic conceptual approach in the field of regulatory ecotoxicology originated several decades ago in the effluent monitoring program in the United States, where it was realized that analytical chemistry alone was not sufficient to account for all chemicals in a complex mixture that might cause toxicity (for a brief review, see Ankley et al. 2011). This challenge essentially resulted in the incorporation of whole effluent testing/toxicity limits into discharge permits to complement targeted analytical monitoring. Of course, for this biological augmentation to be helpful, it was then necessary to be able to determine the identity of the undetected/unmonitored chemicals causing toxicity to implement mitigation options. This need has resulted in the development of a variety of sophisticated effects‐based fractionation approaches that combine biological detection of activity with instrumental identification of causative toxicants (e.g., Ankley et al. 2011). The key challenge for a linked biology–chemistry approach to assessment of complex PFAS mixtures involves identification of an appropriate model/system to “detect” biological response(s) of interest in complex mixtures of concern, and then “track” this activity through various fractionation steps focused on analytical identification of chemicals of concern. In the case of the complex effluent program, toxicity tests with apical endpoints (with fish or cladocerans) were used to detect and track toxicity. These types of apical assays could have a very useful role in terms of using effects‐based fractionation to identify components of greatest concern in PFAS end‐use formulations; however, they would not be as useful for detecting/tracking PFAS mixtures in environmental samples, because the biological system could be affected by any of a number of contaminants in a given sample from the environment. Detection of relevant bioactivity in environmental samples would require more pathway‐based biological responses that could potentially be linked to known/suspected PFAS modes of action. An example of such a system, and its application to environmental monitoring, was recently provided by Blackwell et al. (2018). In that study, the in vitro HTP Attagene system described in the *New Approach Method Applications in PFAS Ecological Risk Assessment* section was used to evaluate pathway‐based bioactivities of 38 surface water samples from around the United States, which had also been subjected to extensive instrumental analytical characterization. Blackwell et al. (2018) were able to demonstrate that some of the pathways stimulated in the Attagene system could be explained by contaminants measured in the samples (e.g., estrogen receptor activation), whereas others could not (e.g., PPAR activation), indicating the presence of unmeasured chemicals of potential concern. As knowledge is gained concerning biological processes/MIEs affected by PFAS, both in vitro systems (like Attagene or more targeted assays with specific receptors relevant to pathways affected by PFAS; e.g., Escher et al. 2013) and pathway‐based in vivo measurements (including omic responses) should prove useful for detecting bioactivities associated with PFAS in complex environmental samples, as well as providing relevant biological systems for tracking activity in effects‐based fractionation analyses focused on the identification of causative toxicants.

## CONCLUSIONS AND RECOMMENDATIONS

Assessing the ecological risks of PFAS is a multifaceted challenge requiring contributions of experts from many disciplines in the field of environmental toxicology and chemistry. Although there are jurisdictional differences as to how many and which PFAS are of concern, the universe of substances that may need to be assessed is substantial and is comprised of chemicals with widely varying physicochemical properties that can differ greatly relative to fate, exposure, and effects. Furthermore, little or no empirical data exist for the majority of PFAS of known or potential concern, requiring innovative approaches to prioritizing and evaluating those requiring risk assessment in either prospective or retrospective settings. In the present review, we sought to 1) capture what is currently known about PFAS relative to potential ecological risk, 2) identify what is not known but is needed for effective assessments, and 3) propose paths forward in terms of addressing these needs. Below is a brief summary of conclusions and recommendations from this analysis.

Widespread concern about the possible ecological impacts of PFAS is comparatively recent, so many regulatory and risk assessment programs are still in early stages of development and implementation (*Select PFAS Ecological Risk Assessment Activities from around the World* section). However, unless a given effort is focused on just a limited number of compounds (e.g., PFOS, PFOA), there is a clear need to prioritize those PFAS of greatest concern for monitoring and testing. Although different regulatory authorities and programs have different mandates in terms of targeted chemicals (e.g., materials already in the environment vs those that might be released), the core considerations provided in the *Prioritizing PFAS for Monitoring and Testing* section provide a foundation for knowledge‐driven prioritization based on potential for exposure and effects: 1) production process, and product volume and use, 2) physicochemical properties, 3) degradation, persistence, and occurrence, and 4) bioactivity. The concept of essentiality (of use) could also be a component of prioritization, although considerations here are largely societal and socioeconomic in nature. Different types of data/models can be used to provide knowledge to address these prioritization criteria, but there remains the need for a conceptual framework to integrate the knowledge. Such a framework would require options for decision‐making when information concerning attributes of a given PFAS is limited or unavailable. Development of a technically robust prioritization framework would be a worthwhile undertaking for scientists and risk managers responsible for PFAS assessments and, ideally, would be conducted in an internationally harmonized manner.

As summarized in the *Current Knowledge about Ecological Exposures to PFAS* section and the associated Supplemental Data, there are many studies reporting the occurrence of PFAS in abiotic and biotic components in the environment. Although this information can provide the basis for exposure characterization of chemicals like PFOS and PFOA, existing data are generally limited with respect to the number of other PFAS that have been assessed, and the range of environments in which they have been measured. There is a need for long‐term monitoring programs focused on a greater number of PFAS, designed in a manner such that the collected data can support probabilistic assessments of risk in different environments (see *Needs and Considerations for Advancing Exposure Assessment*). Monitoring efforts should consider not just parent chemicals, but precursors, degradates, and metabolites. Accomplishing this effectively would require use of both targeted and nontargeted analytical techniques for monitoring. Data from PFAS monitoring efforts would be particularly valuable if they could be collected and curated in open source data repositories.

An important uncertainty in exposure assessments for PFAS involves their potential to bioaccumulate. This has significant repercussions for both human health and ecological assessments. Although there is a reasonable amount of bioaccumulation data for PFOS and PFOA for both aquatic and terrestrial life, there is little to none for the broader spectrum of PFAS. Unfortunately, the types of lipid‐based equilibrium models used to successfully predict the bioaccumulation of neutral organic chemicals like PCBs are not suitable for the majority of PFAS. Consequently, there is a pressing need for the development of structure‐based predictive bioaccumulation models for PFAS. To achieve this requires a better understanding of factors controlling PFAS bioaccumulation in different species, including protein binding and metabolism (see the *Needs and Considerations for Advancing Exposure Assessment* section). There is also a need to better understand the movement of PFAS through aquatic and terrestrial food webs, including predication of biomagnification.

Although a fair amount of ecotoxicity data exists for some PFAS, the overall scope of knowledge is limited relative to the number and structural categories of PFAS that have been tested and the taxa used for testing conducted to date (see the *Current Knowledge about Exposures to PFAS* section). Furthermore, much of the available toxicity data are for acute rather than chronic effects, which, for persistent bioaccumulative chemicals like some PFAS, is an important data gap. In addition, comparatively little is known concerning the toxicity of sediment‐associated PFAS to benthic organisms. Although it clearly is not feasible to collect empirical test data for all chemicals and species of potential concern, the field would benefit from additional in vivo toxicity testing with poorly characterized PFAS and potentially sensitive taxa. Implementation of this type of effort could be strategic in nature, employing chemical prioritization criteria, such as those described in the *Prioritizing PFAS for Monitoring and Testing* section, and information from computational models, in vitro assays and omic responses from short‐term in vivo tests to guide and focus additional testing (see the *Needs and Considerations for Advancing Exposure Assessment* and *Opportunities for Application of NAMs to Ecological Hazard Assessments for PFAS* sections).

Given the physicochemical diversity of PFAS and resultant range of properties (e.g., volatility, water solubility, sorption) that might affect their behavior in test systems, it is critical that effects testing routinely confirm exposures analytically (see the *Needs and Considerations for Toxicity Testing to Support Hazard Assessments* section). Although this is certainly an added expense, especially for PFAS for which analytical techniques and standards are often lacking, the absence of exposure verification results in data of limited value to regulatory risk assessment and decision‐making. A logical approach to addressing some of the challenges posed in terms of exposure characterization would be to more commonly measure tissue residues of PFAS (both parent and, when known, metabolites) in conjunction with routine testing, especially for chronic tests (see the *Needs and Considerations for Toxicity Testing to Support Hazard Assessments* section). Availability of tissue residue–effects data has the added benefit of reducing extrapolation uncertainty across species and chemicals, and from the laboratory to the field.

Although the challenge of potential mixture effects is common to virtually all environmental contaminants, it is particularly problematic for PFAS for several reasons, including: 1) PFAS both (often) enter and occur in the environment as mixtures, 2) there is a lack of knowledge concerning the identity of important degradates or metabolites, and 3) standard analytical methods for fully defining mixtures in formulations or environmental samples are often unavailable (see *Addressing the Challenge of PFAS Mixtures*). Accordingly, critical mixture‐oriented research for PFAS includes the further development of targeted and nontargeted analytical techniques capable of better characterizing components of mixtures in end‐use products, and in the environment as abiotic and biotic factors alter PFAS assemblages. In terms of predicting the biological effects of mixtures, there is a need for baseline empirical studies with formulations and component mixtures designed to mimic those in the environment with potentially susceptible species and endpoints. There also is a need to generate data that would support the use of models for predicting the toxicity of PFAS mixtures before they are used/released to the environment. Prominent here is pathway‐based work aimed at categorizing different PFAS based on biological similarity (common AOPs), which would enhance the use of RPF‐based approaches to forecast mixture toxicity, at least for a subset of well‐defined pathways/outcomes.

In conclusion, the core challenge posed in assessing the ecological risks of PFAS as a broad class of either discrete chemicals or mixtures involves a lack of information to adequately assess their potential fate, exposure, and effects. Although it is certainly possible to generate the empirical exposure and effects data needed to conduct robust ecological assessments for a handful of high‐priority PFAS, it is not reasonable to expect that extensive empirical data can or will be collected for the majority of PFAS precursors, parents, and metabolites/degradates of possible concern. Consequently, there is a need to consider resources such as curated knowledgebases, computational models, and cost‐effective/rapid measures of persistence and biological activity as a basis for predictive approaches to help support risk assessment efforts for PFAS. Although predictive pathway‐based methods such as those described in the *New Approach Method Applications in PFAS Ecological Risk Assessment* section have been and are currently being used to provide insights into PFAS hazards, these types of tools are currently best suited to supporting activities related to screening, prioritization, and designing testing strategies, as opposed to quantitative population‐level risk assessments (see *Opportunities for Application of NAMs to Ecological Hazard Assessments for PFAS*). However, it is possible to envision a tiered approach comprised of predictive tools integrated with higher level monitoring and testing that would provide a reasonable solution for dealing with large numbers of PFAS in a technically defensible, efficient manner. We believe that the needed research advances and tool development strategies we have described would provide the basis for developing and implementing such an approach.

## Supplemental Data

The Supplemental Data are available on the Wiley Online Library at https://doi.org/10.1002/etc.4869.

## Disclaimer

Positions or statements expressed herein are those of the authors and do not necessarily represent the views or policies of their organizations.

## Supporting information

This article includes online‐only Supplemental Data.

Supporting information.

Supporting information.

Supporting information.

Supporting information.

Supporting information.

Supporting information.

Supporting information.

## Data Availability

Data, associated metadata, and calculation tools are available from the corresponding author (ankley.gerald@epa.gov).
