# Supplementary material for: Assessing the Ecological Risks of Per‐ and Polyfluoroalkyl Substances: Current State‐of‐the Science and a Proposed Path Forward
Source: Environ Toxicol Chem. 2020 Nov 6;40(3):564–605. doi: 10.1002/etc.4869 (PMC7984443; doi:10.1002/etc.4869)
Supplement: Supplementary file 1 — Supporting information. [file ETC-40-564-s006.pdf]

Glossary

As a broad class of chemicals with thousands of compounds, the nomenclature of PFAS can be very confusing and uneven across the open literature. This glossary of acronyms represents an effort to define and standardize the acronyms used in the text of this manuscript. For PFAS that appear more than two times in the text of our article, an acronym has been used for the compound, following the nomenclature suggested by Buck et al. (2011). PFAS acronyms that appear in the extensive Supplementary Information (SI) tables have not been harmonized in the same manner. , so as is The SI represents a substantial synthesis of information that can guide the reader, but if specific compounds or concentrations are of interest, we suggest that data associated with PFAS listed in the tables be verified by the reader by consulting the original research articles.

(n:2) FTCAs - (n:2) Fluorotelomer (saturated) carboxylic acids ($C_nF_{2n+1}CH_2COOH$)

(n:2) FTOHs – (n:2) Fluorotelomer alcohols ($C_nF_{2n+1}CH_2CH_2OH$)

(n:2) FTUCAs - (n:2) Fluorotelomer unsaturated carboxylic acids ($C_{n-1}F_{2n-1}CF{=}CHCOOH$)

6:2 Cl-PFAES - 2-(6-chloro-1,1,2,2,3,3,4,4,5,5,6,6-dodecafluorohexyloxy)-1,1,2,2-tetrafluoroethanesulfonate, Potassium 2-(6-chloro-1,1,2,2,3,3,4,4,5,5,6,6-dodecafluorohexyloxy)-1,1,2,2-tetrafluoroethanesulfonic acid (CAS - 73606-19-6)

6:2 FTCA - 6:2 Fluorotelomer carboxylic acid ($C_6F_{13}CH_2COOH$; CAS - 53826-12-3)

6:2 FTOH - 6:2-Fluorotelomer alcohol ($C_6F_{13}CH_2CH_2OH$; CAS - 647-42-7)

8:2 FTOH - 8:2 Fluorotelomer alcohol ($C_8F_{17}CH_2CH_2OH$; CAS - 678-39-7)

AFFF – Aqueous film-forming foams

Cl-PFAES - Chlorinated-polyfluoroalkylether substances

FASAs - Perfluoroalkane sulfonamides ($C_nF_{2n+1}SO_2NH_2$)

FPPPs – Fluorinated plant protection products

PFAAs - Perfluoroalkyl acids - Includes perfluoroalkyl carboxylic, sulfonic, sulfinic, phosphonic, and phosphinic acids

PFAS – Perfluoroalkyl and polyfluoroalkyl substances

PFBA - Perfluoroalkyl carboxylic acids ($C_nF_{2n+1}COOH$) where n=3 and inclusive of all forms such as perfluorobutanoic acid (C_3F_7COOH; CAS - 375-22-4), ammonium perfluorobutanoate (NH_4^+ $C_3F_7COO^-$; CAS - 10495-86-0), sodium perfluorobutanoate (Na^+ $C_3F_7COO^-$; CAS - 2218-54-4)

PFBS - Perfluoroalkane sulfonic acids ($C_nF_{2n+1}SO_3H$) with n=4 and inclusive of all forms such as perfluorobutane sulfonic acid ($C_4F_9SO_3H$; CAS - 375-73-5 or 59933-66-3), perfluorobutane sulfonate anion ($C_4F_9SO_3^-$; CAS - 45187-15-3), potassium perfluorobutane sulfonate (K^+ $C_4F_9SO_3^-$; CAS - 29420-49-3)

PFCAs - Perfluoroalkyl carboxylic acids ($C_nF_{2n+1}COOH$)

PFDA – Perfluoroalkyl carboxylic acids ($C_nF_{2n+1}COOH$) where n=9 and inclusive of all forms such as perfluorodecanoate ($C_9F_{19}COO^-$; CAS - 73829-36-4), perfluorodecanoic acid ($C_9F_{19}COOH$; CAS - 335-76-2), ammonium perfluorodecanoate (NH_4^+ $C_9F_{19}COO^-$; CAS - 3108-42-7)

PFHpA - Perfluoroalkyl carboxylic acids ($C_nF_{2n+1}COOH$) where n=6 and inclusive of all forms such as perfluoroheptanoic acid ($C_6F_{13}COOH$; CAS - 375-85-9), ammonium perfluoroheptanoate (NH_4^+ $C_6F_{13}COO^-$; CAS - 6130-43-4), sodium perfluoroheptanoate (Na^+ $C_6F_{13}COO^-$; CAS - 20109-59-5)

PFHxA - Perfluoroalkyl carboxylic acids ($C_nF_{2n+1}COOH$) where n=5 and inclusive of all forms such as perfluorohexanoic acid ($C_5F_{11}COOH$; CAS - 307-24-4), ammonium perfluorohexanoate (NH_4^+ $C_5F_{11}COO^-$; CAS - 21615-47-4), sodium perfluorohexanoate (Na^+ $C_5F_{11}COO^-$; CAS - 2923-26-4)

PFHxS - Perfluoroalkane sulfonic acids ($C_nF_{2n+1}SO_3H$) with n=6 and inclusive of all forms such as perfluorohexane sulfonic acid ($C_6F_{13}SO_3H$; CAS - 355-46-4), perfluorohexane sulfonate anion ($C_6F_{13}SO_3^-$; CAS - 108427-53-8), potassium perfluorohexane sulfonate (K^+ $C_6F_{13}SO_3^-$; CAS - 3871-99-6)

PFNA - Perfluoroalkyl carboxylic acids ($C_nF_{2n+1}COOH$) where n=8 and inclusive of all forms such as perfluorononanoic acid ($C_8F_{17}COOH$; CAS - 375-95-1), ammonium perfluorononanoate (NH_4^+ $C_8F_{17}COO^-$; CAS - 4149-60-4), sodium perfluorononanoate (Na^+ $C_8F_{17}COO^-$; CAS - 21049-39-8)

PFOA - Perfluoroalkyl carboxylic acids ($C_nF_{2n+1}COOH$) where n=7 and inclusive of all forms such as perfluorooctanoic acid ($C_7F_{15}COOH$; CAS - 335-67-1), ammonium perfluorooctanoate (NH_4^+ $C_7F_{15}COO^-$; CAS - 3825-26-1), sodium perfluorooctanoate (Na^+ $C_7F_{15}COO^-$; CAS - 335-95-5), potassium perfluorooctanoate (K^+ $C_7F_{15}COO^-$; CAS - 2395-00-8)

PFOS - Perfluoroalkane sulfonic acids ($C_nF_{2n+1}SO_3H$) with n=8 and inclusive of all forms such as perfluorooctane sulfonic acid ($C_8F_{17}SO_3H$; CAS - 1763-23-1), perfluorooctane sulfonate anion ($C_8F_{17}SO_3^-$; CAS - 45298-90-6), and various salts (ammonium perfluorooctane sulfonate, (NH_4^+ $C_8F_{17}SO_3^-$; CAS - 29081-56-9), sodium perfluorooctane sulfonate (Na^+ $C_8F_{17}SO_3^-$; CAS - 4021-47-0), potassium perfluorooctane sulfonate (K^+ $C_8F_{17}SO_3^-$; CAS - 2795-39-3), lithium perfluorooctane sulfonate (Li^+ $C_8F_{17}SO_3^-$; CAS - 29457-72-5), tetraethylammonium

perfluorooctane sulfonate ($N(C_2H_5)_4^+$ $C_8F_{17}SO_3^-$; CAS - 56773-42-3), diethanolammonium perfluorooctane sulfonate ($NH_2(CH_2CH_2OH)^{2+}$ $C_8F_{17}SO_3^-$; CAS - 56773-42-3)

PFSAs - Perfluoroalkane sulfonic acids ($C_nF_{2n+1}SO_3H$)

PFUnDA - Perfluoroalkyl carboxylic acids ($C_nF_{2n+1}COOH$) where n=10 and inclusive of all forms such as perfluoroundecanoic acid ($C_{10}F_{21}COOH$; CAS - 2058-94-8), ammonium perfluoroundecanoate (NH_4^+ $C_{10}F_{21}COO^-$; CAS - 4234-23-5), perfluoroundecanoate ($C_{10}F_{21}COO^-$; CAS - 196859-54-8)
