# Supplementary material for: Assessing the Ecological Risks of Per‐ and Polyfluoroalkyl Substances: Current State‐of‐the Science and a Proposed Path Forward
Source: Environ Toxicol Chem. 2020 Nov 6;40(3):564–605. doi: 10.1002/etc.4869 (PMC7984443; doi:10.1002/etc.4869)
Supplement: Supplementary file 2 — Supporting information. [file ETC-40-564-s007.pdf]

SI 2 – Overview of Current Knowledge of Ecological Exposures to Perfluoroalkyl and polyfluoroalkyl Substances (PFAS)

1. PFAS Concentrations in Abiotic Media

Some PFAS are nearly ubiquitous in environmental media (McCarthy et al. 2017) and a variety of PFAS have been measured in multiple media in a variety of environments worldwide. Examples of PFAS found in surface water, sediment, and soil in a variety of habitats are presented in SI-2 Table 1. General trends from these data include:

- Recent studies have detected and quantitated an increasing number of PFAS as analytical methods evolve. Older studies did not detect many of these PFAS, but also were not targeting them.
- Spatial analysis of PFAS generally find urban areas to have higher concentrations than more isolated systems.
- PFOS and PFOA are detected in almost all media.
- Actual concentrations are wide ranging, but can be substantial—for example, PFOA and PFOS in surface water, sediment and soil samples have been reported as high as 33,900 ng PFOA/L and 5,900 ng PFOS/L, and 203 ng/g PFOA and 11 ng PFOS/g, respectively (SI-2 Table 1). These elevated levels are associated with point sources, as PFOA and PFOS background concentrations in surface water and soils and sediments are typically less than 5 ng/L and 10 ng/g, respectively.

2. PFAS Fate and Transport

Per- and poly-fluoroalkyl substances are a large group of substances with diverse structures and potentially wide disparities in associated physico-chemical properties (Buck et al. 2011). They are typically sub-divided into two categories, polymers and non-polymers, and these categories are divided again into several sub-classes. Polymers are generally in one of three classes (i.e., fluoropolymers, perfluoropolyether polymers, and side-chain fluorinated polymers), while the non-polymer category is divided into two classes (i.e., perfluoroalkyl substances and polyfluoroalkyl substances). The number and diversity of PFAS, and their associated physico-chemical properties, makes it difficult to provide a comprehensive assessment of their fate and transport in the environment. Factors that play a role in evaluating their environmental fate and transport include the (sub-)class of PFAS under consideration, manufacturing processes (e.g., single substances versus intentionally manufactured mixtures and “products by process”), emission patterns (e.g., air versus water versus soil), and degradation/decomposition of original source materials. Some PFAS are present as raw materials and intermediates in industrial and consumer products manufactured globally and may also occur as manufacturing byproducts or impurities, as metabolites, and as degradation products. The uses (e.g., industrial versus consumer) and ultimate disposal of the final products containing PFAS will also have ramifications for the environmental fate and transport of PFAS. Additional discussion of PFAS fate and transport considerations are included in Section 2 of this paper which discusses in

greater detail the environmental occurrence and persistence of PFAS in the context of prioritizing those of greatest concern.

3. PFAS Concentrations in Biota

PFAS have been measured and detected in biota from a multitude of habitats worldwide (SI-2 Table 2). Since the first report and reviews on PFAS monitoring in fish and wildlife (Giesy and Kannan 2010; Houde et al. 2006; Lau et al. 2007), multiple publications have summarized the global accumulation of these compounds in biota from Asia (Meng et al. 2017; Wang et al. 2020), Latin America (Llorca et al. 2017), Australia (EPA Victoria 2019 ; Gaylard 2016) and the North American Great Lakes (Remucal 2019) to the Arctic (Butt et al. 2010; Letcher et al. 2010; Muir et al. 2019) and Antarctica (Gao et al. 2020). General observations concerning PFAS in biota include:

- Biological matrices studied include terrestrial plants, lichens, invertebrates, fish, terrestrial and aquatic birds, wolves, caribou, sea turtles, alligators, as well as marine mammals.
- Depending upon the study and taxa sampled, multiple tissues have been investigated including whole body homogenates, muscle, liver, blubber, brain, gonads, kidney, heart, skin, feathers, gill, blood, plasma, eggs, and milk.
- PFAS are widely spatially distributed in species from terrestrial, freshwater, marine and estuarine environments in polar, temperate and tropical ecosystems.
- Most of the analytical emphasis have been on a common suite of perfluoroalkyl carboxylic acids (PFCA) and perfluoroalkyl sulfonic acids (PFSA) but increasingly other PFAS have been measured in recent years as analytical techniques have become more sophisticated and standards more readily available.

Evolving analytical technologies have enabled the detection of hundreds of novel PFAS in the past decade. A review by Xiao (2017) reports that between 2009 and 2017, >450 new PFAS, including anions, zwitterions, cations, and neutrals, have been identified in fish as well as many abiotic samples (e.g., water, fish, sediment, wastewater, sludge, soils). A curated summary by Wang et al. (2019) also indicated that hexafluoropropylene oxide dimer and hexafluoropropylene trimer acids, and 6:2 chlorinatedpolyfluorinated ether sulfonic acid have become globally distributed pollutants. New PFAS, such as perfluoroethylcyclohexanesulfonate, perfluorobutane sulfonamide (a precursor of perfluorobutane sulfonate) and polyfluorinated ether sulfonates, have also been reported in the Arctic environment (Muir et al. 2019).

4. PFAS Bioaccumulation

Several experimental and monitoring studies have evaluated the bioaccumulation of PFAS in different aquatic species, with most of the work thus far being in fish, but also some in invertebrates. The largest amount of fish bioaccumulation data are for PFOS and PFOA, however, a smaller number of fish studies also have examined accumulation of other PFAS such as perfluorododecanoic acid, perfluoroundecanoic acid, perfluorohexane sulfonate and perfluoroalkyl phosphinic acids (Martin et al. 2003; NJDEP 2018). The fish that have been

studied most frequently are freshwater species such as rainbow trout, fathead minnow, largemouth bass, and common carp, but there also has been PFAS bioaccumulation work with some marine fish (e.g., fang goby)(Wang et al. 2020). Several general observations have emerged from bioaccumulation studies with aquatic organisms. For example, in fish, fluorinated sulfonates tend to be more bioaccumulative than fluorinated carboxylates of the same chain length, and fluorinated carboxylates with less than eight carbons are not considered bioaccumulative (Conder et al 2008). Yang et al. (2012) suggested that PFOS bioaccumulates and even biomagnifies in marine food chains whereas PFOA may not have the potential to biomagnify. Others have also suggested that PFOS may biomagnify in aquatic food webs (Sharpe et al. 2010; Hazelton et al. 2012). Biomagnification of PFOA may be lower than that for PFOS due to higher solubility allowing fish excretion through the gills (CRC CARE 2017). Bioaccumulation of PFOA into benthic organisms has been shown by Yang et al. (2012) in Bohai Bay, China whereas Thompson et al. (2011) showed no bioaccumulation in invertebrates from Sydney Harbor, Australia. Shorter chain PFCA and PFSA (of less than eight and six carbons, respectively) are not readily bioconcentrated or accumulated in fish (Conder et al. 2008; Martin et al. 2003; Houde et al. 2011). Giesy et al. (2010) suggested that dietary accumulation of PFAS (e.g., PFOS) in fish appears to be similar to bioconcentration from water and that uptake from diet may not play a significant role. However, Sakurai et al. (2013) did show that up to 25% of the PFOS body burden in flounder was due to ingestion of sediment particulate matter. Time to reach steady state varies across classes and species of organisms. While standardized bioaccumulation bioassays usually require a minimum of 28 days (USEPA 2000; ASTM 2010), Abercrombie et al. (2019) have shown northern leopard frogs reaching steady state for PFOS in just 10 days. Thus, time to steady state is an important consideration in designing/conducting bioaccumulation studies to support exposure assessments. It is also important to recognize that, once absorbed, PFAS precursors may metabolize to the commonly detected PFAS such as PFOS and PFOA (Gebbink et al. 2015; Galatius et al. 2013).

PFAS bioaccumulation in terrestrial plants is highly dependent on soil properties (Higgins and Luthy 2006) and plant species (Mudumbi et al. 2014). PFAS-specific physico-chemical properties, chain length and functional group also influence uptake by plants (Braunig et al. 2019). Ghisi (2019) conducted a broad review of possible sources of PFAS and their potential accumulation in agricultural plants, from where they can transfer to animals (including humans) through the food chain. Low accumulations of PFOA and PFOS were found in peeled potatoes and cereal seeds, while short-chain compounds accumulated at higher levels in leafy vegetables and fruits. Some studies have shown that vegetables and grains grown on agricultural lands that were irrigated with PFAS contaminated water can accumulate these compounds (Stahl et al. 2009; Lechner et al. 2011). Others have shown that PFAS in biosolids applied to agricultural fields can accumulate in silage and into the tissues of the animals feeding on the sileage (Lindstrom 2011) which has implications for both ecological and human health risk assessments. Blaine et al. (2013) measured higher concentrations in shoot or fruit than in root of radish, tomato, pea and celery for most of the PFAS, while Wen et al. (2016) determined higher concentrations in roots than in shoots when the uptake of PFAS by wheat was evaluated.

According to Scher et al. (2018), shorter chain compounds (such as perfluorobutanoic acid and to lesser extent perfluoropentanoic acid) had a greater uptake by plants than longer chain PFAS and short chain sulfonates. As short-chain PFAS serve as commercial substitutes for longer-chain compounds, there is a need for robust mass balance studies to better understand and predict their uptake, bioaccumulation and translocation into plants.

Soil-associated PFAS also can bioaccumulate in earthworms, with uptake being both through absorption via skin and from food through the gut. Bioaccumulation of PFCA increases with an increase in perfluorinated carbon chain length (Rich et al. 2014; Zhao et al. 2013 and 2014). The BAFs of PFSA are greater than those of PFCA with identical perfluorinated carbon chain length (Rich et al. 2014; Zhao et al. 2013 and 2014c). According to Munoz et al. (2020), the BAFs of long-chain zwitterionic fluorotelomers in earthworms are higher than those of short-chain PFCA, but lower than for PFOS. Fluorotelomer compounds such as fluorotelomer sulfonates and fluorotelomer sulfonamidoalkyl betaine may accumulate in invertebrate species with limited ability to metabolize these PFAS. Bioaccumulation factors of PFAS in earthworms decreases with increasing soil concentration (Zhao et al. 2013, 2014a,b; Das et al. 2015; He et al. 2016; Karnjanapiboonwong et al. 2018) and also influenced by soil texture and organic carbon content (Rich et al. 2014; Das et al. 2015; He et al. 2016; Zhao et al. 2016).

Studies have shown that some PFAS also readily bioaccumulate in other terrestrial species such as birds (Gebbink et al. 2012; Custer et al. 2012; Chen et al. 2018; Remucal 2019; EPA Victoria 2019). It is important to consider patterns of accumulation of PFAS in birds in detail. For most of the currently monitored PFAS, the magnitude of the accumulation is associated with the length of the carbon-fluorine chain and functional head group. For PFAS of similar carbon-chain length, sulfonates accumulate to a greater degree than carboxylates (Franklin 2016). These observations are supported by pharmacokinetic data collected in studies with a limited number PFAS compounds and species (Newsted et al. 2006, 2007; Yeung et al. 2009). In addition, studies with birds have found that the accumulation and elimination of PFAS in serum was concentration dependent with serum half-lives increasing with decreasing concentration. For instance, in dietary studies with mallards and quail exposed to mg PFOS/kg feed, serum half-lives ranged from 9 to 25 d while in chickens exposed to 85 ng PFOS/kg bw/d via drinking water (Tarazona et al. 2015) resulted in a serum half-life of 230 d, a value approximately 10-fold greater than that observed in dietary studies with mallards and northern bobwhites. The effect of concentration on the magnitude of the PFOS elimination rates has also been observed in several studies with rodents and monkeys (Chang et al. 2012). One possible mechanism associated with this effect is that in short-term, higher dose studies, there is a more rapid phase of PFOS elimination from serum as well as re-distribution of PFOS within the body that can result in an overestimate of elimination half-lives. In addition, the influence of capacity-limited, saturable mechanisms such as organic anion transporter 1 (OAT1) must be considered (Ng and Hungerbuhler 2013; Zhao et al. 2017). At concentrations that are more environmentally relevant, these anion transport systems have been shown to be involved in the resorption of PFOS in rodents and humans resulting in longer serum half-lives.

Physiologically based pharmacokinetic (PBPK) models represent a powerful computational tool to estimate bioaccumulation relative to simulating the absorption, tissue distribution, metabolism, and excretion of PFAS. Although PBPK models have been developed

and parameterized for specific PFAS in humans (i.e., Wong et al. 2014; Bonadona 2014; Verner et al. 2016; Goeden et al. 2019), PBPK models in non-human biota are limited. One example in fish is the 10-compartment PBPK model for PFOS kinetics in adult rainbow trout developed based on experimental data from fish fed a PFOS-spiked diet for 42 days, followed by depuration for 35 days (Vidal et al. 2019). Comparison of modeled to measured concentrations indicate accurate prediction of PFOS concentrations in both organs and feces. Based on model results, most PFOS is eliminated through feces, with loss through urine being minor. Vidal et al. (2019) also note that branchial uptake of PFOS may be significant even when concentrations in water are low. In PBPK studies with an avian model, Tarazona et al. (2015) evaluated the toxicokinetics of a low PFOS dose (0.085 mg/kg per d) in chickens. The data were best described by a single compartment first-order kinetics model, with an elimination half-life of approximately 230 d and assimilation efficiency of 0.66. Steady state serum concentrations of 65 mg PFOS/L were estimated following 210 weeks of continuous exposure. PFOS elimination rates in birds, however, differ from those for humans and monkeys (Tarazona et al. 2015). Additionally, bioaccumulation models based on high PFOS doses are likely to underestimate bioaccumulation of PFOS (Tarazona et al. 2015).

5. PFAS Trophic Magnification

BMFs and TMFs may be more relevant for determining the overall bioaccumulation potential of PFAS than BCF and BAF values derived from fish (which can excrete PFAS through the gills). BMFs, and particularly TMFs, based on concentrations in whole organisms provide a more useful measure of overall potential for food chain transfer. However, given PFAS partitioning into proteins of the liver and blood, many field measurements for PFAS have focused on these tissues, especially for higher trophic level organisms where whole body analysis is not feasible due to either sampling or laboratory processing constraints

Considerable data indicate that some PFAS are bioaccumulative and readily transported within food webs, accumulating to greater concentrations at higher trophic levels (McCarthy et al. 2017). BMFs and trophic magnification factors (TMFs) have been derived from field collected data to describe the behavior of these contaminants in food webs from a variety of ecosystems ranging from arctic (i.e., Kelly et al. 2009), to temperate (i.e., Munoz et al. 2017), and subtropical food webs (e.g., Loi et al. 2011).

One challenge, not unique to PFAS, relative to interpreting bioaccumulation and biomagnification studies from the standpoint of predictive modeling, which is, is that published BAF and BMF values vary greatly among studies and species, with little standardization in how data are reported (e.g., dry weight versus wet weight, and organic carbon normalization; McCarthy et al. 2017). Also, Burkhard et al. (2012) reported a lack of agreement among laboratory and field bioaccumulation metrics. A detailed review of 24 peer-reviewed studies reporting field-derived BMFs or TMFs for 14 PFAS, determined that the study-to-study (and even within-study) variability of the results is so great that the utility of these data for making definitive conclusions concerning the bioaccumulative behavior and food-web transfer of these compounds is limited (Franklin 2016). These authors identified several possible causes for the orders of magnitude variation in BMFs and TMFs, including differences in how the values are derived and presented; erroneously assuming steady-state conditions; diet composition, food ingestion and habitat use uncertainties; influence of metabolism of precursor compounds; etc.

The implementation of highly controlled studies is critical to mitigate the uncertainty associated with field collected BMF and TMF data.

References

Abercrombie SA, de Perre C, Choi YJ, Tornabene BJ, Sepúlveda, MS, Lee LS, Hoverman JT (2019). Larval amphibians rapidly bioaccumulate poly- and perfluoroalkyl substances. *Ecotox. Environ. Safety* 178:137-145

ASTM International (formerly American Society for Testing and Materials) (ASTM). 2010. Standard Guide for Determination of the Bioaccumulation of Sediment-associated contaminants by Benthic Invertebrates. E1688 – 10.

Blaine, A.C., Rich, C.D., Sedlacko, E.M., Hundal, L.S., Kumar, K., Lau, C., Mills, M.A., Harris, K.M., Higgins, C.P. 2013. Perfluoroalkyl acid distribution in various plant compartments of edible crops grown in biosolids-amended soils. *Environ Sci Technol*. 2014 Jul 15;48(14):7858-65. doi: 10.1021/es500016s.

Bonadona, F.F. 2014. Physiologically-based Pharmacokinetic (PBPK) Modeling of PCDD/FS and PFASs in Humans. PhD Dissertation. Department of Chemical Engineering, Rovira I Virgili University, Tarragona, Spain.

Braunig, J., Baduel, C., Barnes, C.M., Mueller, J.F., 2019. Leaching and bioavailability of selected perfluoroalkyl acids (PFAAs) from soil contaminated by firefighting activities. *Sci. Total Environ.* 646, 471-479.

Buck RC, J Franklin, U Berger, JM Conder, IT Cousins, P de Voogt, AA Jensen, K Kannan, SA Mabury, SPJ van Leeuwen. 2011. Perfluoroalkyl and polyfluoroalkyl substances in the environment: Terminology, classification, and origins. *Integr Environ Assess and Manag*. 7 (4):513-541. doi: 10.1002/ieam.258.

Burkhard, L P, J A Arnot, M R Embry, KJ Farley, RA Hoke, M Kitano, H A Leslie, G R Lotufo, T F Parkerton, K G Sappington, G T Tomy, and Woodburn KB. 2012. Comparing Laboratory and Field Measured Bioaccumulation Endpoints. *Integr Environ Assess and Manag*. 8 (1) 17–31

Karnjanapiboonwong, A., Deb, S.K., Subbiah, S., Wang, D., Anderson, T.A., 2018. Perfluoroalkylsulfonic and carboxylic acids in earthworms (Eisenia fetida): accumulation and effects results from spiked soils at PFAS bracketing environmental relevance. *Chemosphere*, 199, 168–173.

Butt C, Berger U, Bossi R, Tomy G. 2010. Levels and trends of poly- and perfluorinated compounds in the arctic environment. *Sci. Total. Environ.* 408:2936-2965.

Chang SC, Noker PE, Gorman GS, Gibson SJ, Hart J, Ehresman DJ, Butenhoff JL. 2012. Comparative pharmacokinetics of perfluorooctane sulfonate (PFOS) in rats, mice, and monkeys. *Reprod Toxicol* 33:428-440.

Chen H, Han J, Cheng J, Sun R, Wang X, Han G, Yang W, He X. 2018. Distribution, bioaccumulation and trophic transfer of chlorinated polyfluoroalkyl ether sulfonic acids in the marine food web of Bohai, China. *Environ Poll* 241:504-510.

Conder JM, RA Hoke, W de Wolf, MH Russell, RC Buck. 2008. Are PFCAs Bioaccumulative? A Critical Review and Comparison with Regulatory Criteria and Persistent Lipophilic Compounds. *Environ Sci Technol* 42 (4):995-1003. doi: 10.1021/es070895g.

CRC CARE 2017, Assessment, management and remediation guidance for perfluorooctanesulfonate (PFOS) and perfluorooctanoic acid (PFOA) – Part 3: ecological screening levels, CRC CARE Technical Report no. 38, CRC for Contamination Assessment and Remediation of the Environment, Newcastle, Australia.

Custer, CM, TW Custer, HL Schoenfuss, BH Poganski, L Solem. 2012. Exposure and effects of perfluoroalkyl compounds on tree swallows nesting at Lake Johanna in east central Minnesota, USA. *Reprod. Toxicol* 33:556–562

Das, P., Megharaj, M., & Naidu, R. (2015). Perfluorooctane sulfonate release pattern from soils of fire training areas in Australia and its bioaccumulation potential in the earthworm Eisenia fetida. *Environ Sci Pollut Res* 22:8902–8910.

EPA Victoria. 2019. PFAS in Victorian waterfowl Investigation of the presence of PFAS in 19 wetlands in Victoria. Publication1734. March.

Franklin J. 2016. How reliable are field-derived biomagnification factors and trophic magnification factors as indicators of bioaccumulation potential? Conclusions from a case study on per- and polyfluoroalkyl substances. *Integ Environ Assess Manage* 12:6-20.

Galatius, A, R Bossi, C Sonne, F Farsø Rigét, C Kinze, C Lockyer, J Teilmann, R Dietz. 2013. PFAS profiles in three North Sea top predators: Metabolic differences among species? *Environ*. Vol. 20.

Gao K, Miao X, Fu J, Chen Y, Li H, Pan W, Fu J, Zhang Q, Zhang A, Jiang G. 2020. Occurrence and trophic transfer of per- and polyfluoroalkyl substances in an Antarctic ecosystem. *Environ. Poll.* 257: 113383.

Gaylard, S. 2016. Per and polyfluorinated alkyl substances (PFAS) in the marine environment. SA EPA report, Australia. ISBN 978-1-921495-79-3.

Gebbink WA, Letcher RJ. 2012. Comparative tissue and body compartment accumulation and maternal transfer to eggs of perfluoroalkyl sulfonates and carboxylates in Great Lakes herring gulls. *Environ Poll* 162:40-47.

Gebbink, WA., U Berger, IT Cousins. 2015. Estimating human exposure to PFOS isomers and PFCA homologues: The relative importance of direct and indirect (precursor) exposure. *Environ Inter* 74:160-169. doi: https://doi.org/10.1016/j.envint.2014.10.013.

Ghisi, R., Vamerali, T., Manzetti, S. 2019.Accumulation of perfluorinated alkyl substances (PFAS) in agricultural plants: A review. *Environ Res.* 169, 326-34

Giesy JP, Kannan K. 2001. Global distribution of perfluorooctane sulfonate in wildlife. *Environ. Sci. Technol.* 35: 1339-1342.

Giesy, JP, J Naile, JS Khim, P Jones, J Newsted. 2010. *Aquatic Toxicology of Perfluorinated Chemicals*. Vol. 202.

Goeden, HM, CW Greene, JA Jacobus. 2019. A transgenerational toxicokinetic model and its use in derivation of Minnesota PFOA water guidance. *Journal of Exposure Science & Environmental Epidemiology* 29 (2):183-195. doi: 10.1038/s41370-018-0110-5.

Hazelton, PD, Cope, WG, Pandolfo, TJ, Mosher, S, Strynar, MJ, Barnhart, MC & Bringolf, RB 2012. Partial life-cycle and acute toxicity of perfluoroalkyl acids to freshwater mussels, *Environ Toxicol Chem*. 31:1611–1620.

He, W., Megharaj, M., & Naidu, R. (2016). Toxicity of perfluorooctanoic acid towards earthworm and enzymatic activities in soil. *Environ Monit Assess* 188:424

Higgins, CP, Luthy, RG, 2006. Sorption of perfluorinated surfactants on sediments. *Environ. Sci. Technol*. 40 (23), 7251-7256.

Houde M, Martin JW, Letcher RJ, Solomon KR, Muir DCG. 2006. Biological monitoring of polyfluoroalkyl substances: a review. *Environ. Sci. Technol.* 40:3463-3473.

Houde, Magali, Amila De Silva, Derek C G Muir, and Robert Letcher. 2011. *Monitoring of Perfluorinated Compounds in Aquatic Biota: An Updated Review PFCs in Aquatic Biota*. Vol. 45.

Kelly BC, MG Ikonomou, JD Blair, B Surridge, D Hoover, R Grace, FAPC Gobas. *Environ Sci Technol 43*: 4037-4043. DOI: 10.1021/es9003894

Lau C, Anitole K, Hodes C, Lai D, Pfahles-Hutchens A, Seed J. 2007. Perfluoroalkyl acids: A review of monitoring and toxicological findings. *Toxicol. Sci.* 99:366-394.

Lechner, M., Knapp, H., 2011. Carryover of perfluorooctanoic acid (PFOA) and perfluorooctane sulfonate (PFOS) from soil to plant and distribution to the different plant compartments studied in cultures of carrots (*Daucus carota ssp. sativus*), potatoes (*Solanum tuberosum*), and cucumbers (*Cucumis sativus*). *J. Agric. Food Chem*. 59, 11011–11018

Letcher R, Bustnes J, Dietz R, Jenssen B, Jørgensen E, Sonne C, Verreaul J, Vijayan M, Gabrielsen G. 2010. Exposure and effects assessment of persistent organohalogen contaminants in arctic wildlife and fish. *Sci. Total Environ.* 408:2995-3043.

Lindstrom AB, Strynar MJ, Delinsky AD, Nakayama SF, McMillan L, Libelo EL, Neill M, Thomas L. 2011. Application of WWTP biosolids and resulting perfluorinated compound contamination of surface and well water in Decatur, Alabama, USA. *Environ. Sci. Technol.* 45:8015-8021.

Llorca M, Farré M, Eljarrat E, Díaz-Cruz S, Rodríguez-Mozaz S, Wunderlin D, Barcelo D. 2017. Review of emerging contaminants in aquatic biota from Latin America: 2002-2016. *Environ. Toxicol. Chem.* 36:1716-1727.

Loi, EHI, LWY Yeung, S Taniyasu, PKS Lam, K Kannan, N Yamashita. 2011. *Environ. Sci. Technol. 45*: 5506-5513. DOI: 10.1021/es200432n

Martin, J. W., S. A. Mabury, K. R. Solomon, and D. C. Muir. 2003. "Bioconcentration and tissue distribution of perfluorinated acids in rainbow trout (Oncorhynchus mykiss)." *Environ Toxicol Chem* 22 (1):196-204.

McCarthy, C., Kappleman, W. & DiGuiseppi, W. 2017. Ecological Considerations of Per- and Polyfluoroalkyl Substances (PFAS). *Curr Pollution Rep* **3,** 289–301. https://doi.org/10.1007/s40726-017-0070-8

Meng J, Hong S, Wang T, Li Q, Yoon SJ, Lu Y, Giesy JP, Khim JS. 2017. Traditional and new POPs in environments along the Bohai and Yellow Seas: An overview of China and South Korea. *Chemosphere* 169:503-515.

Mudumbi, J.B.N., Ntwampe, S.K.O., Muganza, M., Okonkwo, J.O., 2014. Susceptibility of Riparian wetland plants to perfluorooctanoic acid (PFOA) accumulation. *Int. J. Phytoremediation* 16 (9), 926-936.

Muir D, Bossi R, Carlsson P, Evans M, De Silva A, Halsall C, Rauert C, Herzke D, Hung H, Letcher R, Rigét F, Roos A. 2019. Levels and trends of poly- and perfluoroalkyl substances in the Arctic environment – An update. *Emer. Contam.* 5:240-271.

Munoz, G, H Budzinski, M Babut, H Drouineau, M Lauzent, et al.. 2017. Evidence for the Trophic Transfer of Perfluoroalkylated Substances in a Temperate Macrotidal Estuary. *Environ. Sci. Technol* 51: 8450-8459. 10.1021/acs.est.7b02399.

Munoz, G.; Desrosiers, M.; Vetter, L.; Vo Du, S.; Jarjour, J.; Liu, J.; Sauvé, S. 2020. Bioaccumulation of Zwitterionic Polyfluoroalkyl Substances in Earthworms Exposed to Aqueous Film-Forming Foam Impacted Soils. 54:1687-1697

Newsted JL, Beach SA, Gallagher S, Giesy, JP. 2006. Pharmacokinetics and Acute Lethality of Perfluorooctane sulfonate (PFOS) to the Mallard and Northern Bobwhite. *Arch. Environ. Contam. Toxicol*. 50, 411-420.

Newsted JL, Coady KK, Beach SA, Gallagher SP, Giesy JP. 2007. Effects of perfluorooctane sulfonate (PFOS) on mallard (Anas platyrhynchus) and bobwhite quail (Colinus virginianus) when chronically exposed via the diet. *Environ. Toxicol. Pharmacol*. 23: 1-9.

Ng CA, Hungerbuhler A. 2013. Bioconcentration of perfluorinated alkyl acids: how important is specific binding? *Environ Sci Technol* 47:7214-7223.

NJDEP. 2018. Investigation of levels of perfluorinated compounds in New Jersey fish, surface water, and sediment. SR15-010: Division of Science New Jersey Department of Environmental Protection, Research, and Environmental Health.

Remucal CK. 2019. Spatial and temporal variability of perfluoralkyl substances in the Laurentian Great Lakes. *Environmental Science: Processes & Impacts*:1-19.

Rich, C.D., Blaine, A.C., Hundal, L., Higgins, C.P., 2014. Bioaccumulation of perfluoroalkyl acids by earthworms (Eisenia fetida) exposed to contaminated soils. *Environ. Sci. Technol*. 49, 881–888.

Sakurai, T, Kobayashi, J, Kinoshita, K, Ito, N, Serizawa, S, Shiraishi, H, Lee, J-H, Horiguchi, T, Maki, H, Mizukawa, K, Imaizumi, Y, Kawai, T & Suzuki, N 2013, 'Transfer kinetics of Perfluorooctane sulfonate from water and sediment to a marine benthic fish, the marbled flounder (Pseudopleuronectes yokohamae)', *Environ Toxicol Chem* 32:2009–2017.

Scher, D.P., Kelly, J.E., Huset, C.A., Barry, K.M., Hoffbeck, R.W., Yingling, V.L., Messing, R.B., 2018. Occurrence of perfluoroalkyl substances (PFAS) in garden produce at homes with a history of PFAS-contaminated drinking water. *Chemosphere* 196:548–555.

Sharpe, RL, Benskin, JP, Laarman, AH, MacLeod, SL, Martin, JW, Wong, CS & Goss, GG 2010, 'Perfluorooctane sulfonate toxicity, isomer-specific accumulation and maternal transfer in zebrafish (Danio rerio) and rainbow trout (Oncorhynchus mykiss)', *Environ Toxicol Chem* 29:1957–1966.

Stahl, T., Heyn, J., Thiele, H., Hüther, J., Failing, K., Georgii, S., Brunn, H., 2009. Carryover of perfluorooctanoic acid (PFOA) and perfluorooctane sulfonate (PFOS) from soil to plants. *Arch. Environ. Contam. Toxicol*. 57:289–298.

Tarazona JV, Rodriguez C, Alonso E, Saez M, Gonzalez F, San Andres MD, Jimenez B, San Andres MI. 2015. Toxicokinetics of perfluorooctane sulfonate in birds under environmentally realistic exposure conditions and development of a kinetic predictive model. *Toxicol Lett* 232:363-368.

Thompson, J, Roach, A, Eaglesham, G, Bartkow, ME, Edge, K & Mueller, JF 2011, 'Perfluorinated alkyl acids in water, sediment and wildlife from Sydney Harbour and surroundings', *Marine Pollution Bulletin*, vol. 62, pp. 2869–2875.

USEPA (U.S. Environmental Protection Agency). 2000, Methods for Measuring the Toxicity and Bioaccumulation of Sediment-associated Contaminants with Freshwater Invertebrates, Second Edition, Office of Research and Development Mid-Continent Ecology Division, Duluth, Minnesota, Office of Science and Technology Office of Water Washington, D.C. EPA 600/R-

Verner, Marc-André, Gérard Ngueta, Elizabeth T. Jensen, Hermann Fromme, Wolfgang Völkel, Unni Cecilie Nygaard, Berit Granum, and Matthew P. Longnecker. 2016b. "A Simple Pharmacokinetic Model of Prenatal and Postnatal Exposure to Perfluoroalkyl Substances (PFASs)." *Environ. Sci. Technol* 50 (2):978-986. doi: 10.1021/acs.est.5b04399.99/064.

Vidal, A, M Babut, J Garric, R Beaudouin,2019. Elucidating the fate of perfluorooctanoate sulfonate using a rainbow trout (Oncorhynchus mykiss) physiologically-based toxicokinetic model. *Science of The Total Environment*. 691: 1297-1309. https://doi.org/10.1016/j.scitotenv.2019.07.105.

Wang Y, Chang W, Wang L, Zhang Y, Zhang Y, Wang M, Wang Y, Li P. 2019. A review of sources, multimedia distribution and health risks of novel fluorinated alternatives. *Ecotoxicol. Environ. Saf.* 182:109402.

Wang, Pei, Yonglong Lua, Hongqiao Sub, Chao Sub, Andrew C. Johnson, Longfei Yu, Alan Jenkins. 2020. "Managing health risks of perfluoroalkyl acids in aquatic food from a river-estuary-sea environment affected by fluorochemical industry". *Environment International* 138 (2020) 105621. https://doi.org/10.1016/j.envint.2020.105621

Wen, B., Wu,Y., Zhang, H., Liu, Y., Hu, X., Huang, H., Zhang, S. 2016. The roles of protein and lipid in the accumulation and distribution of perfluorooctane sulfonate (PFOS) and perfluorooctanoate (PFOA) in plants grown in biosolids-amended soils. *Environ Pollut*. 216, 682-688. doi: 10.1016/j.envpol.2016.06.032.

Wong F, M MacLeod, JF Mueller, IT Cousins. 2014. Enhanced Elimination of Perfluorooctane Sulfonic Acid by Menstruating Women: Evidence from Population-Based Pharmacokinetic Modeling. *Environ. Sci. Technol*. *48* (15), 8807-8814. DOI: 10.1021/es500796y

Xiao F. 2017. Emerging poly- and perfluoroalkyl substances in the aquatic environment: A review of current literature. *Water Res.* 124:482-495.

Yang, L, Tian, S, Zhu, L, Liu, Z & Zhang, Y 2012, 'Bioaccumulation and distribution of perfluoroalkyl acids in seafood products from Bohai Bay, China', *Environ Toxicol Chem* 31, no. 9, pp. 1972–1979.

Yeung LWY, Loi EIH, Wong VYY, Guruge KS, Yamanaka N, Tanimura N, Hasegawa J, Yamashita N, Miyazaki S, Lam PKS. 2009. Biochemical responses and accumulation properties of long-chain perfluorinated compounds (PFOS/PFDA/PFOA) in juvenile chickens (Gallus gallus). *Arch Environ Contam Toxicol* 57:377-386.

Zhang, D.Q., Wang, M., He, Q., Niu, X., Liang, Y. 2020. Distribution of perfluoroalkyl substances (PFASs) in aquatic plant-based systems: From soil adsorption and plant uptake to effects on microbial community. *Environ Pollut*. 257:113575. doi:10.1016/j.envpol.2019.113575. Epub 2019 Nov 6

Zhao, L., Bian, J., Zhang, Y., Zhu, L., Liu, Z., 2014a. Comparison of the sorption behaviors and mechanisms of perfluorosulfonates and perfluorocarboxylic acids on three kinds of clay minerals. *Chemosphere* 114, 51–58.

Zhao, L., Zhang, Y., Fang, S., Zhu, L., Liu, Z., 2014b. Comparative sorption and desorption behaviors of PFHxS and PFOS on sequentially extracted humic substances. *J. Environ. Sci*. 26, 2517–2525.

Zhao, L., Zhu, L., Zhao, S., Ma, X. (2016) Sequestration and bioavailability of perfluoroalkyl acids (PFAAs) in soils: Implications for their underestimated risk. *Science of the Total Environment*. 572: 169-176

Zhao, S., Zhu, L., Liu, L., Liu, Z., Zhang, Y., 2013. Bioaccumulation of perfluoroalkyl carboxylates (PFCAs) and perfluoroalkane sulfonates (PFSAs) by earthworms (Eisenia fetida) in soil. *Environ. Pollut*. 179, 45–52

Zhao, S., Fang, S., Zhu, L., Liu, L., Liu, Z., Zhang, Y., 2014c. Mutual impacts of wheat (Triticum aestivum L.) and earthworms (Eisenia fetida) on the bioavailability of perfluoroalkyl substances (PFASs) in soil. *Environ. Pollut*. 184, 495–501

Zhao W, Zitzow JD, Weaver Y, Ehresman DJ, Chang S-C, Butenhoff JL, Hagenbunch B. 2017. Organic anion transporting polypeptides contribute to the disposition of perfluoroalkyl acids in humans and rats. *Toxicol Sci* 156:84-95.

SI-2 Table 1. Overview of geographic and taxonomic coverage of PFASs in the environment

Geographic Area	Ecosystem			Abiotic media represented				
Geographic area	Marine	Freshwater	Terrestrial	Sediment	Water	Soil	PFAS Measured	Reference
Shanghai, China	X		X	X		X	TFA, PFHxA, PFOA, PFDA, PFOS, PFBA, PFPeA, PFNA, PFUnA, PFDoA, PFBS, PFHpA<br>Below detection limit: PFPrA, PFTA, PFHxS	Li et al. (2010)
San Francisco, CA, USA	X			X			PFHxS, PFOS, PFDS, POSAA, N-MeFOSAA, N-EtFOSAA, PFOA, PFNA, PFDA, PFUnA, PFDoA, PFTA	Higgins et al. (2015)
North Sea, Germany	X				X		PFBS, PFPS, PFHxS, PFOS, PFNS, 6:2 FTS, PFPA, PFHxA, PFHpA, PFOA, PFNA, PFDA, PFUnDA, PFDoDA, FOSA, MeFBSA, MeFBSE, FDUEA<br>Below detection limit: PFHpS, PFDS, PFTriDA, PFTeDA, PFPDA, PFHxDA, PFHpDA, PFOcDA, MeFOSA, EtFOSA, MeFOSE, EtFOSE, FHEA, FOEA, FDEA, FHUEA, FOUEA	Ahrens et al. (2009)
Yellow Sea, Dalian, China	X			X			PFOS, PFOA	Ju et al. (2008)
Tokyo Bay, Japan; South China Sea, Atlantic Ocean, Pacific Ocean	X				X		PFOS, PFHS, PFNA, PFOA, PFOSA<br>Below detection limit: PFBS	Yamashita et al. (2005)

SI-2 Table 1. Overview of geographic and taxonomic coverage of PFASs in the environment								
Geographic Area	Ecosystem			Abiotic media represented				
Geographic area	Marine	Freshwater	Terrestrial	Sediment	Water	Soil	PFAS Measured	Reference
Coastal Hong Kong, China	X				X		PFOSA, PFOS, PFNA, PFOA, PFHS, PFBS	So et al. (2004)
North Sea, Germany	X				X		PFHXxA, PFHxS, PFOA, PFOS, PFNoA, PFDeA, PFOSA, PFHpA	Caliebe et al. (2004)
Coastal Japan	X				X		PFOS<br>Below detection limit: PFHS, PFBS	Taniyasu et al. (2003)
Tokyo Bay, Japan	X			X			PFHpA, PFOA, PFNA, PFDA, PFUnDA, PFDoDA, PFTrDA, PFOS, N-MeFOSAA, N-EtFOSAA<br>Below detection limit: PFHxS, PFHpS, 8:2 FTCA, 10:2 FTCA, 82 FTUCA, 10:2 FTUCA	Zushi et al. (2010)
Yellow Sea, South Korea	X			X	X	X	PFBS, PFHxS, PFOS, PFDS, PFBA, PFHxA, PFHpA, PFOA, PFNA, PFDA, PFUnA, and PFDoA<br>Below detection limit: PFDoA (water), PFBS, PFHxS, PFDS, PFBA (sediment and soil), PFHxA and PFNA (sediment)	Naile et al. (2010)
Arctic Ocean	X				X		PFHxS, PFOS, FOSA, EtFOSAA, MeFOSAA, PFHxA, PFHpA, PFOA, PFNA, PFDA, PFUnDA, PFDoDA<br>Below detection limit: PFBS, PFDS, FOSAA	Yeung et al. (2017)

SI-2 Table 1. Overview of geographic and taxonomic coverage of PFASs in the environment

Geographic Area	Ecosystem			Abiotic media represented				
**Geographic area**	**Marine**	**Freshwater**	**Terrestrial**	**Sediment**	**Water**	**Soil**	**PFAS Measured**	**Reference**
Ebro Delta, Spain	X			X	X		PFPeA, PFHxA, PFHxS, PFHpA, PFOA, PFNA, PFOS, PFDA, PFDS, PFUdA<br>Below detection limit: PFBS, PFOSA, PFDoA	Pignotti et al. (2017)
Defense sites, USA		X	X	X	X	X	PFBA, PFBS, PFPA, PFHxA, PFHxS, PFHpA, PFOA, PFOSA, PFOS, PFNA, PFDA, PFDS, PFUnA, PFDoA, PFTriA, PFTeA<br>Below detection limit: PFTriA and PFTeA (water)	Anderson et al. (2016)
Lake Superior and Lake Michigan, North America		X			X		PFHxA, PFHpA, PFOA, PFNA, PFOS, PFDA, PFUnA	Simcik and Dorweiler (2005)
New York State, USA		X			X		PFOS, PFOA, PFHS<br>Below detection limit: PFBS, PFOSA	Sinclair et al. (2006)
Lakes Superior, Michigan, Huron, Erie, Ontario		X			X		PFHxS, PFOS, PFOSA, PFOA, PFNA, PFDA<br>Below detection limit: PFHpA, PFUnA, PFDoA, PFTrA, PFTeA, PFPA, PFDS, 8:2 FTUCA, 10:2 FTUCA	Furdui et al. (2007)
Lake Ontario, North America		X		X	X		PFOS (isomers)	Houde et al. (2008)

SI-2 Table 1. Overview of geographic and taxonomic coverage of PFASs in the environment

Geographic Area	Ecosystem			Abiotic media represented				
**Geographic area**	**Marine**	**Freshwater**	**Terrestrial**	**Sediment**	**Water**	**Soil**	**PFAS Measured**	**Reference**
Lake Superior, North America		X			X		Water: PFHxA, PFHpA, PFOA, PFNA, PFDA, PFUA, PFDoA, PFBS, PFHxS, PFOS, 6:2FTS, PFOSA<br>Waste water treatment plant: TFA, PFPrA, PFBA, PFPeA, PFHxA, PFHpA, PFOA<br>Below detection limit: PFDeA, PFTrDA, PFTeA, FHUEA, POUEA, PFHpS, PFDS	Scott et al. (2010)
Lakes Superior, Huron, Michigan, Erie, Ontario, North America		X			X		PFPeA, PFHxA, PFHpA, PFOA, PFUnA, PFDA, PFBS, PFHxS, PFOS, PFECHS, PFMeCHS<br>Below detection limit: PFTeA, PFDS	De Silva et al. (2011)
Lake Ontario, North America		X		X	X		PFHxS, PFOS, FOSA, PFHpA, PFOA, PFNA, PFDA, PFUnDA, PFDoDA, PFDS,<br>Below detection limit: PFTeDA	Myers et al. (2012)
Lake Michigan, North America		X		X			PFBS, PFPeA, PFTeDA, PFOS, PFBA, PFOA, FOSA, N-MeFOSE and N-EtFOSE<br>Below detection limit: N-MeFOSA, and N-EtFOSA, PFHxA, PFHpA, PFNA, PFDA, PFUnA, PFDoA, PFTrDA, PFODA, PFHxS, PFDS, FOSAA, MeFOSAA, EtFOSAA	Codling et al. (2014)
Daliao River watershed, China		X		X			PFBS, PFOS, PFOA, PFDA, PFDoA<br>Below detection limit: PFHxS, PFNA, PFTA	Bao et al. (2009)

SI-2 Table 1. Overview of geographic and taxonomic coverage of PFASs in the environment

Geographic Area	Ecosystem			Abiotic media represented				
**Geographic area**	**Marine**	**Freshwater**	**Terrestrial**	**Sediment**	**Water**	**Soil**	**PFAS Measured**	**Reference**
Canadian Arctic		X		X	X		PFHxS, PFOS, PFDS, PFHpA, PFOA, PFNA, PFDA, PFUA, PFDoA, 8:2 FTUCA, 10:2 FTUCA, PFBS (sediment)<br>Below detection limit: PFTriA, PFTetA, PFPA, 6:2 FTOH, 8:2 FTOH, 10:2 FTOH, NMeFBSE, NEtFBSE, PFOSA, NEtFOSA, NMeFOSE, NEtFOSE;	Stock et al. (2007)
Lake Erie, Ontario, St. Clair, North America		X		X			PFOS, PFOA, PFPEA, PFHxA, PFHpA, PFNA, PFDA, PFUdA, PFDoA, PFBS, PFHxS, PFBA, PFDS, PFTrDA, PFTeDA, PFHxDA, N-MeFOSAA, NeTFOSSA, FOSAA, FOSA<br>Below detection limit: N-MeFOSE, N-EtFOSE	Codling et al. (2018)
Plastic Lake, Lake Tettegouche, Lake Ontario, North America		X		X			PFHpA, PFOA, PFNA, PFDA, PFUnDA, PFDoDA, PFTrDA, PFTeDA, PFHxS, PFOS, PFDS, 6:2 diPAP, 8:2 diPAP, C6/C6 PFPiA, C6/C8 PFPiA<br>Below detection limit: 10:2 diPAP, C8/C8 PFPiA, C6-PFPA, C8-PFPA, C10-PFPA	Guo et al. (2016)
Lake Ontario, North America		X		X			PFOS, PFHxS, FOSA, PFDoDA, PFUnDA, PFDA, PFNA, PFOA, PFHpA, PFHxA, PFPeA, PFBA<br>Below detection limit: PFDS, PFBS	Yeung et al. (2013)
South Korea		X		X	X		PFHxA, PFHpA, PFOA, PFNA, PFDA, PFUnA, PFDoA, PFHxS, PFOS<br>Below detetction limit: PFDS	Lam et al. (2014)
France		X		X	X		PFBA, PFPeA, PFHxA, PFHpA, PFOA, PFNA, PFDA, PFUnA, PFDoA, PFTrDA, PFBS, PFHxS, PFHpS, Br, PFOS, L-PFOS, FOSA, MeFOSA, EtFOSA, 6:2 FTSA<br>Below detection limit: PFBA, PFPeA, PFHxA, 6:2 FTSA (Sediment); PFTeDA (water)	Munoz et al. (2015)

SI-2 Table 1. Overview of geographic and taxonomic coverage of PFASs in the environment

**Geographic Area**	**Ecosystem**			**Abiotic media represented**				
**Geographic area**	**Marine**	**Freshwater**	**Terrestrial**	**Sediment**	**Water**	**Soil**	**PFAS Measured**	**Reference**
Vaal River, South Africa		X		X	X		PFPeA, PFHxA, PFHpA, PFNA, PFOA, PFBS, PFHxS, PFOS (PFOS only PFAS detected in sediment)<br>Below detection limit: PFBA, PFDA, PFUdA, PFDoA, PFTrA, PFTeA and PFDS (water)	Groffen et al. (2018)
China		X		X			PFBA, PFPeA, PFHxA, PFHpA, PFOA, PFNA, PFDA, PFUnDA, PFDoDA, PFTrDA, PFTeDA, PFOS	Qi et al. (2016)
Lake Tana, Ethiopia		X		X	X		(Water) PFBA, PFHxA, PFOA, PFNA, PFDA, PFUnDA, PFHxS, PFOS<br>(Sediment) PFBA, PFHxA, PFHpA, PFDA, PFUnDA, PFDoDA, PFTriDA, PFTeDA, PFOS<br>Below detection limit: PFPeA, PFBS, PFHxA, 6:2 FTSA, FOSAA, PFDA, N-MeFOSAA, FOSA, N-EtFOSAA, PFDS, N-MeFOSA, N-MeFOSE, PFTriDA, N-EtFOSA, N-EtFOSE, PFHxDA, PFOcDA	Ahrens et al. (2016)
Vietnam		X		X	X		PFHxA, PFHpA, PFOA, PFNA, PFDA, PFUnDA, PFDoDA, PFTrDA, PFTeDA, PFBS, PFHxS, PFOS, PFDS<br>Below detection limit (sediment only): PFHxA, PFHpA	Lam et al. (2017)
Artic lake, Canada		X		X	X		PFHxA, PFOA, PFDA, PFHxS, FOSA, 4:2 FTS, 8:2 FTS, PFOS,<br>Used to derive Σ PFAS, PFCA, PFSA: PFHpA, PFNA, PFUnA, PFDoA, PFTriA, PFTA, PFBS, PFDS, PFECHS	Lescord et al. (2015)

SI-2 Table 1. Overview of geographic and taxonomic coverage of PFASs in the environment

Geographic Area	Ecosystem			Abiotic media represented				
**Geographic area**	**Marine**	**Freshwater**	**Terrestrial**	**Sediment**	**Water**	**Soil**	**PFAS Measured**	**Reference**
North Carolina, Texas, Kentucky, Indiana, USA; Osaka, Hokkaido Japan; Mexico City, Mexico			X			X	PFTrDA, PFTeDA, PFDoA, PFUdA, PFDA, PFNA, PFOA, PFHpA, PFHxA, PFOS, PFHS<br>Below detection limit: PFDS, PFBS	Strynar et al. (2012)
Global survey			X			X	PFHxA, PFHpA, PFOA, PFNA, PFDA, PFUnDA, PFDoDA, PFTrDA, PFTeDA, PFHxS, PFOS, PFDS	Rankin et al. (2016)
Gardermoen, Norway			X			X	PFBA, PFPeA, PFHpA, PFOA, PFDeA, PFOS<br>Below detection limit: PFBS, PFHxS, PFHxA, 6:2 FTS, PFNA, 8:2 FTS	Hale et al. (2017)
North China			X			X	PFOA, PFNA, PFUdA, PFOS, PFBA, PFPeA, PFHxA, PFHpA, PFDA, PFDoA, PFBS<br>Below detection limt: PFDA, PFHxS	Meng et al. (2015)

Supplemental Information 2
Ankley et al. 2020

Assessing the Ecological Risks of Per- and Polyfluoroalkyl Substances: Current State-of-the Science and a Proposed Path Forward

SI-2 Table 1 References

[1] Ahrens L, Gashaw H, Sjöholm M, Gebrehiwot SG, Getahun A, Derbe E, Bishop K, Åkerblom S. 2016. Poly-and perfluoroalkylated substances (PFASs) in water, sediment and fish muscle tissue from Lake Tana, Ethiopia and implications for human exposure. *Chemosphere* 165:352-357.
[2] Ahrens L, Siebert U, Ebinghaus R. 2009. Temporal trends of polyfluoroalkyl compounds in harbor seals (Phoca vitulina) from the German Bight, 1999–2008. *Chemosphere* 76:151-158.
[3] Anderson RH, Long GC, Porter RC, Anderson JK. 2016. Occurrence of select perfluoroalkyl substances at US Air Force aqueous film-forming foam release sites other than fire-training areas: Field-validation of critical fate and transport properties. *Chemosphere* 150:678-685.
[4] Bao J, Jin Y, Liu W, Ran X, Zhang Z. 2009. Perfluorinated compounds in sediments from the Daliao River system of northeast China. *Chemosphere* 77:652-657.
[5] Caliebe C, Gerwinski W, Hühnerfuss H, Theobald N. 2004. Occurrence of perfluorinates organic acids in the water of the North Sea. *Organohalogen Comp* 66:4074-4078.
[6] Codling G, Sturchio NC, Rockne KJ, Li A, Peng H, Timothy JT, Jones PD, Giesy JP. 2018. Spatial and temporal trends in poly-and per-fluorinated compounds in the Laurentian Great Lakes Erie, Ontario and St. Clair. *Environ Pollut* 237:396-405.
[7] Codling G, Vogt A, Jones PD, Wang T, Wang P, Lu Y-L, Corcoran M, Bonina S, Li A, Sturchio NC. 2014. Historical trends of inorganic and organic fluorine in sediments of Lake Michigan. *Chemosphere* 114:203-209.
[8] De Silva AO, Spencer C, Scott BF, Backus S, Muir DC. 2011. Detection of a cyclic perfluorinated acid, perfluoroethylcyclohexane sulfonate, in the Great Lakes of North America. *Environ Sci Technol* 45:8060-8066.
[9] Furdui VI, Stock NL, Ellis DA, Butt CM, Whittle DM, Crozier PW, Reiner EJ, Muir DC, Mabury SA. 2007. Spatial distribution of perfluoroalkyl contaminants in lake trout from the Great Lakes. *Environ Sci Technol* 41:1554-1559.
[10] Groffen T, Wepener V, Malherbe W, Bervoets L. 2018. Distribution of perfluorinated compounds (PFASs) in the aquatic environment of the industrially polluted Vaal River, South Africa. *Sci Tot Environ* 627:1334-1344.
[11] Guo R, Megson D, Myers AL, Helm PA, Marvin C, Crozier P, Mabury S, Bhavsar SP, Tomy G, Simcik M. 2016. Application of a comprehensive extraction technique for the determination of poly-and perfluoroalkyl substances (PFASs) in Great Lakes Region sediments. *Chemosphere* 164:535-546.
[12] Hale SE, Arp HPH, Slinde GA, Wade EJ, Bjørseth K, Breedveld GD, Straith BF, Moe KG, Jartun M, Høisæter Å. 2017. Sorbent amendment as a remediation strategy to reduce PFAS mobility and leaching in a contaminated sandy soil from a Norwegian firefighting training facility. *Chemosphere* 171:9-18.
[13] Higgins CP, Field JA, Criddle CS, Luthy RG. 2005. Quantitative determination of perfluorochemicals in sediments and domestic sludge. *Environ Sci Technol* 39:3946-3956.
[14] Houde M, Czub G, Small JM, Backus S, Wang X, Alaee M, Muir DC. 2008. Fractionation and bioaccumulation of perfluorooctane sulfonate (PFOS) isomers in a Lake Ontario food web. *Environ Sci Technol* 42:9397-9403.
[15] Ju X, Jin Y, Sasaki K, Saito N. 2008. Perfluorinated surfactants in surface, subsurface water and microlayer from Dalian coastal waters in China. *Environ Sci Technol* 42:3538-3542.
[16] Lam NH, Cho C-R, Kannan K, Cho H-S. 2017. A nationwide survey of perfluorinated alkyl substances in waters, sediment and biota collected from aquatic environment in Vietnam: distributions and bioconcentration profiles. *Journal of hazardous materials* 323:116-127.
[17] Lam N-H, Cho C-R, Lee J-S, Soh H-Y, Lee B-C, Lee J-A, Tatarozako N, Sasaki K, Saito N, Iwabuchi K. 2014. Perfluorinated alkyl substances in water, sediment, plankton and fish from Korean rivers and lakes: a nationwide survey. *Sci Tot Environ* 491:154-162.
[18] Lescord GL, Kidd KA, De Silva AO, Williamson M, Spencer C, Wang X, Muir DC. 2015. Perfluorinated and polyfluorinated compounds in lake food webs from the Canadian High Arctic. *Environ Sci Technol* 49:2694-2702.
[19] Li F, Zhang C, Qu Y, Chen J, Chen L, Liu Y, Zhou Q. 2010. Quantitative characterization of short-and long-chain perfluorinated acids in solid matrices in Shanghai, China. *Sci Tot Environ* 408:617-623.
[20] Meng J, Wang T, Wang P, Zhang Y, Li Q, Lu Y, Giesy JP. 2015. Are levels of perfluoroalkyl substances in soil related to urbanization in rapidly developing coastal areas in North China? *Environ Pollut* 199:102-109.

[21] Munoz G, Giraudel J-L, Botta F, Lestremau F, Dévier M-H, Budzinski H, Labadie P. 2015. Spatial distribution and partitioning behavior of selected poly-and perfluoroalkyl substances in freshwater ecosystems: a French nationwide survey. *Sci Tot Environ* 517:48-56.
[22] Myers AL, Crozier PW, Helm PA, Brimacombe C, Furdui VI, Reiner EJ, Burniston D, Marvin CH. 2012. Fate, distribution, and contrasting temporal trends of perfluoroalkyl substances (PFASs) in Lake Ontario, Canada. *Environ Int* 44:92-99.
[23] Naile JE, Khim JS, Wang T, Chen C, Luo W, Kwon B-O, Park J, Koh C-H, Jones PD, Lu Y. 2010. Perfluorinated compounds in water, sediment, soil and biota from estuarine and coastal areas of Korea. *Environ Pollut* 158:1237-1244.
[24] Pignotti E, Casas G, Llorca M, Tellbüscher A, Almeida D, Dinelli E, Farré M, Barceló D. 2017. Seasonal variations in the occurrence of perfluoroalkyl substances in water, sediment and fish samples from Ebro Delta (Catalonia, Spain). *Sci Tot Environ* 607:933-943.
[25] Qi Y, Huo S, Xi B, Hu S, Zhang J, He Z. 2016. Spatial distribution and source apportionment of PFASs in surface sediments from five lake regions, China. *Scientific reports* 6:22674.
[26] Rankin K, Mabury SA, Jenkins TM, Washington JW. 2016. A North American and global survey of perfluoroalkyl substances in surface soils: Distribution patterns and mode of occurrence. *Chemosphere* 161:333-341.
[27] Scott BF, De Silva AO, Spencer C, Lopez E, Backus SM, Muir DC. 2010. Perfluoroalkyl acids in Lake Superior water: Trends and sources. *Journal of Great Lakes Research* 36:277-284.
[28] Simcik MF, Dorweiler KJ. 2005. Ratio of perfluorochemical concentrations as a tracer of atmospheric deposition to surface waters. *Environ Sci Technol* 39:8678-8683.
[29] Sinclair E, Mayack DT, Roblee K, Yamashita N, Kannan K. 2006. Occurrence of perfluoroalkyl surfactants in water, fish, and birds from New York State. *Arch Environ Contam Toxicol* 50:398-410.
[30] So M, Taniyasu S, Yamashita N, Giesy J, Zheng J, Fang Z, Im S, Lam PK. 2004. Perfluorinated compounds in coastal waters of Hong Kong, South China, and Korea. *Environ Sci Technol* 38:4056-4063.
[31] Stock NL, Furdui VI, Muir DC, Mabury SA. 2007. Perfluoroalkyl contaminants in the Canadian Arctic: evidence of atmospheric transport and local contamination. *Environ Sci Technol* 41:3529-3536.
[32] Strynar MJ, Lindstrom AB, Nakayama SF, Egeghy PP, Helfant LJ. 2012. Pilot scale application of a method for the analysis of perfluorinated compounds in surface soils. *Chemosphere* 86:252-257.
[33] Taniyasu S, Kannan K, Horii Y, Hanari N, Yamashita N. 2003. A survey of perfluorooctane sulfonate and related perfluorinated organic compounds in water, fish, birds, and humans from Japan. *Environ Sci Technol* 37:2634-2639.
[34] Yamashita N, Kannan K, Taniyasu S, Horii Y, Petrick G, Gamo T. 2005. A global survey of perfluorinated acids in oceans. *Mar Pollut Bull* 51:658-668.
[35] Yeung LW, De Silva AO, Loi EI, Marvin CH, Taniyasu S, Yamashita N, Mabury SA, Muir DC, Lam PK. 2013. Perfluoroalkyl substances and extractable organic fluorine in surface sediments and cores from Lake Ontario. *Environ Int* 59:389-397.
[36] Zushi Y, Tamada M, Kanai Y, Masunaga S. 2010. Time trends of perfluorinated compounds from the sediment core of Tokyo Bay, Japan (1950s–2004). *Environ Pollut* 158:756-763.

SI-2 Table 2. Overview of geographic and taxonomic coverage of PFASs in the biota.

**Geographic Area** *(what area is represented in the dataset)*	**Ecosystem** *(freshwater, marine, terrestrial)*	**Biological taxa represented** *(what taxa with PFAS data)*	**Tissues** *(which tissues are represented)*	**PFASs Measured** *(list which PFASs are represented in the dataset)*	**Notes** *(anything that might be especially relevant about this study)*	**Reference** *(citation for study)*
Kampala, Uganda	Terrestrial	Yam (*Dioscorea spp.*)<br>Maize (*Zea mays*)<br>Sugarcane (*Saccharum officinarum*)	Yam: root<br>Maize: cob<br>Sugarcane: stem	PFBA<br>PFPeA<br>PFHxA<br>PFHpA<br>PFOA<br>PFNA<br>PFDA<br>PFUnDA<br>PFDoDA<br>PFTrDA<br>PFTeDA<br>PFHxDA<br>PFOcDA<br>PFBS<br>PFHxS<br>PFOS<br>PFDS<br>FOSA<br>MeFOSA<br>EtFOSA<br>MeFOSE<br>EtFOSE<br>FOSAA<br>MeFOSAA<br>EtFOSAA	Of the 26 PFAS analyzed, only PFHpA, PFOA, PFNA, PFBS and FOSA were detected in plant samples.<br>PFCAs were better taken up by plants than PFSAs.	Dalahmeh et *al*. (2018)

SI-2 Table 2. Overview of geographic and taxonomic coverage of PFASs in the biota.

				FTSA		
Remote areas in Yukon and Northwest Territories, Canadian Arctic	Terrestrial	Lichen (C*ladonia mitis/Rangiferina and Flavocetraria nivalis/ Cucullate*)<br>Plants:<br>Cottongrass (*Eriophorum vaginatum*)<br>Aquatic sedge (*Carex aquatilis*)<br>Willow (*Salix pulchra*)<br>Moss (*Rythidium rugosum*)<br>Mushrooms (unknown species)<br>Barren ground caribou (*Rangifer tarandus groenlandicus*)<br>Wolves (*Canis lupus*)	Liver, muscle, kidney (caribou)<br>Liver, muscle (wolf)	PFHxA<br>PFHpA<br>PFOA<br>PFNA<br>PFDA<br>PFUnDA<br>PFDoDA<br>PFTrDA<br>PFTeDA<br>PFHxS<br>PFOS	All concentrations very low in vegetation, but PFCA congener composition differed among types of vegetation.<br>For caribou, PFAS concentrations in muscle and kidney were 10 to 20 times lower than in liver.<br>Wolf displayed similar PFAS distribution as caribou, concentration in liver 5 to 15 times higher than muscle.	Müller et *al*. (2011)
Blokkersdijk and Galgenweel, Belgium	Terrestrial	Fruits:<br>Common blackberry (*Rubus fruticosus*)<br>European elder (*Sambucus nigra*)<br>Invertebrates:<br>Isopods<br>Millipedes<br>Slugs<br>Worms	Mouse: liver, pancreas, lungs, kidneys, spleen<br>Vole: liver	PFOS	Median levels at Galgenweel for both fruit species are below detection limit of 2.0 ng/g.<br>High levels of PFOS in mice liver and kidneys high compared to literature.	D'Hollander et *al*. (2014)

SI-2 Table 2. Overview of geographic and taxonomic coverage of PFASs in the biota.

		Wood mouse (*Apodemus sylvaticus*)<br>Bank vole (*Myodes glareolus*)			PFOS concentrations in the livers of wood mice from Blokkersdijk highest ever reported in wildlife (data from Hoff et al. 2004).	
Lake Thana, Ethiopia	Freshwater	*Labeobarbus megastoma*<br>*Labeobarbus gorguari*<br>*Labeobarbus intermedius*<br>Nile Tilapia (*Oreochromis Niloticus*)<br>African catfish (*Clarias gariepinus*)	Muscle	PFBS<br>PFHxS<br>PFOS<br>PFDS<br>PFBA<br>PFPeA<br>PFHxA<br>PFHpA<br>PFOA<br>PFNA<br>PFDA<br>PFUnDA<br>PFDoDA<br>PFTrDA<br>PFTeDA<br>PFHxDA<br>PFOcDA<br>FOSA<br>MeFOSA<br>EtFOSA<br>MeFOSE<br>EtFOSE<br>FOSAA	-	Ahrens et *al*. (2016)

SI-2 Table 2. Overview of geographic and taxonomic coverage of PFASs in the biota.

				MeFOSAA<br>EtFOSAA<br>6:2 FTSA		
Vaal river, South Africa	Freshwater	Smallmouth yellowfish (*Labeobarbus aeneus*)<br>Orange River mudfish (*Labeo capensis*)<br>African sharptooth catfish (*Clarias gariepinus*)<br>Common carp (*Cyprinus carpio*)	Muscle<br>Liver	PFPeA<br>PFHxA<br>PFOA<br>PFNA<br>PFDA<br>PFUnDA<br>PFDoDA<br>PFTrDA<br>PFTeDA<br>PFBS<br>PFHxS<br>PFOS<br>PFDS<br><u>Not quantifiable or detected:</u><br>PFBA<br>PFHpA	-	Groffen et *al*. (2018)
Lake Halmsjön, Sweden	Freshwater	European perch (*Perca fluviatilis*)	Muscle<br>Blood<br>Liver<br>Gill<br>Gonad	PFHxA<br>PFHpA<br>PFOA<br>PFNA<br>PFDA<br>PFUnDA<br>PFHxS<br>PFOS<br>PFDS<br>PFOSA	-	Ahrens et al. (2015)

SI-2 Table 2. Overview of geographic and taxonomic coverage of PFASs in the biota.

				6:2 FTSA		
Charleston Harbor, Ashley River and Cooper River, South Carolina, United States	Estuarine	Atlantic croaker (*Micropogonias undulatus*)<br>Red drum (*Sciaenops ocellatus*)<br>Spot (*Leiostomus xanthurus*)<br>Spotted seatrout (*Cynoscion nebulosus*)<br>Striped mullet (*Mugil cephalus*)<br>Southern flounder (*Paralichthys lethostigma*)	Whole body<br>Muscle + skin (fillet)	PFPeA<br>PFHxA<br>PFHpA<br>PFOA<br>PFNA<br>PFDA<br>PFUnDA<br>PFDoDA<br>PFOS<br>PFHxS<br>PFOSA	Estimates dietary exposure to PFASs by fish consumption and assess risks in humans and dolphins	Fair et *al.* (2019)
Red, Dong Nai, Sai Gon, Mekong, Ma, Da Rang, Da Nong, Cai and Quan Truong Rivers, Vietnam	Freshwater	Stripped snakehead (*Chana striata*)<br>Dusky sleeper (*Eleotris fusca*)<br>Shark catfish (*Pangasius elongatus*)<br>Flying barb (*Esomus danricus*)<br>Tilapia (*Oreochrommic niloticus*)<br>Giant prawn (*Macrobranchium rosen-gbergii*)<br>Freshwater paddle crab (*Varuna litterata*)	Liver<br>Soft tissues<br>Whole body<br>Muscle	PDHxA<br>PFHpA<br>PFOA<br>PFNA<br>PFDA<br>PFUnDA<br>PFDoDA<br>PFTrDA<br>PFTeDA<br>PFBS<br>PFHxS<br>PFOS<br>PFDS	Investigates PFASs concentrations in water, sediment, various tissues of fish, crustacean, gastropod and bivalve	Lam et *al.* (2017)

SI-2 Table 2. Overview of geographic and taxonomic coverage of PFASs in the biota.

		Golden applesnail (*Pomacea canaliculata*) Golden freshwater clam (*Corbicula fluminea*)				
Rhône River, France	Freshwater	Barbel (Barbus barbus)<br>Gudgeon (Gobio gobio)<br>Roach<br>(Rutilus rutilus)	Fillet<br>Liver	PFHxS<br>PFHpS<br>PFOS<br>PFDS<br>FOSA<br>MeFOSA<br>EtFOSA<br>MeFOSAA<br>EtFOSAA<br>PFOA<br>PFNA<br>PFDA<br>PFUnDA<br>PFDoDA<br>PFTrDA<br>PFTeDA<br>6:2 FTSA	Investigates the influence of fish size, diet, prey contamination and biotransformation (with invertebrate and aquatic plant sampling and analysis)	Babut et *al*. (2017)
Labe, Vltava and Bílina rivers, Czech Republic	Freshwater	European chub (*Squalius cephalus*)<br>Common bream (*Abramis brama*)<br>Asp (*Aspius aspius*)<br>Roaches(*Rutilus rutilus*)<br>Crucian	Fillet	<u>Under LOQ:</u><br>PFBA<br>PFPeA<br>PFHxA<br>PFHpA<br>PFHxDA<br>PFOcDA<br>PFBS<br>N-EtFOSA	-	Svihlikova et *al*. (2015)

SI-2 Table 2. Overview of geographic and taxonomic coverage of PFASs in the biota.

		carp (*Carassius carassius*)<br>Common carp (*Cyprinus carpio*)<br>Nase carp (*Chondrostoma nasus*)		N-EtFOSE<br>N-MeFOSA<br>N-MeFOSE<br>PFHxPA<br>PFOPA<br>PFDPA<br><u>Detected above the LOQ:</u><br>PFOA<br>PFNA<br>PFDA<br>PFUnDA<br>PFDoDA<br>PFTrDA<br>PFTeDA<br>PFHxS<br>L-PFOS<br>Br-PFOS<br>PFDS<br>FOSA		
Port Stephens, Hunter River and Wallis Lake, Australia	Estuarine	Common silverbiddy (*Gerres subfasciatus*)<br>Dusky flathead (*Platycephalus fuscus*)<br>Luderick (*Girella tricuspidata*)<br>Sand Whiting (*Sillago ciliate*)	Muscle	PFOS<br>PFOA<br>8:2 FTS<br>6:2 FTS	-	Taylor et *al*. (2018)

SI-2 Table 2. Overview of geographic and taxonomic coverage of PFASs in the biota.

		Sea Mullet (*Mugil cephalus*)<br>Yellowfin Bream (*Acanthopagrus australis*)<br>Blue Swimmer Crab (*Portunus armatus*)<br>Mud Crab (*Scylla serrata*)<br>Eastern King Prawn (*Penaeus plebejus*)<br>School Prawn (*Metapenaeus macleayi*)				
Lake Varese, Italy	Freshwater	European perch (*Perca fluviatilis*)	Muscle	PFOS<br>PFOA	-	Squadrone et *al*. (2015)
Western Scheldt, The Netherlands<br>Water bodies across Canada<br>Great Lakes Region (Canada and United States)	Freshwater	Flounder (*Platichthys flesus*)<br>Tilapia<br>White bass<br>Australian bass<br>Lake trout<br>Trout perch	Muscle<br>Whole body<br>Liver	FBSA<br>FOSA<br>PFOS	-	Chu et *al*. (2016)
Unguja and Pemba islands, Zanzibar<br>Mtwara region, Tanzania	Marine	Milkfish (*Chanos chanos*)<br>Mullet (*Mugil cephalus*)	Muscle (because most of the liver tissue was	PFHxS<br>PFOS<br>PFOA<br>PFNA<br>PFDA	PFAS was not detected in any of the analysed fish muscle samples in levels above the LOD	Mwakalapa et *al*. (2018)

SI-2 Table 2. Overview of geographic and taxonomic coverage of PFASs in the biota.

			used for other analyses)	PFUnDA<br>PFDoDA<br>PFTrDA		
Ebro and Guadalquivir river basins, Spain	Freshwater	Guadiana bogue (*Pseudochondrostoma willkommii*)<br>Andalusian barbel (*Luciobarbus sclateri*)<br>Common carp (*Cyprinus carpio*)<br>Ebro barbel (*Barbus graellssi*)<br>European catfish (*Silurus glanis*)	Whole body	PFBA<br>PFHxA<br>PFHpA<br>PFOA<br>PFNA<br>PFDA<br>PFUnDA<br>PFBS<br>PFHxS<br>PFOS<br>PFDS<br>PFOSA	-	Lorenzo et *al*. (2016)
Atlantic ocean, southwest of Portugal	Marine	Blue shark (*Prionace glauca*)	Liver<br>Muscle	PFBS<br>PFHxS<br>PFHpS<br>PFOS<br>PFDS<br>PFBA<br>PFPeA<br>PFHxA<br>PFHpA<br>PFOA<br>PFNA<br>PFDA<br>PFUnDA<br>PFDoDA	Also analyzes other POPs and biochemical responses	Alves et *al*. (2016)
Po River and Comacchio Lagoon, Italy	Freshwater (Po River)	European eel (*Anguilla anguilla*)	Liver<br>Kidney	PFOS<br>PFOA	-	Giari et *al*. (2015)

SI-2 Table 2. Overview of geographic and taxonomic coverage of PFASs in the biota.

	Saltwater (Comacchio Lagoon)		Gonad<br>Blood<br>Muscle			
Hokkaido, Northern Japan	Marine	Pacific cod (*Gadus macrocephalus*)	Muscle	PFOA<br>PFNA<br>PFDA<br>PFUnDA<br>PFDoDA<br>PFTrDA<br>PFTeDA	-	Fujii et *al.* (2015)
Eastern Mediterranean Sea, Greece	Marine	Shortfin mako shark (*Isurus oxyrinchus*)<br>Angular roughshark (*Oxynotus centrina*)<br>Giant devil ray (*Mobula mobular*)<br>Smooth hammerhead (*Sphyrna zygaena*)<br>Smalltooth sand tiger (*Odontaspis ferox*)<br>Bigeye thresher (*Alopias superciliosus*)<br>Sharpnose sevengills shark (*Heptranchias perlo*)<br>Bluntnose sixgills shark (*Hexanchus griseus*)<br>Blue shark (*Prionace glauca*)	Muscle<br>Gills<br>Livers<br>Heart<br>Gonads	PFPeA<br>PFOA<br>PFNA<br>PFDA<br>PFUnDA<br>PFDoDA<br>PFTrDA<br>PFTeDA<br>PFBS<br>PFOS<br><LOQ in all samples:<br>PFHxA<br>PFHpA<br>PFHxS<br>PFHpS<br>PFDS	PFASs levels declined in the order of gonads<br>> heart > liver ≈ gills<br>> muscle	Zafeiraki et *al.* (2019)

SI-2 Table 2. Overview of geographic and taxonomic coverage of PFASs in the biota.

Ebro Delta, Catalonia, Spain	Marine (Seawater)<br>Freshwater	*Mugil cephalus*<br>*Squalius laietanus*<br>*Cyprinus carpio*<br>*Anguilla anguilla*<br>*Torpedo torpedo*<br>*Sarpa salpa*<br>*Trachurus mediterraneus*<br>*Boops boops*<br>*Diplodus annularis*<br>*Micropterus salmoides*<br>*Alburnus alburnus*<br>*Cyprinus carpio*<br>*Liza sp.*<br>*Rutilus rutilus*<br>*Scardinius Erythrophtalmus*<br>*Silurus glanis*<br>*Squalius laietanus*	Skin<br>Muscle<br>Whole body	PFPeA<br>PFHxA<br>PFHpA<br>PFOA<br>PFNA<br>PFDA<br>PFUnDA<br>PFDoDA<br>PFBS<br>PFHxS<br>PFOS<br>PFDS<br>PFOSA	The estuarine and marine biota analyzed in this study showed an accumulation of PFAS that were not found in waters or sediments	Pignotti et *al.* (2017)
Fullerton Cove and Tilligerry Creek, Australia	Saltwater	Dusky Flathead<br>Mud Crab<br>School Prawn<br>Sea Mullet<br>Yellowfin Bream<br>Eastern King Prawn<br>Sand Whiting	Muscle<br>Liver	PFHxA<br>PFHpA<br>PFOA<br>PFNA<br>PFDA<br>PFUnDA<br>PFDoDA<br>PFBS<br>PFHxS<br>PFOS<br>6:2 FTS	Also analyses PFAS in crustaceans	Taylor et Johnson (2016)

SI-2 Table 2. Overview of geographic and taxonomic coverage of PFASs in the biota.

				8:2 FTS		
Lakes Kroktjärn, Björntjärn, Brobo-Kroktjärn, Gårdsjön, Långtjärn and Mångstrettstjärn, Sweden	Freshwater	European perch (*Perca fluviatilis*)	Muscle	PFBS<br>PFHxS<br>PFOS<br>PFDS<br>PFBA<br>PFPeA<br>PFHxA<br>PFHpA<br>PFOA<br>PFNA<br>PFDA<br>PFUnDA<br>PFDoDA<br>PFTrDA<br>PFTeDA<br>PFHxDA<br>PFOcDA<br>FOSA<br>*N*-EtFOSA<br>*N*-MeFOSA<br>*N*-EtFOSE<br>*N*-MeFOSE<br>FOSAA<br>*N*-EtFOSAA<br>*N*-MeFOSAA<br>6 :2 FTSA	-	Åkerblom et *al*. (2017)
South China Sea (near Xisha islands and Huangyan island)	Marine	*Myristis murdjan*<br>*Melichthys vidua*<br>*Lethrinus olivaceus*	Muscle	<u>Detected above the LOQ:</u><br>PFOS	First report of the occurrence of PFAS in coral reef fishes in	Pan et *al*. (2018)

SI-2 Table 2. Overview of geographic and taxonomic coverage of PFASs in the biota.

		Gnathodentex aureolineatus<br>*Parupeneus trifasciatus*<br>*Lutjanus kasmira*<br>*Cephalopholis urodelus*		PFDA<br>PFUnDA<br>PFDoDA<br>PFTrDA<br>Not detected:<br>PFBA<br>PFPeA<br>PFBS<br>PFHxA<br>PFHpA<br>PFHxS<br>PFHpS<br>PFNA<br>PFDS<br>PFTeDA	the world (known to authors)	
Pacific coast of British Columbia, Canada (Langara Island, Cleland Island, Lucy Island, Hippa Island, Mandarte Island, Stanley Park)	Marine<br>Freshwater	Ancient murrelet (*Synthliboramphus antiquus*)<br>Leach's storm-petrels (*Oceanodroma leucorhoa*) Rhinoceros auklets (*Cerorhinca monocerata*) Double-crested cormorants (*Phalacrocorax auritus*)<br>Great blue herons (*Ardea herodias*)	Eggs	PFBS<br>PFHxS<br>PFOS<br>PFDS<br>PFHxA<br>PFHpA<br>PFOA<br>PFNA<br>PFDA<br>PDUnDA<br>PFDoDA<br>PFTrDA<br>PFTeDA	Examines trends of PFAS concentrations in aquatic birds from 1990/1991 to 2010/2011 (mainly compares before vs after 2000)	Miller et *al.* (2015)
Dulhonice, Topelec, Nespeky, Predmerice,	Freshwater	European chub (*Squalius cephalus*)	Muscle<br>Liver	PFPeA<br>PFHxA<br>PFHpA	Evaluates differences between monitoring	Cerveny et *al.* (2016)

SI-2 Table 2. Overview of geographic and taxonomic coverage of PFASs in the biota.

Valy and Usti nad Labem, Czech Republic				PFHxS<br>PFOA<br>PFNA<br>PFOS	approaches and tries to find a standardized approach for the biomonitoring of aquatic environments	
Rural areas of Sweden	Freshwater<br>Terrestrial	Osprey (*Pandion haliaetus*)<br>Tawny owl (*Strix aluco*)<br>Common kestrel (*Falco tinnunculus*)	Eggs	<u>Detected in >50% of each bird sp. eggs:</u><br>T-PFOS<br>L-PFOS<br>PFNA<br>PFDA<br>PFUnDA<br>PFDoDA<br>PFTrDA<br>PFTeDA<br><u>Other PFASs found :</u><br>PFHxS<br>PFDS<br>PFOA<br>7:3 FTCA<br>5:3 FTCA<br>8:2 FTUCA<br>diPAP homologues<br>PFOSA<br>FTSA	Longer-chained PFCAs (C12–C14) more abundant in the terrestrial avian species compared to osprey (freshwater). The levels of diPAPs were higher in the freshwater environment than the terrestrial environment. The levels of PFOS in the osprey eggs are in the range where adverse effects such as reduced hatchability have been observed.	Eriksson et *al*. (2016)

SI-2 Table 2. Overview of geographic and taxonomic coverage of PFASs in the biota.

Paju (Gyeonggi-do), Gyungsangbuk-do, Jeollabuk-do , Jeollanam-do, Dokdo Island and Ulleung Island, Korea	Terrestrial	Eurasian eagle owl (*Bubo bubo*) Common kestrel (*Falco tinnunculus*) Collared scops owl (*Otus lempiji*)<br>Black-tailed gull (*Larus crassirostris*)<br>Brown hawk owl (*Ninox scutulata*)<br>Northern goshawk (*Accipiter gentilis*)<br>Cinereous vulture (*Aegypius monachus*)<br>Common buzzard (*Buteo buteo*)<br>Spot-billed duck (*Anas Poecilorhyncha*)<br>Oriental turtle dove (*Streptopelia orientalis*)	Liver	PFPeA<br>PFHxA<br>PFHpA<br>PFOA<br>PFNA<br>PFDA<br>PFUnDA<br>PFDoDA<br>PFTrDA<br>PFTeDA<br>PFHxDA<br>PFOcDA<br>PDBS<br>PFHxS<br>PFOS<br>PFDS	PFAS were detected in all of the liver tissues, indicating ubiquitous contamination in Korean environments. Longer-chained PFAS such as PFUnDA, PFDoDA, and PFTrDA were dominant in predatory birds, while shorter-chained PFAS such as PFOA and PFPeDA were dominant in non-predatory birds. This phenomenon could be attributed to the differences in potentials of biomagnification for PFASs.	Bargi et *al*. (2018)
Western shores of the Scheldt river, west of Antwerp, Belgium	Terrestrial	Great tit (*Parus major*)<br>Northern lapwing (*Vanellus vanellus*)<br>Mediterranean gull (*Larus melanocephalus*)	Eggs<br>Blood	PFOS	This study in the Antwerp harbour region reports some of the highest PFOS levels ever measured in wildlife	Lopez-Antia et *al*. (2017)

SI-2 Table 2. Overview of geographic and taxonomic coverage of PFASs in the biota.

Midway Island, North Pacific Ocean	Marine	Black-footed albatross (*Phoebastria nigripes*)	Muscle<br>Liver<br>Adipose tissues	PFBA<br>PFPeA<br>PFHxA<br>PFHpA<br>PFOA<br>PFEtCHxS<br>PFNA<br>PFDA<br>PFUnDA<br>PFDoDA<br>PFTrDA<br>PFTeDA<br>PFHxDA<br>PFODA<br>PFBS<br>PFHxS<br>PFOS<br>PFDS	Great Pacific Garbage Patch (GPGP) as a PFAS contamination source (PFAS in plastics would be a direct source of exposure and accumulation in Albatrosses)	Chu et *al*. (2015)
Clarks Marsh, Oscoda, Michigan, USA	Terrestrial	Tree swallow (*Tachycineta bicolor*)	Eggs<br>Plasma<br>Liver<br>Brain	PFBA<br>PFPeA<br>PFHxA<br>PFHpA<br>PFOA<br>PFNA<br>PFDA<br>PFUnDA<br>PFDoDA<br>PFBS<br>PFHxS<br>PFOS	Diet also analyzed for PFAS.<br>Brain tissue had the least number of PFASs detected.	Custer et *al*. (2019)

SI-2 Table 2. Overview of geographic and taxonomic coverage of PFASs in the biota.

				PFOSA		
Northern Norway	Terrestrial	White-tailed eagle (*Haliaeetus albicilla*)<br>Northern goshawk (*Accipiter gentilis*)	Plasma<br>Feathers	PFOSA<br>PFBA<br>PFPeA<br>PFHxA<br>PFHpA<br>PFOA<br>PFNA<br>PFDA<br>PFUnDA<br>PFDoDA<br>PFTrDA<br>PFTeDA<br>PFBS<br>PFHxS<br>Lin-PFOS<br>Br-PFOS<br>PFDcS	Significant associations between specific PFAS in blood plasma and body feathers of white-tailed eagle were found, suggesting that analyzing body feathers of White-tailed eagle could potentially be a useful non-invasive strategy to monitor PFAS exposure in nestlings of this species.	Gómez-Ramírez et *al*. (2017)
Lake á Myranar, Faroe Islands<br>Lake Norðara Hálsavatn, Lake Stóravatn and Lake Lítluvatn, Island of Sandur<br>Southwest Greenland<br>Nuuk, Qeqertarsuaq and Ittoqqortoormiit, Greenland	Terrestrial<br>Freshwater	Ptarmigan (*Lagopus muta*)<br>Reindeer (*Rangifer tarandus*)<br>Muskox (*Ovibos moschatus*)<br>Landlocked Arctic char (*Salvelinus alpinus*)<br>Trout (*Salmo trutta*)	Liver<br>Muscle	PFBS<br>PFHxS<br>PFHpS<br>PFOS<br>PFDS<br>PFOSA<br>PFPeA<br>PFHxA<br>PFHpA<br>PFOA<br>PFNA<br>PFDA<br>PFUnDA	Analyses of terrestrial mammals	Bossi et *al*. (2015)

SI-2 Table 2. Overview of geographic and taxonomic coverage of PFASs in the biota.

				PFDoDA<br>PFTrDA<br>PFTeDA		
Pearl River Delta, South China Sea	Marine	Indo-Pacific humpback dolphin (*Sousa chinensis*)<br>Finless porpoise (*Neophocaena phocaenoides*)	Liver	PFBA<br>PFPeA<br>PFHxA<br>PFHpA<br>PFOA<br>PFNA<br>PFDA<br>PFUnDA<br>PFDoDA<br>PFTeDA<br>PFBS<br>PFHxS<br>PFOS<br>PFDS	It is likely that current concentrations of PFOS in livers of dolphin and porpoise are sufficient to cause observable adverse effects on some proportion of the cetacean populations studied, based on the TCCs derived.	Lam et *al*. (2016)
San Francisco Bay, United States	Marine	Double-crested cormorant (*Phalacrocorax auritus*)<br>Pacific harbor seals (*Phoca vitulina richardii*)<br>Yellowfin gobies (*Acanthogobius flavimanus*)<br>Chameleon/cheekspot gobies (*Tridentiger trigonocephalus/ Ilypnus gilbert*)	Eggs (*P. auritus*)<br>Blood (*P. vitulina richardii*)<br>Whole body (fish)	PFBS<br>PFHxS<br>PFOS<br>PFBA<br>PFPeA<br>PFHxA<br>PFHpA<br>PFOA<br>PFNA<br>PFDA<br>PFUnDA<br>PFDoDA<br>PFOSA	Concentrations of PFOS in double-crested cormorant eggs and Pacific harbor seals residing in the southern portion of the Bay were among the highest reported globally between 2006 and 2009. The most recent analyses in 2014 (seal blood) and	Sedlak et *al*. (2017)

SI-2 Table 2. Overview of geographic and taxonomic coverage of PFASs in the biota.

		Northern anchovy (*Engraulis mordax*) Shiner surfperch (*Cymatogaster aggregate*) Staghorn sculpin (*Leptocottus armatus*)			2012 (cormorant eggs) revealed that concentrations in the South Bay region (the most contaminated area) have declined approximately 70 percent since the prior sampling events in 2009 (birds) and 2009-2011 (seals), likely reflecting a delayed response to the 2002 phase-out of PFOS and related substances.	
West Ice, east of Greenland	Marine	Hooded seal (*Cystophora cristata*)	Plasma Milk	PFHxS PFOS PFOA PFNA PFDA PFUnDA PFDoDA PFTrDA	The PFAS were detected and quantified in both plasma and milk from hooded seal, documenting for the first time PFAS in seal milk and maternal transfer in mother–pup pairs in marine mammals.	Grønnestad et *al*. (2016)
Ittoqqortoormiit/Scoresby Sound area and	Marine	Ringed seal (*Pusa*	Liver	F-53B PFBS	The killer whale samples included in	Gebbink et *al*. (2016)

SI-2 Table 2. Overview of geographic and taxonomic coverage of PFASs in the biota.

Tasiilaq/Ammassalik area, Greenland		*hispida)*<br>Polar bear (*Ursus maritimus*)<br>Killer whale (*Orcinus orca*)		PFHxS<br>Br-PFOS<br>L-PFOS<br>PFDS<br>Br-FOSA<br>L-FOSA<br>Tot-FOSA<br>PFHpA<br>PFOA<br>PFNA<br>PFDA<br>PFUnDA<br>PFDoDA<br>PFTrDA<br>PFTeDA<br>PFPeDA	this study contained liver samples from a mother-fetus pair. A significant relationship ($p < 0.0001$) was observed between individual PFAS concentrations in mother and fetus, indicating maternal transfer from the mother whale to the fetus.	
McMurdo Sound, Antarctica	Marine	Weddell seal (*Leptonychotes weddellii*)	Blood plasma	Above detection limit in at least one sample:<br>PFUnDA<br>PFOS lin<br>PFHxA<br>PFTriDA<br>Below detection limit:<br>FOSA<br>PFBS<br>PFHxS<br>PFOS br	Detectable PFAS concentrations in a relatively non-migratory marine mammal at such high latitude in Antarctica.	Routti et *al*. (2015)

SI-2 Table 2. Overview of geographic and taxonomic coverage of PFASs in the biota.

				PFDS<br>PFBA<br>PFPA<br>PFHpA<br>PFOA<br>PFNA<br>PFDA<br>PFDoDA<br>PFTeDA		
Hokkaido, Northern Japan	Marine	Dall's porpoises (*Phocoenoides dalli*)<br>Harbor porpoises (*Phocoena phocoena*)	Blood<br>Liver<br>Blubber	<u>More than 65% detection frequency:</u><br>PFOA<br>PFNA<br>PFDA<br>PFUnDA<br>PFDoDA<br>PFTrDA<br>PFTeDA<br>PFOS<br><u>Less than 65% detection frequency:</u><br>PFHxS<br>PFHpS<br>PFDS<br>PFOSA	[PFAS]: liver>blood>blubber. Results suggest that PFOS is the most liver specific of the PFASs measured, and the specificity of PFCA depends on the alkyl chain length.	Fujii et *al*. (2018)
Svalbard, Norway	Marine	Ringed seals (*Pusa hispida*)	Plasma	PFBS<br>PFHxS<br>PFOS<br>PFDS	-	Routti et *al*. (2016)

SI-2 Table 2. Overview of geographic and taxonomic coverage of PFASs in the biota.

				PFBA<br>PFPeA<br>PFHxA<br>PFHpA<br>PFOA<br>PFNA<br>PFDA<br>PFUnDA<br>PFDoDA<br>PFTrDA<br>PFTeDA<br>FOSA		
Faroe Islands	Marine	North Atlantic pilot whale (*Globicephala melas*)	Muscle	PFBS<br>PFHxS<br>PFHpS<br>PFOS<br>PFDS<br>FOSA<br>PFHxA<br>PFHpA<br>PFOA<br>PFNA<br>PFDA<br>PFUnDA<br>PFDoDA<br>PFTrDA<br>PFTeDA	Lack of biotransformation of FOSA by cetaceans provides opportunity to quantify the exposures attributable to this neutral atmospheric precursor.<br>Results imply that changing atmospheric burdens of FOSA exerted a major influence on biological exposures in the Arctic and Subarctic regions.	Dassuncao et *al*. (2017)

SI-2 Table 2. Overview of geographic and taxonomic coverage of PFASs in the biota.

					FOSA levels in pilot whale muscle reported here indicate that precursors are important exposure sources for marine food webs.	

SI-2 Table 2. Overview of geographic and taxonomic coverage of PFASs in the biota.

References

Ahrens, L, Gashaw, H, Sjöholm, M, Gebrehiwot, SG, Getahun, A, Derbe, E, Bishop, K and Åkerblom, S. 2016. Poly- and perfluoroalkylated substances (PFASs) in water, sediment and fish muscle tissue from Lake Tana, Ethiopia and implications for human exposure. Chemosphere, 165, 352-357.

Ahrens, L, Norström, K, Viktor, T, Cousins, AP and Josefsson, S. 2015. Stockholm Arlanda Airport as a source of per- and polyfluoroalkyl substances to water, sediment and fish. Chemosphere, 129, 33-38.

Alves, LMF, Nunes, M, Marchand, P, Le Bizec, B, Mendes, S, Correia, JPS, Lemos, MFL and Novais, SC. 2016. Blue sharks (*Prionace glauca*) as bioindicators of pollution and health in the Atlantic Ocean: Contamination levels and biochemical stress responses. Science of the Total Environment, 563-564, 282-292.

Åkerblom, S, Negm, N, Wu, P, Bishop, k and Ahrens, L. 2017. Variation and accumulation patterns of poly- and perfluoroalkyl substances (PFAS) in European perch (*Perca fluviatilis*) across a gradient of pristine Swedish lakes. Science of the Total Environment, 599-600, 1685-1692.

Babut, M, Labadie, P, Simonnet-Laprade, C, Munoz, G, Roger, MC, Ferrari, BJD, Budzinski, H and Sivade, E. 2017. Per- and poly-fluoroalkyl compounds in freshwater fish from the Rhône River: Influence of fish size, diet, prey contamination and biotransformation. Science of the Total Environment, 605-606, 38-47.

Bargi, M, Jin, X, Lee, S, Jeong, Y, Yu, JP, Paek, WK and Moon HB. 2018. Accumulation and exposure assessment of persistent chlorinated and fluorinated contaminants in Korean birds. Science of the Total Environment, 645, 220-228.

Bossi, R, Dam, M and Rigét FF. Perfluorinated alkyl substances (PFAS) in terrestrial environments in Greenland and Faroe Islands. Chemosphere, 129, 164-169.

Cerveny, D., Grabic, R., Fedorova, G., Grabicova, K., Turek, J., Kodes, V., Golovko, O., Zlabek, V. and Randak, T. 2016. Perfluoroalkyl substances in aquatic environment-comparison of fish and passive sampling approaches. Environmental Research, 144, 92-98.

Chu, S, Letcher, RJ, McGoldrick, DJ and Backus, SM. 2016. A new fluorinated surfactant contaminant in biota: Perfluorobutane sulfonamide in several fish species. Environmental Science and Technology, 50, 669-675.

SI-2 Table 2. Overview of geographic and taxonomic coverage of PFASs in the biota.

Dassuncao, C, Hu, XC, Zhang, X, Bossi, R, Dam, M, Mikkelsen, B and Sunderland, EM. 2017. Temporal Shifts in Poly- and perfluoroalkyl substances (PFASs) in north Atlantic pilot whales indicate large contribution of atmospheric precursors. Environmental Science and Technology, 51, 4512-4521.

D'Hollander, W, De Bruyn, L, Hagenaars, A, de Voogt, P and Bervoets, L. 2014. Characterisation of perfluorooctane sulfonate (PFOS) in a terrestrial ecosystem near a fluorochemical plant in Flanders, Belgium. Environmental Science and Pollution Research, DOI 10.1007/s11356-013-2449-4.

Dalahmeh, S, Tirgani, S, Komakech, A J, Niwagaba, C B and Arhens, L. 2018. Per- and polyfluoroalkyl substances (PFASs) in water, soil and plants in wetlands and agricultural areas in Kampala, Uganda. Science of the Total Environment, 631-632, 660-667.

Eriksson, U, Roos, A, Lind, Y, Hope, K, Ekblad, A and Kärrman, A. 2016. Comparison of PFASs contamination in the freshwater and terrestrial environments by analysis of eggs from osprey (*Pandion haliaetus*), tawny owl (*Strix aluco*), and common kestrel (*Falco tinnunculus*). Environmental Research, 149, 40-47.

Fair, PA, Wolf, B, White, ND, Arnott, SA, Kannan, K, Karthikraj, R and Vena, JE. 2019. Perfluoroalkyl substances (PFASs) in edible fish species from Charleston Harbor and tributaries, South Carolina, United States: Exposure and risk assessment. Environmental Research, 171, 266-277.

Fujii, Y., Sakurada, T., Harada, K.H., Koizumi, A., Kimura, O., Endo, T. and Haraguchi, K. 2015. Long-chain perfluoroalkyl carboxylic acids in Pacific cods from coastal areas in northern Japan: A major source of human dietary exposure. Environmental Pollution, 199, 35-41.

Gebbink, W.A., Bossi, R., Rigét, F.F., Rosing-Asvid, A., Sonne, C. and Dietz, D. 2016. Observation of emerging per- and polyfluoroalkyl substances (PFASs) in Greenland marine mammals. Chemosphere, 144, 2384-2391.

Giari, L, Guerranti, C, Perra, G, Lanzoni, M, Fano, EA and Castaldelli, G. 2015. Occurrence of perfluorooctane sulfonate and perfluorooctanoic acid and histopathology in eels from north Italian waters. Chemosphere, 118, 117-123.

SI-2 Table 2. Overview of geographic and taxonomic coverage of PFASs in the biota.

Gómez-Ramírez, P, Bustnes, JO, Eulaers, I, Herzke, D, Johnsen, TV, Lepoint, G, Pérez-García, JM, García-Fernández, AJ and Jaspers, VLB. 2017. Per- and polyfluoroalkyl substances in plasma and feathers of nestling birds of prey from northern Norway. Environmental Research, 158, 277-285.

Groffen, T, Wepener, V, Malherbe, W and Bervoets, L. 2018. Distribution of perfluorinated compounds (PFASs) in the aquatic environment of the industrially polluted Vaal River, South Africa. Science of the Total Environment, 627, 1334-1344.

Grønnestad, R, Villanger, GD, Polder, A, Kovacs, KM, Lydersen, C, Jenssen, BM and Borgå, K. 2016. Maternal transfer of perfluoroalkyl substances in hooded seals. Environmental Toxicology and Chemistry, 36, 763-770.

Koizumi, A., Kimura, O., Endo, T. and Haraguchi, K. 2018. Tissue-specific bioaccumulation of long-chain perfluorinated carboxylic acids and halogenated methylbipyrroles in Dall's porpoises (*Phocoenoides dalli*) and harbor porpoises (*Phocoena phocoena*) stranded in northern Japan. Science of the Total Environment, 616-617, 554-563.

Lam, JCW, Lyu, J, Kwok, KY and Lam, PKS. 2016. Perfluoroalkyl substances (PFASs) in Marine mammals from the South China Sea and their temporal changes 2002−2014: Concern for alternatives of PFOS? Environmental Science and Technology, 50, 6728-6736.

Lam, NH, Cho, CR, Kannan, K and Cho, HS. 2017. A nationwide survey of perfluorinated alkyl substances in waters, sediment and biota collected from aquatic environment in Vietnam: Distributions and bioconcentration profiles. Journal of Hazardous Materials, 323, 116-127.

Lopez-Antia, A, Dauwe, T, Meyer, J, Maes, K, Bervoets, L and Eens, M. 2017. High levels of PFOS in eggs of three bird species in the neighborhood of a fluoro-chemical plant. Ecotoxicology and Environmental Safety, 139, 165-171.

Lorenzo, M, Campo, J, Farré, M, Pérez, F, Picó, Y and Barceló, D. 2016. Perfluoroalkyl substances in the Ebro and Guadalquivir river basins (Spain). Science of the Total Environment, 540, 191-199.

Miller, A., Elliott, J.E., Elliott, K.H., Lee, S. and Cyr, F. 2015. Temporal trends of perfluoroalkyl substances (PFAS) in eggs of coastal and offshore birds: increasing PFAS levels associated with offshore bird species

SI-2 Table 2. Overview of geographic and taxonomic coverage of PFASs in the biota.

breeding on the Pacific coast of Canada and wintering near Asia. Environmental Toxicology and Chemistry, 34, 1799-1808.

Müller, C E, De Silva, A O, Small, J, Williamson, M, Wang, X, Morris, A, Katz, S, Gamberg, M and Muir, D C G. 2011. Biomagnification of perfluorinated compounds in a remote terrestrial food chain: lichen-caribou-wolf. Environmental Science and Technology, 45, 8665-8673.

Mwakalapa, EB, Mmochi, AJ, Müller, MHB, Mdegela, RH, Lyche, JL and Polder, A. 2018. Occurrence and levels of persistent organic pollutants (POPs) in farmed and wild marine fish from Tanzania. A pilot study. Chemosphere, 191, 438-449.

Pan, CG, Yu, KF, Wang, YH, Zhang, RJ, Huang, XY, Wei, CS, Wang, WQ, Zeng, WB and Qin, ZJ. 2018. Species-specific profiles and risk assessment of perfluoroalkyl substances in coral reef fishes from the South China Sea. Chemosphere, 191, 450-457.

Pignotti, E, Casas, G, Llorca, M, Tellbüscher, A, Almeida, D, Dinelli, E, Farré, M and Barceló, D. 2017. Seasonal variations in the occurrence of perfluoroalkyl substances in water, sediment and fish samples from Ebro Delta (Catalonia, Spain) Science of the Total Environment, 607-608, 933-943.

Routti, H., Krafft, B.A., Herzke, D., Eisert, R. and Oftedal, O. 2015. Perfluoroalkyl substances detected in the world's southernmost marine mammal, the Weddell seal (*Leptonychotes weddellii)*. Environmental Pollution, 197, 62-67.Fujii, Y., Kato, Y., Sakamoto, K., Matsuishi, T., Harada, K. H.,

Routti, H, Gabrielsen, GW, Herzke, D, Kovacs, KM and Lydersen, C. 2016. Spatial and temporal trends in perfluoroalkyl substances (PFASs) in ringed seals (*Pusa hispida*) from Svalbard. Environmental Pollution, 214, 230-238.

Sedlak, MD, Benskin, JP, Wong, A, Grace, R and Greig, DJ. 2017. Per- and polyfluoroalkyl substances (PFASs) in San Francisco Bay wildlife: Temporal trends, exposure pathways, and notable presence of precursor compounds. Chemosphere, 185, 1217-1226.

Squadrone, S, Ciccotelli, V, Prearo, M, Favaro, L, Scanzio, T, Foglini, C and Abete, MC. 2015. Perfluorooctane sulfonate (PFOS) and perfluorooctanoic acid (PFOA): emerging contaminants of increasing concern in fish from Lake Varese, Italy. Environmental Monitoring and Assessment, 187, 438.

SI-2 Table 2. Overview of geographic and taxonomic coverage of PFASs in the biota.

Taylor, MD and Johnson, DD. 2016. Preliminary investigation of perfluoroalkyl substances in exploited fishes of two contaminated estuaries. Marine Pollution Bulletin, 111, 509-513.

Taylor, MD, Beyer-Robson, J, Johnson, DD, Knott, NA and Bowles, KC. 2018. Bioaccumulation of perfluoroalkyl substances in exploited fish and crustaceans: Spatial trends across two estuarine systems. Marine Pollution Bulletin, 131, 303-313.

Zafeiraki, E, Gebbink, WA, Van Leeuwen, SPJ, Dassenakis, E and Megalofonou, P. 2019. Occurrence and tissue distribution of perfluoroalkyl substances (PFASs) in sharks and rays from the eastern Mediterranean Sea. Environmental Pollution, 252, 379-387.
