# Supplementary material for: Assessing the Ecological Risks of Per‐ and Polyfluoroalkyl Substances: Current State‐of‐the Science and a Proposed Path Forward
Source: Environ Toxicol Chem. 2020 Nov 6;40(3):564–605. doi: 10.1002/etc.4869 (PMC7984443; doi:10.1002/etc.4869)
Supplement: Supplementary file 3 — Supporting information. [file ETC-40-564-s003.pdf]

SUPPORTING INFORMATION

I. ECOTOXICOLOGICAL DATA MINING METHODS

A. Invertebrates (aquatic and terrestrial)

Ecotoxicological data for invertebrates were extracted from US EPA ECOTOX Knowledgebase (last access November 2019). All effects (except accumulation), all endpoints and all invertebrate species (crustacean, insect/spider, molluscs, worms and other invertebrates) were selected for all the compounds included in the PFAS chemical class. The output fields were customised and the final database have been subject to verification. Data were checked for redundancies and errors. Two additional fields (as columns) were added to the final tables: *1)* “PFAS acronyms” for gathering the acid and its corresponding salts of the same chemical and *2)* the number of fluorinated carbons for each PFAS.

Tables SV4 and SI-SV6 list the whole toxicological data for PFAS on aquatic and terrestrial invertebrates. Effect concentrations of all the aquatic ecotoxicological data are converted to the standard unit mg/L. When possible, effect concentrations of the terrestrial ecotoxicological data were converted to standard units (µM or mg/kg). The fields "Organism Lifestage", "Endpoint" and "Test Type" “Concentration Standardized “ of some records of the aquatic database were modified. The original classification (larva, not coded, not reported....) is reported in parentheses.

All codes for fields of the tables are located in the ECOTOX Code Appendix, available from a link in the ECOTOX Help page (https://cfpub.epa.gov/ecotox//pdf/codeappendix.pdf). Unless cited in the manuscript, references of the studies are only reported in the excel tables.

Table SI-3.1. Ecotoxicological data (n of records and of publications) of PFAS for aquatic and terrestrial invertebrates mined from US EPA ECOTOX Knowledgebase (accessed November 2019).

Acronym	Chemical Name	Records	Publications	Year Min	Year Max	Records %
**Fluorotelomers FTx**						5
6:2 FTI	1H,1H,2H,2H-Perfluorooctyl iodide	1	1	2016	2016	0
8(14):2 FTI	gamma-omega-Perfluoroalkyl iodides C8-C14	2	1	1994	1994	0
5:1 FTOH	2,2,3,3,4,4,5,5-Octafluoro-1-pentanol	12	1	2012	2012	0
4:2 FTOH	3,3,4,4,5,5,6,6,6-Nonafluoro-1-hexanol	6	1	2010	2010	0
6:2 FTOH	3,3,4,4,5,5,6,6,7,7,8,8,8-Tridecafluoro-1-octanol	7	2	2010	2016	0
8:2 FTOH	3,3,4,4,5,5,6,6,7,7,8,8,9,9,10,10,10-Heptadecafluoro-1-decanol	4	2	2010	2016	0
10:2 FTOH	3,3,4,4,5,5,6,6,7,7,8,8,9,9,10,10,11,11,12,12,12-Heneicosafluoro-1-dodecanol	6	2	2010	2017	0
5:3 FTCA	2H,2H,3H,3H-Undecafluoro octanoic acid	1	1	2012	2012	0
7:3 FTCA	4,4,5,5,6,6,7,7,8,8,9,9,10,10,10-Pentadecafluorodecanoic acid	2	1	2012	2012	0
4:2 FTCA	3,3,4,4,5,5,6,6,6-Nonafluorohexanoic acid	12	1	2007	2007	0
6:2 FTCA	3,3,4,4,5,5,6,6,7,7,8,8,8-Tridecafluorooctanoic acid	19	3	2007	2012	0
8:2 FTCA	3,3,4,4,5,5,6,6,7,7,8,8,9,9,10,10,10-Heptadecafluorodecanoic acid	34	4	2006	2012	1
10:2 FTCA	3,3,4,4,5,5,6,6,7,7,8,8,9,9,10,10,11,11,12,12,12-Heneicosafluorododecanoic acid	27	3	2006	2011	1
4:2 FTUCA	3,4,4,5,5,6,6,6-Octafluoro-2-hexenoic acid	12	1	2007	2007	0
6:2 FTUCA	3,4,4,5,5,6,6,7,7,8,8,8-Dodecafluoro-2-octenoic acid	18	3	2007	2012	0
8:2 FTUCA	3,4,4,5,5,6,6,7,7,8,8,9,9,10,10,10-Hexadecafluoro-2-decenoic acid	18	3	2007	2012	0
10:2 FTUCA	3,4,4,5,5,6,6,7,7,8,8,9,9,10,10,11,11,12,12,12-Eicosafluoro-2-dodecenoic acid	25	3	2006	2011	1
6:2 FTS	3,3,4,4,5,5,6,6,7,7,8,8,8-Tridecafluoro-1-octanesulfonic acid	5	3	2006	2015	0
6:2 FTMAC	2-Methyl-2-propenoic acid , 3,3,4,4,5,5,6,6,7,7,8,8,8-tridecafluorooctyl ester	1	1	2016	2016	0
**Perfluoroalkyl carboxylic acids PFCA**						30
TFA	Trifluoroacetic acid	3	1	2014	2014	0
PFPrA	Perfluoropropionic acid	9	2	2002	2014	0
PFBA	Perfluorobutanoic acid	28	4	2002	2015	1
PFPeA	Perfluoropentanoic acid	12	4	2002	2014	0
PFHxA	Perfluorohexanoic acid	18	4	2006	2016	0
PFOdA	Dodecafluorosuberic acid	1	1	2006	2006	0
PFHpA	Perfluoroheptanoic acid	17	4	2002	2018	0
PFOA	Perfluorooctanoic acid	943	44	2002	2019	21
PFNA	Perfluorononanoic acid	145	12	2002	2018	3
PFDA	Perfluorodecanoic acid	84	11	2002	2018	2
PFDdA	Perfluorodecanedioic acid	1	1	2006	2006	0
PFUnA	Perfluoroundecanoic acid	41	5	2006	2016	1
PFDoDA	Perfluorododecanoic acid	28	2	2012	2016	1
**Perfluoroalkyl sulfonic acids PFSA**						47
PFBS	Perfluorobutane sulfonic acid	312	8	2006	2019	7
PFHxS	Perfluorohexane sulfonic acid	10	3	2006	2018	0
PFECHS	Perfluoro-4-ethylcyclohexanesulfonic acid	21	1	2016	2016	0
PFOS	Perfluorooctane sulfonic acid	1718	80	1992	2019	39
PFDS	Perfluorodecane sulfonic acid	2	1	2006	2006	0
**FluoroCyclohexanes**						0
C7F14	Perfluoromethylcyclohexane	2	1	1970	1970	0
C6F11H	Undecafluorocyclohexane	1	1	1970	1970	0
C6F12	Dodecafluorocyclohexane	1	1	1970	1970	0
**Perfluoroalkyl Sulfonamides FASA**						0
FOSA	Perfluorooctane sulfonamide	2	2	2016	2018	0
N-Et-FOSE	N-Ethylperfluorooctanesulfonamidoethanol	3	1	2016	2016	0
FOSAPrTMA I	[3-(1,1,2,2,3,3,4,4,5,5,6,6,7,7,8,8,8-Heptadecafluoro-1-octanesulfonamido)propyl]trimethylammonium iodide	2	1	2006	2006	0
**Fluorinated Plant Protection Products (PPP)**						18
Sulfluramid (N-Et-FOSA)	N-Ethyl-1,1,2,2,3,3,4,4,5,5,6,6,7,7,8,8,8-heptadecafluoro-1-octanesulfonamide	388	37	1990	2018	9
Hexaflumuron	N-[[[3,5-Dichloro-4-(1,1,2,2-tetrafluoroethoxy)phenyl]amino]carbonyl]-2,6-difluorobenzamide	247	32	1988	2015	6

Lufenuron	N-[[[2,5-Dichloro-4-(1,1,2,3,3,3-hexafluoropropoxy)phenyl]amino]carbonyl]-2,6-difluorobenzamide	72	16	2002	2015	2
Flubendiamide	N2-[1,1-Dimethyl-2-(methylsulfonyl)ethyl]-3-iodo-N1-[2-methyl-4-[1,2,2,2-tetrafluoro-1-(trifluoromethyl)ethyl]phenyl]-1,2-benzenedicarboxamide	69	9	1992	2018	2

Pareto chart of the ecotoxicological records for invertebrates (aquatic and terrestrial) exposed to 47 different PFAS (ECOTOX Knowledgebase EPA). Red line is the cumulative percentage of the total number of records.

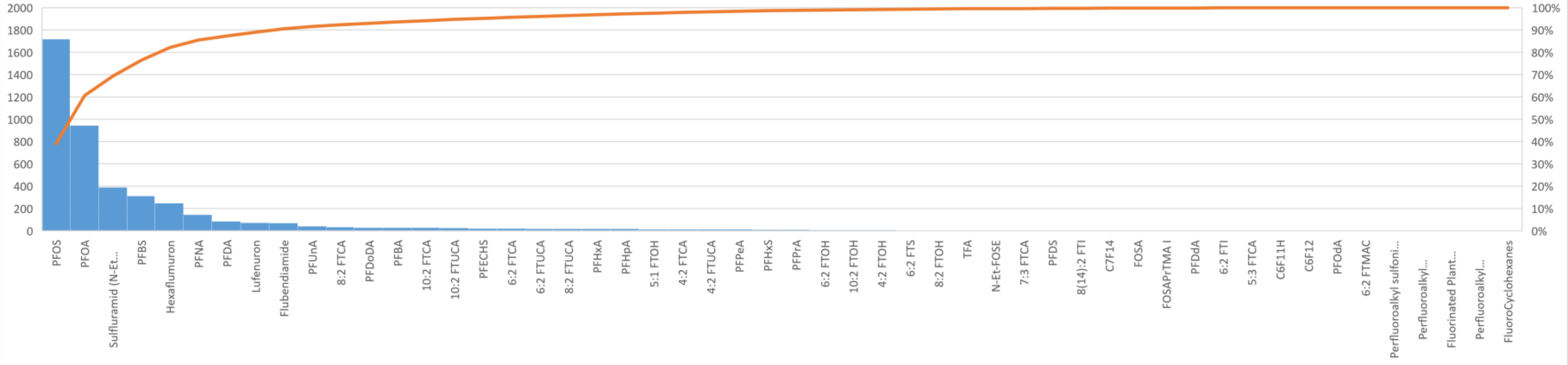

Pareto chart of the ecotoxicological publications for invertebrates (aquatic and terrestrial) exposed to 47 different PFAS (ECOTOX Knowledgebase EPA). Red line is the cumulative percentage of the total number of records.

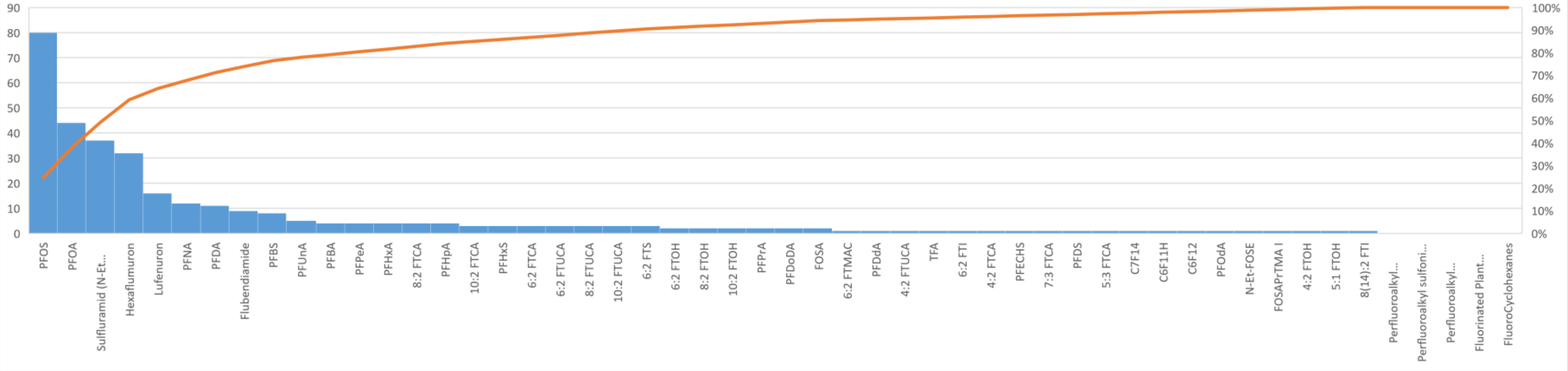

Table SI-3.2a Overview of aquatic invertebrates that were tested with Perfluoroalkyl carboxylic acids (PFCA)

Compound	Number of fluorinated carbons	Protozoa	Rotifera	Crustacea						Echinodermata	Mollusca		Insecta				Worm		
		Ciliate		Copepoda	Isopoda	Mysida	Decapoda (Shrimp, Crab)	Amphipoda	Water Flea		Bivalvia	Gastropoda	Chironomids	Damselfly/Mayfly	Hemiptera	Mosquito	Platyhelminthes	Annelida	Nematoda
**Perfluoroalkyl carboxylic acids PFCA**																			
TFA	1		X																
PFPrA	2		X (STS)						X (STS)										
PFBA	3		X (STS)						X (STS)										
PFPeA	4		X (STS)						X (STS)		X								
PFHxA	5		X (STS)						X (STS)		X								
PFOdA	6	X																	
PFHpA	6								X (STS)		X								
PFOA	7	X	X (STS)	X	X	X	X		X (STS)	X (STS)	X (STS)	X	X (STS)				X	X	
PFDdA	8	X																	
PFNA	8	X							X (STS)		X		X						
PFDA	9	X							X (STS)		X (STS)		X						
PFUnA	10								X (STS)		X (STS)		X						
PFDoDA	11								X (STS)				X						
*(STS) Standard Test Specie		*Paramecium caudatum*	*Asplanchna brightwell*<br>*Brachionus angularis*<br>*Brachionus calyciflorus (STS)*<br>*Brachionus quadridentatus*<br>*Cephalodella sp.*<br>*Filinia longiseta*<br>*Polyarthra vulgaris*<br>*Synchaeta sp.*<br>*Trichocerca sp.*	*Acanthocyclops sp.*<br>*Cyclops sp.*<br>*Diacyclops sp.*	*Proasellus sp.*	*(s) Siriella armata*	*Macrobrachium nipponense*<br>*Neocaridina denticulata*		*Bosmina longirostris*<br>*Ceriodaphnia sp.*<br>*Chydorus sphaericus*<br>*Daphnia ambigua*<br>*Daphnia magna (STS)*<br>*Daphnia pulex*<br>*Daphnia pulicaria*<br>*Moina macrocopa*<br>*Moinodaphnia sp.*<br>*Simocephalus vetulus*	*(s) Paracentrotus lividus (STS)*	*(s) Crassostrea gigas (STS)*<br>*Lampsilis siliquoidea*<br>*Ligumia recta*<br>*(s) Mytilus californianus*<br>*(s) Mytilus galloprovincialis (STS)*<br>*(s) Mytilus viridis*	*Physella acuta*<br>*Cipangopaludina cathayensis*	*Chironomus plumosus*<br>*Chironomus riparius (STS)*<br>*Chironomus tentans (STS)*				*Dugesia japonica*	*Limnodrilus hoffmeisteri*	

Table SI-3.2b Overview of aquatic invertebrates that were tested with PFSA

**Compound**	**Number of fluorinated carbons**	**Protozoa**	**Rotifera**	**Crustacea**						**Echinodermata**	**Mollusca**		**Insecta**				**Worm**		
		Ciliate		Copepoda	Isopoda	Mysida	Decapoda (Shrimp, Crab)	Amphipoda	Water Flea		Bivalvia	Gastropoda	Chironomids	Damselfly/Mayfly	Hemiptera	Mosquito	Platyhelminthes	Annelida	Nematoda
**Perfluoroalkyl sulfonic acids PFSA**																			
PFBS	4	X									X		X (STS)						
PFHxS	6										X								
PFOS	8		X (STS)	X (STS)		X (STS)	X	X	X (STS)	X (STS)	X (STS)	X	X (STS)	X			X	X	
PFECHS	8								X (STS)										
PFDS	10										X								
*(STS) Standard Test Specie		*Paramecium caudatum*	*Brachionus calyciflorus (STS)*	*Cyclops sp.*<br>*(s) Tigriopus japonicus (STS)*		*(s) Americamysis bahia (STS)*<br>*(s) Siriella armata*	*Eriocheir sinensis*<br>*(s) Macrophthalmus japonicus*<br>*Macrobrachium nipponense*<br>*Neocaridina denticulata*	*Monoporeia affinis*	*Daphnia magna (STS)*<br>*Daphnia pulicaria*<br>*Moina macrocopa*	*(s) Glyptocidaris crenularis*<br>*(s) Paracentrotus lividus (STS)*<br>*(s) Psammechinus miliaris*	*(s) Crassostrea gigas (STS)*<br>*(s) Crassostrea virginica (STS)*<br>*(s) Mytilus californianus*<br>*(s) Mytilus galloprovincialis (STS)*<br>*(s) Mytilus viridis*<br>*(s) Perna viridis*<br>*Elliptio complanata*<br>*Lampsilis siliquoidea*<br>*Ligumia recta*<br>*Unio ravoisieri*	*Cipangopaludina cathayensis*<br>*Physella acuta*	*Chironomus plumosus*<br>*Chironomus riparius (STS)*<br>*Chironomus tentans (STS)*	*Enallagma cyathigerum*			*Dugesia japonica*	*Limnodrilus hoffmeisteri*<br>*(s) Perinereis wilsoni*	

Table SI-3.2c Overview of aquatic invertebrates that were tested with FTx, FASA and FluoroCyclohexanes

Compound	Number of fluorinated carbons	Protozoa	Rotifera	Crustacea						Echinodermata	Mollusca		Insecta				Worm		
		Ciliate		Copepoda	Isopoda	Mysida	Decapoda (Shrimp, Crab)	Amphipoda	Water Flea		Bivalvia	Gastropoda	Chironomids	Damselfly/Mayfly	Hemiptera	Mosquito	Platyhelminthes	Annelida	Nematoda
**Fluorotelomers FTx**																			
6:2 FTI	6																		
8(14):2 FTI	8(14)	X (STS)																	
4:2 FTOH	4	X (STS)																	
5:1 FTOH	5								X (STS)										
6:2 FTOH	6	X (STS)																	
8:2 FTOH	8	X (STS)																	
10:2 FTOH	10	X (STS)																	
4:2 FTCA	4								X (STS)				X (STS)						
5:3 FTCA	5								X (STS)										
6:2 FTCA	6							X (STS)	X (STS)				X (STS)						
7:3 FTCA	7								X (STS)										
8:2 FTCA	8							X (STS)	X (STS)				X (STS)						
10:2 FTCA	10							X (STS)	X (STS)				X (STS)						
4:2 FTUCA	4								X (STS)				X (STS)						
6:2 FTUCA	6							X (STS)	X (STS)				X (STS)						
8:2 FTUCA	8							X (STS)	X (STS)				X (STS)						
10:2 FTUCA	10							X (STS)	X (STS)				X (STS)						
6:2 FTS	6	X																	
6:2 FTMAC	6																		
**Perfluoroalkyl Sulfonamides FASA**																			
FOSA	8																		
N-Et-FOSE	8																		
FOSAPrTMA I	8	X																	
**FluoroCyclohexanes**																			
C6F12	6											X							
C6F11H	6											X							
C7F14	7											X							
*(STS) Standard Test Specie		*Paramecium caudatum*<br>*Tetrahymena thermophila (STS,*						*Hyalella azteca (STS)*	*Daphnia magna (STS,*<br>*Chydorus sphaericus*			*Biomphalaria glabrata*	*Chironomus tentans (STS)*						

Table SI-3.2d Overview of aquatic invertebrates that were tested with Fluorinated PPP

**Compound**	**Number of fluorinated carbons**	**Protozoa**	**Rotifera**	**Crustacea**						**Echinodermata**	**Mollusca**		**Insecta**				**Worm**		
		Ciliate		Copepoda	Isopoda	Mysida	Decapoda (Shrimp, Crab)	Amphipoda	Water Flea		Bivalvia	Gastropoda	Chironomids	Damselfly/Mayfly	Hemiptera	Mosquito	Platyhelminthes	Annelida	Nematoda
**Fluorinated Plant Protection Products PPP**																			
Flubendiamide	3					X (STS)		X (STS)	X (STS)		X (STS)		X (STS)	X					
Hexaflumuron	4						X (STS)				X (STS)				X	X (STS)			X
Lufenuron	5															X (STS)			
Sulfluramid (N-Et-FOSA)	8			X					X (STS)										
*(STS) Standard Test Specie				*(s) Lepeophtheirus salmonis*		*(s) Americamysis bahia (STS)*	*(s) Penaeus aztecus (STS)*<br>*(s) Crangon crangon*	*Hyalella azteca (STS)*	*Daphnia magna (STS)*		*(s) Crassostrea virginica (STS)*		*Chironomus tentans (STS)*<br>*Chironomus riparius (STS)*	*Neocloeon triangulifer*	*Anisops bouvieri*<br>*Diplonychus sp.*	*Anopheles stephensi*<br>*Culex peus*<br>*Culex tarsalis*<br>*Toxorhynchites splendens*<br>*Culex quinquefasciatus*<br>*Aedes aegypti (STS)*<br>*Aedes albopictus*			*Romanomermis iyengari*

Table SI-3.3a Overview of aquatic invertebrates that were tested with PFCA

Compound	Number of fluorinated carbons	Insecta											Entognatha	Arachnida	Worm	
		Blattodea	Blattodea Termites	Diptera	Coleoptera	Hemiptera	Hymenoptera Ants	Hymenoptera Bees	Hymenoptera Wasps	Lepidoptera	Neuroptera	Rhynchota	Springtail		Annelida Earthworm	Nematoda
**Perfluoroalkyl carboxylic acids PFCA**																
TFA	1															
PFPrA	2															
PFBA	3															
PFPeA	4															
PFHxA	5															X (STS)
PFOdA	6															
PFHpA	6														X (STS)	X (STS)
PFOA	7			X											X (STS)	X (STS)
PFDdA	8															
PFNA	8														X (STS)	X (STS)
PFDA	9															X (STS)
PFUnA	10															X (STS)
PFDoDA	11															
*(STS) Standard Test Specie				*Drosophila melanogaster*											*Aporrectodea caliginosa*<br>*Eisenia fetida (STS)*	*Caenorhabditis elegans (STS)*

Table SI-3.3b Overview of aquatic invertebrates that were tested with PFSA

**Compound**	**Number of fluorinated carbons**	**Insecta**											**Entognatha**	**Arachnida**	**Worm**	
		Blattodea	Blattodea Termites	Diptera	Coleoptera	Hemiptera	Hymenoptera Ants	Hymenoptera Bees	Hymenoptera Wasps	Lepidoptera	Neuroptera	Rhynchota	Springtail		Annelida Earthworm	Nematoda
**Perfluoroalkyl sulfonic acids PFSA**																
PFBS	4														X (STS)	X (STS)
PFHxS	6														X (STS)	X (STS)
PFOS	8	X		X				X (STS)					X (STS)	X	X (STS)	X (STS)
PFECHS	8															
PFDS	10															
*(STS) Standard Test Specie		*Blattella germanica*		*Drosophila hydei*				*Apis mellifera (STS)*<br>*Bombus terrestris*					*Folsomia candida (STS)*	*Oppia nitens*	*Aporrectodea caliginosa*<br>*Eisenia fetida (STS)*<br>*Eisenia andrei (STS)*	*Caenorhabditis elegans (STS)*

Table SI-3.3c Overview of aquatic invertebrates that were tested with FTx and FASA

**Compound**	**Number of fluorinated carbons**	**Insecta**											**Invertebrates**	**Arachnida**	**Worm**	
		Blattodea	Blattodea Termites	Diptera	Coleoptera	Hemiptera	Hymenoptera Ants	Hymenoptera Bees	Hymenoptera Wasps	Lepidoptera	Neuroptera	Rhynchota	Entognatha Springtail		Annelida Earthworm	Nematoda
**Fluorotelomers FTx**																
6:2 FTI	6															X (STS)
8(14):2 FTI	8(14)															
4:2 FTOH	4															
5:1 FTOH	5															
6:2 FTOH	6															X (STS)
8:2 FTOH	8															X (STS)
10:2 FTOH	10														X (STS)	
4:2 FTCA	4															
5:3 FTCA	5															
6:2 FTCA	6															
7:3 FTCA	7															
8:2 FTCA	8															
10:2 FTCA	10															
4:2 FTUCA	4															
6:2 FTUCA	6															
8:2 FTUCA	8															
10:2 FTUCA	10															
6:2 FTS	6															
6:2 FTMAC	6															X (STS)
**Perfluoroalkyl Sulfonamides FASA**																
FOSA	8														X (STS)	X (STS)
N-Et-FOSE	8														X (STS)	
FOSAPrTMA I	8															
**FluoroCyclohexanes**																
C6F12	6															
C6F11H	6															
C7F14	7															
*(STS) Standard Test Specie															*Eisenia fetida (STS)*	*Caenorhabditis elegans (STS)*

Table SI-3.3d Overview of aquatic invertebrates that were tested with Fluorinated PPP

**Compound**	**Number of fluorinated carbons**	**Insecta**											**Entognatha**	**Arachnida**	**Worm**	
		Blattodea	Blattodea Termites	Diptera	Coleoptera	Hemiptera	Hymenoptera Ants	Hymenoptera Bees	Hymenoptera Wasps	Lepidoptera	Neuroptera	Rhynchota	Springtail		Annelida Earthworm	Nematoda
**Fluorinated Plant Protection Products (PPP)**																
Flubendiamide	3				X	X		X (STS)	X	X	X	X		X		
Hexaflumuron	4		X		X	X		X (STS)	X	X			X (STS)	X	X (STS)	
Lufenuron	5				X	X	X	X	X	X	X			X	X (STS)	
Sulfluramid (N-Et-FOSA)	8	X	X				X		X						X (STS)	X (STS)
*(STS) Standard Test Specie		*Blattella germanica*	*Coptotermes acinaciformis*		*Cheilomenes sp.*	*Amrasca biguttula ssp. biguttula*	*Atta bisphaerica*	*Apis mellifera (STS)*	*Cotesia vestalis*	*Helicoverpa armigera*	*Chrysoperla sp.*	*Aleyrodidae*	*Folsomia candida (STS)*	*Arachnida*	*Eisenia fetida (STS)*	*Caenorhabditis elegans (STS)*
		Periplaneta americana	*Coptotermes lacteus*		*Leptinotarsa decemlineata*	*Adelphocoris lineolatus*	*Atta sexdens ssp. rubropilosa*	*Bombus terrestris*	*Trichogramma pretiosum*	*Plutella xylostella*				*Araniella opisthographa*		
		Periplaneta fuliginosa	*Coptotermes acinaciformis*		*Cheilomenes sp.*	*Laodelphax striatellus*	*Atta texana*		*Trissolcus basalis*	*Striacosta albicosta*				*Clubiona neglecta*		
			Coptotermes formosanus		*Coccinellidae*	*Bemisia taboci*	*Camponotus pennsylvanicus*		*Anagrus nilaparvatae*	*Cydia pomonella*				*Dictyna uncinata*		
			Coptotermes lacteus		*Leptinotarsa decemlineata*		*Ectatomma brunneum*		*Eulophus pennicornis*	*Helicoverpa armigera*				*Pardosa agrestis*		
			Reticulitermes chinensis				*Linepithema humile*		*Habrobracon hebetor*	*Mamestra brassicae*				*Philodromus cespitum*		
			Reticulitermes flavipes				*Solenopsis invicta*		*Leiophron spreta*	*Spodoptera exigua*				*Theridion impressum*		
									Trichogramma chilonis	Helicoverpa armigera				Arachnida		
									Trichogramma evanescens	Maruca vitrata						
									Trichogramma japonicum	Pandemis heparana						
									Trichogramma nubilale	Spodoptera litura						
									Trichogramma ostriniae	Spodoptera sp.						
									Cotesia vestalis							
									Neochrysocharis formosa							
									Neochrysocharis okazakii							
									Telenomus podisi							
									Trichogramma pretiosum							
									Trissolcus basalis							
									Vespula vulgaris							

		**Table SI-3.5a Minimum and maximum values (50% lethal concentration/50% effect concentration [LC50/EC50] mg/L) of the acute toxicity (Mortality or Intoxication) of Perfluoroalkyl and polyfluoroalkyl substances (PFAS) on aquatic invertebrates**					**Table SI-3.5b Minimum and maximum values (lowest observed effect concentration [LOEC] mg/l or 10% effect concentration/no observed effect concentration [EC10/NOEC] if LOEC is not available) of the chronic toxicity (Development, Growth and Reproduction) of Perfluoroalkyl and polyfluoroalkyl substances (PFAS) on aquatic invertebrates.**			
**Acronym**	**Number of fluorinated carbons**	**EC50/LC50 Intoxication, Mortality**					**LOEC Development, Growth and Reproduction**			
		**min**		**max**			**min**		**max**	
**Fluorotelomers FTx**										
6:2 FTI	6									
8(14):2 FTI	8(14)									
4:2 FTOH	4									
5:1 FTOH	5	195	neonate of Chydorus sphaericus (48h) (Ding etal. 2012)	323	neonate of Chydorus sphaericus (24h) (Ding etal. 2012)					
6:2 FTOH	6									
8:2 FTOH	8									
10:2 FTOH	10									
4:2 FTCA	4		>100	neonate of Daphnia magna (48h) (Phillips et al. 2007)/ larva of Chironomus tentans (10d) (Phillips et al. 2007)				>100 (EC10)	growth of larva of Chironomus tentans (10d) (Phillips et al. 2007)	
5:3 FTCA	5		>103	neonate of Daphnia magna (48h) (Hoke et al. 2012)						
6:2 FTCA	6	33	Hyalella azteca (10d) (Mitchell et al., 2011)	>100	neonate of Daphnia magna (48h) (Phillips et al. 2007)			36.5 (EC10)	growth of larva of Chironomus tentans (10d) (Phillips et al. 2007)	
7:3 FTCA	7	0.4	Daphnia magna (48h) (Hoke et al. 2015)	2.3	Daphnia magna (48h) (Hoke et al. 2015)					
8:2 FTCA	8	2.3	neonate of Daphnia magna (48h) (Hoke et al. 2012)	12.4	larva of Chironomus tentans (10d) (Phillips et al. 2007)		0.19 (EC10)	Development of neonate of Chironomus tentants (60d) (MacDonald 2006)	0.47 (EC10)	Growth of neonate of Chironomus tentants (20d) (MacDonald 2006)
10:2 FTCA	10	0.03	neonate of Daphnia magna (48h) (Phillips et al. 2007)	>16.3	larva of Chironomus tentans (10d) (Phillips et al. 2007)			0.03 (EC10)	reproduction of Daphnia magna (21d) (MacDonald 2006)	
4:2 FTUCA	4		>100	neonate of Daphnia magna (48h) (Phillips et al. 2007)/ larva of Chironomus tentans (10d) (Phillips et al. 2007)				>100 (EC10)	growth of larva of Chironomus tentans (10d) (Phillips et al. 2007)	
6:2 FTUCA	6	30	neonate of Daphnia magna (48h) (Hoke et al. 2012)	>100	neonate of Daphnia magna (48h) (Phillips et al. 2007)/ larva of Chironomus tentans (10d) (Phillips et al. 2007)					
8:2 FTUCA	8	3.2	neonate of Daphnia magna (48h) (Hoke et al. 2012)	21	larva of Chironomus tentans (10d) (Phillips et al. 2007)			7.38 (EC10)	growth of larva of Chironomus tentans (10d) (Phillips et al. 2007)	
10:2 FTUCA	10	0.28	neonate of Daphnia magna (48h) (Phillips et al. 2007)	>8.4	larva of Chironomus tentans (10d) (Phillips et al. 2007)			0.12 (EC10)	reproduction of Daphnia magna (21d) (MacDonald 2006)	
6:2 FTS	6		>109	Daphnia magna (48h) (Hoke et al. 2015)						
6:2 FTMAC	6									

		**Table SI-3.5a Minimum and maximum values (50% lethal concentration/50% effect concentration [LC50/EC50] mg/L) of the acute toxicity (Mortality or Intoxication) of Perfluoroalkyl and polyfluoroalkyl substances (PFAS) on aquatic invertebrates**					**Table SI-3.5b Minimum and maximum values (lowest observed effect concentration [LOEC] mg/l or 10% effect concentration/no observed effect concentration [EC10/NOEC] if LOEC is not available) of the chronic toxicity (Development, Growth and Reproduction) of Perfluoroalkyl and polyfluoroalkyl substances (PFAS) on aquatic invertebrates.**			
**Acronym**	**Number of fluorinated carbons**	**EC50/LC50 Intoxication, Mortality**					**LOEC Development, Growth and Reproduction**			
		**min**		**max**			**min**		**max**	
**Perfluoroalkyl carboxylic acids PFCA**										
TFA	1		70	neonate of Brachionus calyciflorus (24h) (Wang et a. 2014)				1 (NOEC)	reproduction Brachionus calyciflorus (72h) (Wang et a. 2014)	
PFPrA	2	80	neonate of Brachionus calyciflorus (24h) (Wang et a. 2014)	>1000	Daphnia magna and Daphnia pulicaria (48h) (Boudreau 2002)			1 (NOEC)	reproduction Brachionus calyciflorus (72h) (Wang et a. 2014)	
PFBA	3	110	neonate of Brachionus calyciflorus (24h) (Wang et a. 2014)	5251	neonate of Daphnia magna (48h) (Barmentlo et al. 2015)			1	reproduction Brachionus calyciflorus (72h) (Wang et a. 2014)	
PFPeA	4	130	neonate of Brachionus calyciflorus (24h) (Wang et a. 2014)	>1000	Daphnia magna and Daphnia pulicaria (48h) (Boudreau 2002)			1	reproduction Brachionus calyciflorus (72h) (Wang et a. 2014)	
PFHxA	5	140	neonate of Brachionus calyciflorus (24h) (Wang et a. 2014)	1048	neonate of Daphnia magna (48h) (Barmentlo et al. 2015)		1	reproduction Brachionus calyciflorus (72h) (Wang et a. 2014)	737 (EC10)	reproduction of Daphnia magna (21d) (Barmentlo et al. 2015)
PFOdA	5									
PFHpA	6		>1000	Daphnia pulicaria (48h) (Boudreau 2002)						
PFOA	7	16	(s) neonate of Siriella armata (96h) (Mhadhbi et . 2012)	>1000	Neocaridina denticulata (24h) (Li 2009)		0.0001	Abnormal development of Embryo of (s) Mytilus galloprovincialis (48h) (Fabbri et al. 2014)	100	reproduction of Daphnia magna (21d) (Li 2010)
PFNA	8	11	neonate of Daphnia magna (48h) (Lu et al. 2015)	223	neonate of Daphnia magna (24h) (Ding etal. 2012)		0.040	growth and reproduction of neonate of Daphnia magna (21d) (Lu et al. 2015)	50	Reproduction of Daphnia magna (21d) (Boudreau 2002)
PFDdA	8									
PFDA	9	45	neonate of Chydorus sphaericus (48h) (Ding etal. 2012)	285	Daphnia pulicaria (48h) (Boudreau 2002)					
PFUnA	10	19	neonate of Chydorus sphaericus (48h) (Ding etal. 2012)	134	neonate of Daphnia magna (24h) (Ding etal. 2012)					
PFDoDA	11	28	neonate of Chydorus sphaericus (48h) (Ding etal. 2012)	99	neonate of Chydorus sphaericus (24h) (Ding etal. 2012)					
**Perfluoroalkyl sulfonic acids PFSA**										
PFBS	4							0.008	Development, growth and reproduction of neonate of Chironomus riparius (lifetime) (Stefani et al. 2014, Marziali et al. 2019)	
PFHxS	6									
PFECHS	6		187	neonate of Daphnia magna (48h) (Huode et al. 2016)						

		**Table SI-3.5a Minimum and maximum values (50% lethal concentration/50% effect concentration [LC50/EC50] mg/L) of the acute toxicity (Mortality or Intoxication) of Perfluoroalkyl and polyfluoroalkyl substances (PFAS) on aquatic invertebrates**					**Table SI-3.5b Minimum and maximum values (lowest observed effect concentration [LOEC] mg/l or 10% effect concentration/no observed effect concentration [EC10/NOEC] if LOEC is not available) of the chronic toxicity (Development, Growth and Reproduction) of Perfluoroalkyl and polyfluoroalkyl substances (PFAS) on aquatic invertebrates.**			
**Acronym**	**Number of fluorinated carbons**	**EC50/LC50 Intoxication, Mortality**					**LOEC Development, Growth and Reproduction**			
		**min**		**max**			**min**		**max**	
PFOS	8	3.6	(s) Americamysis bahia (96h) (Drottar 2000)	271	Physella acuta (24h) (Li 2009)		0.0001	Abnormal development of Embryo of (s) Mytilus galloprovincialis (48h) (Fabbri et al. 2014)	50	reproduction of Daphnia magna (21d) (Boudreau et al. 2003)
PFDS	10									
**FluoroCyclohexanes**										
C6F11H	6		>10	Adult Biomphalaria glabrata (24h) (Duncan et al. 1970)						
C6F12	6		>10	Adult Biomphalaria glabrata (24h) (Duncan et al. 1970)						
C7F14	7		>20	Adult Biomphalaria glabrata (24h) (Duncan et al. 1970)						
**Perfluoroalkyl Sulfonamides FASA**										
FOSA	8									
N-Et-FOSE	8									
FOSAPrTMA I	8									
**Fluorinated Plant Protection Products (PPP)**										
Flubendiamide	3	0.0013	Daphnia magna (48h) (US EPA, 1992)	>28	(s) Americamysis bahia (96h) (US EPA, 1992)		0.001	Hatch of Daphnia magna (21d) (US EPA, 1992)	0.07	Progeny numbers of Daphnia magna (21d) (US EPA, 1992)
Hexaflumuron	4	0.0001	Daphnia magna (48h) (US EPA, 1992)	>2.3	Crangon crangon (96h) (US EPA, 1992)		0.00004	reproduction of Culex quinquefasciatus (Vasuki 1997)	0.001	emergence of larva of Culex quinquefasciatus (Vasukiet al.1997)
Lufenuron	5	1.8	larva of Aedes aegypti (72h) (Ahmed et al. 2015)	6.5	larva of Aedes aegypti (24h) (Ahmed et al. 2015)					
Sulfluramid (N	8	0.37	Daphnia magna (48h) (US EPA, 1992)	>2	Daphnia magna (48h) (US EPA, 1992)					

Table SI-3.7a Studies on alteration of the antioxidant defenSe system in invertebrates exposed to PFAS: minimun LOEC/LOEL values per species. From US EPA ECOTOX Knowledgebase (https://cfpub.epa.gov/ecotox/; accessed November 2019).

Effect	Compound	Species	Endpoint		unit	Effect Measurement	remark	Author	Reference Number	Title	Source	Publication Year
Lipid peroxidation	PFOS	*Unio ravoisieri*	7d-LOEC	2-6	mg/L	Malondialdehyde	Digestive gland, Gill(s)	Amraoui,I., N. Khalloufi, and S. Touaylia	177085	Effects to Perfluorooctane Sulfonate (PFOS) on the Mollusk Unio ravoisieri Under Laboratory Exposure	Chem. Ecol.34(4): 324-339	2018
		Limnodrilus hoffmeisteri	24h-LOEC	0.6	mg/L	Malondialdehyde		Liu,J., R. Qu, L. Yan, L. Wang, and Z. Wang	177071	Evaluation of Single and Joint Toxicity of Perfluorooctane Sulfonate and Zinc to Limnodrilus hoffmeisteri: Acute Toxicity, Bioaccumulation and Oxidative Stress	J. Hazard. Mater.301:342-349	2016
		Eisenia fetida (t)	7d-LOEL	10	mg/kg soil	Malondialdehyde		Xu,D., C. Li, Y. Wen, and W. Liu	166647	Antioxidant Defense System Responses and DNA Damage of Earthworms Exposed to Perfluorooctane Sulfonate (PFOS)	Environ. Pollut.174:121-127	2013
	PFOA	*Dugesia japonica*	24h-LOEC	0.5	mg/L	Malondialdehyde	dose dependent	Yuan,Z., Z. Miao, X. Gong, B. Zhao, Y. Zhang, H. Ma, J. Zhang, and B. Zhao	177044	Changes on Lipid Peroxidation, Enzymatic Activities and Gene Expression in Planarian (Dugesia japonica) Following Exposure to Perfluorooctanoic Acid	Ecotoxicol. Environ. Saf.145:564-568	2017
		Eisenia fetida (t)	7d-LOEL	10	mg/kg soil	Malondialdehyde		Zhao,Y., G. Li, D. Qi, L. Sun, C. Wen, and S. Yin	177161	Biomarker Responses of Earthworms (Eisenia fetida) to Soils Contaminated with Perfluorooctanoic Acid	Environ. Sci. Pollut. Res. Int.24(27): 22073-22081	2017
Reactive Oxygen Species (ROS)	PFOS	*Limnodrilus hoffmeisteri*	7d-LOEC	1	mg/L	Reactive oxygen species		Meng,L., S. Yang, M. Feng, R. Qu, Y. Li, J. Liu, Z. Wang, and C. Sun	177070	Toxicity and Bioaccumulation of Copper in Limnodrilus hoffmeisteri Under Different pH Values: Impacts of Perfluorooctane Sulfonate	J. Hazard. Mater.305:219-228	2016
		(s) Tigriopus japonicus	24h-LOEC	0.5	mg/L	Reactive oxygen species	dose dependent	Han,J., E.J. Won, M.C. Lee, J.S. Seo, S.J. Lee, and J.S. Lee	175656	Developmental Retardation, Reduced Fecundity, and Modulated Expression of the Defensome in the Intertidal Copepod Tigriopus japonicus Exposed to BDE-47 and PFOS	Aquat. Toxicol.165:136-143	2015
Antioxidant enzyme activities	PFBS	*Caenorhabditis elegans (t)*	2d-LOEL	450	mg/L	Catalase (CAT), Glutathione peroxidase (GPx)		Chen,F., C. Wei, Q. Chen, J. Zhang, L. Wang, Z. Zhou, M. Chen, and Y. Liang	178025	Internal Concentrations of Perfluorobutane Sulfonate (PFBS) Comparable to Those of Perfluorooctane Sulfonate (PFOS) Induce Reproductive Toxicity in Caenorhabditis elegans	Ecotoxicol. Environ. Saf.158:223-229	2018
	PFOS	*Dugesia japonica*	24h-LOEC	0.5	mg/L	Superoxide dismutase (SOD)		Yuan,Z., J. Zhang, W. Meng, and Y. Zhou	175659	Effects of Perfluorooctane Sulfonate on Behavioural Activity, Regeneration and Antioxidant Enzymes in Planarian Dugesia japonica	Chem. Ecol.30(2): 187-195	2014
		Eriocheir sinensis	14d-LOEC	10	mg/L	Superoxide dismutase (SOD)		Zhang,F., J. Wei, Q. Li, R. Jiang, N. Yu, J. Qin, and L. Chen	175661	Effects of Perfluorooctane Sulfonate on the Immune Responses and Expression of Immune-Related Genes in Chinese Mitten-Handed Crab Eriocheir sinensis	Comp. Biochem. Physiol. C Toxicol. Pharmacol.172/173:13-18	2015
		(s) Glyptocidaris crenularis	72h-LOEC	0.01	mg/L	Catalase (CAT)	Coelomic fluid	Ding,G., L. Wang, J. Zhang, Y. Wei, L. Wei, Y. Li, M. Shao, and D. Xiong	175705	Toxicity and DNA Methylation Changes Induced by Perfluorooctane Sulfonate (PFOS) in Sea Urchin Glyptocidaris crenularis	Chemosphere128:225-230	2015
		Limnodrilus hoffmeisteri	48h-LOEC	0.2	mg/L	Superoxide dismutase (SOD)		Qu,R., J. Liu, L. Wang, and Z. Wang	175703	The Toxic Effect and Bioaccumulation in Aquatic Oligochaete Limnodrilus hoffmeisteri After Combined Exposure to Cadmium and Perfluorooctane Sulfonate at Different pH Values	Chemosphere152:496-502	2016
		Unio ravoisieri	7d-LOEC	2	mg/L	Superoxide dismutase (SOD)	Digestive gland, Gill(s)	Amraoui,I., N. Khalloufi, and S. Touaylia	177085	Effects to Perfluorooctane Sulfonate (PFOS) on the Mollusk Unio ravoisieri Under Laboratory Exposure	Chem. Ecol.34(4): 324-339	2018
		Eisenia fetida (t)	3d-LOEL	12.5	mg/kg soil	Superoxide dismutase (SOD)		Yuan,Z., J. Zhang, L. Zhao, J. Li, and H. Liu	177158	Effects of Perfluorooctanoic Acid and Perfluorooctane Sulfonate on Acute Toxicity, Superoxide Dismutase, and Cellulase Activity in the Earthworm Eisenia fetida	Environ. Sci. Pollut. Res. Int.24(22): 18188-18194	2017
		Caenorhabditis elegans (t)	2d-LOEL	1	mg/L	Catalase (CAT)		Chen,F., C. Wei, Q. Chen, J. Zhang, L. Wang, Z. Zhou, M. Chen, and Y. Liang	178025	Internal Concentrations of Perfluorobutane Sulfonate (PFBS) Comparable to Those of Perfluorooctane Sulfonate (PFOS) Induce Reproductive Toxicity in Caenorhabditis elegans	Ecotoxicol. Environ. Saf.158:223-229	2018
		(s) Perna viridis	7d-LOEC	0.11	mg/L	Catalase (CAT)		Liu,C., K.Y.H. Gin, and V.W.C. Chang	177196	Multi-Biomarker Responses in Green Mussels Exposed to PFCs: Effects at Molecular, Cellular, and Physiological Levels	Environ. Sci. Pollut. Res.21:2785-2794	2014
		Daphnia magna	7d-LOEC	0.04	mg/L	Superoxide dismutase (SOD)		Lu,G., J. Liu, L. Sun, and L. Yuan	177104	Toxicity of Perfluorononanoic Acid and Perfluorooctane Sulfonate to Daphnia magna	Water Sci. Eng.8(1): 40-48	2015
	PFOA	*Dugesia japonica*	24h-LOEC	0.5	mg/L	Glutathione S-transferase (GST)	dose dependent	Yuan,Z., Z. Miao, X. Gong, B. Zhao, Y. Zhang, H. Ma, J. Zhang, and B. Zhao	177044	Changes on Lipid Peroxidation, Enzymatic Activities and Gene Expression in Planarian (Dugesia japonica) Following Exposure to Perfluorooctanoic Acid	Ecotoxicol. Environ. Saf.145:564-568	2017
		Eisenia fetida (t)	14d-LOEL	5	mg/kg soil	Catalase, Glutathione S-transferase (GST), Peroxidase activity, Superoxide dismutase (SOD)		Zhao,Y., G. Li, D. Qi, L. Sun, C. Wen, and S. Yin	177161	Biomarker Responses of Earthworms (Eisenia fetida) to Soils Contaminated with Perfluorooctanoic Acid	Environ. Sci. Pollut. Res. Int.24(27): 22073-22081	2017
		(s) Perna viridis	7d-LOEC	0.10	mg/L	Catalase (CAT)		Liu,C., K.Y.H. Gin, and V.W.C. Chang	177196	Multi-Biomarker Responses in Green Mussels Exposed to PFCs: Effects at Molecular, Cellular, and Physiological Levels	Environ. Sci. Pollut. Res.21:2785-2794	2014
	PFNA	*Daphnia magna*	7d-LOEC	0.04	mg/L	Catalase (CAT)	dose dependent	Lu,G., J. Liu, L. Sun, and L. Yuan	177104	Toxicity of Perfluorononanoic Acid and Perfluorooctane Sulfonate to Daphnia magna	Water Sci. Eng.8(1): 40-48	2015
		(s) Perna viridis	7d-LOEC	1.07	mg/L	Glutathione peroxidase (GPx)		Liu,C., V.W.C. Chang, and K.Y.H. Gin	175762	Oxidative Toxicity of Perfluorinated Chemicals in Green Mussel and Bioaccumulation Factor Dependent Quantitative Structure-Activity Relationship	Environ. Toxicol. Chem.33(10): 2323-2332	2014
	PFDA	*(s) Perna viridis*	7d-LOEC	0.98	mg/L	Glutathione peroxidase (GPx)		Liu,C., V.W.C. Chang, and K.Y.H. Gin	175762	Oxidative Toxicity of Perfluorinated Chemicals in Green Mussel and Bioaccumulation Factor Dependent Quantitative Structure-Activity Relationship	Environ. Toxicol. Chem.33(10): 2323-2332	2014
	N-Et-FOSE	*Eisenia fetida (t)*	4d-LOEL	0.55	mg/kg soil	Catalase (CAT), Peroxidase activity, Superoxide dismutase (SOD)		Zhao,S., T. Liu, B. Wang, J. Fu, T. Liang, Z. Zhong, J. Zhan, and L. Liu	179674	Accumulation, Biodegradation and Toxicological Effects of N-Ethyl Perfluorooctane Sulfonamidoethanol on the Earthworms Eisenia fetida Exposed to Quartz Sands	Ecotoxicol. Environ. Saf.181:138-145	2019
Regulation of gene involved in oxidative stress	PFBS	*Caenorhabditis elegans (t)*	48h-LOEL	300	mg/L	Catalase-2 mRNA, Glutathione S-transferase 4 mRNA, Superoxide dismutase [Mn] 2 mRNA		Chen,F., C. Wei, Q. Chen, J. Zhang, L. Wang, Z. Zhou, M. Chen, and Y. Liang	178025	Internal Concentrations of Perfluorobutane Sulfonate (PFBS) Comparable to Those of Perfluorooctane Sulfonate (PFOS) Induce Reproductive Toxicity in Caenorhabditis elegans	Ecotoxicol. Environ. Saf.158:223-229	2018
	PFOS	*(s) Tigriopus japonicas*	6h-LOEC	1	mg/L	Glutathione S-transferase sigma class mRNA, p53 mRNA		Han,J., E.J. Won, M.C. Lee, J.S. Seo, S.J. Lee, and J.S. Lee	175656	Developmental Retardation, Reduced Fecundity, and Modulated Expression of the Defensome in the Intertidal Copepod Tigriopus japonicus Exposed to BDE-47 and PFOS	Aquat. Toxicol.165:136-143	2015
		Caenorhabditis elegans (t)	48h-LOEL	1	mg/L	Catalase-2 mRNA, Glutathione S-transferase 4 mRNA, Superoxide dismutase [Mn] 2 mRNA		Chen,F., C. Wei, Q. Chen, J. Zhang, L. Wang, Z. Zhou, M. Chen, and Y. Liang	178025	Internal Concentrations of Perfluorobutane Sulfonate (PFBS) Comparable to Those of Perfluorooctane Sulfonate (PFOS) Induce Reproductive Toxicity in Caenorhabditis elegans	Ecotoxicol. Environ. Saf.158:223-229	2018
		(s) Macrophthalmus japonicus	24h-LOEC	0.001	mg/L	Superoxide dismutase 1 mRNA, Catalase mRNA, Phospholipid glutathione peroxidase mRNA	Liver	Park,K., C. Nikapitiya, T.S. Kwak, and I.S. Kwak	177086	Antioxidative-Related Genes Expression Following Perfluorooctane Sulfonate (PFOS) Exposure in the Intertidal Mud Crab, Macrophthalmus japonicus	Ocean Sci. J.50(3): 547-556	2015
	PFOA	*Dugesia japonica*	24h-LOEC	0.5	mg/L	HSP70 mRNA	dose dependent	Yuan,Z., Z. Miao, X. Gong, B. Zhao, Y. Zhang, H. Ma, J. Zhang, and B. Zhao	177044	Changes on Lipid Peroxidation, Enzymatic Activities and Gene Expression in Planarian (Dugesia japonica) Following Exposure to Perfluorooctanoic Acid	Ecotoxicol. Environ. Saf.145:564-568	2017

Table SI-3.7b Studies on alteration of genotoxicity in invertebrates exposed to PFAS: minimun LOEC/LOEL values per species. From US EPA ECOTOX Knowledgebase (https://cfpub.epa.gov/ecotox/; accessed November 2019).

Effect	Compound	Species	Endpoint		unit	Effect Measurement	remark	Author	Reference Number	Title	Source	Publication Year
Permanent DNA damage (Chromosomal breaks and mitosis dysfunction)	PFOS	*(s) Mytilus galloprovincialis*	30d-LOEC	2	mg/L	Micronucleus assay	Gill(s)	Nalbantlar,B., and O.C. Arslan	177105	Determination of the Perflorooctane Sulfonate-Induced Genotoxic Response in Mytilus galloprovincialis Using a Micronucleus Assay	Zool. Ecol.27(2): 161-167	2017
	PFOS	*(s) Perna viridis*	7d-LOEC	0.096	mg/L	Micronucleus assay	Hemolymph	Liu,C., V.W.C. Chang, K.Y.H. Gin, and V.T. Nguyen	175715	Genotoxicity of Perfluorinated Chemicals (PFCs) to the Green Mussel (Perna viridis)	Sci. Total Environ.487:117-122	2014
	PFOA	*(s) Perna viridis*	7d-LOEC	0.950	mg/L	Micronucleus assay	Hemolymph	Liu,C., V.W.C. Chang, K.Y.H. Gin, and V.T. Nguyen	175715	Genotoxicity of Perfluorinated Chemicals (PFCs) to the Green Mussel (Perna viridis)	Sci. Total Environ.487:117-122	2014
	PFNA	*(s) Perna viridis*	7d-LOEC	0.097	mg/L	Micronucleus assay	Hemolymph	Liu,C., V.W.C. Chang, K.Y.H. Gin, and V.T. Nguyen	175715	Genotoxicity of Perfluorinated Chemicals (PFCs) to the Green Mussel (Perna viridis)	Sci. Total Environ.487:117-122	2014
	PFDA	*(s) Perna viridis*	7d-LOEC	0.099	mg/L	Micronucleus assay	Hemolymph	Liu,C., V.W.C. Chang, K.Y.H. Gin, and V.T. Nguyen	175715	Genotoxicity of Perfluorinated Chemicals (PFCs) to the Green Mussel (Perna viridis)	Sci. Total Environ.487:117-122	2014
DNA damage (fragmentation)	PFBS	*Caenorhabditis elegans (t)*	48h-LOEL	150	mg/L	Germ-cell apoptosis	Gonad(s)	Chen,F., C. Wei, Q. Chen, J. Zhang, L. Wang, Z. Zhou, M. Chen, and Y. Liang	178025	Internal Concentrations of Perfluorobutane Sulfonate (PFBS) Comparable to Those of Perfluorooctane Sulfonate (PFOS) Induce Reproductive Toxicity in Caenorhabditis elegans	Ecotoxicol. Environ. Saf.158:223-229	2018
	PFOS	*Eisenia fetida (t)*	48h-LOEL	0.250	mg/Kg	Alkaline comet assay		Xu,D., C. Li, Y. Wen, and W. Liu	166647	Antioxidant Defense System Responses and DNA Damage of Earthworms Exposed to Perfluorooctane Sulfonate (PFOS)	Environ. Pollut.174:121-127	2013
	PFOS	*(s) Perna viridis*	7d-LOEC	0.096	mg/L	Comet assay (Comet tail moment)	Hemolymph	Liu,C., V.W.C. Chang, K.Y.H. Gin, and V.T. Nguyen	175715	Genotoxicity of Perfluorinated Chemicals (PFCs) to the Green Mussel (Perna viridis)	Sci. Total Environ.487:117-122	2014
	PFOS	*Dugesia japonica*	72h-LOEC	5	mg/L	Comet Assay		Shao,X., B. Zhao, B. Wang, B. Zhao, Y. Zhu, Z. Yuan, and J. Zhang	178849	Neuroprotective Effects of Blueberry Anthocyanins Against Perfluorooctanoic Sulfonate on Planarian Dugesia japonica	Ecotoxicol. Environ. Saf.175:39-47	2019
	PFOS	*Caenorhabditis elegans (t)*	48h-LOEL	0.05	mg/L	Germ-cell apoptosis	Gonad(s)	Chen,F., C. Wei, Q. Chen, J. Zhang, L. Wang, Z. Zhou, M. Chen, and Y. Liang	178025	Internal Concentrations of Perfluorobutane Sulfonate (PFBS) Comparable to Those of Perfluorooctane Sulfonate (PFOS) Induce Reproductive Toxicity in Caenorhabditis elegans	Ecotoxicol. Environ. Saf.158:223-229	2018
	PFOA	*Eisenia fetida (t)*	15d-LOEL	50	mg/kg dry wt	Comet assay (Olive tail moment)	Coelomocytes	Zheng,X.Q., Y.J. Shi, Y.L. Lu, and X.B. Xu	176944	Growth Inhibition and DNA Damage in the Earthworm (Eisenia fetida) Exposed to Perfluorooctane Sulphonate and Perfluorooctanoic Acid	Chem. Ecol.32(2): 103-116	2016
	PFOA	*(s) Perna viridis*	7d-LOEC	0.950	mg/L	Comet assay (Comet tail moment)	Hemolymph	Liu,C., V.W.C. Chang, K.Y.H. Gin, and V.T. Nguyen	175715	Genotoxicity of Perfluorinated Chemicals (PFCs) to the Green Mussel (Perna viridis)	Sci. Total Environ.487:117-122	2014
	PFNA	*(s) Perna viridis*	7d-LOEC	0.992	mg/L	Comet assay (Comet tail moment)	Hemolymph	Liu,C., V.W.C. Chang, K.Y.H. Gin, and V.T. Nguyen	175715	Genotoxicity of Perfluorinated Chemicals (PFCs) to the Green Mussel (Perna viridis)	Sci. Total Environ.487:117-122	2014
	PFDA	*(s) Perna viridis*	7d-LOEC	0.099	mg/L	Comet assay (Comet tail moment)	Hemolymph	Liu,C., V.W.C. Chang, K.Y.H. Gin, and V.T. Nguyen	175715	Genotoxicity of Perfluorinated Chemicals (PFCs) to the Green Mussel (Perna viridis)	Sci. Total Environ.487:117-122	2014
Apoptosis (programmed cell death)	PFBS	*Caenorhabditis elegans (t)*	24h-LOEL	0.125	mg/L	Acridine orange staining	germ line	Guo,X., Q. Li, J. Shi, L. Shi, B. Li, A. Xu, G. Zhao, and L. Wu	175704	Perfluorooctane Sulfonate Exposure Causes Gonadal Developmental Toxicity in Caenorhabditis elegans Through ROS-Induced DNA Damage	Chemosphere155:115-126	2016
	PFOS	*(s) Perna viridis*	7d-LOEC	0.096	mg/L	DNA diffusion assay	Hemolymph	Liu,C., V.W.C. Chang, K.Y.H. Gin, and V.T. Nguyen	175715	Genotoxicity of Perfluorinated Chemicals (PFCs) to the Green Mussel (Perna viridis)	Sci. Total Environ.487:117-122	2014
	PFOA	*Dugesia japonica*	7d-LOEC	5	mg/L	Acridine orange staining	brain, eye, and parenchyma region	Yuan,Z., J. Zhang, Y. Zhang, H. Zhen, and Y. Sun	177055	The Effect of Perfluorooctanoic Acid on the Planarian Dugesia japonica	Pol. J. Environ. Stud.24(2): 801-807	2015
	PFNA	*(s) Perna viridis*	7d-LOEC	0.097	mg/L	DNA diffusion assay	Hemolymph	Liu,C., V.W.C. Chang, K.Y.H. Gin, and V.T. Nguyen	175715	Genotoxicity of Perfluorinated Chemicals (PFCs) to the Green Mussel (Perna viridis)	Sci. Total Environ.487:117-122	2014
	PFDA	*(s) Perna viridis*	7d-LOEC	0.099	mg/L	DNA diffusion assay	Hemolymph	Liu,C., V.W.C. Chang, K.Y.H. Gin, and V.T. Nguyen	175715	Genotoxicity of Perfluorinated Chemicals (PFCs) to the Green Mussel (Perna viridis)	Sci. Total Environ.487:117-122	2014
	6:2 FTOH	*Tetrahymena thermophila*	24h-LOEC	50	mg/L	Shape of the macronucleus indicating apoptosis	Nuclei	Wang,Z., A. Ud-Daula, S. Fiedler, and K.W. Schramm	164493	Impact of Fluorotelomer Alcohols (FTOH) on the Molecular and Macroscopic Phenotype of Tetrahymena thermophila	Environ. Sci. Pollut. Res.17(1): 154-164	2010
Integrative genotoxicity	PFOS	*(s) Perna viridis*	7d-LOEC	0.010	mg/L	Genetics, general	0.033 mg/L (EC50)	Liu,C., V.W.C. Chang, K.Y.H. Gin, and V.T. Nguyen	175715	Genotoxicity of Perfluorinated Chemicals (PFCs) to the Green Mussel (Perna viridis)	Sci. Total Environ.487:117-122	2014
	PFOA	*(s) Perna viridis*	7d-LOEC	0.950	mg/L	Genetics, general	0.594 mg/L (EC50)	Liu,C., V.W.C. Chang, K.Y.H. Gin, and V.T. Nguyen	175715	Genotoxicity of Perfluorinated Chemicals (PFCs) to the Green Mussel (Perna viridis)	Sci. Total Environ.487:117-122	2014
	PFNA	*(s) Perna viridis*	7d-LOEC	0.992	mg/L	Genetics, general	0.195 mg/L (EC50)	Liu,C., V.W.C. Chang, K.Y.H. Gin, and V.T. Nguyen	175715	Genotoxicity of Perfluorinated Chemicals (PFCs) to the Green Mussel (Perna viridis)	Sci. Total Environ.487:117-122	2014
	PFDA	*(s) Perna viridis*	7d-LOEC	0.099	mg/L	Genetics, general	0.078 mg/L (EC50)	Liu,C., V.W.C. Chang, K.Y.H. Gin, and V.T. Nguyen	175715	Genotoxicity of Perfluorinated Chemicals (PFCs) to the Green Mussel (Perna viridis)	Sci. Total Environ.487:117-122	2014
	PFOA	*Dugesia japonica*	24h-LOEC	15	mg/L	DNA methylation		Zhang,J., B. Wang, B. Zhao, Y. Li, X. Zhao, and Z. Yuan	178340	Blueberry Anthocyanin Alleviate Perfluorooctanoic Acid-Induced Toxicity in Planarian (Dugesia japonica) by Regulating Oxidative Stress Biomarkers, ATP Contents, DNA Methylation and mRNA Expression	Environ. Pollut.245:957-964	2019

Table SI-3.7c Studies on neurotoxicity in invertebrates exposed to PFAS: minimun LOEC/LOEL values per species. From US EPA ECOTOX Knowledgebase (https://cfpub.epa.gov/ecotox/; accessed November 2019).

Effect	Compound	Species	Endpoint		unit	Effect Measurement	remark	Author	Reference Number	Title	Source	Publication Year
Alteration of acetylcholinesterase enzyme activity	PFOS	*Daphnia magna*	7d-LOEC	0.008	mg/L	Acetylcholinesterase	dose dependent	Lu,G., J. Liu, L. Sun, and L. Yuan	177104	Toxicity of Perfluorononanoic Acid and Perfluorooctane Sulfonate to Daphnia magna	Water Sci. Eng.8(1): 40-48	2015
	PFNA	*Daphnia magna*	25d-LOEC	0.04	mg/L	Acetylcholinesterase		Lu,G., J. Liu, L. Sun, and L. Yuan	177104	Toxicity of Perfluorononanoic Acid and Perfluorooctane Sulfonate to Daphnia magna	Water Sci. Eng.8(1): 40-48	2015
	PFOS	*Dugesia japonica*	1d-LOEC	0.5	mg/L	Acetylcholinesterase		Yuan,Z., X. Shao, Z. Miao, B. Zhao, Z. Zheng, and J. Zhang	178033	Perfluorooctane Sulfonate Induced Neurotoxicity Responses Associated with Neural Genes Expression, Neurotransmitter Levels and Acetylcholinesterase Activity in Planarians Dugesia japonica	Chemosphere206:150-156	2018
Neurotransmitter levels	PFOS	*Dugesia japonica*	1d-LOEC	0.5	mg/L	Dopamine		Yuan,Z., X. Shao, Z. Miao, B. Zhao, Z. Zheng, and J. Zhang	178033	Perfluorooctane Sulfonate Induced Neurotoxicity Responses Associated with Neural Genes Expression, Neurotransmitter Levels and Acetylcholinesterase Activity in Planarians Dugesia japonica	Chemosphere206:150-156	2018
Expression of neurodevelopmental related genes	PFOS	*Dugesia japonica*	1d-LOEC	0.5	mg/L	Forkhead transcription factor G mRNOrthodenticle A mRNA; Forkhead box D transcription factor mRNA;		Yuan,Z., X. Shao, Z. Miao, B. Zhao, Z. Zheng, and J. Zhang	178033	Perfluorooctane Sulfonate Induced Neurotoxicity Responses Associated with Neural Genes Expression, Neurotransmitter Levels and Acetylcholinesterase Activity in Planarians Dugesia japonica	Chemosphere206:150-156	2018
	PFOA	*Dugesia japonica*	1d-LOEC	0.5	mg/L	Forkhead box D transcription factor mRNA		Yuan,Z., J. Zhang, B. Zhao, Z. Miao, and X. Wu	176960	Effects of Perfluorooctanoic Acid on Neural Genes Expression and Neuronal Morphology in the Planarian Dugesia japonica	Chem. Ecol.32(6): 575-582	2016
Neuronal morphology defects	PFOS	*Dugesia japonica*	1d-LOEC	0.5	mg/L	Brains with abnormal morphology and smaller cephalic ganglia, which displayed reduced nerve fiber density and brain branches compared to controls. The number of SYNORF-positive neurons were drastically declined in a dose-dependent manner	Central nervous system	Yuan,Z., X. Shao, Z. Miao, B. Zhao, Z. Zheng, and J. Zhang	178033	Perfluorooctane Sulfonate Induced Neurotoxicity Responses Associated with Neural Genes Expression, Neurotransmitter Levels and Acetylcholinesterase Activity in Planarians Dugesia japonica	Chemosphere206:150-156	2018
	PFOA	*Dugesia japonica*	7d-LOEC	0.5	mg/L	Neural regeneration defects with a decreased number of neuronal cells and decreased lateral branches compared to controls		Yuan,Z., J. Zhang, B. Zhao, Z. Miao, and X. Wu	176960	Effects of Perfluorooctanoic Acid on Neural Genes Expression and Neuronal Morphology in the Planarian Dugesia japonica	Chem. Ecol.32(6): 575-582	2016

Table SI-3.7d Studies on alteration of the metabolic profile in invertebrates exposed to PFAS: minimun LOEC/LOEL values per species. From US EPA ECOTOX Knowledgebase (https://cfpub.epa.gov/ecotox/; accessed November 2019).

Effect	Compound	Species	Endpoint		unit	Effect Measurement	remark	Author	Reference Number	Title	Source	Publication Year
Alteration of metabolite concentrations	PFOS	*Daphnia magna*	48h-LOEC	30	mg/L	Alanine, Asparagine, Glucose, Glutamate, Glutamine, Glycine, Leucine, Phenylalanine, Serine, Threonine, Tryptophan, Tyrosine	adult (20 d)	Kovacevic,V., A.J. Simpson, and M.J. Simpson	178338	The Concentration of Dissolved Organic Matter Impacts the Metabolic Response in Daphnia magna Exposed to 17a-Ethynylestradiol and Perfluorooctane Sulfonate	Ecotoxicol. Environ. Saf.170:468-478	2019
		Daphnia magna	48h-LOEC	36	mg/L	Alanine, Arginine, Isoleucine, Leucine, Lysine, Phenylalanine, Tyrosine, Valine	Neonates (<24h)	Wagner,N.D., A.J. Simpson, and M.J. Simpson	174537	Metabolomic Responses to Sublethal Contaminant Exposure in Neonate and Adult Daphnia magna	Environ. Toxicol. Chem.36(4): 938-946	2016
		Daphnia magna	48h-LOEC	36	mg/L	Alanine, Arginine, Asparagine, Glutamic acid, Glutamine, Isoleucine, Leucine, Lysine, Methionine, Phenylalanine, Tyrosine, Valine	Adults (18 d)	Wagner,N.D., A.J. Simpson, and M.J. Simpson	174537	Metabolomic Responses to Sublethal Contaminant Exposure in Neonate and Adult Daphnia magna	Environ. Toxicol. Chem.36(4): 938-946	2016

Table SI-3.7e Studies on alteration of the immune system in invertebrates exposed to PFAS: minimun LOEC/LOEL values per species. From US EPA ECOTOX Knowledgebase (https://cfpub.epa.gov/ecotox/; accessed November 2019).

Effect	Compound	Species	Endpoint		unit	Effect Measurement	remark	Author	Reference Number	Title	Source	Publication Year
Coelomocyte mortality, lysosomal membrane fragility	PFBS	*Eisenia hortensis (t)*	28d-LOEL	0.008	mg/kg soil	Trypan blue assay, NRRT assay		Tallandini L, Bonato M, Corrà F, Dolfato E, Guidolin L, Irato P, Pietropoli E, Santovito G, Vantini A, Valsecchi S.	Not included in ECOTOX	Bioaccumulation Potential and cytological responses of a short -chain PFAS, PFBS, in the sediment dwelling worm Eisenia hortensis (Annelida)	SETAC North America Focused Topic Meeting: Understanding Environmental Risk from Exposure to Per- and Polyfluoroalkyl Substances (PFAS), Durham, North Caroline, USA, August 12-15 2019	2019
Hemocyte; Immune-related enzyme activities; expression of immune-related genes	PFOS	*Eriocheir sinensis*	4d-LOEC	0.100	mg/L	Total hemocyte count, Phenoloxidase, C-type Lectin like mRNA, Prophenoloxidase mRNA	Hemolymph, Hepatopancreas	Zhang,F., J. Wei, Q. Li, R. Jiang, N. Yu, J. Qin, and L. Chen	175661	Effects of Perfluorooctane Sulfonate on the Immune Responses and Expression of Immune-Related Genes in Chinese Mitten-Handed Crab Eriocheir sinensis	Comp. Biochem. Physiol. C Toxicol. Pharmacol.172/173:13-18	2015
Hemocyte, Ability of hemocytes to engulf foreign materials, lysosome membrane staibility	PFOS	*(s) Mytilus viridis*	14d-LOEC	0.106	mg/L	Cell Viability, Phagocytosis, Neutral red uptake	Hemolymph	Liu,C., and K.Y. Gin	177106	Immunotoxicity in Green Mussels Under Perfluoroalkyl Substance (PFAS) Exposure: Reversible Response and Response Model Development	Environ. Toxicol. Chem.37(4): 1138-1145	2018
Hemocyte	PFOA	*(s) Mytilus viridis*	14d-LOEC	0.099	mg/L	Cell Viability	Hemolymph	Liu,C., and K.Y. Gin	177106	Immunotoxicity in Green Mussels Under Perfluoroalkyl Substance (PFAS) Exposure: Reversible Response and Response Model Development	Environ. Toxicol. Chem.37(4): 1138-1145	2018
Hemocyte, Ability of hemocytes to kill foreign cells, lysosome membrane staibility	PFNA	*(s) Mytilus viridis*	14d-LOEC	0.117	mg/L	Cell Viability, Cytotoxicity, Neutral red uptake	Hemolymph	Liu,C., and K.Y. Gin	177106	Immunotoxicity in Green Mussels Under Perfluoroalkyl Substance (PFAS) Exposure: Reversible Response and Response Model Development	Environ. Toxicol. Chem.37(4): 1138-1145	2018
Ability of hemocytes to kill foreign cells	PFDA	*(s) Mytilus viridis*	14d-LOEC	0.010	mg/L	Cytotoxicity	Hemolymph	Liu,C., and K.Y. Gin	177106	Immunotoxicity in Green Mussels Under Perfluoroalkyl Substance (PFAS) Exposure: Reversible Response and Response Model Development	Environ. Toxicol. Chem.37(4): 1138-1145	2018
Alteration of multixenobiotic resistance (MXR) mechanism	PFHxS	*(s) Mytilus californianus*	1.5h-LOEC	21.96	mg/L	Xenobiotic-transporting ATPase	Gill(s)	Stevenson,C.N., L.A. MacManus-Spencer, T. Luckenbach, R.G. Luthy, and D. Epel	175712	New Perspectives on Perfluorochemical Ecotoxicology: Inhibition and Induction	Environ. Sci. Technol.40(17): 5580-5585	2006
	PFOA	*(s) Mytilus californianus*	1.5h-LOEC	20.70	mg/L	Xenobiotic-transporting ATPase	Gill(s)	Stevenson,C.N., L.A. MacManus-Spencer, T. Luckenbach, R.G. Luthy, and D. Epel	175712	New Perspectives on Perfluorochemical Ecotoxicology: Inhibition and Induction	Environ. Sci. Technol.40(17): 5580-5585	2006
	PFNA	*(s) Mytilus californianus*	1.5h-IC50	2.23	mg/L	Xenobiotic-transporting ATPase	Gill(s)	Stevenson,C.N., L.A. MacManus-Spencer, T. Luckenbach, R.G. Luthy, and D. Epel	175712	New Perspectives on Perfluorochemical Ecotoxicology: Inhibition and Induction	Environ. Sci. Technol.40(17): 5580-5585	2006
	PFDA	*(s) Mytilus californianus*	1.5h-IC50	3.65	mg/L	Xenobiotic-transporting ATPase	Gill(s)	Stevenson,C.N., L.A. MacManus-Spencer, T. Luckenbach, R.G. Luthy, and D. Epel	175712	New Perspectives on Perfluorochemical Ecotoxicology: Inhibition and Induction	Environ. Sci. Technol.40(17): 5580-5585	2006

Table SI-3.7f Studies on endocrine disruption effects in invertebrates exposed to PFAS: minimun LOEC/LOEL values per species. From US EPA ECOTOX Knowledgebase (https://cfpub.epa.gov/ecotox/; accessed November 2019).

Effect	Compound	Species	Endpoint		unit	Effect Measurement	remark	Author	Reference Number	Title	Source	Publication Year
expression of VTG-related genes	PFBS	*Caenorhabditis elegans (t)*	48h-LOEL	150	mg/L	Vitellogenin 6 mRNA		Chen,F., C. Wei, Q. Chen, J. Zhang, L. Wang, Z. Zhou, M. Chen, and Y. Liang	178025	Internal Concentrations of Perfluorobutane Sulfonate (PFBS) Comparable to Those of Perfluorooctane Sulfonate (PFOS) Induce Reproductive Toxicity in Caenorhabditis elegans	Ecotoxicol. Environ. Saf.158:223-229	2018
	PFECHS	*Daphnia magna*	12d-LOEC	0.6	mg/L	Vitellogenin 1 mRNA		Houde,M., M. Douville, M. Giraudo, K. Jean, M. Lepine, C. Spencer, and A.O. De Silva	175700	Endocrine-Disruption Potential of Perfluoroethylcyclohexane Sulfonate (PFECHS) in Chronically Exposed Daphnia magna	Environ. Pollut.218:950-956	2016
	PFOS	*Caenorhabditis elegans (t)*	48h-LOEL	0.05	mg/L	Vitellogenin 6 mRNA		Chen,F., C. Wei, Q. Chen, J. Zhang, L. Wang, Z. Zhou, M. Chen, and Y. Liang	178025	Internal Concentrations of Perfluorobutane Sulfonate (PFBS) Comparable to Those of Perfluorooctane Sulfonate (PFOS) Induce Reproductive Toxicity in Caenorhabditis elegans	Ecotoxicol. Environ. Saf.158:223-229	2018
VTG content	PFECHS	*Daphnia magna*	12d-LOEC	0.06	mg/L	VTG content		Houde,M., M. Douville, M. Giraudo, K. Jean, M. Lepine, C. Spencer, and A.O. De Silva	175700	Endocrine-Disruption Potential of Perfluoroethylcyclohexane Sulfonate (PFECHS) in Chronically Exposed Daphnia magna	Environ. Pollut.218:950-956	2016

Table SI-3.9. Summary of PFAS studied in fish.

CAS Number	Acronymn	Group	Number of Entries	Chemical Name
53826134	10:2 FTCA	FTx	15	3,3,4,4,5,5,6,6,7,7,8,8,9,9,10,10,11,11,12,12,12-Heneicosafluorododecanoic acid
53826123	6:2 FTCA	FTx	62	3,3,4,4,5,5,6,6,7,7,8,8,8-Tridecafluorooctanoic acid
647427	6:2 FTOH	FTx	110	3,3,4,4,5,5,6,6,7,7,8,8,8-Tridecafluoro-1-octanol
27619972	6:2 FTS	FTx	4	3,3,4,4,5,5,6,6,7,7,8,8,8-Tridecafluoro-1-octanesulfonic acid
812704	7:3 FTCA	FTx	7	4,4,5,5,6,6,7,7,8,8,9,9,10,10,10-Pentadecafluorodecanoic acid
27905459	8:2 FTAc	FTx	4	3,3,4,4,5,5,6,6,7,7,8,8,9,9,10,10,10-Heptadecafluorodecyl ester 2-propenoic acid
27854315	8:2 FTCA	FTx	6	3,3,4,4,5,5,6,6,7,7,8,8,9,9,10,10,10-Heptadecafluorodecanoic acid
678397	8:2 FTOH	FTx	67	3,3,4,4,5,5,6,6,7,7,8,8,9,9,10,10,10-Heptadecafluoro-1-decano
70887842	8:2 FTUCA	FTx	6	3,4,4,5,5,6,6,7,7,8,8,9,9,10,10,10-Hexadecafluoro-2-decenoic acid
678411	8:2 Perfluoroalkyl phosphate diester	PAP	8	3,3,4,4,5,5,6,6,7,7,8,8,9,9,10,10,10-Heptadecafluoro-1-decanol, 1,1'-(Hydrogen phosphate
73606196	F53B	Novel Polyfluorinated Et	136	2-[(6-Chloro-1,1,2,2,3,3,4,4,5,5,6,6-dodecafluorohexyl)oxy]-1,1,2,2-tetrafluoroethanesulfonic acid postassium salt (1:1
30381987	FC807	FASA	8	N,N'-[Phosphinicobis(oxy-2,1-ethanediyl)]bis[N-ethyl-1,1,2,2,3,3,4,4,5,5,6,6,7,7,8,8,8-heptadecafluoro-1-octanesulfonamide ammonium salt (1:1
375224	PFBA	PFAA PFCA	78	2,2,3,3,4,4,4-Heptafluorobutanoic acid
45048622	PFBA	PFAA PFCA	1	2,2,3,3,4,4,4-Heptafluorobutanoic acid ion (1-)
375735	PFBS	PFAA PFSA	5	1,1,2,2,3,3,4,4,4-Nonafluoro-1-butanesulfonic acid
29420493	PFBS	PFAA PFSA	14	1,1,2,2,3,3,4,4,4-Nonafluoro-1-butanesulfonic acid potassium salt (1:1)
45187153	PFBS	PFAA PFSA	80	1,1,2,2,3,3,4,4,4-Nonafluoro-1-butanesulfonic acid ion(1-)
335762	PFDA	PFAA PFCA	91	2,2,3,3,4,4,5,5,6,6,7,7,8,8,9,9,10,10,10-Nonadecafluorodecanoic acid
307551	PFDoDS	PFAA PFSA	120	2,2,3,3,4,4,5,5,6,6,7,7,8,8,9,9,10,10,11,11,12,12,12-Tricosafluorododecanoic acid
67905195	PFHeDA	PFAA PFCA	3	2,2,3,3,4,4,5,5,6,6,7,7,8,8,9,9,10,10,11,11,12,12,13,13,14,14,15,15,16,16,16-Hentriacontafluorohexadecanoic aci
307244	PFHxA	PFAA PFCA	80	2,2,3,3,4,4,5,5,6,6,6-Undecafluorohexanoic acid
3871996	PFHxS	PFAA PFSA	1	1,1,2,2,3,3,4,4,5,5,6,6,6-Tridecafluoro-1-hexanesulfonic acid potassium salt
108427538	PFHxS	PFAA PFSA	40	1,1,2,2,3,3,4,4,5,5,6,6,6-Tridecafluoro-1-hexanesulfonic acid ion (1-)
375951	PFNA	PFAA PFCA	274	2,2,3,3,4,4,5,5,6,6,7,7,8,8,9,9,9-Heptadecafluorononanoic acid
335671	PFOA	PFAA PFCA	1258	2,2,3,3,4,4,5,5,6,6,7,7,8,8,8-Pentadecafluorooctanoic acid
335955	PFOA	PFAA PFCA	6	2,2,3,3,4,4,5,5,6,6,7,7,8,8,8-pentadecafluorooctanoic acid, Sodium salt (1:1)
3825261	PFOA	PFAA PFCA	55	2,2,3,3,4,4,5,5,6,6,7,7,8,8,8-Pentadecafluorooctanoic acid, Ammonium salt (1:1)
45285516	PFOA	PFAA PFCA	30	2,2,3,3,4,4,5,5,6,6,7,7,8,8,8-Pentadecafluorooctanoic acid ion(1-)
16517116	PFOcDA	PFAA PFCA	3	2,2,3,3,4,4,5,5,6,6,7,7,8,8,9,9,10,10,11,11,12,12,13,13,14,14,15,15,16,16,17,17,18,18,18-Pentatriacontafluorooctadecanoic aci
1763231	PFOS	PFAA PFSA	637	1,1,2,2,3,3,4,4,5,5,6,6,7,7,8,8,8-Heptadecafluoro-1-octanesulfonic acid
2795393	PFOS	PFAA PFSA	1003	1,1,2,2,3,3,4,4,5,5,6,6,7,7,8,8,8-Heptadecafluoro-1-octanesulfonic acid potassium salt
4021470	PFOS	PFAA PFSA	142	1,1,2,2,3,3,4,4,5,5,6,6,7,7,8,8,8-Heptadecafluoro-1-octanesulfonic acid sodium salt (1:1
29457725	PFOS	PFAA PFSA	4	1,1,2,2,3,3,4,4,5,5,6,6,7,7,8,8,8-Heptadecafluoro-1-octanesulfonic acid, Lithium salt
45298906	PFOS	PFAA PFSA	542	1,1,2,2,3,3,4,4,5,5,6,6,7,7,8,8,8-Heptadecafluoro-1-octanesulfonic acid ion(1-
56773423	PFOS	PFAA PFSA	9	N,N,N-triethylethanaminium 1,1,2,2,3,3,4,4,5,5,6,6,7,7,8,8,8-heptadecafluoro-1-octanesulfonate (1:1)
754916	PFOSA	FASA	111	1,1,2,2,3,3,4,4,5,5,6,6,7,7,8,8,8-Heptadecafluoro-1-octanesulfonamide
2706903	PFPeS	PFAA PFSA	3	2,2,3,3,4,4,5,5,5-Nonafluoropentanoic acid
376067	PFTeDA	PFAA PFCA	3	2,2,3,3,4,4,5,5,6,6,7,7,8,8,9,9,10,10,11,11,12,12,13,13,14,14,14-Heptacosafluorotetradecanoic acid
72629948	PFTrDA	PFAA PFCA	932	2,2,3,3,4,4,5,5,6,6,7,7,8,8,9,9,10,10,11,11,12,12,13,13,13-Pentacosafluorotridecanoic acid
2058948	PFUnDA	PFAA PFCA	3	2,2,3,3,4,4,5,5,6,6,7,7,8,8,9,9,10,10,11,11,11-Heneicosafluoroundecanoic acid
4151502	Sulfluramid	PPP	8	N-Ethyl-1,1,2,2,3,3,4,4,5,5,6,6,7,7,8,8,8-heptadecafluoro-1-octanesulfonamide
76051	TFA	PFAA PFCA	6	2,2,2-Trifluoroacetic acid

Table SI-3.10. Summary of PFAS toxicity refferece values (LC50, EC50, LOEC, NOEC) for Cyprinids, by PFAS (and chain length).
Endpoints summarized include mortality, growth, development and reproduction. Data in italics are from more than one study.
Cyprinids include: Chinese rare minnow, common carp, goldfosh, fathead minnow, sheepshead minnow, and zebrafish.

CAS Number	PFAS (Chain Length)	Endpoint Low Range (mg/L)	Endpoint High Range (mg/L)	Endpoint	Organism Lifestage	References
**Perfluoroalkyl acids (PFAAs)**						
	Perfluorobutanoic acid		137.00	NOEC - Growth	Embryo	
**375224**	***PFBA (C4)***	>3000	13795	LC50	Embryo	Hagenaars et al. 2011, Ulhaq et al. 2013, Godfrey et al. 2017a, 2017b, 2019
**375735**	***Perfluorobutane sulfonic acid PFBS (C4)***		1500	LC50	Embryo	Ulhaq et al. 2013
**29420493**	***Potassium perfluorobutane sulfonate PFBS (C4)***		>3000	LC50	Embryo	Hagenaars et al. 2011
**2706903**	***Perfluoropentanoic acid PFPeA (C5)***	0.09	5.00	NOEC - Reproduction	Embryo	
**307244**	***Perfluorohexanoic acid PFHxA (C6)***		91.07	LC50	Embryo	Annunziato et al. 2019
		24.60	759.00	LC50	Embryo	Hagenaars et al. 2011, Zheng et al. 2012, Du et al. 2013, Ulhaq et al. 2013, Yang et al. 2014, Kalasekar et al. 2015, Jantzen et al. 2016, Corrales et al. 2017, Godfrey et al. 2017, Stengel et al. 2017
			0.0034	LOEC - Reproduction	Embryo	
	Perfluorooctanoic acid	0.0034	4.70	LOEC - Growth	Embryo	
**335671**	***PFOA (C8)***	0.08	75.94	NOEC - Growth	Embryo	
**3825261**	***Ammonium perfluorooctanoate PFOA (C8)***	386.26	624.23	LC50	Embryo	Ding et al. 2013
		3.50	81.18	LC50	Embryo	Drottar and Krueger 2000, Ankley et al. 2005, Oakes el al. 2005, Du et al. 2006, 2009, Shi et al. 2009, Sharpe et al. 2010, Hagenaars et al. 2011, 2014, Hoke et al. 2012, Keiter et al. 2012, Ding et al. 2013, Liu rt al. 2013, Yang et al. 2014, Du et al. 2016, 2017, 2018, Qiang et al. 2015, 2016, Wang et al. 2017, Xia and Niu 2017
		0.0007	3.07	LOEC - Growth	Embryo/Larvae	
		0.0007	5.57	NOEC - Growth	Embryo	
		0.33	0.50	NOEC - Development	Embryo	
		0.01	0.25	NOEC - Reproduction	Embryo	
	Perfluorooctane sulfonic	0.20	0.90	LOEC - Reproduction	Adult	
**1763231**	***acid PFOS (C8)***		0.23	EC50 - Reproduction	Adult	
	Ammonium perfluorooctane		0.10	LOEC - Growth	Juvenile	Hagenaars 2008
**56773423**	***sulfonic acid PFOS (C8)***		1.00	NOEC - Growth	Juvenile	
	Perfluorocotane sulfonate		3.00	LOEC - Growth	Embryo	Shi et al. 2004, 2008, Kim et al. 2010, Jantzen et al. 2016
**45298906**	***PFOS (C8)***	0.99	48.24	NOEC -Growth	Embryo	
		84.00	193.00	LC50	Embryo	Zhang et al. 2012, Zheng et al. 2012, Ulhaq et al. 2013, Jantzen et al. 2016
	Perfluorononanoic acid	0.01	1.00	LOEC - Growth	Embryo	
**375951**	***PFNA (C9)***	0.10	0.92	NOEC - Growth	Embryo	
	Perfluorodecanoic acid		8.40	LC50	Embryo	Ulhaq et al. 2013, Jo et al. 2014

Table SI-3.10. Summary of PFAS toxicity refferece values (LC50, EC50, LOEC, NOEC) for Cyprinids, by PFAS (and chain length).
Endpoints summarized include mortality, growth, development and reproduction. Data in italics are from more than one study.
Cyprinids include: Chinese rare minnow, common carp, goldfosh, fathead minnow, sheepshead minnow, and zebrafish.

CAS Number	PFAS (Chain Length)	Endpoint Low Range (mg/L)	Endpoint High Range (mg/L)	Endpoint	Organism Lifestage	References
**335762**	***PFDA (C10)***	0.10	10.00	NOEC - Growth	Embryo	
**72629948**	***Perfluorotridecanoic acid (PFTrDA) (C13)***		0.1	NOEC - Growth	Embryo	Jo et al. 2014
**Fluorotelomere Substances (FTxs)**						
			302.21	*LC50*	*Embryo*	Huang et al. 2010, Wang et al. 2011, Zheng et al. 2012, Ulhaq et al. 2013, Yang et al. 2014, Li et al. 2015, Chen et al. 2016, Cheng et al. 2016, Jantzen et al. 2016, Cui et al. 2017, Stengel et al. 2017, Dang et al. 2018, Annunziato et al. 2019
		34.00	*182.00*	*LC50*	*Embryo*	
			7.28	*NOEC - Growth*	*Embryo*	
		0.01	*1.00*	*NOEC - Growth*	*Embryo*	
		0.05	0.25	LOEC - Growth	Embryo	
		1.12	113.00	EC50 - Development	Embryo	
		0.25	16.00	LOEC - Development	Embryo	
		0.01	0.25	LOEC - Reproduction	Embryo	
**647427**	***Fluorotelomere alcohol 6:2 FTOH (C6)***	0.25	16.00	NOEC - Development	Embryo	
**53826123**	***Tridecafluorooctanoic acid 6:2 FTCA (C6)***	7.33	25.10	LC50	Embryo	Shi et al. 2017
	Fluorotelomere alcohol 8:2	0.03	0.27	LOEC - Reproduction	Adult	Liu et al. 2010
**678397**	***FTOH (C8)***	0.01	0.09	NOEC - Reproduction	Adult	
	Heneicosafluorododecanoic	3.21	7.86	NOEC - Growth	Adult	Mitchell 2010
**53826123**	***acid 10:2 FTCA (C10)***		7.86	NOEC - Development	Embryo	
**53826134**	***Heneicosafluorododecanoic acid 10:2 FTUCA (C10)***		0.32	LOEC - Reproduction	Adult	Mitchell 2010
**Perfluoroalkane sulfonamides (FASAs)**						
**754916**	***Perfluorooctane sulfonamide FOSA (C8)***		0.02	NOEC - Growth	Embryo	Chen et al. 2015
**30381987**	***Ammonium octane sulfonamide FC807 (C8)***		211.00	LC50	Embryo	Zheng et al. 2012
**Polyfluorinated Ether Sulfonates (PFAES)**						
			13.77	LC50	Embryo	Shi et al. 2017, Deng et al. 2018
	Potassium polyfluorinated		0.14	LOEC -Growth	Embryo	
**73606196**	***ether sulfonate F-53B (C8)***	0.01	0.14	NOEC - Growth	Embryo	

Table SI-3.11. Summary of PFAS toxicity refferece values (LC50, EC50, LOEC, NOEC) for other fish Families, by PFAS (and chain length). Endpoints summarized include mortality, growth, development and reproduction.

Fish Family	CAS Number	PFAS (Chain Length)	Endpoint Low Range (mg/L)	Endpoint High Range (mg/L)	Endpoint	Organism Lifestage	References
**Adrianichthyidae (Japanese medaka)**	**Perfluoroalkyl acids (PFAAs)**						
	**375224**	***Perfluorobutanoic acid PFBA (C4)***		137	LOEC - Growth	Embryo	Godfrey 2017
				137	NOEC - Growth	Embryo	
	**335671**	***Perfluorooctanoic acid PFOA (C8)***	0.1	10	LOEC - Mortality	Adult	Ji et al. 2008, Lee et al. 2017, Kang et al. 2019
			1	10	NOEC - Mortality	Adult	
			10	30	NOEC - Growth	Adult	
			10	30	LOEC - Reproduction	Adult	
	**45285516**	***Perfluorooctanoate PFOA (C8)***		1	NOEC - Growth	NR	Oh et al. 2013
				3	NOEC - Reproduction	Adult	
	**1763231**	***Perfluorooctane sulfonic acid PFOS (C8)***	0.01	1	LOEC - Mortality	Adult	Ji et al. 2008
			0.1	1	NOEC - Mortality	Adult	
				0.01	LOEC - Growth	Adult	
				1	NOEC - Growth	Adult	
	**45298906**	***Perfluorocotane sulfonate PFOS (C8)***	1	4	LOEC - Mortality	Embryo	Fang et al. 2012, Wu et al. 2012, Fang et al. 2013, Kang et al. 2019
			1	16	NOEC - Mortality	Embryo	
	**4021470**	***Sodium perfluorooctane sulfonate PFOS (C8)***		1	LOEC - Reproduction	Adult	Qiu et al. 2019
				1	LOEC - Development	Embryo	
				5	LOEC - Mortality	Embryo	
				5	NOEC - Mortality	Embryo	
**Salmonidae (Atlantic salmon, rainbow trout)**	**Perfluoroalkyl acids (PFAAs)**						
	**335671**	***Perfluorooctanoic acid PFOA (C8)***		0.1	NOEC - Growth	Embryo	Spachmo and Arukwe 2012, Arukwe et al. 2013
				0.2	NOEC - Growth	Juvenile	Mortensen et al. 2011
	**3825261**	***Ammonium perfluorooctanoate PFOA (C8)***		40	NOEC - Growth	Embryo	Du Pont 2000, Colombo et al. 2008
			700	4001	LC50	NR	
		Potassium perfluorooctane		3	LOEC - Growth	Adult	Sharpe et al. 2010, Oakes et al. 2005
			1	3	NOEC - Growth	Adult	
			1	3	NOEC - Mortality	Adult	

Table SI-3.11. Summary of PFAS toxicity refferece values (LC50, EC50, LOEC, NOEC) for other fish Families, by PFAS (and chain length). Endpoints summarized include mortality, growth, development and reproduction.

Fish Family	CAS Number	PFAS (Chain Length)	Endpoint Low Range (mg/L)	Endpoint High Range (mg/L)	Endpoint	Organism Lifestage	References
	**2795393**	***sulfonate PFOS (C8)***		2.5	LC50	Parr	
	**4021470**	***Sodium perfluorooctane sulfonate PFOS (C8)***		0.1	NOEC - Growth	Embryo	Mortensen et al. 2011, Arukwe et al. 2013
				0.2	NOEC - Growth	Juvenile	
	**29457725**	***Lithium perfluorooctane sulfonate PFOS (C8)***		4.2	LC50	NR	USEPA 1992
				2	NOEL - Mortality	NR	
	**45298906**	***Perfluorooctane sulfonate PFOS (C8)***		0.1	NOEC - Growth	Embryo	Spachmo and Arukwe 2012
	**335762**	***Perfluorodecanoic acid PFDoDA (C10)***		32	LC50	NR	Hoke et al. 2012
	**59587381**	***Potassium tridecafluorooctane sulfonate PFTrDA (C13)***	4.85	8.7	LOEC -Mortality	Embryo	Hoke et al. 2015
			2.62	4.85	NOEC -Mortality	Embryo	
				8.7	LOEC -Development	Embryo	
				4.85	NOEC - Development	Embryo	
				0.013	NOEC - Growth	NR	
				>107	LC50	NR	
	**Fluorotelomere Substances (FTxs)**						
	**27854315**	***Fluorotelomer saturated carboxylate 8:2 FTCA (C8)***		>100	LC50	NR	Hoke et al. 2012
	**70887842**	***Fluorotelomer unsaturated carboxylate 8:2 FTUCA (C8)***		81	LC50	NR	Hoke et al. 2012
	**27619972**	***Fluorotelomer sulfonic acid 6:2 FTSA (C6)***		>108	LC50	NR	Hoke et al. 2015
	**Plant Protection Products (PPP)**						
	**4151502**	***Sulfluramid Mirex S (C10)***	0.21	0.4	NOEL - Mortality	NR	USEPA 1992
				>0.21	LC50	NR	
**Other Families (catfish, centrarchid, cichlid, cod, eel, flounder, killifish, mullet, mosquitofish, pufferfish, rockfish, sculpin, sea bream,**	**Perfluoroalkyl acids (PFAAs)**						
	**3825261**	***Ammonium perfluorooctanoate PFOA (C8)***		25	NOEC -Growth	NR	Dupont 2000, Li 2011
				634	LC50	NR	
	**29457725**	***Lithium perfluorooctane sulfonate PFOS (C8)***		16	NOEL - Mortality		EPA 1992
				49	LC50		
	**1763231**	***Perfluorooctane sulfonic acid PFOS (C8)***		0.2	NOEC - Growth	Juvenile	Preus-Olsen et al. 2014
				0.2	NOEC - Mortality	Juvenile	

Table SI-3.11. Summary of PFAS toxicity refferece values (LC50, EC50, LOEC, NOEC) for other fish Families, by PFAS (and chain length).
Endpoints summarized include mortality, growth, development and reproduction.

Fish Family	CAS Number	PFAS (Chain Length)	Endpoint Low Range (mg/L)	Endpoint High Range (mg/L)	Endpoint	Organism Lifestage	References
**shiner, sucker)**							Han and Fang 2010, Jeon et al. 2010, Hagenaars et al. 2011, Li 2011, Roland et al. 2014
				0.011	NOEC - Growth	NR	
				2.5	NOEC - Growth	Adult	
				0.5	LOEC - Growth	Juvenile	
				1	NOEC - Growth	NR	
				0.1	LOEC - Growth	Larvae	
				0.1	NOEC - Growth	Larvae	
	**2795393**	***Potassium perfluorooctane sulfonate PFOS (C8)***		2.5	NOEC - Reproduction	Adult	
	**Polyfluoroalkyl phosphates (PAPs)**						
	**678411**	***Perfluoroalkyl phosphate diester***		29	NOEC - Growth	Juvenile	Zabaleta et al. 2017

References for Tables SI-3.8 through 3.11

ECOTOX ID	Authors	Title	Journal, Vol, Pages	Year
344	U.S. Environmental Protection Agency	Pesticide Ecotoxicity Database (Formerly: Environmental Effects Database (EEDB))	Environmental Fate and Effects Division, U.S.EPA, Washington, D.C.:	1992
81515	Ankley,G.T., D.W. Kuehl, M.D. Kahl, K.M. Jensen, A. Linnum, R.L. Leino, and D.A. Villeneuve	Reproductive and Developmental Toxicity and Bioconcentration of Perfluorooctanesulfonate in a Partial Life-Cycle Test with the Fathead Minnow (Pimephales promelas)	Environ. Toxicol. Chem.24(9): 2316-2324	2005
93441	Oakes,K.D., P.K. Sibley, J.W. Martin, D.D. MacLean, K.R. Solomon, S.A. Mabury, and G.J. Van der Kraak	Short-Term Exposures of Fish to Perfluorooctane Sulfonate: Acute Effects on Fatty Acyl-CoA Oxidase Activity, Oxidative Stress, and Circulating Sex Steroids	Environ. Toxicol. Chem.24(5): 1172-1181	2005
114603	Shi,X., Y. Du, P.K.S. Lam, R.S.S. Wu, and B. Zhou	Developmental Toxicity and Alteration of Gene Expression in Zebrafish Embryos Exposed to PFOS	Toxicol. Appl. Pharmacol.230(1): 23-32	2008
114976	Ji,K., Y. Kim, S. Oh, B. Ahn, H. Jo, and K. Choi	Toxicity of Perfluorooctane Sulfonic Acid and Perfluorooctanoic Acid on Freshwater Macroinvertebrates (Daphnia magna and Moina macrocopa) and Fish (Oryzias latipes)	Environ. Toxicol. Chem.27(10): 2159-2168	2008
116895	Du,Y., X. Shi, C. Liu, K. Yu, and B. Zhou	Chronic Effects of Water-Borne PFOS Exposure on Growth, Survival and Hepatotoxicity in Zebrafish: A Partial Life-Cycle Test	Chemosphere74(5): 723-729	2009
118237	Shi,X., L.W.Y. Yeung, P.K.S. Lam, R.S.S. Wu, and B. Zhou	Protein Profiles in Zebrafish (Danio rerio) Embryos Exposed to Perfluorooctane Sulfonate	Toxicol. Sci.110(2): 334-340	2009
119304	Shi,X., C. Liu, G. Wu, and B. Zhou	Waterborne Exposure to PFOS Causes Disruption of the Hypothalamus-Pituitary-Thyroid Axis in Zebrafish Larvae	Chemosphere77(7): 1010-1018	2009
120575	Liu,C., J. Deng, L. Yu, M. Ramesh, and B. Zhou	Endocrine Disruption and Reproductive Impairment in Zebrafish by Exposure to 8:2 Fluorotelomer Alcohol	Aquat. Toxicol.96(1): 70-76	2010
151364	Dupont Haskell Laboratory	Summaries of Studies Conducted at DuPont Haskell Laboratory with Ammonium Perfluorooctanoate and Perfluorononanoate, (with Cover Letter Dated 052500)	EPA/OTS Doc #FYI-OTS-0600-1378:54 p.	2000
151611	Colombo,I., W. De Wolf, R.S. Thompson, D.G. Farrar, R.A. Hoke, and J. L'Haridon	Acute and Chronic Aquatic Toxicity of Ammonium Perfluorooctanoate (APFO) to Freshwater Organisms	Ecotoxicol. Environ. Saf.71(3): 749-756	2008
151613	Han,J., and Z. Fang	Estrogenic Effects, Reproductive Impairment and Developmental Toxicity in Ovoviparous Swordtail Fish (Xiphophorus helleri) Exposed to Perfluorooctane Sulfonate (PFOS)	Aquat. Toxicol.99(2): 281-290	2010
151614	Huang,H., C. Huang, L. Wang, X. Ye, C. Bai, M.T. Simonich, R.L. Tanguay, and Q. Dong	Toxicity, Uptake Kinetics and Behavior Assessment in Zebrafish Embryos Following Exposure to Perfluorooctanesulphonicacid (PFOS)	Aquat. Toxicol.98(2): 139-147	2010
151615	Kim,W.K., S.K. Lee, and J. Jung	Integrated Assessment of Biomarker Responses in Common Carp (Cyprinus carpio) Exposed to Perfluorinated Organic Compounds	J. Hazard. Mater.180(1-3): 395-400	2010
151619	Sharpe,R.L., J.P. Benskin, A.H. Laarman, S.L. MacLeod, J.W. Martin, C.S. Wong, and G.G. Goss	Perfluorooctane Sulfonate Toxicity, Isomer-Specific Accumulation, and Maternal Transfer in Zebrafish (Danio rerio) and Rainbow Trout (Oncorhynchus mykiss)	Environ. Toxicol. Chem.29(9): 1957-1966	2010
152104	Hagenaars,A., L. Vergauwen, W. De Coen, and D. Knapen	Structure-Activity Relationship Assessment of Four Perfluorinated Chemicals Using a Prolonged Zebrafish Early Life Stage Test	Chemosphere82:764-772	2011
159194	Wu,X., Q. Huang, C. Fang, T. Ye, L. Qiu, and S. Dong	PFOs Induced Precocious Hatching of Oryzias melastigma - from Molecular Level to Individual Level	Chemosphere87(7): 703-708	2012
159201	Spachmo,B., and A. Arukwe	Endocrine and Developmental Effects in Atlantic Salmon (Salmo salar) Exposed to Perfluorooctane Sulfonic or Perfluorooctane Carboxylic Acids	Aquat. Toxicol.108:112-124	2012
160092	Keiter,S., L. Baumann, H. Farber, H. Holbech, D. Skutlarek, M. Engwall, and T. Braunbeck	Long-Term Effects of a Binary Mixture of Perfluorooctane Sulfonate (PFOS) and Bisphenol A (BPA) in Zebrafish (Danio rerio)	Aquat. Toxicol.118/119:116-129	2012
160547	Zheng,X.M., H.L. Liu, W. Shi, S. Wei, J.P. Giesy, and H.X. Yu	Effects of Perfluorinated Compounds on Development of Zebrafish Embryos	Environ. Sci. Pollut. Res.19(7): 2498-2505	2012
160550	Fang,C., L. Qiu, X. Wu, Q. Huang, Y. Liao, L. Liu, H. Shen, and S. Dong	PFOS Elicits Transcriptional Responses of the ER, AHR and PPAR Pathways in Oryzias melastigma in a Stage-Specific Manner	Aquat. Toxicol.106/107:9-19	2012
161077	Hoke,R.A., L.D. Bouchelle, B.D. Ferrell, and R.C. Buck	Comparative Acute Freshwater Hazard Assessment and Preliminary PNEC Development for Eight Fluorinated Acids	Chemosphere87(7): 725-733	2012
165818	Ulhaq,M., G. Carlsson, S. Orn, and L. Norrgren	Comparison of Developmental Toxicity of Seven Perfluoroalkyl Acids to Zebrafish Embryos	Environ. Toxicol. Pharmacol.36:423-426	2013

References for Tables SI-3.8 through 3.11

ECOTOX ID	Authors	Title	Journal, Vol, Pages	Year
166317	Arukwe,A., M.V. Cangialosi, R.J. Letcher, E. Rocha, and A.S. Mortensen	Changes in Morphometry and Association Between Whole-Body Fatty Acids and Steroid Hormone Profiles in Relation to Bioaccumulation Patterns in Salmon Larvae Exposed to Perfluorooctane Sulfonic or Perfluorooctane Carboxyl	Aquat. Toxicol.130/131:219-230	2013
168368	Chen,J., R.L. Tanguay, T.L. Tal, Z. Gai, X. Ma, C. Bai, S.C. Tilton, D. Jin, D. Yang, C. Huang, and Q. Dong	Early Life Perfluorooctanesulphonic Acid (PFOS) Exposure Impairs Zebrafish Organogenesis	Aquat. Toxicol.150:124-132	2014
170323	Liu,C., Q. Wang, K. Liang, J. Liu, B. Zhou, X. Zhang, H. Liu, J.P. Giesy, and H. Yu	Effects of Tris(1,3-Dichloro-2-Propyl) Phosphate and Triphenyl Phosphate on Receptor-Associated mRNA Expression in Zebrafish Embryos/Larvae	Aquat. Toxicol.128:147-157	2013
172976	Kalasekar,S.M., E. Zacharia, N. Kessler, N.A. Ducharme, J.A. Gustafsson, I.A. Kakadiaris, and M. Bondesson	Identification of Environmental Chemicals that Induce Yolk Malabsorption in Zebrafish Using Automated Image Segmentation	Reprod. Toxicol.55:20-29	2015
175185	Preus-Olsen,G., M.O. Olufsen, S.A. Pedersen, R.J. Letcher, and A. Arukwe	Effects of Elevated Dissolved Carbon Dioxide and Perfluorooctane Sulfonic Acid, Given Singly and in Combination, on Steroidogenic and Biotransformation Pathways of Atlantic Cod	Aquat. Toxicol.155:222-235	2014
175190	Wang,S., C. Zhuang, J. Du, C. Wu, and H. You	The Presence of MWCNTs Reduces Developmental Toxicity of PFOS in Early Life Stage of Zebrafish	Environ. Pollut.222:201-209	2017
175198	Mortensen,A.S., R.J. Letcher, M.V. Cangialosi, S. Chu, and A. Arukwe	Tissue Bioaccumulation Patterns, Xenobiotic Biotransformation and Steroid Hormone Levels in Atlantic Salmon (Salmo salar) Fed a Diet Containing Perfluorooctane Sulfonic or Perfluorooctane Carboxylic Acids	Chemosphere83(8): 1035-1044	2011
175199	Jeon,J., H.K. Lim, K. Kannan, and S.D. Kim	Effect of Perfluorooctanesulfonate on Osmoregulation in Marine Fish, Sebastes schlegeli, Under Different Salinities	Chemosphere81(2): 228-234	2010
175213	Fang,C., Q. Huang, T. Ye, Y. Chen, L. Liu, M. Kang, Y. Lin, H. Shen, and S. Dong	Embryonic Exposure to PFOS Induces Immunosuppression in the Fish Larvae of Marine Medaka	Ecotoxicol. Environ. Saf.92:104-111	2013
175218	Chen,M., L. Qiang, X. Pan, S. Fang, Y. Han, and L. Zhu	In Vivo and In Vitro Isomer-Specific Biotransformation of Perfluorooctane Sulfonamide in Common Carp (Cyprinus carpio)	Environ. Sci. Technol.49(23): 13817-13824	2015
175221	Ding,G., J. Zhang, Y. Chen, L. Wang, M. Wang, D. Xiong, and Y. Sun	Combined Effects of PFOS and PFOA on Zebrafish (Danio rerio) Embryos	Arch. Environ. Contam. Toxicol.64(4): 668-675	2013
175222	Oh,J.H., H.B. Moon, and E.S. Choe	Alterations in Differentially Expressed Genes After Repeated Exposure to Perfluorooctanoate and Perfluorooctanesulfonate in Liver of Oryzias latipes	Arch. Environ. Contam. Toxicol.64(3): 475-483	2013
175223	Jantzen,C.E., K.A. Annunziato, S.M. Bugel, and K.R. Cooper	PFOS, PFNA, and PFOA Sub-Lethal Exposure to Embryonic Zebrafish have Different Toxicity Profiles in Terms of Morphometrics, Behavior and Gene Expression	Aquat. Toxicol.175:160-170	2016
175260	Yang,S., F. Xu, F. Wu, S. Wang, and B. Zheng	Development of PFOS and PFOA Criteria for the Protection of Freshwater Aquatic Life in China	Sci. Total Environ.470/471:677-683	2014
175366	Drottar,K.R., and H.O. Krueger	PFOS: An Early Life-Stage Toxicity Test with the Fathead Minnow (Pimephales promelas)	Project 454-108, Wildlife International Ltd., Easton, MD:88 p.	2000
175499	Stengel,D., F. Zindler, and T. Braunbeck	An Optimized Method to Assess Ototoxic Effects in the Lateral Line of Zebrafish (Danio rerio) Embryos	Comp. Biochem. Physiol. C Toxicol. Pharmacol.193:18-29	2017
175649	Cheng,J., S. Lv, S. Nie, J. Liu, S. Tong, N. Kang, Y. Xiao, Q. Dong, C. Huang, and D. Yang	Chronic Perfluorooctane Sulfonate (PFOS) Exposure Induces Hepatic Steatosis in Zebrafish	Aquat. Toxicol.176:45-52	2016
175658	Hagenaars,A., E. Stinckens, L. Vergauwen, L. Bervoets, and D. Knapen	PFOS Affects Posterior Swim Bladder Chamber Inflation and Swimming Performance of Zebrafish Larvae	Aquat. Toxicol.157:225-235	2014
175667	Qiang,L., X. Shi, X. Pan, L. Zhu, M. Chen, and Y. Han	Facilitated Bioaccumulation of Perfluorooctanesulfonate in Zebrafish by Nano-TiO2 in Two Crystalline Phases	Environ. Pollut.206:644-651	2015
175685	Chen,J., X. Wang, X. Ge, D. Wang, T. Wang, L. Zhang, R.L. Tanguay, M. Simonich, C. Huang, and Q. Dong	Chronic Perfluorooctanesulphonic Acid (PFOS) Exposure Produces Estrogenic Effects in Zebrafish	Environ. Pollut.218:702-708	2016
175706	Jo,A., K. Ji, and K. Choi	Endocrine Disruption Effects of Long-Term Exposure to Perfluorodecanoic Acid (PFDA) and Perfluorotridecanoic Acid (PFTrDA) in Zebrafish (Danio rerio) and Related Mechanisms	Chemosphere108:360-366	2014

References for Tables SI-3.8 through 3.11

**ECOTOX ID**	**Authors**	**Title**	**Journal, Vol, Pages**	**Year**
175708	Du,G., H. Huang, J. Hu, Y. Qin, D. Wu, L. Song, Y. Xia, and X. Wang	Endocrine-Related Effects of Perfluorooctanoic Acid (PFOA) in Zebrafish, H295R Steroidogenesis and Receptor Reporter Gene Assays	Chemosphere91(8): 1099-1106	2013
175716	Roland,K., P. Kestemont, R. Loos, S. Tavazzi, B. Paracchini, C. Belpaire, M. Dieu, M. Raes, and F. Silvestre	Looking for Protein Expression Signatures in European Eel Peripheral Blood Mononuclear Cells After In Vivo Exposure to Perfluorooctane Sulfonate and a Real World Field Study	Sci. Total Environ.468/469:958-967	2014
175760	Jantzen,C.E., K.M. Annunziato, and K.R. Cooper	Behavioral, Morphometric, and Gene Expression Effects in Adult Zebrafish (Danio rerio) Embryonically Exposed to PFOA, PFOS, and PFNA	Aquat. Toxicol.180:123-130	2016
176328	Stengel,D., S. Wahby, and T. Braunbeck	In Search of a Comprehensible Set of Endpoints for the Routine Monitoring of Neurotoxicity in Vertebrates: Sensory Perception and Nerve Transmission in Zebrafish (Danio rerio) Embryos	Environ. Sci. Pollut. Res. Int.12:19 p.	2017
176905	Cui,Y., S. Lv, J. Liu, S. Nie, J. Chen, Q. Dong, C. Huang, and D. Yang	Chronic Perfluorooctanesulfonic Acid Exposure Disrupts Lipid Metabolism in Zebrafish	Hum. Exp. Toxicol.36(3): 207-217	2017
177079	Lee,J.W., J.W. Lee, K. Kim, Y.J. Shin, J. Kim, S. Kim, H. Kim, P. Kim, and K. Park	PFOA-Induced Metabolism Disturbance and Multi-Generational Reproductive Toxicity in Oryzias latipes	J. Hazard. Mater.340:231-240	2017
177084	Li,Y., Z. Han, X. Zheng, Z. Ma, H. Liu, J.P. Giesy, Y. Xie, and H. Yu	Comparison of Waterborne and In Ovo Nanoinjection Exposures to Assess Effects of PFOS on Zebrafish Embryos	Environ. Sci. Pollut. Res. Int.22(3): 2303-2310	2015
177092	Du,J., S. Wang, H. You, and Z. Liu	Effects of ZnO Nanoparticles on Perfluorooctane Sulfonate Induced Thyroid-Disrupting on Zebrafish Larvae	J. Environ. Sci.47:153-164	2016
177094	Qiang,L., X. Pan, L. Zhu, S. Fang, and S. Tian	Effects of Nano-TiO2 on Perfluorooctanesulfonate Bioaccumulation in Fishes Living in Different Water Layers: Implications for Enhanced Risk of Perfluorooctanesulfonate	Nanotoxicology10(4): 471-479	2016
177119	Li,M.H.	Changes of Cholinesterase and Carboxylesterase Activities in Male Guppies, Poecilia reticulate, After Exposure to Ammonium Perfluorooctanoate, but not to Perfluorooctane Sulfonate	Fresenius Environ. Bull.20(8a): 2065-2070	2011
177124	Du,J., S. Wang, H. You, R. Jiang, C. Zhuang, and X. Zhang	Developmental Toxicity and DNA Damage to Zebrafish Induced by Perfluorooctane Sulfonate in the Presence of ZnO Nanoparticles	Environ. Toxicol.31(3): 360-371	2016
177131	Godfrey,A., B. Hooser, A. Abdelmoneim, K.A. Horzmann, J.L. Freemanc, and M.S. Sepulveda	Thyroid Disrupting Effects of Halogenated and Next Generation Chemicals on the Swim Bladder Development of Zebrafish	Aquat. Toxicol.193:228-235	2017
177136	Corrales,J., L.A. Kristofco, W.B. Steele, G.N. Saari, J. Kostal, E.S. Williams, M. Mills, E.P. Gallagher, T.J. Kavanagh	Toward the Design of Less Hazardous Chemicals: Exploring Comparative Oxidative Stress in Two Common Animal Models	Chem. Res. Toxicol.30(4): 893-904	2017
177139	Godfrey,A., A. Abdel-Moneim, and M.S. Sepulveda	Acute Mixture Toxicity of Halogenated Chemicals and Their Next Generation Counterparts on Zebrafish Embryos	Chemosphere181:710-712	2017
177144	Xia,J., and C. Niu	Acute Toxicity Effects of Perfluorooctane Sulfonate on Sperm Vitality, Kinematics and Fertilization Success in Zebrafish	Chin. J. Oceanol. Limnol.35(4): 723-728	2017
177170	Zabaleta,I., E. Bizkarguenaga, U. Izagirre, N. Negreira, A. Covaci, J.P. Benskin, A. Prieto, and O. Zuloaga	Biotransformation of 8:2 Polyfluoroalkyl Phosphate Diester in Gilthead Bream (Sparus aurata)	Sci. Total Environ.609:1085-1092	2017
177205	Godfrey,A.E.	Endocrine Disrupting Effects of Halogenated Chemicals on Fish	Ph.D.Thesis, Purdue University, West Lafayette, IN:125 p.	2017
177252	Shi,G., Q. Cui, Y. Pan, N. Sheng, Y. Guo, and J. Dai	6:2 Fluorotelomer Carboxylic Acid (6:2 FTCA) Exposure Induces Developmental Toxicity and Inhibits the Formation of Erythrocytes During Zebrafish Embryogenesis	Aquat. Toxicol.190:53-61	2017
177254	Hoke,R.A., B.D. Ferrell, T. Ryan, T.L. Sloman, J.W. Green, D.L. Nabb, R. Mingoia, R.C. Buck, and S.H. Korzeniowski	Aquatic Hazard, Bioaccumulation and Screening Risk Assessment for 6:2 Fluorotelomer Sulfonate	Chemosphere128:258-265	2015
178026	Dang,Y., F. Wang, and C. Liu	Real-Time PCR Array to Study the Effects of Chemicals on the Growth Hormone/Insulin-Like Growth Factors (GH/IGFs) Axis of Zebrafish Embryos/Larvae	Chemosphere207:365-376	2018
178030	Shi,G., Q. Cui, Y. Pan, N. Sheng, S. Sun, Y. Guo, and J. Dai	6:2 Chlorinated Polyfluorinated Ether Sulfonate, a PFOS Alternative, Induces Embryotoxicity and Disrupts Cardiac Development in Zebrafish Embryos	Aquat. Toxicol.185:67-75	2017
178388	Mitchell,R.J.	The Toxicity of Fluorotelomer Acids to Freshwater Organisms and a Preliminary Evaluation of Mechanism of Action	M.S. Thesis, University of Guelph, Ontario, Canada:118 p.	2010

References for Tables SI-3.8 through 3.11

ECOTOX ID	Authors	Title	Journal, Vol, Pages	Year
178562	Annunziato,K.M., C.E. Jantzen, M.C. Gronske, and K.R. Cooper	Subtle Morphometric, Behavioral and Gene Expression Effects in Larval Zebrafish Exposed to PFHxA, PFHxS and 6:2 FTOH	Aquat. Toxicol.208:126-137	2019
178563	Qiu,X., N. Iwasaki, K. Chen, Y. Shimasaki, and Y. Oshima	Tributyltin and Perfluorooctane Sulfonate Play a Synergistic Role in Promoting Excess Fat Accumulation in Japanese Medaka (Oryzias latipes) via In Ovo Exposure	Chemosphere220:687-695	2019
179525	Du,J., J. Cai, S. Wang, and H. You	Oxidative Stress and Apotosis to Zebrafish (Danio rerio) Embryos Exposed to Perfluorooctane Sulfonate (PFOS) and ZnO Nanoparticles	Int. J. Occup. Med. Environ. Health30(2): 213-229	2017
179529	Du,J., J. Tang, S. Xu, J. Ge, Y. Dong, H. Li, and M. Jin	Parental Transfer of Perfluorooctane Sulfonate and ZnO Nanoparticles Chronic Co-Exposure and Inhibition of Growth in F1 Offspring	Regul. Toxicol. Pharmacol.98:41-49	2018
179846	Kang,J.S., T.G. Ahn, and J.W. Park	Perfluorooctanoic Acid (PFOA) and Perfluooctane Sulfonate (PFOS) Induce Different Modes of Action in Reproduction to Japanese Medaka (Oryzias latipes)	J. Hazard. Mater.368:97-103	2019
179858	Deng,M., Y. Wu, C. Xu, Y. Jin, X. He, J. Wan, X. Yu, H. Rao, and W. Tu	Multiple Approaches to Assess the Effects of F-53B, a Chinese PFOS Alternative, on Thyroid Endocrine Disruption at Environmentally Relevant Concentrations	Sci. Total Environ.624:215-224	2018
180256	E.I. Du Pont De Nemours	Support: Summaries of Environmental & Health Effects Studies with Ammonium Perfluorooctanoate & Perfluorononanoate, with Cover Letter Dated 052500	EPA/OTS Doc #FYI-OTS-0600-1378:54 p.	2000
180423	Drottar,K.R., and H.O. Krueger	PFOS: A 96-Hour Static Acute Toxicity Test with the Fathead Minnow (Pimephales promelas)	Project 454A-102, Wildlife International Ltd., Easton, MD:58 p.	2000

Table SI-3.13. Summary of PFAS toxicity reference values (50% lethal concentration [LC50], 10% effect concentration [EC10], loewest observed effect concentration [LOEC], no observed effect concentration [NOEC]) for amphibians, by perfluoroalkyl and polyfluoroalkyl substances (PFAS) and chain length.

CAS Number	PFAS (chain length)	Endpoint Low Range (mg/L)	Endpoint High Range (mg/L)	Endpoint	Organism Lifestage	Exposure (days)	References
**Perfluoroalkyl acids (PFAAs)**							
	**Perfluorohexane**		0.01	LOEC - Stage	Tadpole GS26	40	Hoover et al. 2017
108427538	**sulfonic acid PFHxS**		1.00	NOEC - Length/Stage	Tadpole GS26	40	
							Kim et al. 2013, Yang et al.
		114.74	377.47	LC50	Tadpole	4	2014, Hoover et al. 2017
			5.89	EC10 - Lifespan	Tadpole	30	
			207.00	LOEC - Length	Embryo	4	
	Perfluorooctanoic acid	1.00	104.00	NOEC - Length/Weight/Stage	Tadpole	30	
335671	***PFOA (C8)***		1	NOEC - Length/Stage	Tadpole GS26	40	
							Palmer and Krueger 2001, Ankley et al. 2004, Cheng et al. 2011, Yang et al. 2014, San-Segundo et al. 2016, Hoover et al. 2017
**1763231**	***Perfluorooctanesulfonic acid PFOS (C8)***		0.10	NOEC - Mortality	Tadpole	67	
	Perfluorooctane		0.10	LOEC - Stage	Tadpole GS26	40	
45298906	***sulfonate PFOS (C8)***	0.01	1.00	NOEC - Length/Stage	Tadpole GS26	40	
		6.21	48.21	LC50	Tadpole	35	
			2.00	EC10 - Lifespan	Tadpole	30	
	Potassium	3.55	48.00	LOEC - Length/Metamorphosis	Embryo	8	
	perfluorooctane						
2795393	***sulfonate PFOS (C8)***	0.97	96.00	NOEC - Length/Weight/Metamorphosis	Tadpole	30	
			335.81	LC50	Embryo	4	Kim et al. 2013
	Perfluorononanoic		232.04	LOEC - Length	Embryo	4	Mitchell 2010
375951	***acid PFNA (C9)***	3.50	116.02	NOEC - Length/Weight	Embryo	4	
			64.26	LOEC - Length	Embryo	4	Kim et al. 2013, Mitchell 2010
		3.50	51.41	NOEC - Length/Weight	Embryo	4	
	Perfluorodecanoic acid						
	PFDA (C10)		76.50	LC50	Embryo	4	
			68.82	LC50	Embryo	4	Kim et al. 2013, Mitchell 2010
	Perfluoroundecanoic		45.13	LOEC - Length	Embryo	4	
2058948	***acid PFUnA (C11)***	3.50	33.85	NOEC - Length/Weight	Embryo	4	
**Fluorotelomere Substances (FTxs)**							
**425670753**	***Fluorotelomere sulfonate 6:2 FTS (C6)***		1.00	NOEC - Length/Stage	Tadpole GS26	30	Hoover et al. 2017
**53826134**	***Heneicosafluorododecanoic acid 10:2 FTCA (C10)***		15.00	NOEC - Length/Weight	Embryo	4	Mitchell 2010
**70887944**	***Fluorotelomer unsaturated carboxylate 10:2 FTUCA (C10)***		7.50	NOEC - Length/Weight	Embryo	4	Mitchell 2010

References for Table Table SI-3.13

ECOTOX ID	Authors	Title	Journal, Vol, Pages	Year
77666	Ankley,G.T., D.W. Kuehl, M.D. Kahl, K.M. Jensen, B.C. Butterworth, and J.W. Nichols	Partial Life-Cycle Toxicity and Bioconcentration Modeling of Perfluorooctanesulfonate in the Northern Leopard Frog (Rana pipiens)	Environ. Toxicol. Chem.23(11): 2745-2755	2004
157821	Cheng,Y., Y. Cui, H.M. Chen, and W.P. Xie	Thyroid Disruption Effects of Environmental Level Perfluorooctane Sulfonates (PFOS) in Xenopus laevis	Ecotoxicology20(8): 2069-2078	2011
170608	Kim,M., J. Son, M.S. Park, Y. Ji, S. Chae, C. Jun, J.S. Bae, T.K. Kwon, Y.S. Choo, H. Yoon, D. Yoon, J. Ryoo, S.H. Kim,	In Vivo Evaluation and Comparison of Developmental Toxicity and Teratogenicity of Perfluoroalkyl Compounds Using Xenopus Embryos	Chemosphere93(6): 1153-1160	2013
175260	Yang,S., F. Xu, F. Wu, S. Wang, and B. Zheng	Development of PFOS and PFOA Criteria for the Protection of Freshwater Aquatic Life in China	Sci. Total Environ.470/471:677-683	2014
175357	Palmer,S.J., and H.O. Krueger	PFOS: A Frog Embryo Teratogenesis Assay - Xenopus (FETAX)	Project 454A-116, Wildlife International Ltd., Easton, MD:181 p.	2001
175663	San-Segundo,L., L. Guimaraes, C.F. Torija, E.M. Beltran, L. Guilhermino, and M.V. Pablos	Alterations in Gene Expression Levels Provide Early Indicators of Chemical Stress During Xenopus laevis Embryo Development: a Case Study with Perfluorooctane Sulfonate (PFOS)	Ecotoxicol. Environ. Saf.127:51-60	2016
176982	Hoover,G.M., M.F. Chislock, B.J. Tornabene, S.C. Guffey, Y.J. Choi, C. De Perre, J.T. Hoverman, L.S. Lee, and M.S. Sepu	Uptake and Depuration of Four Per/Polyfluoroalkyl Substances (PFASs) in Northern Leopard Frog Rana pipiens Tadpoles	Environ. Sci. Technol. Lett.4(10): 399-403	2017
178388	Mitchell,R.J.	The Toxicity of Fluorotelomer Acids to Freshwater Organisms and a Preliminary Evaluation of Mechanism of Action	M.S. Thesis, University of Guelph, Ontario, Canada:118 p.	2010

Table SI-3.14 Avian ecotoxicological studies conducted with PFAS-Laboratory studies

		Egg Injection Studies[1]				Acute/Chronic Studies[1]					
						Mallard		Bobwhite Quail		Japanese Quail	
**PFSA**		Chicken	Cormorant	Herring Gull	Yellow-legged Gull	Acute	Chronic	Acute	Chronic	Acute	Chronic
PFBS	4										
PFHxS	6	X				X		X	X		
PFOS	8	X	X	X	X	X	X	X	X	X	X
F-35B	8	X									
3M-AFFF[2]	8									X	X
**PFCA**											
PFHXA	5	X									
PFOA	7	X	X	X						X	
**Fluorotelome**r											
Ansul[3]	5									X	

[1] Table does not include mechanistic studies, only those that included apical endpoints
[2] 3M-AFFF contains over 90% PFOS
[3] Ansul is a fluorotelomer, 6:2 FtTAoS

Table SI-3.15 Dietary acute toxicity of PFAS to avian species

PFAS	Species	NOEC (mg/kg)	LC50 (mg/kg)	LD50 (mg/kg bw/d)	Reference
PFBS	Bobwhite Quail	>10,000	>10,000	>2,304	Newsted JL et al. 2008. Arch. Environ. Contam. Toxicol. 54:535-545
	Mallard	>10,000	>10,000	>3,974	Newsted JL et al. 2008. Arch. Environ. Contam. Toxicol. 54:535-545
PFOS	Bobwhite Quail	70.3	212(158-278)	61 (48-77)	Newsted JL et al. 2006. Arch. Environ. Contam. Toxicol. 50:411-420
	Mallard	141	603 (431-938)	150 (117-201)	Newsted JL et al. 2006. Arch. Environ. Contam. Toxicol. 50:411-420
	Japanese Quail	66	245 (190-315)	24 (21-28)	Bursian SB et al. 2018. 39th Annual Meeting of SETAC, Sacramento CA, USA
3M -AFFF[1]	Japanese Quail	73	631 (486-821)	184 (131-257)	Bursian SB et al. 2018. 39th Annual Meeting of SETAC, Sacramento CA, USA
PFOA	Japanese Quail	162	550 (473-639)	69 (64-76)	Bursian SB et al. 2018. 39th Annual Meeting of SETAC, Sacramento CA, USA
Ansul[2]	Japanese Quail	1,1180	>1,118	>257	Bursian SB et al. 2018. 39th Annual Meeting of SETAC, Sacramento CA, USA

[1] 3M AFFF concentrations are based on PFOS measured in the formulation. PFOS contributes > 90% of the fluorinated content
[2] Ansul is a 6:2 fluorotelomer (6:2 FtTAoS)

Table SI-3.16 Chronic laboratory studied with avian species exposed to PFAS in the diet (feed)

PFAS	Species	Endpoints	NOAEL (mg/kg bw/d)	LOAEL (mg/kg bw/d)	Reference
PFOS	Mallard	Mortality<br>Body Weight<br>Reproduction	1.48<br>1.48<br>1.48	6.36	Newsted JL et al. 2007. Environ. Toxicol. Pharmacol. 23: 1-9
	Bobwhite Quail	Mortality<br>Body Weight<br>Chick growth	0.77<br>0.77	2.64<br><br>0.77	Newsted JL et al. 2007. Environ. Toxicol. Pharmacol. 23: 1-9
PFOS	Japanese Quail	Body Weight (female)<br>Egg production<br>Hatchability<br>Chick survival	 <br>0.55<br>0.55	0.28<br>0.28<br>1.1<br>1.1	Bursian SB et al. 2019. 40th Annual Meeting of SETAC, Toronto Canada
3M AFFF[1]	Japanese Quail	Body Weight<br>Egg production<br>Hatchability<br>Chick Survival	2.5<br>1.4<br>1.4<br>0.66	3.4<br>2.5<br>2.5<br>1.4	Bursian SB et al. 2019. 40th Annual Meeting of SETAC, Toronto Canada
PFBS	Bobwhite Quail	Body weight<br>Egg production<br>Hatchability	87.7<br>87.7<br>87.7		Newsted JL et al. 2008. Arch. Environ. Contam. Toxicol. 54: 535-545

[1] 3M AFFF concentrations are based on PFOS measured in the formulation. PFOS contributes > 90% of the fluorinated content

Table SI-3.17 Egg injection studies in avian species: Effects of PFAS on environmentally relevant endpoints

PFAS	Species	Endpoints	NOAEL (ug/g egg)	LOAEL (ug/g egg)	Reference
PFOS	Chicken	Hatching Success	0.1		Molina ED. et al. 2006. Environ. Toxicol. Chem. 25: 227-232
PFOS	Chicken	Pipping Success	5.0	100	O'Brien JM. et al. 2009. Comp. Biochem. Physiol. Part C 149:524-530
PFOS	Chicken	Embryo Survival	3.0	10	Norden M. et al. 2016. Environ. Sci. Poll. Res. 23: 10855-10862
PFOS	Chicken	Hatching Success<br>Immune Function	5.0	 1.0	Peden-Adams J. et al. 2009. Reprod. Toxicol. 27: 307-318
PFOS	Chicken	Embryo Survival<br>Hatching Success		5.0<br>5.0	Pinkas A. et al. 2010. Neurotoxicology and Teratology 32: 182-186
PFOS	Chicken	Embryo Survival		>100	Stromquist M et al. 2012. Comp. Biochem. Physiol. Part C 156: 29-36
PFOS	Cormorant	Embryo Survival			Norden M. et al. 2016. Environ. Sci. Poll. Res. 23: 10855-10862
PFOS	Herring Gull	Embryo Survival			Norden M. et al. 2016. Environ. Sci. Poll. Res. 23: 10855-10862
PFOS	Yellow-legged Gull	Embryo Survival			Parolini M. et al. 2016. Environ. Sci. Poll. Res. 23: 426-437
PFOS	Chicken	Embryo Survival	0.5	1.6	Norden M. et al. 2016. Environ. Sci. Poll. Res. 23: 10855-10862
PFOA	Cormorant	Embryo Survival		>10	Norden M. et al. 2016. Environ. Sci. Poll. Res. 23: 10855-10862
PFOA	Herring Gull	Embryo Survival		10	Norden M. et al. 2016. Environ. Sci. Poll. Res. 23: 10855-10862
PFOA	Chicken	Hatching Success<br>Heart Morphology	1.0	2.0<br>0.5	Norden M. et al. 2016. Environ. Sci. Poll. Res. 23: 10855-10862
PFOA	Chicken	Hatching Success		5.0	Norden M. et al. 2016. Environ. Sci. Poll. Res. 23: 10855-10862
PFHxS	Chicken	Pipping Success<br>Brain II 5'-deiodinase (D2)	9.3<br>0.89	38<br>9.3	Cassone CG. et al. 2012. Toxicol. Sci. 127:216-224
PFHxA	Chicken	Pipping Success<br>Brain II 5'-deiodinase (D2)		>9.7<br>>9.7	Cassone CG. et al. 2012. Toxicol. Sci. 127:216-224
F-53B	Chicken	Pipping Success<br>Hatching Success<br>Heart Rate		>1.5<br>>1.5<br>0.15	Briels N. et al. 2018 Environ. Sci. Technol. 52: 12859-12867

Table SI-3.18. Laboratory studies with non-wildlife mammals with endpoints that are relevant for ecological risk assessments

Chemical	Species	Endpoint	NOAEL (mg/kg bw d)	LOAEL (mg/kg bw/d)	Reference
**PFCA**					
PFBA	Rat	Delayed eye opening<br>Delayed puberty	35<br><35	175	Das KP et al. (2008). Toxicol. Sci. 105(1): 173-181.
PFHxA	Rat	Survival	30	200	Klaunig J et al. 2015. Toxicol Pathol 43:209-220
PFOA	Rat	F1-pup mortality	1.3	14	Butenhoff JL et al. 2004. Toxicol. 196:95-116
PFNA	Mouse	F1-pup mortality	0.83	1.1	Wolf CJ et al. 2010. PPAR Res. 282896:1-11
PFDA	Mouse	Growth	0.3	1.0	Harris and Birnbaum. 1989. Fund. Applied Toxicol. 12: 442-448
PFUnDA	Rat	Pup growth	0.3	1.0	Takahashi M et al. 2014. J. Toxicol. Sci. 39(1): 97-108
PFDoA	Rat	Body weight gain	0.5	2.5	Kato H et al. 2015. Environ. Toxicol. 30:1244-1263
PFTeDA	Rat	Growth	3	10	Hirata-Koizumi M et al. 2015. Toxicol. Sci. 2(4): 177-190
**PFSA**					
PFBS	Rat	F1 Boddy weight<br>Reproduction	300<br>1000	1000	Lieder P. 2009. Toxicol 259:33-45
PFHxS	Mouse	Decreased litter size	0.3	1.0	Chang SC et al. 2018. Reprod Toxicol 78:150-168
PFOS	Rat	Pup growth/development	0.4	1.6	Luebker DJet al. 2005. Toxicol 215:126-148
Et-FOSE	Rat	Fetal weight	5	10	Case MT et al. 2001. Inter J Toxicol 20:101-109
**Fluorotelomer**					
6:2 FTOH	Rat	Reproduction	25	125	O'Connor JC et al. 2014Toxicol 317:6-16
8:2 FTOH	Rat	Reproduction	50	200	Mylchreest E et al. 2005. Drug Chem Toxicol 28:315-328

**Table SI-3.19**. Examination species sensitivity of laboratory mammals to PFOS exposure: Effects on growth				
Species	Endpoint	NOAEL (mg/kd bw/d)	LOAEL (mg/kg bw/d)	Reference
Rat	Body weight ↓	1.0	2.0	Thibodeaux JR et al. 2003
Mouse	Body weight ↓	10	15	
Rat	Body weight ↓	0.4	1.6	Luebker et al. 2005
Rat (Male)	Body Weight ↓	0.42	0.98	Butenhoff et al. 2012
Rat (Female)	Body Weight ↓	0.299	1.25	
Cynomolgus monkey	Body Weight ↓	0.15	0.75	Seacat et al. 2002
Mink	Body Weight ↓	0.34	1.33	Newsted et al. 2010
Rabbit	Body Weight ↓	0.1	1.0	Case et al. 2001

Table SI-3.20 Laboratory studies with non-wildlife mammals: Non-apical endpoints that may be relevant for ecological risk assessments

Chemical	Species	Endpoint	NOAEL (mg/kg/d)	LOAEL (mg/kg/d)	Reference
**Immunological**					
PFOS	Mouse	Spleenocyte, IL-4↓	0.017	0.083	Dong GH. 2011. Arch Toxicol 85:1235-1244.
	Mouse	Sheep RBC plaque forming cell ↓	0.0017	0.017	Peden-Adams MM. 2008 Toxicol Sci 104:144-154
PFOA	Mouse	TDAR IgM AB↓	7.5	30	Dewitt JC et al. 2016. J. Immunotox. 13: 38-45
PFNA	Mouse	CD49 and IL-4↓		1.0	Fang X et al. 2008. Toxicol. Sci. 105(2): 312-321
GenX	Mouse	TDAR IgM AB↓	10	100	Rushing BR et al. 2017. Toxicol. Sci. 156(1): 179-189
**Neurological/Behavioral**					
PFHxS	Rat	Locomotor activity	3.0		Butenhoff JL et al. 2009. Reprod Toxicol 27:331–341
PFOS	Mouse	Spatial Memory	0.43	2.15	Long M et al. 2013. Plos One 8(1):e54176.
PFOS	Rat	Hippocampus HVA↓	0.1		Zeng H-C. 2011. Neurotox 32:130-139
PFBA	Rat	Locomotory activity	30		Butenhoff JL et al. 2012. Reprod Toxicol 33:513–530
PFOA	Mouse	Locomotor activity		0.3	Onishchenko N et al. 2011. Neurotox Res. 19:452-461
