# Supplementary material for: Assessing the Ecological Risks of Per‐ and Polyfluoroalkyl Substances: Current State‐of‐the Science and a Proposed Path Forward
Source: Environ Toxicol Chem. 2020 Nov 6;40(3):564–605. doi: 10.1002/etc.4869 (PMC7984443; doi:10.1002/etc.4869)
Supplement: Supplementary file 5 — Supporting information. [file ETC-40-564-s001.pdf]

Supplemental Information 4
Ankley et al. 2020
Assessing the Ecological Risks of Per- and Polyfluoroalkyl Substances: Current State-of-the Science and a Proposed Path Forward

Table SI-4.1. Attendees at the 2019 Society for Environmental Chemistry and Toxicology (SETAC) Perfluoroalkyl and Polyfluoroalkyl substances (PFAS) Focus Topic Meeting who participated in the

Name	Organization	City/Town	State/Prov	Country	Sector
Damien Moodie	RMIT Uni.,Melbourne	Melbourne	Victoria	Australia	Academia
Tanya Paige	RMIT University	Melbourne	Victoria	Australia	Academia
Drew Szabo	RMIT University	Melbourne	Victoria	Australia	Academia
Kirsten Broadgate	Golder Associates Pty, Ltd.	Richmond, Victoria	Victoria	Australia	Business
Sara Broomhall	Australian Government Department of the Environment and Energy	Parkes	Australian Capital Territory	Australia	Government
Anu Kumar	CSIRO	Glen Osmond	South Australia	Australia	Government
Filipe Gimenes	GEOKLOCK	São Paulo	SP	Brazil	Business
Philippa Cureton	Environment and Climate Change Canada	Gatineau	Quebec	Canada	Government
Magali Houde	Environment and Climate Change Canada	Montreal	Quebec	Canada	Government
Lisa Sealock	Environment and Climate Change Canada	Toronto	Ontario	Canada	Government
Jessy Kurias	Environment and Climate Change Canada	Gatineau	Quebec	Canada	Government
Sara Valsecchi	Water Research Institute - Italian National Research Council IRSA-CNR	Brugherio	MB	Italy	Academia
Dick Roelofs	Vrije Universiteit	Amsterdam	VU campus	Netherlands	Academia
Louis Tremblay	Cawthron Institute	Nelson	South Island	New Zealand	Academia
Andrew Rumsby	Pattle Delamore Partners	Auckland	nz	New Zealand	Business
Karina Petersen	NIVA - Norwegian Institute for Water Research	Oslo	Oslo	Norway	Other
Scott Belcher	North Carolina State University	Raleigh	North Carolina	United States	Academia
Marianna Brown-Augustine	University of California, Berkeley	Berkeley	California	United States	Academia
G. Allen Burton	University of Michigan	Ann Arbor	Michigan	United States	Academia
W. Gregory Cope	North Carolina State University	Raleigh	North Carolina	United States	Academia
Daniel Elias	North Carolina Wesleyan College	Rocky Mount	North Carolina	United States	Academia
Theresa Guillette	North Carolina State University	Raleigh	North Carolina	United States	Academia
Matt Hamilton	Purdue University	West Lafayette	Indiana	United States	Academia
Tyler Hoskins	Purdue University	West Lafayette	Indiana	United States	Academia
Tabitha King	George Mason University	Warrenton	Virginia	United States	Academia
Roman Lanno	Ohio State University	Columbus	Ohio	United States	Academia
Melissa Lech	Purdue University	West Lafayette	Indiana	United States	Academia
Yuanbo Li	North Carolina State University	Raleigh	North Carolina	United States	Academia
Cheryl Murphy	Michigan State University	East Lansing	Michigan	United States	Academia
Edgar Perez	Purdue University	West Lafayette	Indiana	United States	Academia
Anna Robuck	University of Rhode Island	Narragansett	Rhode Island	United States	Academia
Christopher Salice	Towson University	Towson	Maryland	United States	Academia
Bonner Anthony	Arcadis	Raleigh	North Carolina	United States	Business
Aaron Bernhardt	Tetra Tech, Inc.	Pittsburgh	Pennsylvania	United States	Business
Amanda Bess	Chevron Energy Technology Company	Houston	Texas	United States	Business
Karen Christensen	ExxonMobil Biomedical Sciences Inc.	Annandale	New Jersey	United States	Business
William Goodfellow	Exponent	York	Pennsylvania	United States	Business
Heather Govenor	EnSafe Inc.	Radford	Virginia	United States	Business
Robert Hoke	E. I. du Pont de Nemours and Company	Wilmington	Delaware	United States	Business
Steven Jones	GHD	Plainville	Connecticut	United States	Business
Katrina Leigh	Ramboll	Beachwood	Ohio	United States	Business
Evelyn Majoris	Covestro	Pittsburgh	Pennsylvania	United States	Business
Christopher McCarthy	Jacobs Engineering Group Inc	Boston	Massachusetts	United States	Business
Lisa McIntosh	Woodard & Curran	Providence	Rhode Island	United States	Business
Jonathan Naile	Shell Oil Company	Houston	Texas	United States	Business

Table SI-4.1. Attendees at the 2019 Society for Environmental Chemistry and Toxicology (SETAC) Perfluoroalkyl and Polyfluoroalkyl substances (PFAS) Focus Topic Meeting who participated in the

Name	Organization	City/Town	State/Prov	Country	Sector
John Newsted	Ramboll	East Lansing	Michigan	United States	Business
Jeff Peterson	SLR International Corporation	West Linn	Oregon	United States	Business
Bradley Sample	Ecological Risk, Inc	Rancho Murieta	California	United States	Business
Ryan Sleeper	EHS Support LLC	Burlington	Vermont	United States	Business
Jamie Suski	EA Engineering, Science, and Technology, Inc., PBC	Hunt Valley	Maryland	United States	Business
Sagar Thakali	AECOM	Conshohocken	Pennsylvania	United States	Business
Sergio A. Villalobos	BP	Naperville	Illinois	United States	Business
Jeanmarie Zodrow	Arcadis US Inc	Highlands Ranch	Colorado	United States	Business
Jonathan Ali	New Hampshire Department of Environmental Services	Concord	New Hampshire	United States	Government
Richard Anderson	U.S Air Force	San Antonio	Texas	United States	Government
Gerald Ankley	U.S. Environmental Protection Agency	Duluth	Minnesota	United States	Government
Mike Aplin	Texas Commission on Environmental Quality	Austin	Texas	United States	Government
Tom Augspurger	U.S. Fish and Wildlife Service	Raleigh	North Carolina	United States	Government
JayneAnne Bond	AFCEC/CZTE	JBSA-Lackland	Texas	United States	Government
Christine Custer	U.S. Geological Survey	La Crosse	Wisconsin	United States	Government
Russell Erickson	U.S. Environmental Protection Agency	Duluth	Minnesota	United States	Government
Matthew Etterson	U.S. Environmental Protection Agency	Duluth	Minnesota	United States	Government
Jeff Frithsen	EPA/ORD/IOAA/NPD	Washington, DC	District of Columbia	United States	Government
Kathryn Gallagher	U.S. Environmental Protection Agency	Washington	Virginia	United States	Government
Nicholas Hayman	NIWC Pacific (Naval Information Warfare Center Pacific)	San Diego	California	United States	Government
Dale Hoff	U.S. Environmental Protection Agency	Duluth	Minnesota	United States	Government
Amanda Jarvis	U.S. Environmental Protection Agency	Washington D.C.	District of Columbia	United States	Government
Warren Johnson	NOAA/NCCOS/SDI/MAB	Silver Springs	Maryland	United States	Government
James Justice	U.S. Environmental Protection Agency	Washington	District of Columbia	United States	Government
Roman Kuperman	U.S. ARMY CCDC Chemical Biological Center	Aberdeen Proving Ground	Maryland	United States	Government
Guilherme Lotufo	U.S. Army Corps of Engineers	Vicksburg	Mississippi	United States	Government
Allison Narizzano	U.S. Army Public Health Center	Aberdeen Proving Ground	Maryland	United States	Government
Elena Nilsen	U.S. Geological Survey	Portland	Oregon	United States	Government
Jennifer Olker	U.S. Environmental Protection Agency	Duluth	Minnesota	United States	Government
Edward Perkins	U.S. Army Engineer Research and Development Center	Vicksburg	Mississippi	United States	Government
Kimberly Plank	US EPA	Philadelphia	Pennsylvania	United States	Government
Michael Quinn	U.S. Army Public Health Center	Aberdeen Proving Ground	Maryland	United States	Government
Marisol Sepulveda	Purdue University	West Lafayette	Indiana	United States	Government
Jason Speicher	NAVFAC Atlantic	Philadelphia	Pennsylvania	United States	Government
Jeff Steevens	U.S. Geological Survey	Columbia	Missouri	United States	Government
Judith Strawhecker	U.S. Army Corps of Engineers	Omaha	Nebraska	United States	Government
Timothy Thompson	SEE, LLC	Seattle	Washington	United States	Government
Christopher Ventaloro	North Carolina Department of Environmental Quality	Raleigh	North Carolina	United States	Government
Natalia Vinas	U.S. Army Corps of Engineers	Vicksburg	Mississippi	United States	Government
Laura Wood	NAVFAC Atlantic	Norfolk	Virginia	United States	Government
Ihor Hlohowskyj	Argonne National Laboratory	Lemont	Illinois	United States	Other
