# Supplementary material for: Assessing the Ecological Risks of Per‐ and Polyfluoroalkyl Substances: Current State‐of‐the Science and a Proposed Path Forward
Source: Environ Toxicol Chem. 2020 Nov 6;40(3):564–605. doi: 10.1002/etc.4869 (PMC7984443; doi:10.1002/etc.4869)
Supplement: Supplementary file 6 — Supporting information. [file ETC-40-564-s004.pdf]

Supplemental Information 5
Ankley et al. 2020
Assessing the Ecological Risks of Per- and Polyfluoroalkyl Substances: Current State-of-the Science and a Proposed Path Forward

Table SI-5.1: Summary of toxicity test conditions and characterization for selected perfluoroalkyl and polyfluoroalkyl substances (PFAS) aquatic toxicity studies from the primary literature.

Author	Year	PFAS	CAS	Measured	Background measured	Tissue residues	Solvent carrier	Species	Medium	Env Param	Test type	Guidance	Container	Endpoint
Hagenaars	2014	PFOS ≥98%	Y	N	N	N	N	*Danio*-ELS	Recon mod hard water	T-P-pH-C	SR	OECD 2013	48-well plate	Embryo morph, SBI
Vogs	2019	PFOS, PFHxS, PFBA >98%; PFOA >96%	Y	Y	N	Y	DMSO	*Danio*-ELS	E3	T-P-pH	S	OECD 2013	Glass	Embryo morph, toxicokinetics
Sant	2017	PFOS	N	N	N	N	DMSO	*Danio*-ELS	0.3X Danieau	pH	SR	NC	Glass	Embryo
Ankley	2005	PFOS >98%	N	Y	N	Y	N	*P. promelas*	Lake Superior water	T-P-pH-DO-A-H	FT	US EPA 2002	Glass	Repro, biochem
Phillips	2007	4:2, 6:2, 8:2, 10:2 FTCA and FTUCA >98%	N	Y	Y	N	N	*D. magna*	Recon hard water	T-P	S	Environment Canada 2000; ASTM 2001	PP	Survival, immobility
			N	Y	Y	N	N	*C. tentans*	ASTM recon	T-P	SR	ASTM 2001	PP	Survival, growth
			N	Y	Y	N	N	*L. gibba*	Hutner's	T-P	SR	ASTM 2001	PS	Frond number, biomass
Rodriguez-Jorquera	2019	PFOS >99%	Y	Y	Y	Y	TEG	*P. promelas* male	River	T-P	S	NC	Glass	Microarray
Shi	2017	6:2 FTCA >96%	Y	Y	Y	N	DMSO	*Danio*-ELS	Recon soft	T	SR	NC	6-well plate	Embryo morph, mort, physiol
Du	2009	PFOS >99%	N	N	N	N	DMSO	*Danio*-PLC	Tap	T-P	SR	NC	Glass	Adult biochem, ELS
Sant	2019	PFBS 97%	N	N	N	N	DMSO	*Danio*-ELS	0.3X Danieau	N	SR	OECD 2013	96-well plate; PS petri	Embryo, hatch, mort, SBI, physiol
Wu	2019	F-53B >99%	Y	Y	Y	Y	DMSO	*Danio*-ELS	E3	T	SR	OECD 1996	Glass	Embryo, BCF
Martinez	2019	PFOS ≥98%	Y	N	N	N	DMSO	*Danio*-ELS	Instant Ocean/RO	T-P-pH	SR	OECD 2013	6-well plate	Embryo morph, mort, SBI, physiol
Keiter	2012	PFOS >98%	Y	Y	Y	N	N	*Danio*-LC	Tap-DI	T-P-pH-DO-C-H	SR, FT	ISO 1996 OECD 2011	Glass	Embryo/adult
Han	2010	PFOS >98%	N	N	N	N	N	Swordtail	Tap-dechlor	T-P	SSR	NC	Glass	Adult biochem, ELS, molec, repro
Hoke	2012	6:2FTCA, 8:2FTCA, 6:2 FTUCA, 8:2FTUCA 5:3acid, 7:3acid, PFPeA, PFDA-97-99.8%	Y	Y/N	Y	N	N	*P. promelas*	Well	T-P-pH-DO	S, SR	ASTM 1988 OECD 1992	SS	ELS
				Y/N	Y	N	N	*O. mykiss*	Well	T-P-pH-DO		ASTM 1988, OECD 1992	SS	ELS
				Y/N	Y	N	N	*P. capitata*	AAAP nutrient medium	T-P-pH-DO	S	ASTM 1990, Miller et al. 1978	Glass	IC
				Y/N	Y	N	N	*D. magna*	Well	T-P-pH-DO	S	ASTM 1988	Glass	Immobility

A – alkalinity; BCF – bioconcentration factor; C – conductivity; DMSO – dimethyl sulfoxide; DO – dissolved oxygen; ELS – early life stage; FT – flow-through; H – hardness; IC – inhibitory concentration; NC – not cited; P – photoperiod; PP – polypropylene; PS – polystyrene; S – static; SBI – swim bladder inflation; SS – stainless steel; SR – static replacement; T – temperature; TEG – triethylene glycol
