# Supplementary material for: Assessing the Ecological Risks of Per‐ and Polyfluoroalkyl Substances: Current State‐of‐the Science and a Proposed Path Forward
Source: Environ Toxicol Chem. 2020 Nov 6;40(3):564–605. doi: 10.1002/etc.4869 (PMC7984443; doi:10.1002/etc.4869)
Supplement: Supplementary file 7 — Supporting information. [file ETC-40-564-s005.pdf]

Table SI-6.1 Overview of *in-vivo* and *in-vitro* studies of the toxicity of perfluoroalkyl and polyfluoroalkyl substances (PFAS) mixtures

Substances[1], variables	Test type	Response Site	Effect	Response	Author	Title	Source	Publication Year
Single and binary mixtures: PFOS, pentachlorophenol (PCP), atrazine, diuron	in vivo	*Scenedesmus obliquus.*	growth rate inhibition	PFOS **INCREASED** PCP toxicity and **DECREASED** atrazine and diuron toxicity	Liu W, Zhang YB, Quan X, Jin YH, Chen S	Effect of perfluorooctane sulfonate on toxicity and cell uptake of other compounds with different hydrophobicity in green alga	Chemosphere 75:405-409	2009
Single and binary, multicomponent mixtures: PFOA, PFOS, PFNA, PFDA, PFDoA, 8:2 FTOH	in vitro	Primary *Gobiocypris rarus* hepatocytes culture	Transcriptional profile analysis	**NO ADDITIVITY**	Wei Y, Shi X, Zhang H, Wang J, Zhou B, Dai J.	Combined effects of polyfluorinated and perfluorinated compounds on primary cultured hepatocytes from rare minnow (Gobiocypris rarus) using toxicogenomic analysis	Aquatic toxicology 95:27-36	2009
Single and binary, ternary mixtures: PFOS, triclosan, 2,4,6-trichlorophenol, gemfibrozil, bezafibrate	in vivo	Pseudokirchneriella subcapitata	growth rate inhibition	Binary mixtures exhibited **ANTAGONISM** at all effect levels. The addition of a third component induced a less ANTAGONISTIC or even **SYNERGISTIC** behaviour.	Boltes K, Rosal R, Garcia-Calvo E	Toxicity of mixtures of perfluorooctane sulphonic acid with chlorinated chemicals and lipid regulators	Chemosphere 86:24-29	2012
Single and binary, ternary, multicomponent mixtures: PFOS, PFOA, Hg, Cd, propylparaben, 2-(2,4-dichlorophenoxi) acetic acid, furazolidone	in vivo	Anabaena CPB4337	inhibition of constitutive luminescence	Binary PFOA and PFOS mixture showed an **ANTAGONISTIC** interaction<br>Multicomponent PFOA and PFOS mixture showed **SYNERGISM** at low/very low effect levels.<br>Multicomponent PFOA and PFOS mixture showed **ANTAGONISTIC** behavior at high effect levels<br>Both PFOA and PFOS interacted **ANTAGONISTICALLY** with both heavy metals	Rodea-Palomares I, Leganes F, Rosal R, Fernandez-Pinas F.	Toxicological interactions of perfluorooctane sulfonic acid (PFOS) and perfluorooctanoic acid (PFOA) with selected pollutants	J Hazard Mater 201-202:209-218.	2012
Single and binary mixtures: PFOS, PFOA	in vivo	*Danio rerio* (zebrafish) embryos	Lethality	complex interactive effects: from **ADDITION** to **SYNERGISTIC** effect, then to **ANTAGONISTIC** effect	Ding G, Zhang J, Chen Y, Wang L, Wang M, Xiong D, Sun Y.	Combined effects of PFOS and PFOA on zebrafish (Danio rerio) embryos	Arch Environ Contam Toxicol 64:668-675	2013
Single and multicomponent mixtures: PFHxS, PFOS, PFOA, PFNA, PFDA, PFUnA, PFDoA	in vitro	stably transfected MVLN cell line	Estrogen receptor (ER) and androgen receptor (AR) transactivity and aromatase enzyme activity	A mixture effect **MORE THAN ADDITIVE** was observed on androgen receptor (AR) function	Kjeldsen LS, Bonefeld-Jorgensen EC.	Perfluorinated compounds affect the function of sex hormone receptors	Environmental science and pollution research international 20:8031-8044	2013
Single, binary and multicomponent mixtures: PFOA, PFOS, PFHxS, PFNA	in vitro	COS-1 cells	PPARα activation	"less than" **ADDITIVITY** with a **POTENTIAL** for **ANTAGONISTIC** interactions.	Carr CK, Watkins AM, Wolf CJ, Abbott BD, Lau C, Gennings C.	Testing for departures from additivity in mixtures of perfluoroalkyl acids (PFAAs)	Toxicology 306:169-175	2013
Single and binary mixture: Pentachlorophenol (PCP), PFOA, PFOS	in vitro	HepG2 cell line	Cell viability, reduced (GSH) and oxidized (GSSG) glutathione contents, reactive oxygen species, mitochondrial membrane potential and permeability of cell membrane, biochemical assays	PFOS (or PFOA) displayed **SYNERGISTIC** effect with PCP to the cytotoxicity of human liver cells	Shan G, Ye M, Zhu B, Zhu L	Enhanced cytotoxicity of pentachlorophenol by perfluorooctane sulfonate or perfluorooctanoic acid in HepG2 cells	Chemosphere 93:2101-2107	2013
Binary mixture of PFOA and PFOS, PFHxA, PFHxS, PFNA	in vitro	COS-1 cells	PPARα activation	ADDITIVITY at low concentrations	Wolf CJ, Rider CV, Lau C, Abbott BD.	Evaluating the additivity of perfluoroalkyl acids in binary combinations on peroxisome proliferator-activated receptor-alpha activation	Toxicology 316:43-54	2014

Table SI-6.1 Overview of *in-vivo* and *in-vitro* studies of the toxicity of perfluoroalkyl and polyfluoroalkyl substances (PFAS) mixtures

Substances[1], variables	Test type	Response Site	Effect	Response	Author	Title	Source	Publication Year
Single and binary mixture PFOS and Cd at different pH	in vivo	*Limnodrilus hoffmeisteri*	Lethality and oxidative stress biomarkers	Lethality of Cd was decreased in the combined Cd / PFOS system **(ANTAGONISM)** Integrated Biomarker Response (IBR) of three biochemical parameters shows **ANTAGONISM** responses between Cd and PFOS	Qu R, Liu J, Wang L, Wang Z.	The toxic effect and bioaccumulation in aquatic oligochaete Limnodrilus hoffmeisteri after combined exposure to cadmium and perfluorooctane sulfonate at different pH values	Chemosphere 152:496-50	2016
Single and binary mixture PFOS and Cu at different pH	in vivo	*Limnodrilus hoffmeisteri*	Lethality and oxidative stress biomarkers	Lethality of Cu was decreased in the combined Cd / PFOS system **(ANTAGONISM)** Integrated Biomarker Response (IBR) of three biochemical parameters shows **SINERGISM** responses between Cu and PFOS	Meng L, Yang S, Feng M, Qu R, Li Y, Liu J, Wang Z, Sun C.	Toxicity and bioaccumulation of copper in Limnodrilus hoffmeisteri under different pH values: Impacts of perfluorooctane sulfonate	Hazard Mater 305:219-228	2016
Single and ternary combinations of TBBPA (tetrabromobisphenal A), TDCPP (tris (1,3-dichloro-2-propyl) phosphate), PFOA, DOPO (9,10-dihydro-9-oxa-10-phosphaphenanthrene-10-oxdie), PFBA	in vivo	*Danio rerio* (zebrafish) embryos	developmental toxicity data modeled by quantitative structure-toxicity relationship (QSTR) tool	all mixture combinations showed the **CONCENTRATION ADDITIONS** suggesting similar MOA for all studied chemicals along with strong non-interactions among them	Kar S, Ghosh S, Leszczynski J.	Single or mixture halogenated chemicals? Risk assessment and developmental toxicity prediction on zebrafish embryos based on weighted descriptors approach	Chemosphere 210:588-596	2018
Single, binary and multicomponent mixutes: 2,4-dichlorophenol (DCP), PFBA, PFOA, PFNA, PFBS, PFHxS, PFOS, 3M AFFF	in vivo	anaerobic digester microbial community	methane production, DCP degradation	PFAS and AFFF can **ALTER** the toxicity of DCP, **INHIBIT** DCP degradation, **DECREASE** the number of methanogens present, and **CHANGE** the microbial community structure.	Fitzgerald NJM, Temme HR, Simcik MF, Novak PJ.	Aqueous film forming foam and associated perfluoroalkyl substances inhibit methane production and Co-contaminant degradation in an anaerobic microbial community	Environmental science Processes & impacts 21:1915-1925	2019
Single and binary mixtures: PFOS, PFOA	in vivo	*Daphna magna*	Lethality, fecundity, grown	**SYNERGISM** in acute and chronic toxicity	Yang HB, Zhao YZ, Tang Y, Gong HQ, Guo F, Sun WH, Liu SS, Tan H, Chen F.	Antioxidant defence system is responsible for the toxicological interactions of mixtures: A case study on PFOS and PFOA in Daphnia magna	Total Environ 667:435-443	2019
